# A comprehensive evaluation of COVID-19 policies and outcomes in 50 countries and territories

**DOI:** 10.1038/s41598-022-12853-7

**Published:** 2022-05-25

**Authors:** Hsiao-Hui Tsou, Shu-Chen Kuo, Yu-Hsuan Lin, Chao A. Hsiung, Hung-Yi Chiou, Wei J. Chen, Shiow-Ing Wu, Huey-Kang Sytwu, Pau-Chung Chen, Meng-Hsuan Wu, Ya-Ting Hsu, Hsiao-Yu Wu, Fang-Jing Lee, Shu-Man Shih, Ding-Ping Liu, Shan-Chwen Chang

**Affiliations:** 1 Institute of Population Health Sciences, National Health Research Institutes, 35 Keyan Road, Zhunan, Miaoli County 350 Taiwan; 2 Graduate Institute of Biostatistics, College of Public Health, China Medical University, Taichung, Taiwan; 3 National Institute of Infectious Diseases and Vaccinology, National Health Research Institutes, Zhunan, Miaoli County Taiwan; 4 Department of Psychiatry, National Taiwan University Hospital, Taipei, Taiwan; 5 Department of Psychiatry, College of Medicine, National Taiwan University, Taipei, Taiwan; 6 Institute of Health Behaviors and Community Sciences, College of Public Health, National Taiwan University, Taipei, Taiwan; 7 School of Public Health, College of Public Health, Taipei Medical University, Taipei, Taiwan; 8 Master’s Program in Applied Epidemiology, College of Public Health, Taipei Medical University, Taipei, Taiwan; 9 Center for Neuropsychiatric Research, National Health Research Institutes, Zhunan, Miaoli County Taiwan; 10 Institute of Epidemiology and Preventive Medicine, College of Public Health, National Taiwan University, Taipei, Taiwan; 11 National Institute of Environmental Health Sciences, National Health Research Institutes, Zhunan, Miaoli County Taiwan; 12 Institute of Environmental and Occupational Health Sciences, National Taiwan University College of Public Health, Taipei, Taiwan; 13 Department of Public Health, National Taiwan University College of Public Health, Taipei, Taiwan; 14 Department of Environmental and Occupational Medicine, National Taiwan University Hospital and National Taiwan University College of Medicine, Taipei, Taiwan; 15 National Mosquito-Borne Diseases Control Research Center, National Health Research Institutes, Zhunan, Miaoli County Taiwan; 16 Taiwan Centers for Disease Control, Taipei, Taiwan; 17 Department of Health Care Management, National Taipei University of Nursing and Health Sciences, Taipei, Taiwan; 18 Department of Internal Medicine, National Taiwan University Hospital, Taipei, Taiwan

**Keywords:** Epidemiology, Health policy, Infectious diseases, Statistics

## Abstract

The COVID-19 pandemic struck the world unguarded, some places outperformed others in COVID-19 containment. This longitudinal study considered a comparative evaluation of COVID-19 containment across 50 distinctly governed regions between March 2020 and November 2021. Our analysis distinguishes between a pre-vaccine phase (March–November 2020) and a vaccinating phase (December 2020–November 2021). In the first phase, we develop an indicator, termed lockdown efficiency (LE), to estimate the efficacy of measures against monthly case numbers. Nine other indicators were considered, including vaccine-related indicators in the second phase. Linear mixed models are used to explore the relationship between each government policy & hygiene education (GP&HE) indicator and each vital health & socioeconomic (VH&SE) measure. Our ranking shows that surveyed countries in Oceania and Asian outperformed countries in other regions for pandemic containment prior to vaccine development. Their success appears to be associated with non-pharmaceutical interventions, acting early, and adjusting policies as needed. After vaccines have been distributed, maintaining non-pharmacological intervention is the best way to achieve protection from variant viral strains, breakthrough infections, waning vaccine efficacy, and vaccine hesitancy limiting of herd immunity. The findings of the study provide insights into the effectiveness of emerging infectious disease containment policies worldwide.

## Introduction

The COVID-19 (coronavirus disease 2019) pandemic unleashed extraordinary challenges on humanity. Governments implemented various policies aimed at containing the spread of infection and stabilizing their economies. Many institutions—including the Economist Intelligence Unit^1^, Oxford COVID-19 Government Response Tracker (OxCGRT)^2^, NLI Research Institute^3^, and Bloomberg^4^—have assessed global responses to the COVID-19 pandemic. However, evaluation criteria differ across these institutions (Supplementary Information 1–3), and their studies have had different focuses and produced varying evaluation results.

Here, we introduce a set of criteria for assessing virus-containment effectiveness, where the aim of containment is to minimize harm to societies and economies. The proposed criteria include several previously examined indicators, namely monthly cases per 100,000 members of a population (per capita), fatality rate, and GDP loss. Additionally, because OxCGRT's stringency index (SI)^2^ cannot reflect rigor accurately based on monthly case numbers (Supplementary Information 4), we propose a lockdown efficiency (LE) parameter, which relates stringency to incidence^5^ (Supplementary Information 5). We include the following five OxCGRT indicators of health-system policies^2^: public information campaigns, testing policy, contact tracing, facial coverings, and (from December 2020 onward) vaccination policy.

To track long-term impacts on health literacy and mental health, we collated data from Google searches for “wash hands”, “face mask”, and “insomnia” as surrogates for national population health literacy and mental health. Surveys on COVID-19–related mental health and health literacy are mostly based on self-reported, cross-sectional studies and rarely multinational^6^. However, population health literacy and mental health trends have been shown to be reflected in search volume trends^7,8^. The search engine analysis website Google Trends has been used for population mental-health surveillance as well as for long-term tracking of depression^9^ and suicide^10^.

The availability of COVID-19 vaccines cast new hope for curbing the pandemic. Until August 19, 2021, the World Health Organization had issued only emergency-use authorizations for COVID-19 vaccines^11^. By September 12, 2021, 42% of the worldwide population had received at least one dose of a vaccine^12^. Therefore, we incorporate vaccine-related indicators into our research.

We propose a set of ten indices, including four government policy and hygiene education (GP&HE) indicators and six vital health and socioeconomic (VH&SE) measures. Our research is divided into pre- and post-vaccine availability phases. For the first phase (March 2020 to November 2020), we focus on evaluating the appropriateness of the LE indicator, which we developed using linear mixed models to determine how each GP&HE indicator correlates with each VH&SE measure. In the second phase during which there was ongoing vaccine distribution (December 2020 to November 2021), we added vaccine-related indicators to enable more comprehensive containment performance analyses. We compared indicator scores for 50 distinctly governed territories.

## Methods

Our scoring system, modified from previous studies^2,3^, includes GP&HE indicators and VH&SE measures. The GP&HE indicators include: LE; health-system policies; health literacy indicators; and vaccine coverage of the population (December 2020 onward). The VH&SE measures include: one-month cases per capita; infection growth rate; fatality rate; GDP loss due to the pandemic; unemployment rate; and psychological impact (Table 1)^2,5,13–15^.

**Table 1:** Summary of 10 indicators included in the present analyses.

Concern indicators^a^	Description	Source
**Government policy & hygiene education**		
Lockdown efficiency	Average stringency index of the last 2 weeks of the month compared to the total incidence of COVID-19 of the first 2 weeks of the month	Our World in Data^5,^ OxCGRT^2^
Health-system policies	Average of public information campaigns, testing policy, contact tracing, facial coverings, and vaccination policy scores^b^	OxCGRT^2^
Health literacy	Google Trends value for “wash hands” and “face mask”	Google Trends^13^
Vaccine coverage of the population	People covered by vaccines	Our World in Data^5^, Bloomberg^15^
**Vital health & socioeconomic indicators**		
One-month cases per 100,000 members of the population (per capita)	Number of COVID-19 cases per capita in the past month	Our World in Data^5^
Infection growth rate (%)	Ratio of one-month cases to the total cumulative number of cases up until the previous month (%)	Our World in Data^5^
One-month case fatality rate (%)	Ratio of one-month cumulative deaths to one-month cumulative infections (%)	Our World in Data^5^
GDP loss	Difference in GDP annual growth rate between 2019 and 2020 or 2020 and 2021 over the same period	Trading Economics^14^
Unemployment rate	Difference in monthly/ seasonal unemployment rate between 2019 and 2020 or 2020 and 2021 during the same timeframe	Trading Economics^14^
Insomnia	Increase in Google searches for “insomnia”	Google Trends^13^

^a^All indicators are updated monthly except for GDP loss, which is updated quarterly, and the unemployment increase rate, which is updated monthly or quarterly.

^b^Vaccination policy included in December 2020 and later.

### GP&HE indicators

Government response is associated with COVID-19 incidence^2^. Our newly introduced indicator LE is calculated based on COVID-19 incidence data and the SI^2^, a factor that does not account for COVID-19 incidence. LE has a reasonable correlation with one-month cases per capita (Supplementary Information 4 and 5).

We developed another indicator called health-system policies, which is a simple average of the four OxCGRT indicators: public information campaigns, testing policy, contact tracing, and facial coverings^2^. Each of these indicators is derived from daily data, and thus could potentially be represented with monthly means or modes. We used monthly modes as the representative score for each month. From December 2020 onward, we added OxCGRT vaccination policy^2^ to the calculation of the health-system policies indicator.

Health literacy is beneficial to reducing the spread of infectious diseases^7^. Therefore, Google Trends were used to obtain search trends for “wash hands” and “face mask”, which act as surrogate indicators of health literacy^7^ (Supplementary Information 6).

For the second phase of the research (December 2020–November 2021), we added the aforementioned indicator people covered by vaccines^15^ by modifying the calculation of health-system policies to include vaccination policy. This indicator was calculated as the ratio of the number of vaccine doses administered to the number of doses required for full vaccination. For simplicity, we assumed that every individual in each country needs only two doses of vaccine.

### VH&SE measures

NLI Research Institute definitions of one-month cases per capita, infection growth rate, and fatality rate were adopted^3^. Unemployment rate and GDP loss were defined relative to the same timeframe in 2020–2021 and 2019–2020 (Table 1). We quantified change in searches for “insomnia” to indicate the extent of the pandemic’s impact on a population’s mental health. Searches for insomnia have been used to assess the mental impact of COVID-19^8,16,17^ more commonly than searches for anxiety, panic attack^17,18^, depression^8^, or suicide^8^.

### Correlation analyses

In phase one, linear mixed models were used to explore the relationship between each GP&HE indicator and VH&SE measure. Because these values vary over time and may affect each other, we conducted two-stage analyses (Supplementary Information 4). We tested LE and SI legitimacy^2^. In phase two, we calculated Spearman's rank correlation coefficients to determine whether the indicator “people covered by vaccines” correlates with three vital health indicators, namely one-month cases per capita, infection growth rate, and one-month case fatality rate.

### Scoring of GP&HE indicators and VH&SE measures

We ranked the performance of 50 countries and territories according to total indicator scores (Supplementary Information 7 and 8), where a higher score implies better containment performance. We graphed the score distribution (Supplementary Figs. S11-S31) to visualize the relationship between pre-pandemic baseline risk levels and monthly containment scores. We assigned all 50 countries and territories to low, medium, or high baseline risk categories and calculated monthly average containment scores for each risk level (Supplementary Information 9).

### Geographic comparison

We calculated monthly average containment scores for six geographic group and conducted two-sample t-tests and Mann–Whitney U-tests to compare containment performance between Latin American and non-Latin American countries and between Asian and non-Asian countries prior to vaccine introduction (Supplementary Information 10 and 11). Additionally, we compared mean containment performance scores before versus after inclusion of vaccine indicators (Fig. 2).

Statistical analyses were conducted via SAS software (version 9.4); *P* values were two-tailed with 0.05 significance level.

### Sensitivity analysis

We conducted sensitivity analysis to determine the robustness of our study results in relation to uncertainties in the indicator calculation methods and the inclusion/exclusion of Google Trends indicators. Nine scenarios were evaluated (e.g. daily score means vs. modes; see Supplementary Table S35). The results obtained with different methods of calculating indices are given in Supplementary Tables S36–S40. Revised calculations with the inclusion of Google Trends are reported in Supplementary Tables S41–S44. The results obtained with the main scenarios (as defined in Table 1) are shown in Supplementary Tables S45 and S46, with the former including Google Trends indicators and the latter excluding Google Trends indicators. For the sensitivity analysis, we compared: (1) data in each of Supplementary Tables S36–S40 to the results in Supplementary Table S46; (2) data in each of Supplementary Tables S41–S44 to the results in Supplementary Table S45; and (3) data in each of Supplementary Tables S36–S39 to data in respective Supplementary Tables S41–S44 (i.e., Supplementary Table S41 vs. Supplementary Table S36, Supplementary Table S42 vs. Supplementary Table S37; Supplementary Table S43 vs. Supplementary Table S38; Supplementary Table S44 vs. Supplementary Table S39).

### Ethical approval

The institutional review board of the National Health Research Institutes approved this study (EC1091110-E-R1).

## Results

### Comparison between LE and SI prior to vaccine availability

Two-stage linear mixed models revealed no reasonable relationship between OxCGRT’s SI and one-month cases per capita (Supplementary Information 4). For each unit increase in the SI in July 2020, the estimated one-month cases per capita in August 2020 increased by 7.83 people (*P* = 0.03; Supplementary Table S2). Similar trends are observable for other months, contradicting the expectation that an increase in government policies in month *s* should reduce case numbers in month *s* + 1.

For every unit of increase in LE (defined in Table 1) in month *s*, the estimated one-month cases per capita in month *s* + 1 decreases. For each unit of increase in LE in September and October 2020, one-month cases per capita in October and November 2020 decrease by 629.55 and 597.94 people, respectively (both *P* < 0.0001; Supplementary Table S3). Although the relationships between LE and one-month cases per capita are not statistically significant in other months, we observe a consistent downward trend in cases subsequent to lockdowns.

From our analyses, we deduce that our LE indicator is legitimate, and that the SI does not have a reasonable correlation with one-month cases per capita. Therefore, we use LE as an indicator in our subsequent evaluation.

Two stage-linear mixed models also show that the health-system policy in month *s* does impact fatality in month *s* + 1 within some periods of time. For every unit of increase in health-system policies in April and May 2020, fatality rates in May and June 2020 decrease by 7.84% and 3.86%, respectively (*P* < 0.05; Supplementary Table S4).

### COVID-19 containment effectiveness

**Figure 1:**
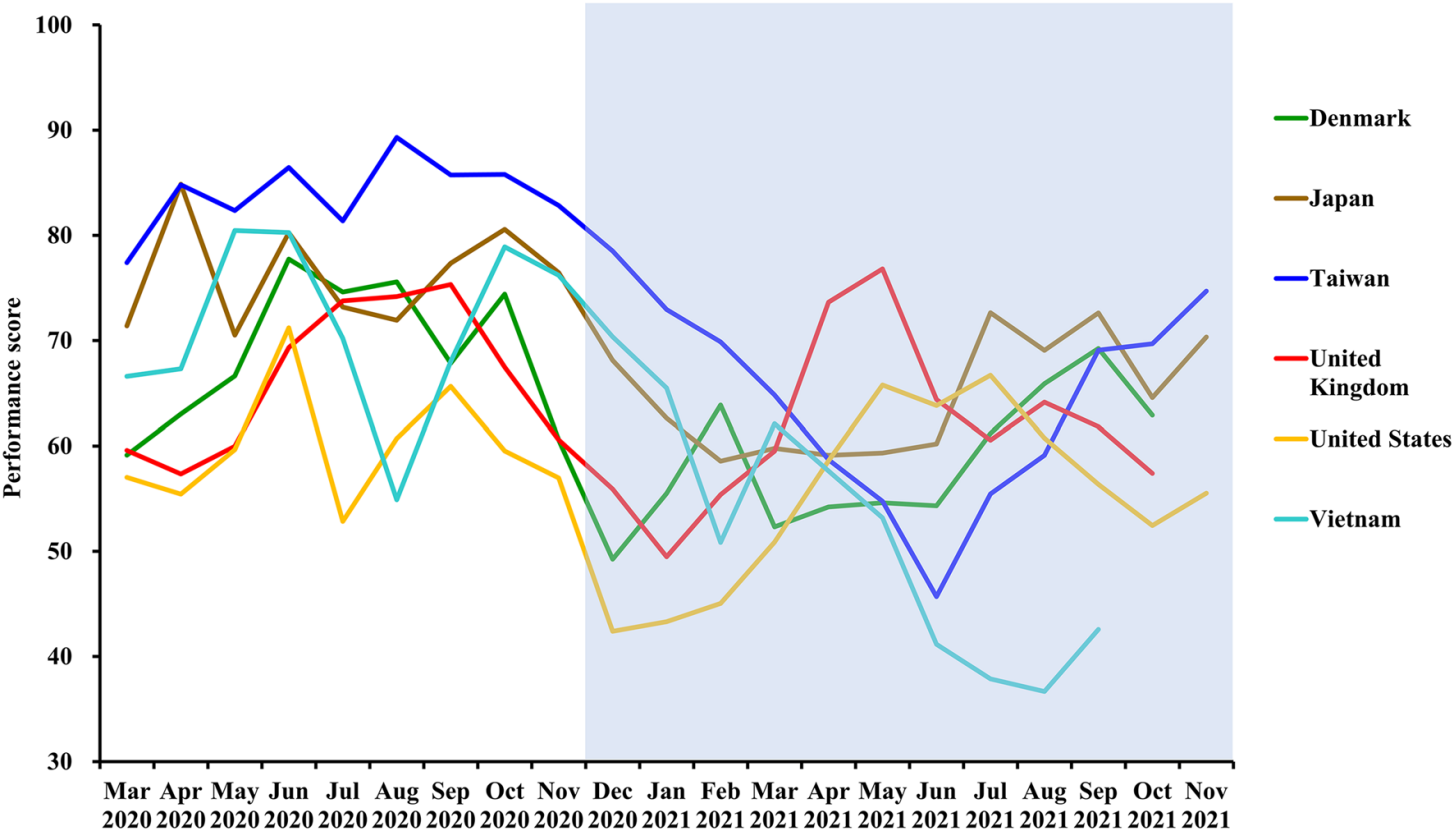
Performance scores of six countries analyzed from March 2020 to November 2021. A vaccination indicator was available only from December 2020 to November 2021 (shaded area). The calculation criteria include Google Trends data (i.e., health literacy and psychological impacts indexed by insomnia). Score range, 0–100, with 0 being the worst containment performance.

Performance scores for citizens’ health literacy and insomnia (Google Trends data) for six territories over the 21-month study period are shown in Fig. 1. All monthly COVID-19 containment rankings and scores from March 2020 to November 2021 are shown in Supplementary Tables S6 and S7 and in Supplementary Tables S13–S33 (Supplementary Information 8 and 12). The best performing countries and territories in 2020 are located mainly in Asia and Oceania, including Taiwan, the Republic of Korea (Korea), and New Zealand.

### Baseline risk

Low- and middle-baseline-risk territories are concentrated in Asia, including Vietnam, Korea, and Taiwan, whereas high-baseline-risk nations are located mostly in Europe, including France and the UK. Countries in the Americas span the three risk levels (Fig. 3a). In the first phase, most low- and middle-baseline-risk countries and territories achieved better containment than their high-risk counterparts. Some countries, such as New Zealand and Finland, were able to restrain COVID-19 transmission despite having high baseline risk levels (Fig. 3b, Supplementary Information 9 and Supplementary Fig. S32a).

### Geographical comparison prior to vaccine allocation

Prior to vaccine distribution, Latin America was severely impacted by the pandemic, while Oceania and Asia had good containment (Fig. 4a). Non-Latin American nations achieved better overall containment performance than their Latin American counterparts, which were severely affected. From April 2020 to September 2020, performance differed between these two groups, with and without Google Trends data inclusion (*P* < 0.027; Supplementary Information 10).

Asian governments outperformed their non-Asian counterparts in phase one (Supplementary Information 11). From March to November 2020, with the exception of July 2020, performance scores of Asian countries were higher than those of non-Asian countries, with and without Google Trends data inclusion (*P* < 0.039).

Asian countries performed dramatically better than non-Asian countries in LE and health-system policies early in the pandemic (April 2020 LE scores *P* = 0.007; March/April 2020 health-system policy scores *P* < 0.003). These results suggest that Asian governments enacted effective policies early (Supplementary Information 11).

### Scoring system robustness

Sensitivity analyses revealed that (1) changing indicator calculation methods and (2) adding Google Trends indicators altered overall rankings slightly (Supplementary Information 13).

### Performance after vaccine distribution

**Figure 2:**
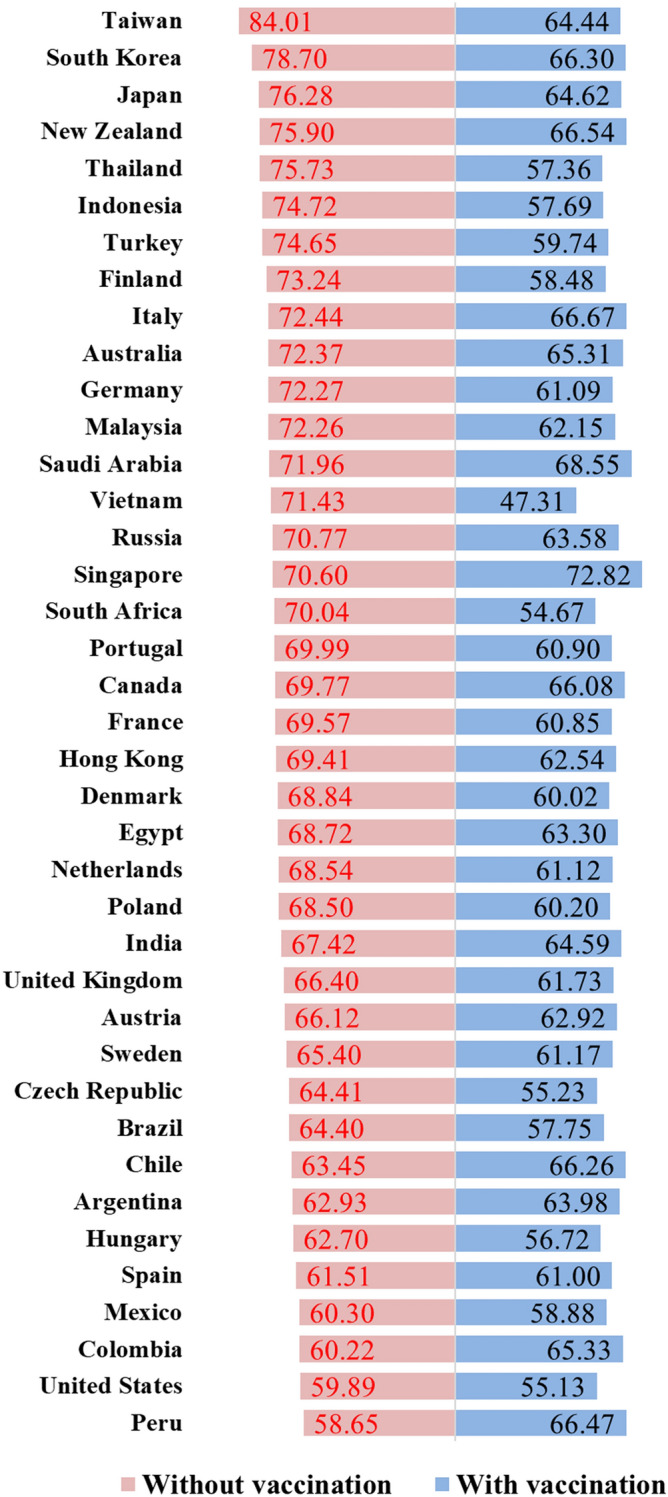
Average of performance scores in pre-vaccine and vaccine phases of the study. Nine and ten indicators (including Google Trends data) were used to obtain performance scores in pre-vaccine and vaccine phases of the study, respectively. Note that countries/territories with missing data were excluded.

**Figure 3:**
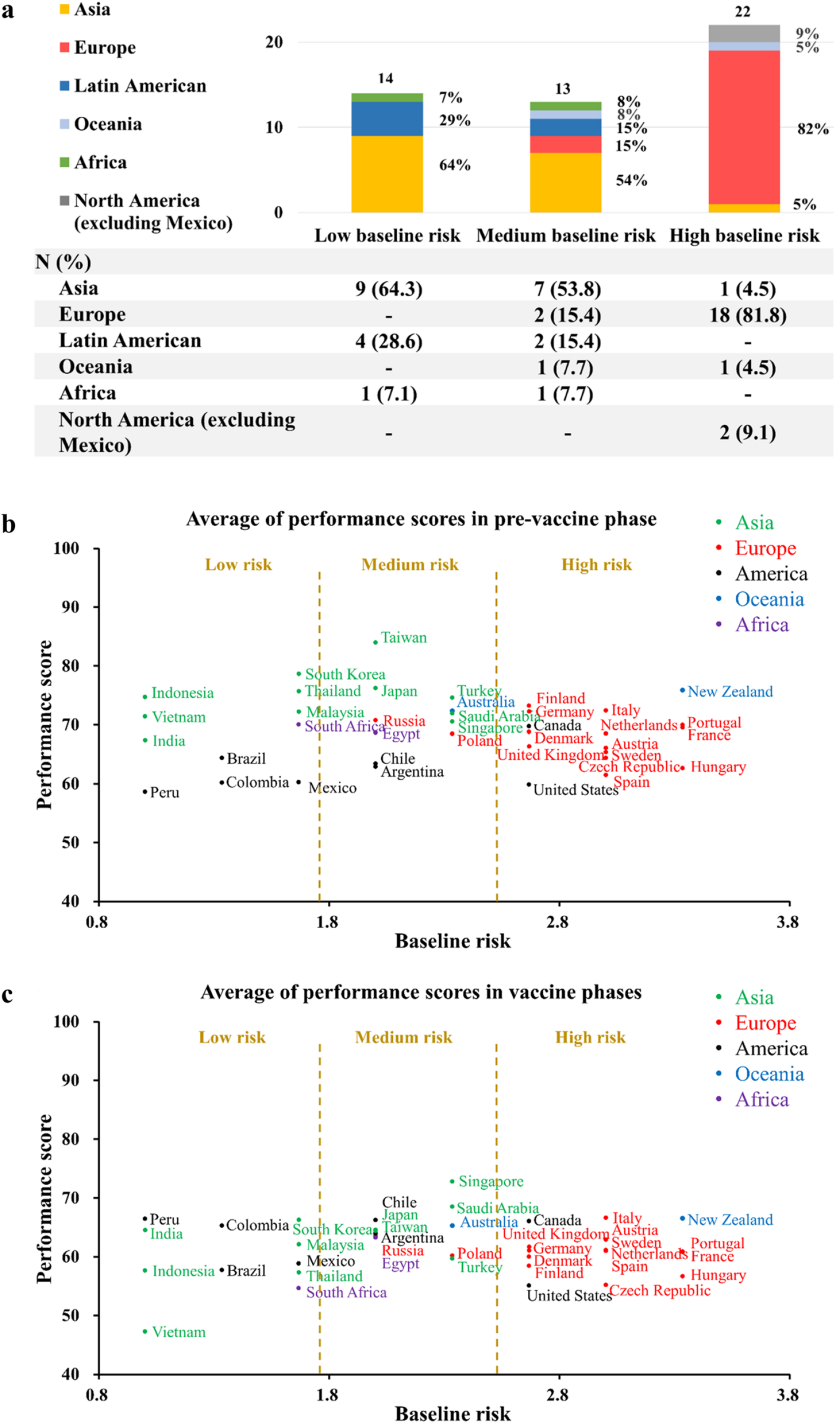
Distribution of baseline risk levels of countries and territories in each geographic region (**a**), and for containment scores (with Google Trends data) in pre-vaccine phase (**b**) and in vaccine phases (**c**). Note that most low- and medium-baseline-risk countries and territories are located in Asia, Latin America, and Africa, while high-baseline-risk areas are located mainly in Europe and North America (excluding Mexico). Note: Hong Kong is excluded due to insufficient data for calculating baseline risk.

**Figure 4:**
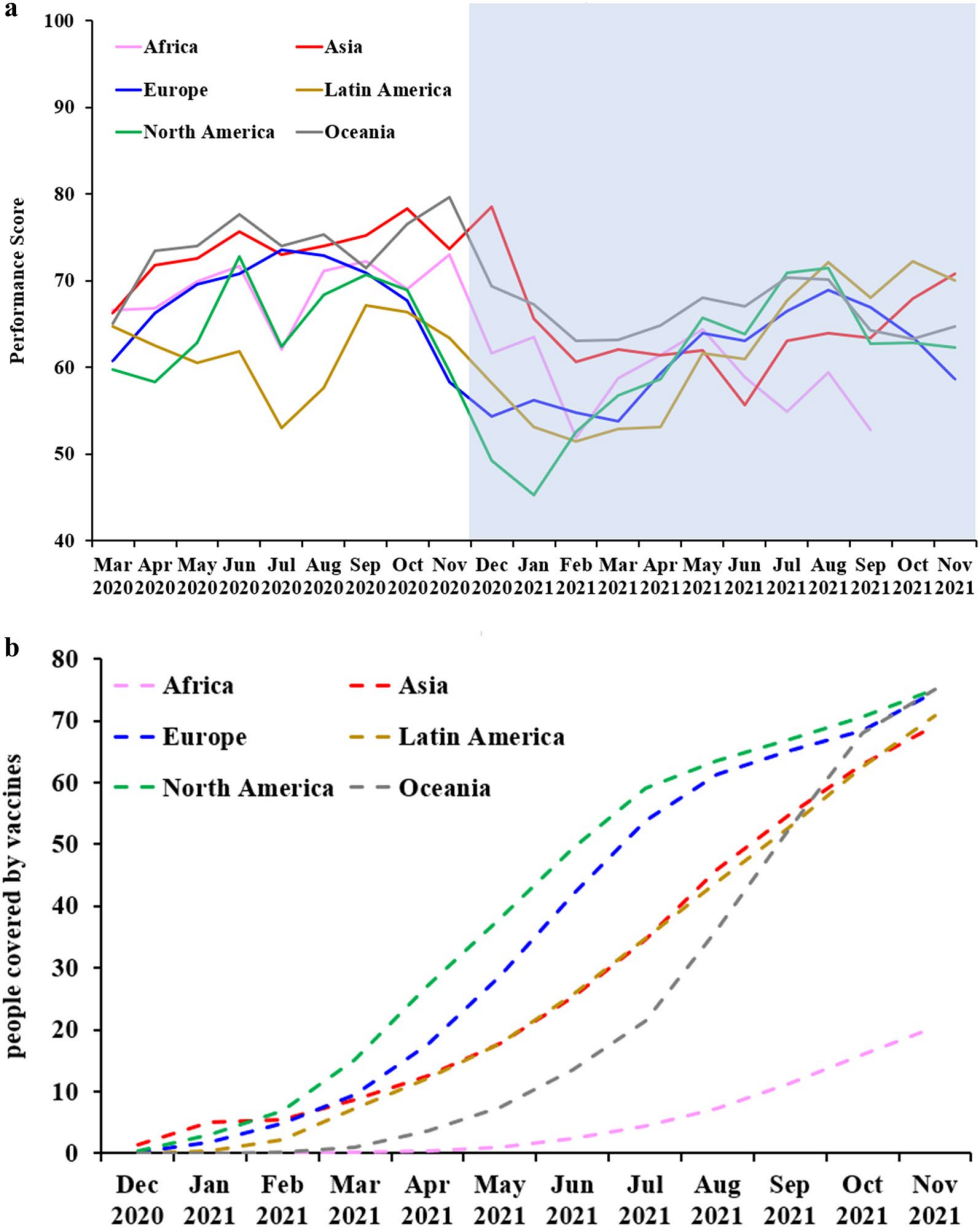
Comparison among geographic regions for containment effectiveness (**a**) and people covered by vaccines (**b**) from March 2020 to November 2021. Vaccination indicator available only from December 2020 to November 2021 (shaded area).

The best performers in 2020 were mostly in Asia and Oceania, including Taiwan, Korea, Japan, Thailand, and New Zealand. After vaccine indicators are included, the containment performance scores of these countries decrease. Inclusion of vaccine indicators from December 2020 onward results in higher containment performance scores in Singapore, Peru, Chile, Colombia, and Argentina (Figs. 2, 3b,c and 4). Among the six geographic regions analyzed, North American countries have the highest vaccine coverage. Their containment performance improves rapidly when vaccine indicators are included. Similar improvements are observed in Europe and Latin America (Fig. 4). Most high-baseline risk countries benefit from their high vaccination coverage (Supplementary Fig. S32).

### Vaccination coverage

In the first 6 months of vaccine distribution, one-month cases per capita in month t correlates positively with vaccine coverage in month t and subsequent months. With the exception of the relationship between one-month cases per capita in May 2021 and people covered by vaccines in August 2021, all remaining months correlated (*P* < 0.0246). In the first 3 months after vaccine distribution ensued (December 2020–February 2021), people covered by vaccines in month t correlated negatively with infection growth rate in the subsequent t + 3, t + 4, or t + 5 months (*P* < 0.0461). Starting in March 2021, people covered by vaccines correlates negatively with infection growth rate in respective subsequent months, though the relationship does not reach statistical significance for all months. A delayed negative correlation of people covered by vaccines with fatality rate can be seen. Starting from June 2021, the number of people vaccinated in month t correlated negatively with fatality rate in month t and subsequent months (*P* < 0.0394) (Supplementary Tables S47–S49).

## Discussion

To evaluate COVID-19 containment performance impartially, we developed and tested a comprehensive evaluation method that accounts for GP&HE indicators, VH&SE measures, and vaccination. LE captures a nation’s effectiveness in enacting measures according to recent incidence. As variants continue to emerge, a new normal, in which pharmaceutical and non-pharmaceutical interventions are considered should be established. Because it is unreasonable to expect life to normalize completely in the short term, we did not include the global normalcy index^19^ in our analyses.

Our sensitivity analysis showed that inclusion of the Google Trends indicator altered the overall rankings of the 50 examined countries and territories slightly. We included it for three reasons: Google Trends data reflect health literacy and impacts on mental health^7,8^; none of the global institutions assessing COVID-19 containment effectiveness have included public responses in their evaluation criteria; and we believe that public response is important for transmission control.

Prior to vaccine allocation, some countries and territories in Asia and Oceania were more successful at suppressing COVID-19 outbreaks than their European and American counterparts. They exhibited a relative weakening of containment performance after inclusion of the vaccine indicator (since December 2020), likely due, at least in part, to their lower vaccination coverage (Supplementary Information 12, Fig. 4).

New Zealand and Singapore emerged as the most stable performers. New Zealand has been steadfast in its fight against the vexatious COVID-19, increasing its LE to overcome spikes in infection (March/April 2020) and in fatality (May 2020). Successful containment has contributed to their economic stability during the first phase of pandemic (Supplementary Information 12). After New Zealand was invaded by the Delta variant in August 2021^20^, the number of confirmed cases and infection rates increased, and New Zealand responded with an accelerated vaccination strategy (Supplementary Information 12).

Although Singapore did not control rapid viral spread initially, they recovered from outbreaks well. Following a 5-month period of rising cases (April–August 2020), the Singapore government improved its health-system policies and LE, leading to decreased case numbers thereafter. Since January 2021, Singapore has been administering vaccines efficiently, making it a model nation for curbing COVID-19 (Supplementary Information 12). However, the surge in coronavirus infections and confirmed cases in Singapore since September 2021 may be related to Singapore’s “coexisting with COVID-19” strategy and the Delta variant outbreak^21,22^. Whether Singapore can achieve the goal of "live with the virus" has become the focus of global attention.

Changes in containment performance scores of six locations associated with notable performance, activities, or policies are shown in Fig. 1. Denmark and the UK have high vaccination coverage^23^. Denmark lifted restrictions on September 10, 2021^24^. Starting on July 19, 2021, the UK lifted most of its restrictions and removed social distancing recommendations^25^. In all US regions, restrictions have eased and people have returned to generally normal life^26^. The 2020 Olympic and Paralympic Games took place in Tokyo, Japan from July 23 to August 8 and from August 24 to September 5 of 2021, respectively^27^. Vietnam was Asia's top-performing economy in 2020^28^. Taiwan has been praised for its 253-day streak without local infections^29^.

Denmark's infection growth increased rapidly from September 2020 to January 2021, though their fatality rate remained low. Transmission was mitigated through strict health-system policies, improved LE, and mass vaccinations. Although the number of confirmed cases increased after May 2021, the fatality rate was low. The pandemic within Denmark appeared to be under stable control, but at the end of the study Denmark faced another new wave of outbreaks, the development of which remains to be seen (Fig. 1, Supplementary Information 12).

Containment performance in the UK was not satisfactory in phase one. The UK improved LE and health-system policies while administering vaccinations on a massive scale. These efforts enabled a gradual containment of COVID-19 transmission, which was suppressed by March 2021. There was a case/infection growth surge in June 2021 (Fig. 1, Supplementary Information 12).

The USA did not control infection well in the first phase, but its containment performance improved in the second phase. They have endured large numbers of confirmed cases with cascading regional surges (Fig. 1, Supplementary Information 12).

Japan adopted inclusive health-system policies. Although their confirmed cases increased during the 2021 Olympics, Japan had several prior waves of increased case numbers, including two in 2021 (January and May). Whether the increase in confirmed cases during the Olympics can be attributed to Olympic event gatherings remains to be clarified (Supplementary Information 12).

Vietnam performed well in early but experienced a short-term containment performance decline in August 2020. Vietnam controlled an outbreak that emerged in February 2021 successfully. Infection growth in Vietnam has been relatively high since May 2021, and was only slightly contained until October, but then increased again in November. Transmission in late summer 2021 may reflect Delta variant invasion under low vaccination coverage (Fig. 1, Supplementary Information 12).

Taiwan is an exemplary case. COVID-19 transmission was suppressed early owing to a rapid response by Taiwan's Central Epidemic Command Center with cooperation from local government agencies and the public^30^. Taiwan suffered a case surge under low vaccine coverage in May and June 2021. The Central Epidemic Command Center responded with intensive data gathering, expansive testing, quarantines, and stringent non-pharmaceutical policies^31^. Faced with the highly transmissible Delta variant, Taiwan suppressed case numbers within 2 ~ 3 months (Fig. 1, Supplementary Information 12).

While countries seek to increase vaccination coverage, vaccine effectiveness has been tested by the Delta variant and breakthrough infections. In addition to vaccination, non-pharmacological interventions, such as handwashing, masking, and physical distancing, remain important for preventing transmission.

### Public health implications

Our research suggests that the top performing nations tend to enact rigorous health system policies and locally-appropriate lockdown measures, especially early in the pandemic (Supplementary Information 12, and Supplementary Table S11). Asian countries’ border control policies were implemented earlier than non-Asian countries. In Asian and non-Asian countries, the median times to any border closure from the first reported case in China were 24.50 and 67.50 days, respectively (*P* = 0.0003), and the median times to any border closure from the first domestic case within each country were -10.50 and 8.00 days, respectively (*P* = 0.0027) (Supplementary Table S50, Supplementary Information 15). Notwithstanding, stringent lockdown measures should not be enacted without careful consideration, and lockdown policies should be adjusted in response to recent incidence. Flexibility in their implementation can yield containment with minimal harm to societies, economies, and mental health.

### Limitations

Several methodological limitations should be noted. First, we include unemployment rate and GDP loss, but not international aid, economic stimulus packages, or other fiscal measures. Second, Google Trends data do not represent a random sampling and may exclude vulnerable groups without internet access or those not actively searching. Third, ideally, only local infection rates should be considered to obtain precise LE measurements. However, because daily local case data are not fully available, we used incidence data inclusive of imported cases to determine LE scores. Therefore, minor inaccuracies in our LE data are expected (Supplementary Information 5).

## Conclusions

This longitudinal study considers government policy indicators, health literacy, health, and socioeconomic criteria between March 2020 and November 2021. For analyses of the data from the period of time preceding vaccine availability, we factor in nine GP&HE indicators and VH&SE measures of the overall COVID-19 containment performance in 50 countries and territories. Following introduction of vaccines, we incorporate relevant vaccine indicators. Our findings provide insights into the effectiveness of emerging infectious disease containment policies worldwide.

## Supplementary Information

Supplementary Information.

## Data Availability

All data generated or analysed during this study are included in this published article and its supplementary information files.

## References

[1] EIU. How well have OECD countries responded to the coronavirus crisis? *The Economist Intelligence Unit*; 2020. https://www.eiu.com/n/campaigns/oecd-countries-responded-to-the-coronavirus-crisis/. Accessed November 25, 2020.

[2] OxCGRT. Coronavirus government response tracker. *University of Oxford*; 2020. https://www.bsg.ox.ac.uk/research/research-projects/coronavirus-government-response-tracker. Accessed November 25, 2020.

[3] NLI Research Institute. The impact of coronavirus on national economy—which countries survive from the pandemic, the ranking of 49 countries. *NLI Research Institute*; Jul 3, 2020. https://www.nli-research.co.jp/report/detail/id=64863?site=nli. Accessed November 25, 2020.

[4] Chang, R., Hong, J., Varley, K. The best and worst places to be in the coronavirus era. *Bloomberg*; Nov 24, 2020. https://www.bloomberg.com/graphics/covid-resilience-ranking/. Accessed December 6, 2020.

[5] Our World in Data. COVID-19 dataset. *Our World in Data.* https://ourworldindata.org/coronavirus-source-data. Accessed December 15, 2020.

[6] Lin YH, Chen CY, Wu SI (2021). Efficiency and quality of data collection among public mental health surveys conducted during the COVID-19 pandemic: Systematic review. J. Med. Internet Res..

[7] Lin YH, Liu CH, Chiu YC (2020). Google searches for the keywords of “wash hands” predict the speed of national spread of COVID-19 outbreak among 21 countries. Brain Behav. Immun..

[8] Lin YH, Chiang TW, Lin YL (2020). Increased internet searches for insomnia as an indicator of global mental health during the COVID-19 pandemic: Multinational longitudinal study. J. Med. Internet Res..

[9] Yang AC, Huang NE, Peng C, Tsai S (2010). Do seasons have an influence on the incidence of depression? The use of an internet search engine query data as a proxy of human affect. PLoS ONE.

[10] Solano P (2016). A Google-based approach for monitoring suicide risk. Psychiatry Res..

[11] WHO (World Health Organization). Status of COVID-19 Vaccines within WHO EUL/PQ evaluation process. https://extranet.who.int/pqweb/sites/default/files/documents/Status_COVID_VAX_02July2021.pdf.

[12] Our World in Data. Coronavirus (COVID-19) Vaccinations. *Our World in Data*. https://ourworldindata.org/covid-vaccinations#. Accessed September 13, 2021.

[13] Google Trends. Suicide. *Google*. https://trends.google.com.tw/trends/explore?q=suicide&geo=TW. Accessed November 30, 2020.

[14] Trading Economics. https://tradingeconomics.com/countries. Accessed November 30, 2020.

[15] Bloomberg. More Than 5.71 Billion Shots Given: Covid-19 Tracker. *Bloomberg*. https://www.bloomberg.com/graphics/covid-vaccine-tracker-global-distribution/. Accessed September 13, 2021.

[16] Zitting K (2021). Google Trends reveal increases in internet searches for insomnia during the 2019 coronavirus disease (COVID-19) global pandemic. J. Clin. Sleep Med..

[17] Stijelja S, Mishara BL (2020). COVID-19 and psychological distress—changes in internet searches for mental health issues in New York during the pandemic. JAMA Intern. Med..

[18] Ayers JM (2020). Internet searches for acute anxiety during the early stages of the COVID-19 pandemic. JAMA Intern. Med..

[19] The Economist. The global normalcy index. *The Economist*. https://www.economist.com/graphic-detail/tracking-the-return-to-normalcy-after-covid-19?fbclid=IwAR0CVKqefRb1rZO6TKk82pjTQ6bDmuqSj7vPPMPj6uD5cMOH_T8kniJ-kxs. Accessed September 15, 2021.

[20] The Independent. New Zealand locks down as number of infections after first Delta Covid case leaps to seven. https://www.independent.co.uk/news/world/australasia/covid-nz-delta-lockdown-update-b1904561.html. Accessed January 18, 2022.

[21] Bloomberg. Singapore Confronts the Division and Fear That Come From Living With Covid. https://www.bloomberg.com/news/articles/2021-10-14/singapore-confronts-division-and-fear-bred-by-living-with-covid. Accessed January 18, 2022.

[22] Ben, Westcott. Delta variant outbreak threatens Singapore's 'living with Covid' model. *CNN*. https://edition.cnn.com/2021/09/07/asia/singapore-covid-19-restrictions-intl-hnk/index.html. Accessed January 18, 2022.

[23] Our World in Data. COVID-19 vaccine doses administered per 100 people. *Our World in Data*. https://ourworldindata.org/grapher/covid-vaccination-doses-per-capita?country=BRA~CHN~DEU~IND~IDN~ISR~JPN~MEX~TUR~ARE~GBR~USA~URY~OWID_WRL~DNK. Accessed September 27, 2021.

[24] The Washington Post. Denmark lifts all coronavirus restrictions and celebrates ‘a whole new era’. https://www.washingtonpost.com/world/europe/denmark-ends-covid-restrictions/2021/09/10/6d6a762e-1210-11ec-baca-86b144fc8a2d_story.html. Accessed September 10, 2021.

[25] HSE. Keeping workplaces safe as most coronavirus (COVID-19) restrictions are removed. *HSE*. https://www.hse.gov.uk/coronavirus/roadmap-further-guidance.htm. Accessed September 1, 2021.

[26] USA Today. COVID-19 restrictions: Map of COVID-19 case trends, restrictions and mobility. *USA Today*. https://www.usatoday.com/storytelling/coronavirus-reopening-america-map/. Accessed September 26, 2021.

[27] Tokyo 2020 Olympic Games. https://olympics.com/tokyo-2020/en/. Accessed September 28, 2021.

[28] CNBC. This is Asia’s top-performing economy in the Covid pandemic — it’s not China. *CNBC*. https://www.cnbc.com/2021/01/28/vietnam-is-asias-top-performing-economy-in-2020-amid-covid-pandemic.html. Accessed January 28, 2021.

[29] Taiwan Ministry of Health and Welfare. Timeline COVID-19. *Taiwan Ministry of Health and Welfare*; 2021. https://covid19.mohw.gov.tw/en/sp-timeline0-206.html. Accessed March 9, 2021.

[30] Wang CJ, Ng CY, Brook RH (2020). Response to COVID-19 in Taiwan: Big data analytics, new technology, and proactive testing. JAMA.

[31] Chen, C.J. Taiwan’s response to COVID-19 (Lecture). *Baltimore, MD: John Hopkins University; *2020. https://www.youtube.com/watch?v=ReI6ROZNbkk. Accessed March 9, 2021.

