# Supplementary material for: A comprehensive evaluation of COVID-19 policies and outcomes in 50 countries and territories
Source: Sci Rep. 2022 May 25;12:8802. doi: 10.1038/s41598-022-12853-7 (PMC9130690; doi:10.1038/s41598-022-12853-7)
Supplement: Supplementary file 1 — Supplementary Information. [file 41598_2022_12853_MOESM1_ESM.pdf]

A comprehensive evaluation of COVID-19 policies and outcomes in 50 countries and territories

Hsiao-Hui Tsou[1,2*], Shu-Chen Kuo[3], Yu-Hsuan Lin[1,4,5,6], Chao A. Hsiung[1], Hung-Yi Chiou[1,7,8], Wei J. Chen[9,10], Shiow-Ing Wu[1], Huey-Kang Sytwu[3], Pau-Chung Chen[11,12,13,14], Meng-Hsuan Wu[1], Ya-Ting Hsu[1], Hsiao-Yu Wu[1], Fang-Jing Lee[15], Shu-Man Shih[1], Ding-Ping Liu[16,17], Shan-Chwen Chang[18].

[1] Institute of Population Health Sciences, National Health Research Institutes, Zhunan, Miaoli County, Taiwan

[2] Graduate Institute of Biostatistics, College of Public Health, China Medical University, Taichung, Taiwan

[3] National Institute of Infectious Diseases and Vaccinology, National Health Research Institutes, Zhunan, Miaoli County, Taiwan

[4] Department of Psychiatry, National Taiwan University Hospital, Taipei, Taiwan

[5] Department of Psychiatry, College of Medicine, National Taiwan University, Taipei, Taiwan

[6] Institute of Health Behaviors and Community Sciences, College of Public Health, National Taiwan University, Taipei, Taiwan

[7] School of Public Health, College of Public Health, Taipei Medical University, Taipei,

Taiwan

[8] Master's Program in Applied Epidemiology, College of Public Health, Taipei Medical University, Taipei, Taiwan

[9] Center for Neuropsychiatric Research, National Health Research Institutes, Zhunan, Miaoli County, Taiwan

[10] Institute of Epidemiology and Preventive Medicine, College of Public Health, National Taiwan University, Taipei, Taiwan

[11] National Institute of Environmental Health Sciences, National Health Research Institutes, Zhunan, Miaoli County, Taiwan

[12] Institute of Environmental and Occupational Health Sciences, National Taiwan University College of Public Health, Taipei, Taiwan

[13] Department of Public Health, National Taiwan University College of Public Health, Taipei, Taiwan

[14] Department of Environmental and Occupational Medicine, National Taiwan University Hospital and National Taiwan University College of Medicine, Taipei, Taiwan

[15] National Mosquito-Borne Diseases Control Research Center, National Health Research Institutes, Zhunan, Miaoli County, Taiwan

[16] Taiwan Centers for Disease Control, Taipei, Taiwan

[17] Department of Health Care Management, National Taipei University of Nursing and Health Sciences, Taipei, Taiwan

[18] Department of Internal Medicine, National Taiwan University Hospital, Taipei, Taiwan

Supplementary Information

Contents

Supplementary Information 1. Economist Intelligence Unit, EIU[1] 10

Supplementary Table S1. Quality of response and risk factors in EIU *10*

Supplementary Information 2. Oxford COVID-19 Government Response Tracker (OxCGRT)[2] 11

Supplementary Information 3. NLI Research Institute[3] 11

Supplementary Information 4. Statistical methods 12

Supplementary Table S2. Impact of stringency index in month s on one-month cases per 100,000 members of the population in month s+1 .... *13*

Supplementary Table S3. Impact of lockdown efficiency in month s on one-month cases per 100,000 members of the population in month s+1 .... *14*

Supplementary Table S4. Impact of health-system policies in month s on the fatality rate in month s+1 .... *14*

Supplementary Information 5. Lockdown Efficiency 15

Supplementary Figure S1. Lockdown efficiency, a transformation of stringency index to incidence rate *16*

Supplementary Table S5. Percentage of countries with improved/worsened status in month s+1 as compared to their respective statuses in month s. *18*

Supplementary Figure S2. Lockdown efficiency in March 2020 .... *19*

Supplementary Figure S3. Lockdown efficiency in April 2020 .... *20*

Supplementary Figure S4. Lockdown efficiency in May 2020 .... *20*

Supplementary Figure S5. Lockdown efficiency in June 2020 .... *21*

Supplementary Figure S6. Lockdown efficiency in July 2020 .... *21*

Supplementary Figure S7. Lockdown efficiency in August 2020 .... *22*

Supplementary Figure S8. Lockdown efficiency in September 2020 .... *22*

Supplementary Figure S9. Lockdown efficiency in October 2020 .... *23*

Supplementary Figure S10. Lockdown efficiency in November 2020 .... *23*

Supplementary Information 6. Google Trends 24

Supplementary Information 7. Conversion of the ten indicators on “government policies & hygiene education” and “vital health & socioeconomic” measures 26

Supplementary Information 8. Overall COVID-19 containment ranking of 50 countries and territories from March 2020 to November 2021 29

Supplementary Table S6. Overall COVID-19 containment ranking (with Google Trends) *29*

Supplementary Table S7. Overall COVID-19 containment ranking (without Google Trends) *39*

Supplementary Information 9. Different baseline risk levels of 50 countries and territories and their respective COVID-19 containment scores (with Google Trends) from March 2020 to November 2021 50

Supplementary Figure S11. Different baseline risk levels of 50 countries and territories and their respective COVID-19 containment scores (with Google Trends) in March 2020. .... *51*

Supplementary Figure S12. Different baseline risk levels of 50 countries and territories and their respective COVID-19 containment scores (with Google Trends) in April 2020 .... *51*

Supplementary Figure S13. Different baseline risk levels of 50 countries and territories and their respective COVID-19 containment scores (with Google Trends) in May 2020 .... *52*

Supplementary Figure S14. Different baseline risk levels of 50 countries and territories and their respective COVID-19 containment scores (with Google Trends) in June 2020 .... *52*

Supplementary Figure S15. Different baseline risk levels of 50 countries and territories and their respective COVID-19 containment scores (with Google Trends) in July 2020 .... *53*

Supplementary Figure S16. Different baseline risk levels of 50 countries and territories and their respective COVID-19 containment scores (with Google Trends) in August 2020 .... *53*

Supplementary Figure S17. Different baseline risk levels of 50 countries and territories and their respective COVID-19 containment scores (with Google Trends) in September 2020 .... *54*

Supplementary Figure S18. Different baseline risk levels of 50 countries and territories and their respective COVID-19 containment scores (with Google Trends) in October 2020 .... *54*

Supplementary Figure S19. Different baseline risk levels of 50 countries and territories and their respective COVID-19 containment scores (with Google Trends) in November 2020 .... *55*

Supplementary Figure S20. Different baseline risk levels of 50 countries and territories and their respective COVID-19 containment scores (with Google Trends) in December 2020 .... *55*

Supplementary Figure S21. Different baseline risk levels of 50 countries and territories and their respective COVID-19 containment scores (with Google Trends) in January 2021 .... *56*

Supplementary Figure S22. Different baseline risk levels of 50 countries and territories and their respective COVID-19 containment scores (with Google Trends) in February 2021 .... *56*

Supplementary Figure S23. Different baseline risk levels of 50 countries and territories and their respective COVID-19 containment scores (with Google Trends) in March 2021 .... *57*

Supplementary Figure S24. Different baseline risk levels of 50 countries and territories and their respective COVID-19 containment scores (with Google Trends) in April 2021 .... *57*

Supplementary Figure S25. Different baseline risk levels of 50 countries and territories and their respective COVID-19 containment scores (with Google Trends) in May 2021 .... *58*

Supplementary Figure S26. Different baseline risk levels of 50 countries and territories and their respective COVID-19 containment scores (with Google Trends) in June 2021 .... *58*

Supplementary Figure S27. Different baseline risk levels of 50 countries and territories and their respective COVID-19 containment scores (with Google Trends) in July 2021 .... *59*

Supplementary Figure S28. Different baseline risk levels of 50 countries and territories and their respective COVID-19 containment scores (with Google Trends) in August 2021 .... *59*

Supplementary Figure S29. Different baseline risk levels of 50 countries and territories and their respective COVID-19 containment scores (with Google Trends) in September 2021 .... *60*

Supplementary Figure S30. Different baseline risk levels of 50 countries and territories and their respective COVID-19 containment scores (with Google Trends) in October 2021 .... *60*

Supplementary Figure S31. Different baseline risk levels of 50 countries and territories and their respective COVID-19 containment scores (with Google Trends) in November 2021 .... *61*

Supplementary Figure S32a. The comparison of performance scores among three levels of baseline risk from March 2020 to November 2021, with vaccination indicator only from December 2020 to November 2021 (marked light blue area). *61*

Supplementary Figure S32b. The comparison of people covered by vaccines among three levels of baseline risk from December 2020 to November 2021 .... *62*

Supplementary Information 10. Two-sample t-test and Mann-Whitney U test of comparison between Latin American and non-Latin American nations 63

Supplementary Table S8. Comparison of the overall containment effectiveness between Latin American and non-Latin American countries from March to November 2020 .... *65*

Supplementary Table S9. Comparison of government policy & hygiene education indicators between Latin American and non-Latin American countries from March 2020 to November 2020 .... *67*

Supplementary Table S10. Comparison of vital health & socioeconomic indicators between Latin American and non-Latin American countries from March 2020 to November 2020 .... *69*

Supplementary Information 11. Two-sample t-test and Mann-Whitney U test of government actions and COVID-19 containment effectiveness for Asian and non-Asian nations and territories 73

Supplementary Table S11. Comparison of government actions between Asian and non-Asian nations and territories from March 2020 to November 2020 .... *74*

Supplementary Table S12. Comparison of the containment effectiveness between Asian and non-Asian nations and territories from March 2020 to November 2020 .... *76*

Supplementary Information 12. Complete ranking and raw scores of 50 countries and territories, from March 2020 to November 2021 78

Supplementary Table S13. Complete ranking and raw scores of indicators in 50 countries and territories in March 2020 .... *78*

Supplementary Table S14. Complete ranking and raw scores of indicators in 50 countries and territories in April 2020 .... *81*

Supplementary Table S15. Complete ranking and raw scores of indicators in 50 countries and territories in May 2020 .... *84*

Supplementary Table S16. Complete ranking and raw scores of indicators in 50 countries in June 2020 .... *87*

Supplementary Table S17. Complete ranking and raw scores of indicators in 50 countries and territories in July 2020 .... *90*

Supplementary Table S18. Complete ranking and raw scores of indicators in 50 countries and territories in August 2020 .... *93*

Supplementary Table S19. Complete ranking and raw scores of indicators in 50 countries and territories in September 2020 .... *96*

Supplementary Table S20. Complete ranking and raw scores of indicators in 50 countries and territories in October 2020 .... *99*

Supplementary Table S21. Complete ranking and raw scores of indicators in 50 countries and territories in November 2020 .... *102*

Supplementary Table S22. Complete ranking and raw scores of indicators in 50 countries and territories in December 2020 .... *104*

Supplementary Table S23. Complete ranking and raw scores of indicators in 50 countries and territories in January 2021 .... *107*

Supplementary Table S24. Complete ranking and raw scores of indicators in 50 countries and territories in February 2021 .... *110*

Supplementary Table S25. Complete ranking and raw scores of indicators in 50 countries and territories in March 2021 .... *113*

Supplementary Table S26. Complete ranking and raw scores of indicators in 50 countries and territories in April 2021 .... *116*

Supplementary Table S27. Complete ranking and raw scores of indicators in 50 countries and territories in May 2021 .... *119*

Supplementary Table S28. Complete ranking and raw scores of indicators in 50 countries and territories in June 2021 .... *122*

Supplementary Table S29. Complete ranking and raw scores of indicators in 50 countries and territories in July 2021 .... *125*

Supplementary Table S30. Complete ranking and raw scores of indicators in 50 countries and territories in August 2021 .... *128*

Supplementary Table S31. Complete ranking and raw scores of indicators in 50 countries and territories in September 2021 .... *131*

Supplementary Table S32. Complete ranking and raw scores of indicators in 50 countries and territories in October 2021 .... *134*

Supplementary Table S33. Complete ranking and raw scores of indicators in 50 countries and territories in November 2021 .... *137*

Supplementary Information 13. Sensitivity analysis 140

Supplementary Table S34. List of 50 countries and territories included in the analysis *142*

Supplementary Table S35: Summary of sensitivity analysis for COVID-19 pandemic performance *144*

Supplementary Table S36. Rankings of 50 countries and territories March–November 2020, excluding indicators of health literacy and insomnia (one-way sensitivity analysis) *146*

Supplementary Table S37. Rankings of 50 countries and territories March–November 2020 by a three-month moving average, excluding indicators of health literacy and insomnia (two-way sensitivity analysis) *149*

Supplementary Table S38. Rankings of 50 countries and territories March–October 2020, excluding indicators of health literacy and insomnia (three-way sensitivity analysis) *152*

Supplementary Table S39. Rankings of 50 countries and territories March–October 2020, excluding indicators of health literacy and insomnia (two-way sensitivity analysis) *..................... 155*

Supplementary Table S40. Rankings of 50 countries and territories March–October 2020, excluding indicators of health literacy, insomnia, and unemployment rate (three-way sensitivity analysis) *... 158*

Supplementary Table S41. Rankings of 50 countries and territories March–November 2020 (two-way sensitivity analysis) *... 161*

Supplementary Table S42. Rankings of 50 countries and territories March–November 2020 by a three-month moving average (three-way sensitivity analysis) *.. 164*

Supplementary Table S43. Rankings of 50 countries and territories March–October 2020 (four-way sensitivity analysis) *... 167*

Supplementary Table S44. Rankings of 50 countries and territories March–October 2020 (three-way sensitivity analysis) *... 170*

Supplementary Table S45. Rankings of 50 countries and territories March 2020–July 2021 (with Google Trends)[a] *.. 173*

Supplementary Table S46. Rankings of 50 countries and territories March 2020–August 2021 (without Google Trends)[a] *.. 180*

Supplementary Information 14. Vaccination Coverage 188

Supplementary Table S47. Association between people covered by vaccines and one-month cases per 100,000 members of the population from December 2020 to August 2021. *............................. 188*

Supplementary Table S48. Association between people covered by vaccines and infection growth rate from December 2020 to August 2021. *.. 190*

Supplementary Table S49. Association between people covered by vaccines and one-month case fatality rate from December 2020 to August2021. *... 192*

Supplementary Information 15. COVID-19 government responses in border closure........................... 194

Supplementary Table S50. Comparison of the time to border closure among geographic regions. *.. 194*

Supplementary Information 1. Economist Intelligence Unit, EIU[1]

Economist Intelligence Unit (EIU) ranks 21 countries in terms of "quality of response" and "risk factors". The "quality of response" consists of three indicators (1) the number of people tested for COVID-19, (2) provision of non-COVID-19 health care, and (3) death rates for COVID-19. The "risk factors" are (1) obesity prevalence, (2) the share of the population over the age of 65, and (3) international arrivals. Supplementary Table S1 displays the definitions of the "quality of response" indicators and "risk factors". By weighting the death rate as four and the other five indicators as one, the EIU constructs the score for each nation by averaging the six indicators.[1]

***Supplementary Table S1*. Quality of response and risk factors in EIU**

**Quality of response**	**Score**
Tests (tests per million people)	
<10,000	1
10,000–20,000	2
20,000–50,000	3
>50,000	4
Provision of non-COVID-19 healthcare (share of cancer-related surgeries cancelled)	
>50%	1
40–50%	2
30–40%	3
<30%	4
Death rate (excess deaths per million people)	
>600	1
400–600	2
200–400	3
<200	4
**Risk factors**	
Obesity prevalence (share of obese people, age-standardized)	
<20%	1
20–25%	2
25–30%	3
>30%	4
Share of the population aged 65+ (percentage of population aged 65 and over)	
<10%	1
10–15%	2
15–20%	3

>20%	4
International arrivals (number of international arrivals as a share of the population)	
<40%	1
40–70%	2
70–100%	3
>100%	4

Supplementary Information 2. Oxford COVID-19 Government Response Tracker (OxCGRT)[2]

The Oxford COVID-19 Government Response Tracker (OxCGRT)[2] provides a systematic way of tracking government responses to COVID-19 in over 180 nations. The data is characterized into indicators that aggregate various measures of government responses, including containment and closure policies, economic policies, health-system policies and miscellaneous policies.[2] These indicators aim to identify each government action, and its intensity and effectiveness in curbing the rate of infection. Data is collected and updated by a team of over 100 Oxford students, alumni, staff, and project partners.

Supplementary Information 3. NLI Research Institute[3]

The NLI Research Institute evaluates each country's performance of epidemic preparedness and response in two domains: health and economic outcomes.[3] The indicators and indexes that are considered include the (1) cumulative number of COVID-19 cases, (2) infection growth rate, (3) fatality rate, and (4) GDP loss due to the pandemic. The cumulative number of COVID-19 cases is defined as the cumulative number of COVID-19 infections per 10,000 members of population, which measures the prevalence rate of the epidemic. The growth rate of the infection refers to the ratio of the number of new COVID-19 infections to the cumulative number of infections in the past two weeks, which is used to assess the development of the epidemic. The fatality rate is the ratio of the cumulative number of COVID-19 deaths to the cumulative number of COVID-19 infections, which is an indicator of whether a place is effective in treating infected people and preventing COVID-19 deaths. GDP loss is the year-to-year increase/decrease of the annual GDP growth rate before (2019) and during (2020) the pandemic.[3]

Supplementary Information 4. Statistical methods

“Government policy & hygiene education” and “vital health & socioeconomic” measures are dynamic, meaning that they would vary over time and affect each other. We use linear mixed models to explore the relationship between “government policy & hygiene education” and “vital health & socioeconomic” measures in a two-stage method.

Let Y_{it} denote the outcome at the tth time point for the ith country. Then the model can be written as follows:

$$Y_{it} = \beta_0 + b_{i0} + \beta_1 X_{i,t-1} + \beta_2 time_{it} + \beta_3 X_{i,t-1} \times time_{it} + \varepsilon_{it}\,,$$

where $X_{i,\,t\text{-}1}$ denotes the predicted variables at the previous time point t-1. The selection of the lag value depends on the correlation coefficient, and a lag of one is chosen. The variable $time_{it}$ is a categorical variable, which represents the observation time (month) of the tth observation for the ith country. $X_{i,t-1} \times time_{it}$ are the interaction terms of the variables $X_{i,\,t\text{-}1}$ and $time_{it}$. If the interaction term is not significant, the interaction term is removed from the model. The parameter β_0 is a fixed-effect intercept term; the parameter b_{i0} is a random-effect intercept term, which represents the difference in intercept for the ith country from the average intercept. The parameters β1, β2, and β3 are fixed-effect coefficients, which are identical for all countries. The error term ε_{it} is the error for the ith country at the tth time point. The errors for country i are assumed to be multivariate normally distributed.

The steps of the two stages are as follows:

“Stage 1”: Create fitted values from a linear mixed model, where “government policy & hygiene education at time point t” are the outcome variables and “vital health & socioeconomic measures at time point t-1” are predictors.

“Stage 2”: Use stage 1 fitted values as the predictors to create a model of the outcome variable. The outcome variables refer to “vital health & socioeconomic measures at time t+1”.

Supplementary Table S2 analyzes the impact of OxCGRT's “stringency index” in month s on the one-month cases per 100,000 members of the population in month s+1. For every unit of increase in stringency index in July 2020, the one-month cases per 100,000 members of the population in August 2020 increase by 7.83 people, which reaches statistical significance (*P* = 0.03). However, this result does not meet with our expectation that a higher stringency index in month s, meaning government policies should be more rigorous, should lead to a lower one-month cases per 100,000 members of the population in the following month (s+1). In addition, for every unit of increase in the stringency index in

October 2020, the one-month cases per 100,000 members of the population in November 2020 decrease by 14.53 people, which reaches statistical significance (*P* = 0.002). Due to the inconsistency of the outcome produced by utilizing the stringency index, we determine that the stringency index does not have a strong correlation with one-month cases per 100,000 members of the population.

Supplementary Table S3 analyzes the impact of lockdown efficiency in month s on the one-month cases per 100,000 members of the population in month s+1. For every unit of increase of lockdown efficiency in September 2020, the one-month cases per 100,000 members of the population in October 2020 decrease by 629.55 people, which reaches statistical significance (*P* < 0.0001). Similarly, for every unit of increase of lockdown efficiency in October 2020, the one-month cases per 100,000 members of the population in November 2020 decrease by 597.94 people, which also reaches statistical significance (*P* <0.0001). Although the relationship between lockdown efficiency in month s and one-month case per 100,000 members of the population in month s+1 does not reach statistical significance in other periods, we still observe a trend that for every unit of increase in lockdown efficiency in month s, the one-month cases per 100,000 members of the population decrease in the following month (s+1).

Supplementary Table S4 presents the impact of health-system policies in month s on the fatality rate in month s+1. For every unit of increase in health-system policies in April, the fatality rate in May 2020 decreases by 7.84%, which reaches statistical significance (*P* < 0.0001). Similarly, for each unit of increase of health-system policies in May 2020, the fatality rate in June 2020 decreases by 3.86%, which is also statistically significant (*P* = 0.03).

Supplementary Table S2. **Impact of stringency index in month s on one-month cases per 100,000 members of the population in month s+1**

**Parameters**	**Estimate**	**Standard Error**	***P* value**
Stringency index, s=4	1.26	4.38	0.77
Stringency index, s=5	4.35	4.18	0.30
Stringency index, s=6	6.87	3.94	0.08
Stringency index, s=7	7.83	3.69	0.03
Stringency index, s=8	6.87	4.22	0.10
Stringency index, s=9	-5.48	4.10	0.18
Stringency index, s=10	-14.53	4.75	0.002

Supplementary Table S3. **Impact of lockdown efficiency in month s on one-month cases per 100,000 members of the population in month s+1**

**Parameters**	**Estimate**	**Standard Error**	***P* value**
Lockdown efficiency, s=4	-69.78	113.20	0.54
Lockdown efficiency, s=5	-61.25	112.48	0.59
Lockdown efficiency, s=6	-56.12	116.49	0.63
Lockdown efficiency, s=7	-46.95	116.57	0.69
Lockdown efficiency, s=8	-142.91	113.53	0.21
Lockdown efficiency, s=9	-629.55	118.97	<0.0001
Lockdown efficiency, s=10	-597.94	128.28	<0.0001

Supplementary Table S4. **Impact of health-system policies in month s on the fatality rate in month s+1**

**Parameters**	**Estimate**	**Standard Error**	***P* value**
Health-system policies, s=4	-7.84	1.74	<0.0001
Health-system policies, s=5	-3.86	1.74	0.03
Health-system policies, s=6	-0.21	1.82	0.91
Health-system policies, s=7	0.77	1.80	0.67
Health-system policies, s=8	1.23	1.78	0.49
Health-system policies, s=9	0.53	1.93	0.78
Health-system policies, s=10	-0.01	2.07	0.99

Supplementary Information 5. Lockdown Efficiency

The Oxford COVID-19 Government Response Tracker (OxCGRT) collects publicly available information on 24 indicators of government responses across the following four aspects: (1) containment and closure, (2) economic response, (3) health systems, and (4) vaccine policies.[2] These 24 indicators can also be aggregated into four common indices: the (1) government response index, (2) containment and health index, (3) economic support index, and (4) stringency index; each of the four indices is reported as a number between 0 and 100 by the formula provided by OxCGRT.[2] The stringency index is one of the policy indices measured by the OxCGRT; the indicators used for calculating the "stringency index" include school closing (C1), workplace closing (C2), cancel public events (C3), restrictions on gatherings (C4), close public transport (C5), stay at home requirements (C6), restrictions on internal movement (C7), international travel controls (C8), and public information campaign (H1).[2] Stringency index records the strictness of "lockdown style" policies that primarily restrict people's behavior.[2] Nevertheless, the stringency index should not be interpreted as a measure of the appropriateness or effectiveness of a government's response. The COVID-19 pandemic has caused many governments to implement lockdowns. Although lockdowns may help control the spread of the virus, they may also lead to mental health problems and economic damage. Therefore, the appropriateness of the implementation of various strictness levels of lockdown policies should consider the amount of COVID-19 cases in that particular nation and territory. Because of the trade-off between the stringency index and the incidence of COVID-19, we combine the two into a new indicator, "lockdown efficiency", to compare the effectiveness of lockdown across countries; the incidence of COVID-19 for the first two weeks of the month and the average stringency index of the last two weeks of the month are used to present this (Supplementary Figure S1). Supplementary Figure S1 is inspired by the figure produced by Fernández-Villaverde & Jones.[4] The incidence of COVID-19 is calculated based on newly confirmed cases per million population.

In Supplementary Figure S1, we focus on the correlation between the average "stringency index" of the last two weeks of the month and the total "incidence of COVID-19" of the first two weeks of the month for a deeper observation of "lockdown efficiency". We use the average "stringency index" of the last two weeks of the month and the total "incidence of COVID-19" of the first two weeks of the month to illustrate the subtle correlations that occur in the pandemic. There can be a positive correlation between the stringency index and the incidence of COVID-19. The lower the incidence, the less that stringent policy measures are required. The lower-left corner of Supplementary Figure S1 is the quadrant with the best outcomes. Good policies mean that countries in this location would have experienced comparatively few COVID cases as a fraction of their populations but have simultaneously kept economic losses relatively low. Conversely, in the adjacent quadrant, the higher the incidence, the more that stringent policy measures are required. This is also reasonable. Thus, the countries located in the lower-left corner of Supplementary Figure S1 are evaluated as having the best performance and are given four points,

while the countries located in the upper-right corner of Supplementary Figure S1 are evaluated as having the second-best performance and are given three points.

A strict policy can abruptly shut down the economy, which increases economic losses and mental health problems. To contain the spread of COVID-19 but still maintain a healthy economy, the government must carefully consider when to implement various policies. In the upper-left corner of Supplementary Figure S1, the incidence rate is low but stricter policy measures are implemented. In this case, such measures would unnecessarily increase mental health problems and losses in economic activity, given the low severity of the health crisis the country faces. However, in the adjacent quadrant, the high incidence rate does not lead to more rigorous policy measures, leading to the worst outcomes. Policy mistakes are likely responsible for poor performance in containing the virus. Thus, the countries located in the upper-left corner of Supplementary Figure S1 are given two points, while the countries located in the lower-right corner of Supplementary Figure S1 are evaluated as having the worst performance and are given one point.

Supplementary Figure S1. **Lockdown efficiency, a transformation of stringency index to incidence rate**

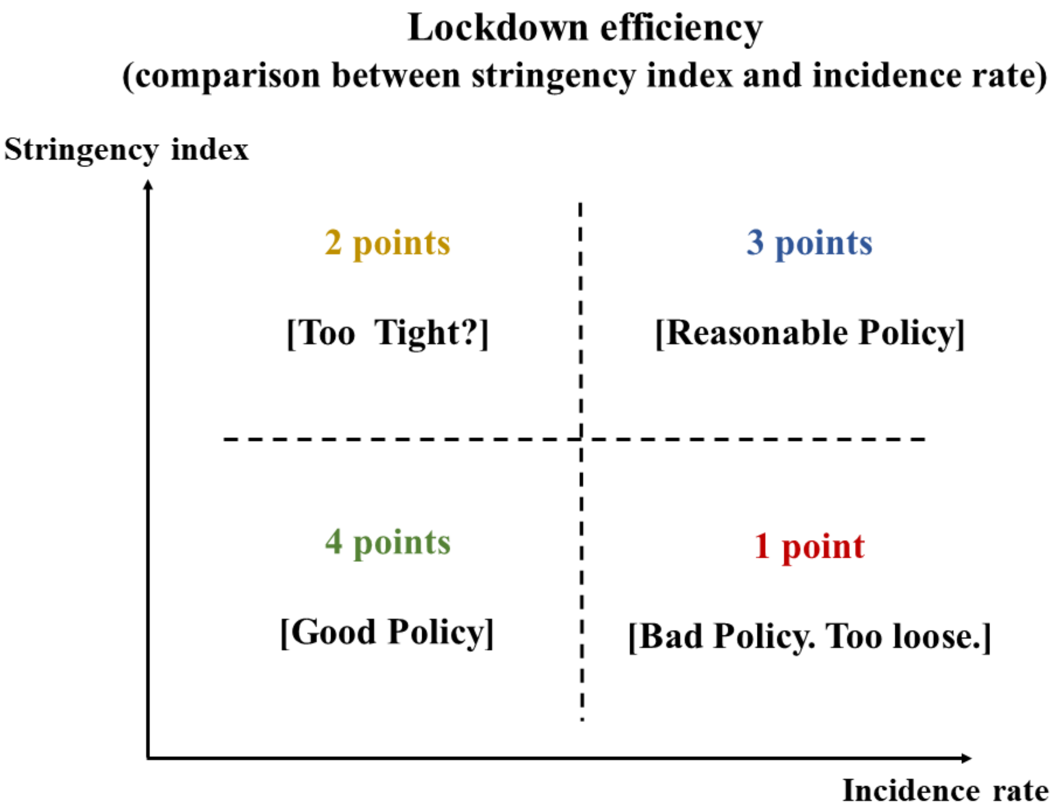

Note: Supplementary Figure S1 is inspired by the figure produced by Fernández-Villaverde & Jones.[4]

Now, we use real data to show why the countries in the upper-left corner of Supplementary Figure S1

are given two points but the countries in the upper-right corner are given three points. First, we come up with a new measurement, “status”, to indicate the decrease/increase of a country’s number of confirmed cases in a two-month period. If a country has a lower number of newly confirmed cases this month (s+1) as compared to the previous month (s), then the “status” of this country is defined as “better”. Conversely, the “status” of this country is defined as “worse”. Supplementary Table S5 presents the percentage of countries that became better or worse in month s+1 when the countries are in the upper-left corner or upper-right corner of Supplementary Figure S1 in month s. For example, 33% of the countries located in the upper-right corner of Supplementary Figure S1 in March improved in April, while only 10% of countries located in the upper-left corner in March improved in April. In Supplementary Table S5, we find that the countries located in the upper-right corner of Supplementary Figure S1 in month s have a higher chance to improve in month s+1 than the countries located in the upper-left corner in month s. Thus, it is reasonable that the countries in the upper-left corner of Supplementary Figure S1 are given two points while the countries in the upper-right corner are given three points.

Supplementary Table S5. **Percentage of countries with improved/worsened status in month s+1 as compared to their respective statuses in month s.**

"Lockdown efficiency" in month s	Status in this month	Apr	May	Jun	Jul	Aug	Sep	Oct	Nov
**Upper-right corner of Supplementary Figure S1**	Better	33%	64%	60%	55%	67%	71%	47%	60%
	Worse	67%	36%	40%	45%	33%	29%	53%	40%
**Upper-left corner of Supplementary Figure S1**	Better	10%	14%	30%	0%	43%	55%	30%	30%
	Worse	90%	86%	70%	100%	57%	45%	70%	70%

Supplementary Figures S2–S10 present "lockdown efficiency" as the average stringency index of the last two weeks of the month compared to the two-week total incidence of COVID-19 of the first two weeks of the month. The horizontal axis is the total "incidence of COVID-19". The vertical axis shows the average "stringency index". All countries considered are marked in the graphs as data points. We present the dynamics of how each country moves around this plane over time. One can divide the graph into four quadrants, based on higher versus lower incidence of COVID-19 and on higher versus lower value of the "stringency index".

Supplementary Figure S2. **Lockdown efficiency in March 2020**

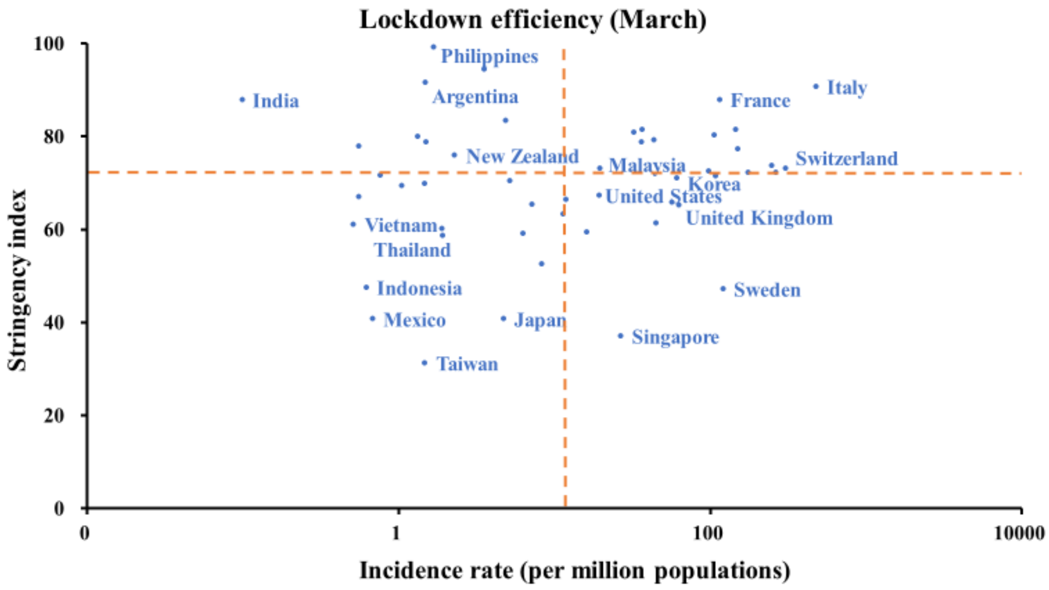

Supplementary Figure S3. **Lockdown efficiency in April 2020**

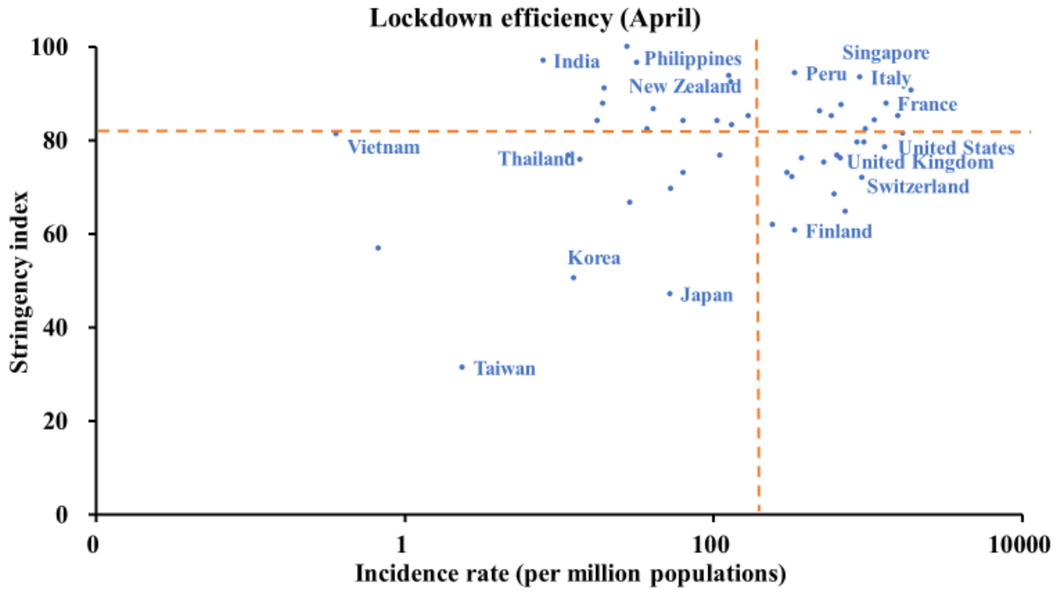

Supplementary Figure S4. **Lockdown efficiency in May 2020**

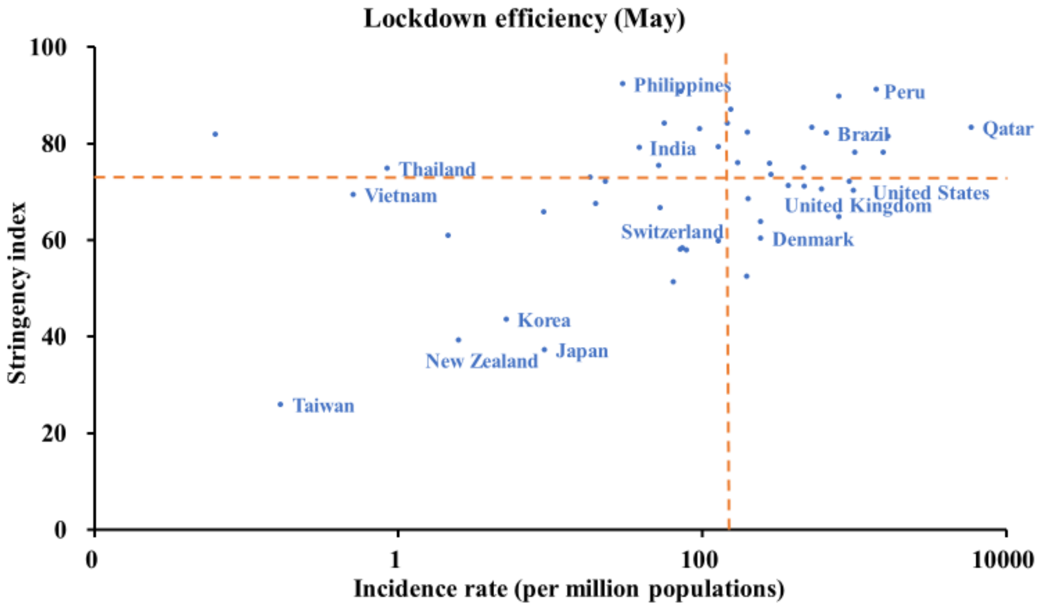

Supplementary Figure S5. **Lockdown efficiency in June 2020**

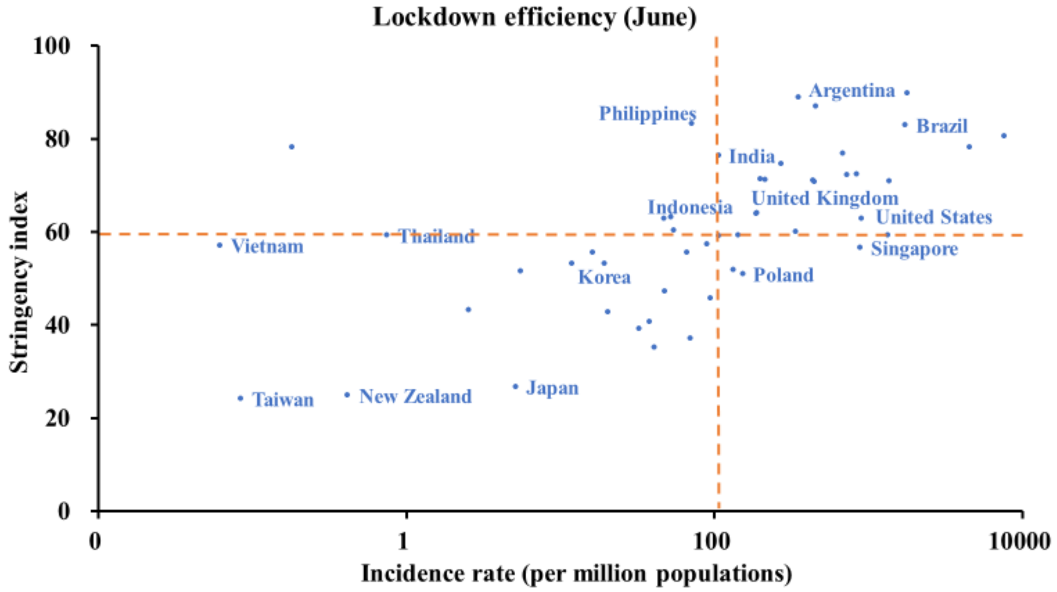

Supplementary Figure S6. **Lockdown efficiency in July 2020**

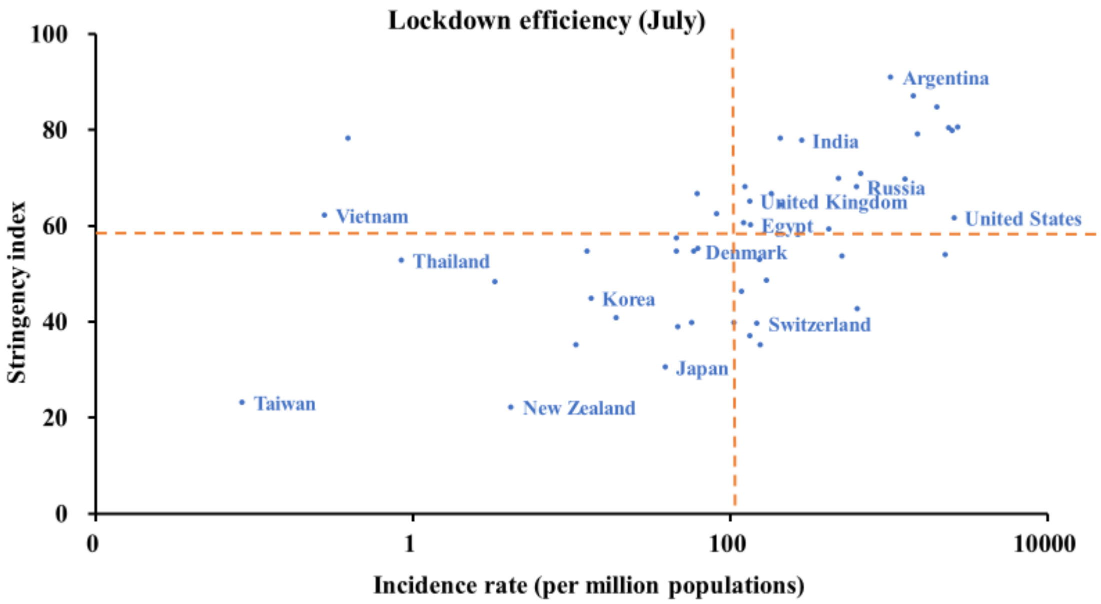

Supplementary Figure S7. **Lockdown efficiency in August 2020**

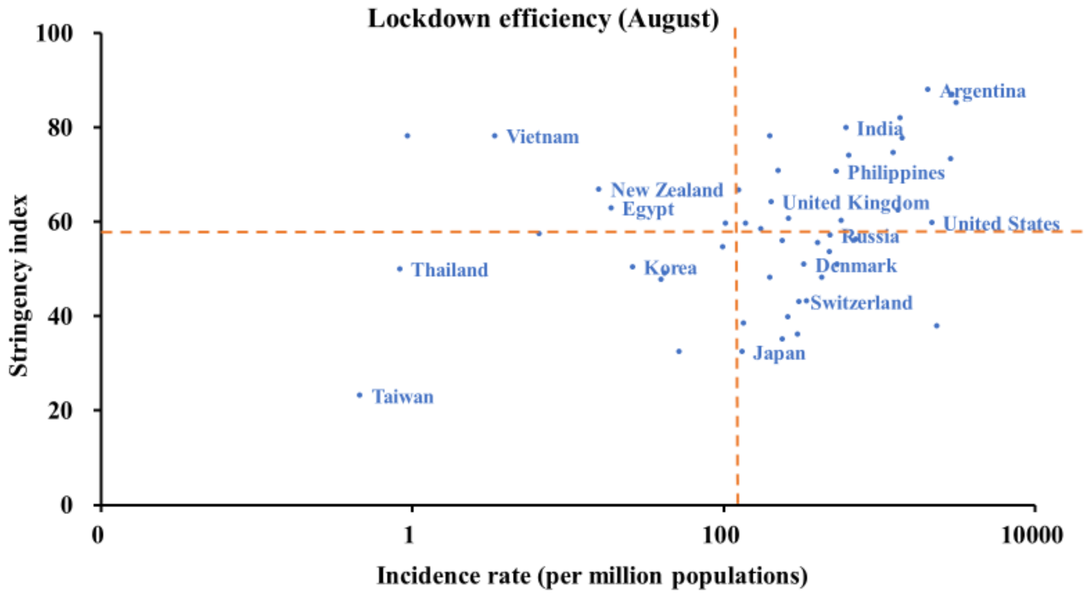

Supplementary Figure S8. **Lockdown efficiency in September 2020**

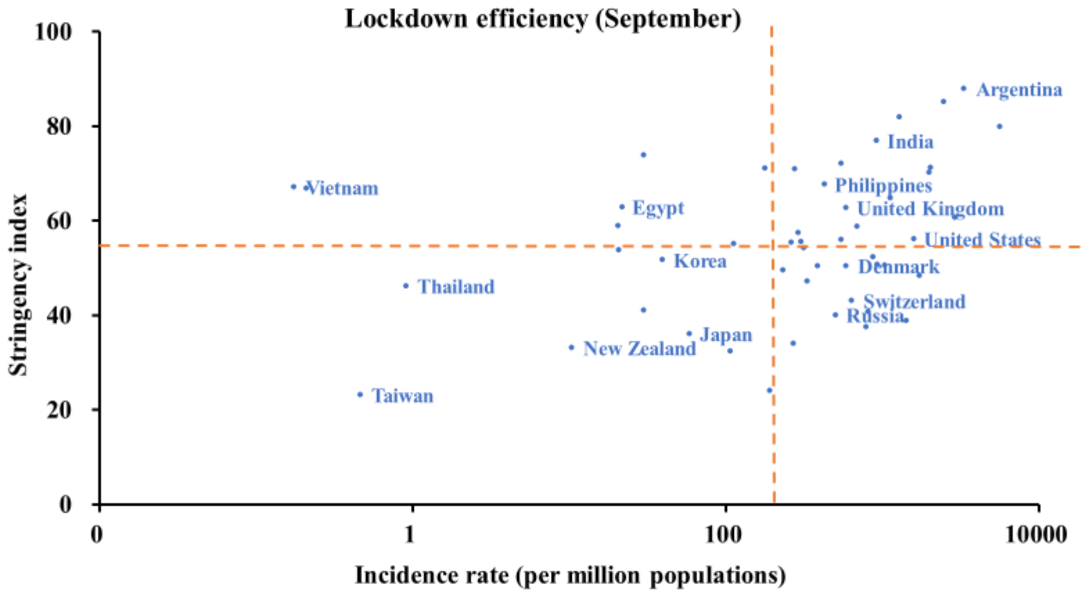

Supplementary Figure S9. **Lockdown efficiency in October 2020**

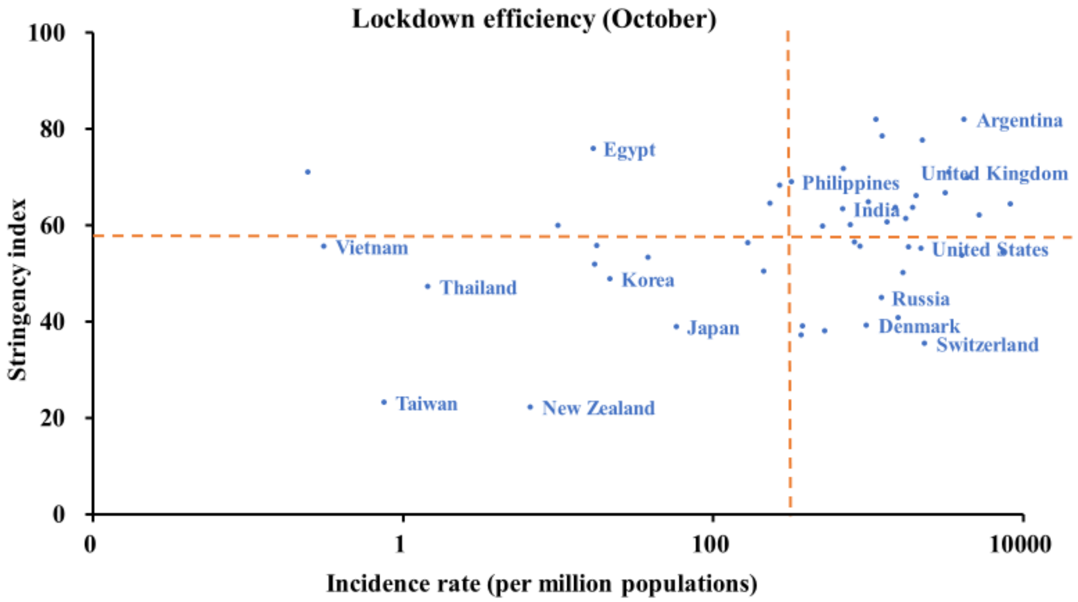

Supplementary Figure S10. **Lockdown efficiency in November 2020**

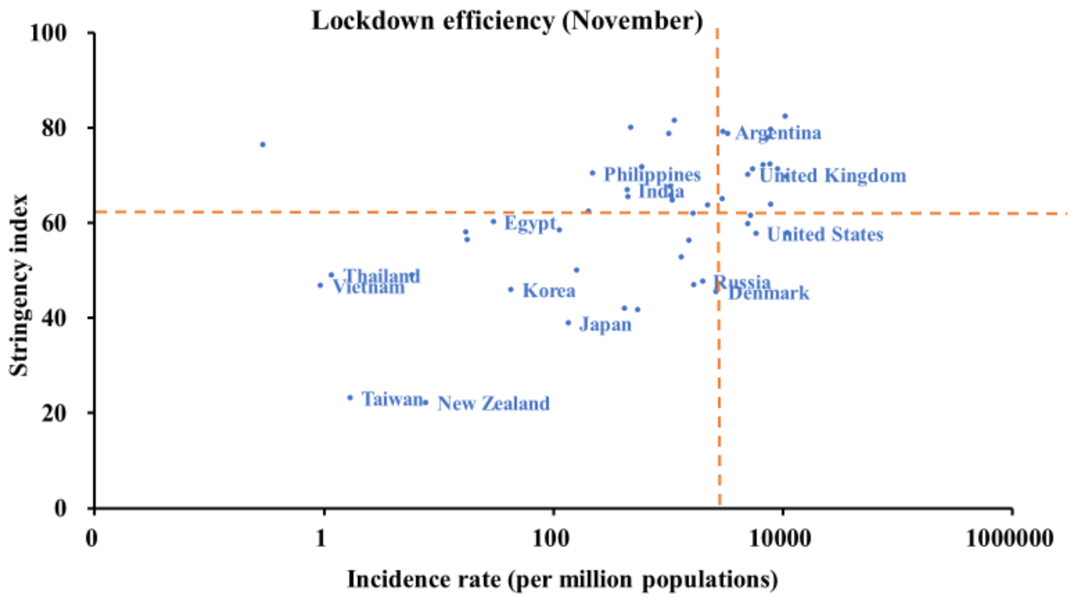

"Lockdown efficiency" is presented as the average stringency index of the last two weeks of the month compared to the two-week total incidence of COVID-19 of the first two weeks of the month. When calculating the incidence rate of COVID-19, we consider both imported and local cases. Since the purpose of enforcing lockdown policy is to suppress the possibility of domestic person-to-person transmission, it is, however, more accurate to consider only COVID-19 local cases when determining

the lockdown efficiency score of each country and region. Similarly, a more accurate lockdown efficiency score can be obtained if the stringency index of each nation and place is calculated by only considering the indicators related to domestic blockade, meaning that the stringency index should be modified by excluding indicator of international travel controls (C8).

However, since the daily number of local cases in each country and region cannot be fully obtained in the public domain, it is not feasible to only consider local infections when calculating the incidence rate. This model limitation might slighly influence the lockdown efficiency score for the 50 nations and places. For example, in Taiwan, as of November 30, 2020, among the 675 COVID-19 cases, 583 cases are imported; 55 cases are local; 36 cases are from the Navy's "Goodwill Fleet"; one case are from undeclared source.[5] Therefore, since approximately 86.37% of the cases are imported, and 8.15% of the cases are local, Taiwan should have lower incidence rate if the determination of lockdown efficiency only considers local infections.

Supplementary Information 6. Google Trends

In our analysis, we utilize Google search trends for "wash hands" and "face mask" as surrogate indicators for health literacy and search queries of "insomnia" as COVID-19's impact on the general population's mental health.[6,7] We confirm the translation of these three terms into the local languages of all countries by using translations from both Chinese and English, with back-translation on Google Translate.

(1) Health literacy indicators: "wash hands" and "face mask"

Google Trends does not provide information on the absolute numbers of searches for "wash hands" and "face mask". Instead, it provides relative search values (on a scale from 0 to 100) to display search activities for a given term, according to a specific time period and area. Each popularity data point is defined by the total searches within a certain geographical location and time range. A value of 100 is the peak popularity of the term, while a value of 50 means that the term is half as popular in a given time period/area.

(2) Mental health indicator: insomnia

The use of Google Trends data to investigate the growth of insomnia search queries is based on the study of Lin et al.[7] Insomnia can be used as a surrogate indicator of the impact of the pandemic on global mental health. A significant increase/decrease is defined as when the actual weekly search query (from March 1, 2020, to November 30, 2021) is beyond the 90% CIs of the forecast from the baseline (from January 1, 2019, to January 19, 2020) via ARIMA (autoregressive integrated moving average) modeling. The detailed

description is as follows.

We obtain Google Trends data on "Insomnia" from January 1, 2019, to November 30, 2021, in 45 countries. We examine weekly searches from March 1, 2020, to November 30, 2021, using this period because most countries enforced lockdowns in mid-March of 2020, to compare observed search volumes with expected search volumes from the one-year baseline period between January 1, 2019, and January 19, 2020. This baseline period is based on the fact that on January 20, 2020, the human-to-human transmission aspect of COVID-19 was confirmed by the Chinese authorities. We forecast a counterfactual scenario of the expected search rates had the COVID-19 rapid outbreak and lockdown policies not occurred after January 19, 2020. The expected relative search volumes are estimated using Hyndman and Khandakar's algorithm for autoregressive integrated moving average (ARIMA) modeling.[8] The historical search rate from January 1, 2019, to January 19, 2020, is compared with the observed search rate from March 1, 2020, to November 30, 2021. For the residuals of each of the chosen models, we verify that they show no significant autocorrelation by a Ljung–Box test. Subsequently, we use weekly trends from January 1, 2019, to January 19, 2020, to forecast the future values with bootstrap CIs, which are computed using R software, version 4.0.3 (R Foundation for Statistical Computing).

We examine the number of weeks with an increase of search volume for insomnia. We define increases in search queries based on both the intensity and the duration of increases in the population of interest, according to previous studies.[6,7] The intensity of a significant increase is defined when the actual daily Google Trends value during March 1, 2020, to November 30, 2021, is higher than the expected by +1.645 standard error (via ARIMA), which is the upper limit of the 95% CI in a one-tailed test. We calculate the number of weeks with a significant increase in searches for insomnia higher than the 95% CI in each country from March 1, 2020, to November 30, 2021.

Supplementary Information 7. Conversion of the ten indicators on "government policies & hygiene education" and "vital health & socioeconomic" measures

We describe the calculation methods of ten indicators in this section.

(A) For health literacy, people covered by vaccines, vital health indicators (one-month cases per 100,000 members of the population, infection growth rate, one-month case fatality rate), GDP loss, unemployment rate, and insomnia, our calculation method is as follows:

Use a standardized scoring method[9] for the indicators to grade the performance of each country relative to others in the range of 0-100. Two formulas were used to calculate a standardized score of the indicator. For indicators where a larger value indicates better performance, for example, health literacy, people covered by vaccines, and GDP loss, the following formula was used:

$$\text{stdscore}_x = 100 \times \frac{indicator_{max} - indicator_x}{indicator_{max} - indicator_{min}}$$

where indicator_x represents the original data for the country, indicator_{max} and indicator_{min} represent the upper and lower bounds for the data set, and stdscore_x represents the computed indicator score for the country.

However, sometimes a lower value for a measure indicates better performance in the underlying data, such as one-month cases per 100,000 members of the population, infection growth rate, one-month case fatality rate, unemployment rate and insomnia. In this case, subtract 100 from the previously calculated formula to obtain the following standardized score:

$$stdscore_x^{low} = 100 - stdsocre_x$$

(B) For the index: health-system policies, our calculation method is as follows:

Health-system policies is a measurement index based on five response indicators of OxCGRT[2], including

H1: Public Information Campaigns (0-2),

H2: Testing Policy (0-3),

H3: Contact tracing (0-2),

H6: Facial coverings (0-4), and

H7: Vaccination policy (0-5).

Each indicator is recorded in the order of 0 to 5 according to the strictness of the representative policy. We calculated Health-system policies as a simple average of the above-mentioned component indicators (vaccination policy is added in December 2020 and later), and rescaled to a value from 0 to 100 (100 = most stringent). The formula is as follows, where k is the number of component indicators in an index and H_j is the sub-index score for an individual indicator.

$$\text{Health-system policies} = \frac{1}{k}\sum_{j=1}^{k} H_j$$

where

Index name	k	H1	H2	H3	H6	H7*
Health-system policies	4 (5)	x	x	x	x	x

* vaccination policy is added in December 2020 and later

(C) <u>For the index: lockdown efficiency, our calculation method is as follows:</u>

Lockdown efficiency, as described in Supplementary Information 5, is a new indicator that combines the stringency index of OxCGRT[2] and the incidence of COVID-19. Stringency index is a comprehensive measurement index based on nine response indicators, including

C1: school closures (0-3),

C2: workplace closing (0-3),

C3: cancel public events (0-2),

C4: restrictions on gatherings (0-4),

C5: public transportation (0-2),

C6: stay at home order (0-2),

C7: restrictions on internal movement (0-2),

C8: international travel controls (0-4), and

H1: public information campaigns (0-2).

Each indicator is recorded in the order of 0 to 4 according to the strictness of the representative policy. The most stringent government policy is represented by the highest ordinal value, and rescaled to a value from 0 to 100. According to the Oxford Covid-19 Government Response Tracker[10], the stringency index is a simple average of the individual component indicators, ranging from 0-100 (100 = strictest). This is described in equation 1 below where k is the number of component indicators in an index and I_j is the sub-index score for an individual indicator.

$$(1) \quad \text{index} = \frac{1}{k}\sum_{j=1}^{k} I_j$$

where

Index	k	C1	C2	C3	C4	C5	C6	C7	C8	H1

name										
Stringency index	9	x	x	x	x	x	x	x	x	x

According to the method of OxCGRT, lockdown efficiency is recorded in the order of 1 to 4, where 4 is evaluated as the best performance, and the score is rescaled to 0 to 100.

A country's performance score is a simple average of the standardized scores of ten indicators. Then we rank the performance of 50 countries and territories according to the total scores of the indicators. The results were displayed and explained in main text.

We also considered some alternative ways to calculate these indicators. For example, we first divided each indicator into a 1-4 scale based on quartiles; then aggregated all scores with an arithmetic average; and finally rescaled the arithmetic average into a score between 0 and 100. We compared the results with different calculation methods for sensitivity analysis.

Supplementary Information 8. Overall COVID-19 containment ranking of 50 countries and territories from March 2020 to November 2021

Supplementary Table S6. **Overall COVID-19 containment ranking (with Google Trends)**

Mar 2020	R[a]	Apr 2020	R[a]	May 2020	R[a]	Jun 2020	R[a]	Jul 2020	R[a]	Aug 2020	R[a]	Sep 2020	R[a]	Oct 2020	R[a]	Nov 2020	R[a]
Taiwan	1	Japan	1	South Korea	1	Taiwan	1	Malaysia	1	Taiwan	1	Taiwan	1	Taiwan	1	Taiwan	1
Thailand	2	Taiwan	2	Taiwan	2	South Korea	2	Netherlands	2	Turkey	2	Italy	2	Singapore	2	Australia	2
Japan	3	South Korea	3	Vietnam	3	Turkey	3	Taiwan	3	Italy	3	Singapore	3	India	3	India	3
Qatar	4	Indonesia	4	Germany	4	Vietnam	4	South Korea	4	New Zealand	4	New Zealand	4	New Zealand	4	New Zealand	4
Russia	5	Australia	5	Czech Republic	5	Japan	5	France	5	Finland	5	South Africa	5	Japan	5	South Korea	5
Egypt	6	Hong Kong	6	Singapore	6	Australia	6	New Zealand	6	South Korea	6	Finland	6	South Korea	6	Japan	6
Vietnam	7	Poland	7	Austria	7	Malaysia	7	Thailand	7	Malaysia	7	South Korea	7	Vietnam	7	Vietnam	7
Chile	8	Hungary	8	Russia	8	Denmark	8	Germany	8	Germany	8	Germany	8	Canada	8	Egypt	8
New Zealand	9	Finland	9	Saudi Arabia	9	Russia	9	Finland	9	Thailand	9	Turkey	9	Finland	9	Singapore	9
Italy	10	Portugal	10	Hong Kong	10	New Zealand	10	Italy	10	Canada	10	Japan	10	Thailand	10	Turkey	10
France	11	Malaysia	11	Australia	11	Czech Republic	11	Denmark	11	Poland	11	Poland	11	Italy	11	Saudi Arabia	11
Mexico	12	Argentina	12	New Zealand	12	Saudi Arabia	12	Poland	12	Denmark	12	Canada	12	Hong Kong	12	Thailand	12
Finland	13	New Zealand	13	Portugal	13	Canada	13	Turkey	13	United Kingdom	13	United Kingdom	13	Saudi Arabia	13	Hong Kong	13

Australia	14	Saudi Arabia	14	France	14	Finland	14	United Kingdom	14	France	14	Russia	14	Denmark	14	South Africa	14
Brazil	15	Italy	15	Poland	15	Hong Kong	15	Japan	15	South Africa	15	Brazil	15	Turkey	15	Chile	15
Canada	16	South Africa	16	Egypt	16	Portugal	16	Czech Republic	16	Hungary	16	Malaysia	16	Netherlands	16	Germany	16
India	17	Czech Republic	17	Japan	17	Austria	17	Canada	17	Saudi Arabia	17	Thailand	17	South Africa	17	Brazil	17
Hong Kong	18	Vietnam	18	Malaysia	18	France	18	Russia	18	Singapore	18	Mexico	18	Portugal	18	Finland	18
Netherlands	19	Germany	19	Indonesia	19	Indonesia	19	Spain	19	Austria	19	India	19	Chile	19	Argentina	19
South Korea	20	Austria	20	Finland	20	Italy	20	Hungary	20	Japan	20	Netherlands	20	Germany	20	Russia	20
Malaysia	21	France	21	South Africa	21	Germany	21	Vietnam	21	Sweden	21	Portugal	21	Australia	21	Sweden	21
United Kingdom	22	Netherlands	22	Turkey	22	South Africa	22	Portugal	22	Portugal	22	Saudi Arabia	22	Brazil	22	Peru	22
Denmark	23	Egypt	23	Italy	23	Egypt	23	Australia	23	Russia	23	France	23	Russia	23	Malaysia	23
Sweden	24	Russia	24	Denmark	24	United States	24	Egypt	24	Australia	24	Vietnam	24	Sweden	24	Canada	24
Germany	25	Turkey	25	Sweden	25	Netherlands	25	Sweden	25	Egypt	25	Sweden	25	Malaysia	25	France	25
United States	26	Chile	26	Canada	26	United Kingdom	26	Saudi Arabia	26	Netherlands	26	Denmark	26	United Kingdom	26	Colombia	26
Singapore	27	Denmark	27	Hungary	27	Poland	27	Austria	27	Hong Kong	27	Austria	27	Austria	27	United Kingdom	27
Austria	28	Colombia	28	Colombia	28	India	28	India	28	Brazil	28	Spain	28	Argentina	28	Denmark	28
Spain	29	Qatar	29	Argentina	29	Singapore	29	Hong Kong	29	Spain	29	Hong Kong	29	Peru	29	Mexico	29
Argentina	NA[b]	Singapore	30	Netherlands	30	Spain	30	Singapore	30	India	30	United States	30	Egypt	30	Spain	30
Belgium	NA[b]	Sweden	31	Qatar	31	Colombia	31	Brazil	31	Chile	31	Chile	31	France	31	Netherlands	31

China	NA[b]	Canada	32	Brazil	32	Qatar	32	Argentina	32	Czech Republic	32	Egypt	32	Colombia	32	Italy	32
Colombia	NA[b]	Peru	33	United Kingdom	33	Chile	33	South Africa	33	United States	33	Argentina	33	Spain	33	United States	33
Czech Republic	NA[b]	Mexico	34	United States	34	Sweden	34	Chile	34	Mexico	34	Peru	34	Mexico	34	Portugal	34
Greece	NA[b]	India	35	Spain	35	Brazil	35	United States	35	Argentina	35	Australia	35	United States	35	Austria	35
Hungary	NA[b]	Brazil	36	Peru	36	Argentina	36	Mexico	36	Vietnam	36	Colombia	36	Poland	36	Czech Republic	36
Indonesia	NA[b]	United Kingdom	37	India	37	Peru	37	Colombia	37	Colombia	37	Czech Republic	37	Hungary	37	Hungary	37
Ireland	NA[b]	Spain	38	Chile	38	Hungary	38	Peru	38	Peru	38	Hungary	38	Czech Republic	38	Poland	38
Israel	NA[b]	United States	39	Mexico	39	Mexico	39	Belgium	NA[b]	Belgium	NA[b]	Belgium	NA[b]	Belgium	NA[b]	Belgium	NA[b]
Norway	NA[b]	Belgium	NA[b]	Belgium	NA[b]	Belgium	NA[b]	China	NA[b]	China	NA[b]	China	NA[b]	China	NA[b]	China	NA[b]
Pakistan	NA[b]	China	NA[b]	China	NA[b]	China	NA[b]	Greece	NA[b]	Greece	NA[b]	Greece	NA[b]	Greece	NA[b]	Greece	NA[b]
Peru	NA[b]	Greece	NA[b]	Greece	NA[b]	Greece	NA[b]	Indonesia	NA[b]	Indonesia	NA[b]	Indonesia	NA[b]	Indonesia	NA[b]	Indonesia	NA[b]
Philippines	NA[b]	Ireland	NA[b]	Ireland	NA[b]	Ireland	NA[b]	Ireland	NA[b]	Ireland	NA[b]	Ireland	NA[b]	Ireland	NA[b]	Ireland	NA[b]
Poland	NA[b]	Israel	NA[b]	Israel	NA[b]	Israel	NA[b]	Israel	NA[b]	Israel	NA[b]	Israel	NA[b]	Israel	NA[b]	Israel	NA[b]
Portugal	NA[b]	Norway	NA[b]	Norway	NA[b]	Norway	NA[b]	Norway	NA[b]	Norway	NA[b]	Norway	NA[b]	Norway	NA[b]	Norway	NA[b]
Saudi Arabia	NA[b]	Pakistan	NA[b]	Pakistan	NA[b]	Pakistan	NA[b]	Pakistan	NA[b]	Pakistan	NA[b]	Pakistan	NA[b]	Pakistan	NA[b]	Pakistan	NA[b]
South Africa	NA[b]	Philippines	NA[b]	Philippines	NA[b]	Philippines	NA[b]	Philippines	NA[b]	Philippines	NA[b]	Philippines	NA[b]	Philippines	NA[b]	Philippines	NA[b]
Switzerland	NA[b]	Switzerland	NA[b]	Switzerland	NA[b]	Switzerland	NA[b]	Qatar	NA[b]	Qatar	NA[b]	Qatar	NA[b]	Qatar	NA[b]	Qatar	NA[b]

Turkey	NA[b]	Thailand	NA[b]	Thailand	NA[b]	Thailand	NA[b]	Switzerland	NA[b]	Switzerland	NA[b]	Switzerland	NA[b]	Switzerland	NA[b]	Switzerland	NA[b]
United Arab Emirates	NA[b]	United Arab Emirates	NA[b]	United Arab Emirates	NA[b]	United Arab Emirates	NA[b]	United Arab Emirates	NA[b]	United Arab Emirates	NA[b]	United Arab Emirates	NA[b]	United Arab Emirates	NA[b]	United Arab Emirates	NA[b]

Notes:

a. R means each country's ranking.

b. NA: not applicable. Indicates a country not ranked due to incomplete data.

Dec 2020	R[a]	Jan 2021	R[a]	Feb 2021	R[a]	Mar 2021	R[a]	Apr 2021	R[a]	May 2021	R[a]	Jun 2021	R[a]
Taiwan	1	Taiwan	1	Singapore	1	Singapore	1	United Kingdom	1	Singapore	1	Singapore	1
Finland	2	Singapore	2	Taiwan	2	South Korea	2	Singapore	2	United Kingdom	2	Czech Republic	2
Chile	3	Saudi Arabia	3	India	3	Taiwan	3	Malaysia	3	Austria	3	Spain	3
Argentina	4	Russia	4	Saudi Arabia	4	Saudi Arabia	4	Russia	4	Spain	4	France	4
Germany	5	Egypt	5	Australia	5	Australia	5	Italy	5	Italy	5	Austria	5
Sweden	6	Italy	6	Italy	6	Egypt	6	Germany	6	United States	6	United Kingdom	6
Poland	7	Indonesia	7	Denmark	7	New Zealand	7	Spain	7	Russia	7	Italy	7
Russia	8	Austria	8	New Zealand	8	Russia	8	Portugal	8	Portugal	8	Portugal	8
Canada	9	Argentina	9	Canada	9	Canada	9	Austria	9	Malaysia	9	United States	9
United Kingdom	10	India	10	Argentina	10	Hong Kong	10	South Korea	10	South Korea	10	South Korea	10
Austria	11	Sweden	11	South Korea	11	Vietnam	11	Japan	11	Germany	11	Sweden	11
Portugal	12	Germany	12	Japan	12	Thailand	12	Taiwan	12	Mexico	12	Mexico	12
France	13	Turkey	13	Netherlands	13	Argentina	13	United States	13	France	13	Germany	13
Mexico	14	France	14	Poland	14	India	14	France	14	Hong Kong	14	Japan	14
Italy	15	Netherlands	15	Sweden	15	Austria	15	Vietnam	15	Japan	15	Russia	15
Denmark	16	Finland	16	Turkey	16	Malaysia	16	Hong Kong	16	Sweden	16	Poland	16
Hungary	17	Denmark	17	Hong Kong	17	Japan	17	Mexico	17	Poland	17	Malaysia	17
Czech Republic	18	Poland	18	United Kingdom	18	United Kingdom	18	Poland	18	Taiwan	18	Hong Kong	18
United States	19	Hungary	19	Finland	19	Germany	19	Czech Republic	19	Czech Republic	19	Taiwan	19
Australia	NA[b]	Chile	20	Austria	20	Portugal	20	Sweden	20	Vietnam	20	Vietnam	20
Belgium	NA[b]	Spain	21	Germany	21	Italy	21	Argentina	NA[b]	Argentina	NA[b]	Argentina	NA[b]

Brazil	NA[b]	Brazil	22	Thailand	22	Indonesia	22	Australia	NA[b]	Australia	NA[b]	Australia	NA[b]
China	NA[b]	Portugal	23	Mexico	23	Turkey	23	Belgium	NA[b]	Belgium	NA[b]	Belgium	NA[b]
Colombia	NA[b]	United Kingdom	24	Chile	24	France	24	Brazil	NA[b]	Brazil	NA[b]	Brazil	NA[b]
Egypt	NA[b]	Mexico	25	Malaysia	25	South Africa	25	Canada	NA[b]	Canada	NA[b]	Canada	NA[b]
Greece	NA[b]	Canada	26	Portugal	26	Netherlands	26	Chile	NA[b]	Chile	NA[b]	Chile	NA[b]
Hong Kong	NA[b]	Czech Republic	27	France	27	Chile	27	China	NA[b]	China	NA[b]	China	NA[b]
India	NA[b]	United States	28	Indonesia	28	Sweden	28	Colombia	NA[b]	Colombia	NA[b]	Colombia	NA[b]
Indonesia	NA[b]	Australia	NA[b]	South Africa	29	Denmark	29	Denmark	NA[b]	Denmark	NA[b]	Denmark	NA[b]
Ireland	NA[b]	Belgium	NA[b]	Spain	30	Colombia	30	Egypt	NA[b]	Egypt	NA[b]	Egypt	NA[b]
Israel	NA[b]	China	NA[b]	Hungary	31	Spain	31	Finland	NA[b]	Finland	NA[b]	Finland	NA[b]
Japan	NA[b]	Colombia	NA[b]	Colombia	32	United States	32	Greece	NA[b]	Greece	NA[b]	Greece	NA[b]
Malaysia	NA[b]	Greece	NA[b]	Brazil	33	Mexico	33	Hungary	NA[b]	Hungary	NA[b]	Hungary	NA[b]
Netherlands	NA[b]	Hong Kong	NA[b]	United States	34	Peru	34	India	NA[b]	India	NA[b]	India	NA[b]
New Zealand	NA[b]	Ireland	NA[b]	Peru	35	Brazil	35	Indonesia	NA[b]	Indonesia	NA[b]	Indonesia	NA[b]
Norway	NA[b]	Israel	NA[b]	Czech Republic	36	Poland	36	Ireland	NA[b]	Ireland	NA[b]	Ireland	NA[b]
Pakistan	NA[b]	Japan	NA[b]	Belgium	NA[b]	Finland	37	Israel	NA[b]	Israel	NA[b]	Israel	NA[b]
Peru	NA[b]	Malaysia	NA[b]	China	NA[b]	Czech Republic	38	Netherlands	NA[b]	Netherlands	NA[b]	Netherlands	NA[b]
Philippines	NA[b]	New Zealand	NA[b]	Egypt	NA[b]	Hungary	39	New Zealand	NA[b]	New Zealand	NA[b]	New Zealand	NA[b]
Qatar	NA[b]	Norway	NA[b]	Greece	NA[b]	Belgium	NA[b]	Norway	NA[b]	Norway	NA[b]	Norway	NA[b]
Saudi Arabia	NA[b]	Pakistan	NA[b]	Ireland	NA[b]	China	NA[b]	Pakistan	NA[b]	Pakistan	NA[b]	Pakistan	NA[b]
Singapore	NA[b]	Peru	NA[b]	Israel	NA[b]	Greece	NA[b]	Peru	NA[b]	Peru	NA[b]	Peru	NA[b]
South Africa	NA[b]	Philippines	NA[b]	Norway	NA[b]	Ireland	NA[b]	Philippines	NA[b]	Philippines	NA[b]	Philippines	NA[b]
South Korea	NA[b]	Qatar	NA[b]	Pakistan	NA[b]	Israel	NA[b]	Qatar	NA[b]	Qatar	NA[b]	Qatar	NA[b]

Spain	NA[b]	South Africa	NA[b]	Philippines	NA[b]	Norway	NA[b]	Saudi Arabia	NA[b]	Saudi Arabia	NA[b]	Saudi Arabia	NA[b]
Switzerland	NA[b]	South Korea	NA[b]	Qatar	NA[b]	Pakistan	NA[b]	South Africa	NA[b]	South Africa	NA[b]	South Africa	NA[b]
Thailand	NA[b]	Switzerland	NA[b]	Russia	NA[b]	Philippines	NA[b]	Switzerland	NA[b]	Switzerland	NA[b]	Switzerland	NA[b]
Turkey	NA[b]	Thailand	NA[b]	Switzerland	NA[b]	Qatar	NA[b]	Thailand	NA[b]	Thailand	NA[b]	Thailand	NA[b]
United Arab Emirates	NA[b]	United Arab Emirates	NA[b]	United Arab Emirates	NA[b]	Switzerland	NA[b]	Turkey	NA[b]	Turkey	NA[b]	Turkey	NA[b]
Vietnam	NA[b]	Vietnam	NA[b]	Vietnam	NA[b]	United Arab Emirates	NA[b]	United Arab Emirates	NA[b]	United Arab Emirates	NA[b]	United Arab Emirates	NA[b]

Notes:

a. R means each country’s ranking.

b. NA: not applicable. Indicates a country not ranked due to incomplete data.

Jul 2021	R[a]	Aug 2021	R[a]	Sep 2021	R[a]	Oct 2021	R[a]	Nov 2021	R[a]
Singapore	1	Singapore	1	Colombia	1	Chile	1	Italy	1
Canada	2	Canada	2	Chile	2	Peru	2	Taiwan	2
Chile	3	Peru	3	Japan	3	Canada	3	Malaysia	3
Peru	4	Saudi Arabia	4	Peru	4	Italy	4	Chile	4
Japan	5	Chile	5	South Korea	5	Malaysia	5	South Korea	5
Hungary	6	Colombia	6	Poland	6	Brazil	6	Peru	6
Italy	7	Czech Republic	7	Czech Republic	7	Taiwan	7	Japan	7
Austria	8	Argentina	8	Russia	8	Sweden	8	Mexico	8
Saudi Arabia	9	Italy	9	Spain	9	Hong Kong	9	Sweden	9
Sweden	10	Poland	10	Portugal	10	Colombia	10	Canada	10
South Korea	11	Hungary	11	Italy	11	South Korea	11	Colombia	11
New Zealand	12	South Korea	12	Denmark	12	Poland	12	Australia	12
Australia	13	Australia	13	Taiwan	13	Mexico	13	Hong Kong	13
Colombia	14	Sweden	14	Canada	14	Austria	14	Russia	14
Poland	15	Portugal	15	France	15	Japan	15	Germany	15
Czech Republic	16	Netherlands	16	New Zealand	16	Russia	16	Netherlands	16
Netherlands	17	Austria	17	Hungary	17	Turkey	17	Finland	17
Germany	18	New Zealand	18	Austria	18	Australia	18	United States	18
United States	19	Japan	19	India	19	Denmark	19	Poland	19
Hong Kong	20	France	20	Singapore	20	Finland	20	Austria	20
India	21	Hong Kong	21	Hong Kong	21	Netherlands	21	Hungary	21

Argentina	22	Germany	22	Germany	22	Hungary	22	Czech Republic	22
Portugal	23	Spain	23	Argentina	23	Germany	23	Argentina	NA[b]
Mexico	24	Russia	24	Brazil	24	Czech Republic	24	Belgium	NA[b]
France	25	Denmark	25	Saudi Arabia	25	United Kingdom	25	Brazil	NA[b]
Spain	26	Mexico	26	Netherlands	26	United States	26	China	NA[b]
Russia	27	United Kingdom	27	Turkey	27	Argentina	NA[b]	Denmark	NA[b]
Denmark	28	Brazil	28	United Kingdom	28	Belgium	NA[b]	Egypt	NA[b]
Finland	29	India	29	Sweden	29	China	NA[b]	France	NA[b]
United Kingdom	30	Finland	30	Malaysia	30	Egypt	NA[b]	Greece	NA[b]
Thailand	31	Turkey	31	Finland	31	France	NA[b]	India	NA[b]
Malaysia	32	United States	32	Australia	32	Greece	NA[b]	Indonesia	NA[b]
Turkey	33	South Africa	33	Mexico	33	India	NA[b]	Ireland	NA[b]
Brazil	34	Taiwan	34	United States	34	Indonesia	NA[b]	Israel	NA[b]
Taiwan	35	Thailand	35	Thailand	35	Ireland	NA[b]	New Zealand	NA[b]
South Africa	36	Malaysia	36	South Africa	36	Israel	NA[b]	Norway	NA[b]
Vietnam	37	Vietnam	37	Vietnam	37	New Zealand	NA[b]	Pakistan	NA[b]
Belgium	NA[b]	Belgium	NA[b]	Belgium	NA[b]	Norway	NA[b]	Philippines	NA[b]
China	NA[b]	China	NA[b]	China	NA[b]	Pakistan	NA[b]	Portugal	NA[b]
Egypt	NA[b]	Egypt	NA[b]	Egypt	NA[b]	Philippines	NA[b]	Qatar	NA[b]
Greece	NA[b]	Greece	NA[b]	Greece	NA[b]	Portugal	NA[b]	Saudi Arabia	NA[b]
Indonesia	NA[b]	Indonesia	NA[b]	Indonesia	NA[b]	Qatar	NA[b]	Singapore	NA[b]
Ireland	NA[b]	Ireland	NA[b]	Ireland	NA[b]	Saudi Arabia	NA[b]	South Africa	NA[b]
Israel	NA[b]	Israel	NA[b]	Israel	NA[b]	Singapore	NA[b]	Spain	NA[b]

Norway	NA[b]	Norway	NA[b]	Norway	NA[b]	South Africa	NA[b]	Switzerland	NA[b]
Pakistan	NA[b]	Pakistan	NA[b]	Pakistan	NA[b]	Spain	NA[b]	Thailand	NA[b]
Philippines	NA[b]	Philippines	NA[b]	Philippines	NA[b]	Switzerland	NA[b]	Turkey	NA[b]
Qatar	NA[b]	Qatar	NA[b]	Qatar	NA[b]	Thailand	NA[b]	United Arab Emirates	NA[b]
Switzerland	NA[b]	Switzerland	NA[b]	Switzerland	NA[b]	United Arab Emirates	NA[b]	United Kingdom	NA[b]
United Arab Emirates	NA[b]	United Arab Emirates	NA[b]	United Arab Emirates	NA[b]	Vietnam	NA[b]	Vietnam	NA[b]

Notes:

a. R means each country's ranking.

b. NA: not applicable. Indicates a country not ranked due to incomplete data.

Supplementary Table S7. **Overall COVID-19 containment ranking (without Google Trends)**

Mar 2020	R[a]	Apr 2020	R[a]	May 2020	R[a]	Jun 2020	R[a]	Jul 2020	R[a]	Aug 2020	R[a]	Sep 2020	R[a]	Oct 2020	R[a]	Nov 2020	R[a]
Vietnam	1	Taiwan	1	Vietnam	1	Taiwan	1	Taiwan	1	Turkey	1	Taiwan	1	Taiwan	1	Taiwan	1
Taiwan	2	South Korea	2	South Korea	2	South Korea	2	South Korea	2	Taiwan	2	Singapore	2	Singapore	2	Singapore	2
Qatar	3	Hong Kong	3	Hong Kong	3	Vietnam	3	New Zealand	3	Italy	3	Italy	3	Hong Kong	3	China	3
Japan	4	Australia	4	Taiwan	4	Norway	4	Germany	4	South Korea	4	South Korea	4	Vietnam	4	Australia	4
Thailand	5	Indonesia	5	Australia	5	Australia	5	Thailand	5	Thailand	5	Turkey	5	New Zealand	5	Vietnam	5
Hong Kong	6	Vietnam	6	China	6	China	6	Netherlands	6	Norway	6	Germany	6	Japan	6	New Zealand	6
Malaysia	7	Japan	7	Norway	7	Switzerland	7	Norway	7	Germany	7	New Zealand	7	Thailand	7	South Korea	7
Mexico	8	Malaysia	8	Czech Republic	8	New Zealand	8	Malaysia	8	China	8	Norway	8	South Korea	8	Hong Kong	8
Egypt	9	Israel	9	Germany	9	Turkey	9	China	9	Finland	9	Thailand	9	China	9	Japan	9
Greece	10	South Africa	10	Turkey	10	Czech Republic	10	Ireland	10	Ireland	10	China	10	Norway	10	Thailand	10
Chile	11	China	11	Indonesia	11	Hong Kong	11	Denmark	11	Austria	11	Finland	11	Finland	11	Egypt	11
Russia	12	Hungary	12	Singapore	12	Finland	12	France	12	New Zealand	12	Poland	12	India	12	India	12
New Zealand	13	New Zealand	13	Austria	13	Japan	13	Finland	13	Poland	13	Japan	13	Turkey	13	Finland	13
Israel	14	Italy	14	Switzerland	14	Denmark	14	Greece	14	Malaysia	14	South Africa	14	Saudi Arabia	14	Saudi Arabia	14
South Korea	15	Poland	15	Russia	15	Greece	15	Italy	15	Hungary	15	Greece	15	Canada	15	Israel	15
Singapore	16	Egypt	16	New Zealand	16	Malaysia	16	Czech Republic	16	Portugal	16	Vietnam	16	Brazil	16	South Africa	16

Norway	17	Czech Republic	17	Israel	17	Russia	17	Turkey	17	Saudi Arabia	17	Malaysia	17	Chile	17	Ireland	17
Australia	18	Norway	18	Greece	18	Israel	18	Poland	18	Switzerland	18	United Kingdom	18	Ireland	18	Turkey	18
Philippines	19	Greece	19	Saudi Arabia	19	Austria	19	Japan	19	Singapore	19	Portugal	19	South Africa	19	Chile	19
Denmark	20	Argentina	20	Ireland	20	France	20	Hungary	20	United Kingdom	20	Ireland	20	Israel	20	Argentina	20
India	21	Portugal	21	Malaysia	21	Portugal	21	Switzerland	21	Japan	21	Brazil	21	Australia	21	Brazil	21
France	22	Saudi Arabia	22	South Africa	22	Germany	22	Vietnam	22	France	22	India	22	Germany	22	France	22
Brazil	23	Finland	23	Poland	23	Saudi Arabia	23	Spain	23	Hong Kong	23	Switzerland	23	Italy	23	Norway	23
Finland	24	Brazil	24	Japan	24	Singapore	24	Portugal	24	South Africa	24	United States	24	Greece	24	Malaysia	24
Netherlands	25	Germany	25	Egypt	25	Egypt	25	Russia	25	Denmark	25	Hong Kong	25	Austria	25	Germany	25
Canada	26	Colombia	26	Denmark	26	Italy	26	Saudi Arabia	26	Canada	26	Saudi Arabia	26	Portugal	26	Russia	26
Sweden	27	Chile	27	Finland	27	Netherlands	27	Singapore	27	Belgium	27	Sweden	27	Denmark	27	Denmark	27
Ireland	28	France	28	Portugal	28	Indonesia	28	Belgium	28	Netherlands	28	Canada	28	Argentina	28	Colombia	28
Italy	29	Austria	29	Spain	29	Canada	29	Egypt	29	Australia	29	Russia	29	Russia	29	Belgium	29
Germany	30	Turkey	30	Hungary	30	Spain	30	Austria	30	Russia	30	Egypt	30	Malaysia	30	United Kingdom	30
Switzerland	31	Ireland	31	Brazil	31	Qatar	31	United Kingdom	31	Sweden	31	Belgium	31	Sweden	31	Netherlands	31
United Kingdom	32	Switzerland	32	Belgium	32	Brazil	32	Sweden	32	Greece	32	Mexico	32	United Kingdom	32	Canada	32
China	33	Denmark	33	Qatar	33	United States	33	Canada	33	Czech	33	Denmark	33	Spain	33	Mexico	33

										Republic							
Austria	34	Mexico	34	Argentina	34	Poland	34	Australia	34	Egypt	34	Austria	34	Netherlands	34	Peru	34
United States	35	Netherlands	35	France	35	South Africa	35	Hong Kong	35	United States	35	France	35	United States	35	Spain	35
Spain	36	Qatar	36	Colombia	36	United Kingdom	36	India	36	India	36	Netherlands	36	Egypt	36	Greece	36
Belgium	37	Sweden	37	Mexico	37	Belgium	37	Brazil	37	Spain	37	Chile	37	Peru	37	Philippines	37
Argentina	NA[b]	Belgium	38	Sweden	38	Hungary	38	Argentina	38	Brazil	38	Spain	38	France	38	Italy	38
Colombia	NA[b]	Singapore	39	Italy	39	Ireland	39	Philippines	39	Vietnam	39	Philippines	39	Colombia	39	Portugal	39
Czech Republic	NA[b]	Russia	40	Netherlands	40	Sweden	40	Mexico	40	Mexico	40	Argentina	40	Switzerland	40	Czech Republic	40
Hungary	NA[b]	Spain	41	United States	41	Argentina	41	United States	41	Chile	41	Australia	41	Mexico	41	United States	41
Indonesia	NA[b]	Philippines	42	Canada	42	India	42	Chile	42	Israel	42	Israel	42	Philippines	42	Sweden	42
Pakistan	NA[b]	Canada	43	Chile	43	Mexico	43	South Africa	43	Argentina	43	Czech Republic	43	Hungary	43	Poland	43
Peru	NA[b]	Peru	44	Philippines	44	Chile	44	Israel	44	Philippines	44	Colombia	44	Poland	44	Austria	44
Poland	NA[b]	United Kingdom	45	United Kingdom	45	Colombia	45	Peru	45	Colombia	45	Peru	45	Czech Republic	45	Switzerland	45
Portugal	NA[b]	United States	46	Peru	46	Philippines	46	Colombia	46	Peru	46	Hungary	46	Belgium	46	Hungary	46
Saudi Arabia	NA[b]	India	47	India	47	Peru	47	Indonesia	NA[b]	Indonesia	NA[b]	Indonesia	NA[b]	Indonesia	NA[b]	Indonesia	NA[b]
South Africa	NA[b]	Pakistan	NA[b]	Pakistan	NA[b]	Pakistan	NA[b]	Pakistan	NA[b]	Pakistan	NA[b]	Pakistan	NA[b]	Pakistan	NA[b]	Pakistan	NA[b]
Turkey	NA[b]	Thailand	NA[b]	Thailand	NA[b]	Thailand	NA[b]	Qatar	NA[b]	Qatar	NA[b]	Qatar	NA[b]	Qatar	NA[b]	Qatar	NA[b]
United Arab	NA[b]	United Arab	NA[b]	United Arab	NA[b]	United Arab	NA[b]	United Arab	NA[b]	United Arab	NA[b]	United Arab	NA[b]	United Arab	NA[b]	United Arab	NA[b]

Emirates	Emirates	Emirates	Emirates	Emirates	Emirates	Emirates	Emirates	Emirates

Notes:

a. R means each country's ranking.

b. NA: not applicable. Indicates a country not ranked due to incomplete data.

Dec 2020	R[a]	Jan 2021	R[a]	Feb 2021	R[a]	Mar 2021	R[a]	Apr 2021	R[a]	May 2021	R[a]	Jun 2021	R[a]
Taiwan	1	China	1	China	1	China	1	United Kingdom	1	United Kingdom	1	Philippines	1
China	2	Singapore	2	Singapore	2	Singapore	2	Singapore	2	Singapore	2	Singapore	2
Israel	3	Taiwan	3	India	3	India	3	Malaysia	3	Spain	3	Austria	3
Norway	4	Saudi Arabia	4	Saudi Arabia	4	Saudi Arabia	4	Spain	4	Austria	4	Portugal	4
Finland	5	Argentina	5	Taiwan	5	Australia	5	Portugal	5	United States	5	China	5
Chile	6	Israel	6	Australia	6	Taiwan	6	United States	6	Philippines	6	Italy	6
Argentina	7	India	7	New Zealand	7	New Zealand	7	China	7	Portugal	7	United States	7
Ireland	8	Finland	8	Denmark	8	Vietnam	8	Austria	8	China	8	Czech Republic	8
Canada	9	Turkey	9	Argentina	9	Hong Kong	9	Italy	9	Italy	9	United Kingdom	9
Germany	10	Russia	10	Israel	10	Canada	10	Philippines	10	Malaysia	10	Mexico	10
Italy	11	Italy	11	South Korea	11	Israel	11	Russia	11	Germany	11	Spain	11
Portugal	12	Indonesia	12	Turkey	12	South Korea	12	South Korea	12	South Korea	12	France	12
United Kingdom	13	Egypt	13	Canada	13	Argentina	13	Vietnam	13	Mexico	13	South Korea	13
France	14	Denmark	14	Italy	14	Austria	14	France	14	France	14	Belgium	14
Poland	15	France	15	Hong Kong	15	United Kingdom	15	Mexico	15	Russia	15	Sweden	15

Russia	16	Norway	16	Japan	16	Thailand	16	Japan	16	Hong Kong	16	Germany	16
Greece	17	Greece	17	United Kingdom	17	Japan	17	Hong Kong	17	Japan	17	Hong Kong	17
Austria	18	Chile	18	Finland	18	Turkey	18	Taiwan	18	Belgium	18	Japan	18
Mexico	19	Netherlands	19	Austria	19	Egypt	19	Germany	19	Sweden	19	Malaysia	19
Czech Republic	20	Austria	20	Chile	20	Russia	20	Belgium	20	Czech Republic	20	Poland	20
Belgium	21	Poland	21	Netherlands	21	Portugal	21	Poland	21	Poland	21	Russia	21
Switzerland	22	Spain	22	Ireland	22	Chile	22	Czech Republic	22	Vietnam	22	Vietnam	22
Hungary	23	Germany	23	Norway	23	France	23	Sweden	23	Taiwan	23	Taiwan	23
Denmark	24	Belgium	24	Thailand	24	Ireland	24	Argentina	NA[b]	Argentina	NA[b]	Argentina	NA[b]
Sweden	25	Sweden	25	Switzerland	25	Germany	25	Australia	NA[b]	Australia	NA[b]	Australia	NA[b]
United States	26	Switzerland	26	Portugal	26	Malaysia	26	Brazil	NA[b]	Brazil	NA[b]	Brazil	NA[b]
Australia	NA[b]	United Kingdom	27	Poland	27	Spain	27	Canada	NA[b]	Canada	NA[b]	Canada	NA[b]
Brazil	NA[b]	Czech Republic	28	Greece	28	United States	28	Chile	NA[b]	Chile	NA[b]	Chile	NA[b]
Colombia	NA[b]	Hungary	29	France	29	Denmark	29	Colombia	NA[b]	Colombia	NA[b]	Colombia	NA[b]
Egypt	NA[b]	Brazil	30	Spain	30	Italy	30	Denmark	NA[b]	Denmark	NA[b]	Denmark	NA[b]
Hong Kong	NA[b]	Ireland	31	South Africa	31	Switzerland	31	Egypt	NA[b]	Egypt	NA[b]	Egypt	NA[b]
India	NA[b]	Canada	32	Sweden	32	Indonesia	32	Finland	NA[b]	Finland	NA[b]	Finland	NA[b]

Indonesia	NA[b]	Mexico	33	Colombia	33	Netherlands	33	Greece	NA[b]	Greece	NA[b]	Greece	NA[b]
Japan	NA[b]	United States	34	Belgium	34	South Africa	34	Hungary	NA[b]	Hungary	NA[b]	Hungary	NA[b]
Malaysia	NA[b]	Portugal	35	Hungary	35	Colombia	35	India	NA[b]	India	NA[b]	India	NA[b]
Netherlands	NA[b]	Australia	NA[b]	Germany	36	Norway	36	Indonesia	NA[b]	Indonesia	NA[b]	Indonesia	NA[b]
New Zealand	NA[b]	Colombia	NA[b]	Indonesia	37	Greece	37	Ireland	NA[b]	Ireland	NA[b]	Ireland	NA[b]
Pakistan	NA[b]	Hong Kong	NA[b]	Mexico	38	Mexico	38	Israel	NA[b]	Israel	NA[b]	Israel	NA[b]
Peru	NA[b]	Japan	NA[b]	United States	39	Brazil	39	Netherlands	NA[b]	Netherlands	NA[b]	Netherlands	NA[b]
Philippines	NA[b]	Malaysia	NA[b]	Philippines	40	Belgium	40	New Zealand	NA[b]	New Zealand	NA[b]	New Zealand	NA[b]
Qatar	NA[b]	New Zealand	NA[b]	Malaysia	41	Sweden	41	Norway	NA[b]	Norway	NA[b]	Norway	NA[b]
Saudi Arabia	NA[b]	Pakistan	NA[b]	Brazil	42	Finland	42	Pakistan	NA[b]	Pakistan	NA[b]	Pakistan	NA[b]
Singapore	NA[b]	Peru	NA[b]	Czech Republic	43	Philippines	43	Peru	NA[b]	Peru	NA[b]	Peru	NA[b]
South Africa	NA[b]	Philippines	NA[b]	Peru	44	Czech Republic	44	Qatar	NA[b]	Qatar	NA[b]	Qatar	NA[b]
South Korea	NA[b]	Qatar	NA[b]	Egypt	NA[b]	Poland	45	Saudi Arabia	NA[b]	Saudi Arabia	NA[b]	Saudi Arabia	NA[b]
Spain	NA[b]	South Africa	NA[b]	Pakistan	NA[b]	Peru	46	South Africa	NA[b]	South Africa	NA[b]	South Africa	NA[b]
Thailand	NA[b]	South Korea	NA[b]	Qatar	NA[b]	Hungary	47	Switzerland	NA[b]	Switzerland	NA[b]	Switzerland	NA[b]
Turkey	NA[b]	Thailand	NA[b]	Russia	NA[b]	Pakistan	NA[b]	Thailand	NA[b]	Thailand	NA[b]	Thailand	NA[b]
United Arab Emirates	NA[b]	United Arab Emirates	NA[b]	United Arab Emirates	NA[b]	Qatar	NA[b]	Turkey	NA[b]	Turkey	NA[b]	Turkey	NA[b]

Vietnam	NA[b]	Vietnam	NA[b]	Vietnam	NA[b]	United Arab Emirates	NA[b]	United Arab Emirates	NA[b]	United Arab Emirates	NA[b]	United Arab Emirates	NA[b]

Notes:

a. R means each country's ranking.

b. NA: not applicable. Indicates a country not ranked due to incomplete data.

Jul 2021	R[a]	Aug 2021	R[a]	Sep 2021	R[a]	Oct 2021	R[a]	Nov 2021	R[a]
Chile	1	Singapore	1	Denmark	1	Chile	1	Italy	1
Austria	2	Canada	2	Chile	2	Colombia	2	Malaysia	2
Hungary	3	Colombia	3	Colombia	3	Canada	3	Taiwan	3
Singapore	4	Chile	4	Spain	4	Peru	4	South Korea	4
Greece	5	Hungary	5	China	5	Brazil	5	Peru	5
China	6	Czech Republic	6	Portugal	6	Hong Kong	6	Japan	6
Czech Republic	7	Greece	7	Greece	7	China	7	Sweden	7
Norway	8	Argentina	8	Hungary	8	Sweden	8	Chile	8
Peru	9	Norway	9	Czech Republic	9	Switzerland	9	China	9
Canada	10	Portugal	10	South Korea	10	Israel	10	Canada	10
Switzerland	11	Peru	11	Peru	11	South Korea	11	Hong Kong	11
Sweden	12	Italy	12	France	12	Poland	12	Israel	12
Italy	13	Poland	13	Austria	13	Italy	13	Australia	13
South Korea	14	Sweden	14	Poland	14	Taiwan	14	Colombia	14
Saudi Arabia	15	South Korea	15	Hong Kong	15	Greece	15	Mexico	15
Japan	16	Hong Kong	16	Japan	16	Austria	16	Switzerland	16

Portugal	17	Austria	17	Italy	17	Malaysia	17	United States	17
Colombia	18	Saudi Arabia	18	Argentina	18	Australia	18	Russia	18
United States	19	China	19	Germany	19	Denmark	19	Finland	19
New Zealand	20	France	20	Canada	20	Turkey	20	Germany	20
Argentina	21	Denmark	21	Netherlands	21	Philippines	21	Netherlands	21
Hong Kong	22	Netherlands	22	India	22	Finland	22	Austria	22
Poland	23	Germany	23	Singapore	23	Japan	23	Ireland	23
Australia	24	Belgium	24	Belgium	24	Netherlands	24	Poland	24
Philippines	25	India	25	Philippines	25	Hungary	25	Hungary	25
Mexico	26	Mexico	26	New Zealand	26	Mexico	26	Czech Republic	26
Belgium	27	Spain	27	Saudi Arabia	27	Russia	27	Argentina	NA[b]
Germany	28	Australia	28	Taiwan	28	United States	28	Belgium	NA[b]
Denmark	29	Philippines	29	Finland	29	Czech Republic	29	Brazil	NA[b]
India	30	Finland	30	Brazil	30	Ireland	30	Denmark	NA[b]
Israel	31	New Zealand	31	Norway	31	Germany	31	Egypt	NA[b]
France	32	Japan	32	Turkey	32	Belgium	32	France	NA[b]
Finland	33	United States	33	Ireland	33	United Kingdom	33	Greece	NA[b]
Brazil	34	Ireland	34	Sweden	34	Argentina	NA[b]	India	NA[b]

Turkey	35	Switzerland	35	Russia	35	Egypt	NA[b]	Indonesia	NA[b]
Ireland	36	United Kingdom	36	Switzerland	36	France	NA[b]	New Zealand	NA[b]
Netherlands	37	Israel	37	United Kingdom	37	India	NA[b]	Norway	NA[b]
Spain	38	Brazil	38	United States	38	Indonesia	NA[b]	Pakistan	NA[b]
United Kingdom	39	Russia	39	Israel	39	New Zealand	NA[b]	Philippines	NA[b]
Thailand	40	Turkey	40	Thailand	40	Norway	NA[b]	Portugal	NA[b]
Russia	41	Thailand	41	Australia	41	Pakistan	NA[b]	Qatar	NA[b]
Malaysia	42	Malaysia	42	Malaysia	42	Portugal	NA[b]	Saudi Arabia	NA[b]
South Africa	43	South Africa	43	Mexico	43	Qatar	NA[b]	Singapore	NA[b]
Taiwan	44	Taiwan	44	South Africa	44	Saudi Arabia	NA[b]	South Africa	NA[b]
Vietnam	45	Vietnam	45	Vietnam	45	Singapore	NA[b]	Spain	NA[b]
Egypt	NA[b]	Egypt	NA[b]	Egypt	NA[b]	South Africa	NA[b]	Thailand	NA[b]
Indonesia	NA[b]	Indonesia	NA[b]	Indonesia	NA[b]	Spain	NA[b]	Turkey	NA[b]
Pakistan	NA[b]	Pakistan	NA[b]	Pakistan	NA[b]	Thailand	NA[b]	United Arab Emirates	NA[b]
Qatar	NA[b]	Qatar	NA[b]	Qatar	NA[b]	United Arab Emirates	NA[b]	United Kingdom	NA[b]
United Arab Emirates	NA[b]	United Arab Emirates	NA[b]	United Arab Emirates	NA[b]	Vietnam	NA[b]	Vietnam	NA[b]

Notes:

a. R means each country’s ranking.

b. NA: not applicable. Indicates a country not ranked due to incomplete data.

Supplementary Information 9. Different baseline risk levels of 50 countries and territories and their respective COVID-19 containment scores (with Google Trends) from March 2020 to November 2021

To obtain the baseline risk score for each of the 50 nations, we take the average of the three risk factors in Supplementary Table S1: obesity prevalence, share of population over the age of 65, and international arrivals. After acquiring the average baseline risk score for each country, we characterize the three diverse baseline risk levels — Low, Medium, and High — by subtracting the minimum baseline risk score from the maximum baseline risk score among the 50 nations and dividing the difference into three equal portions.

Supplementary Figures S11–S31 demonstrate the three different COVID-19 baseline risk levels and each country's respective COVID-19 containment score (with Google Trends) for the 50 nations of interest during the period of March 2020- November 2021. Most of the European countries are characterized to be at the high baseline risk level, while the Asian and African countries are characterized to be at low or middle baseline risk levels. However, the American nations were found to be evenly distributed across the three distinct baseline risk levels. In addition, from these graphs, it is evident that most Asian nations have higher COVID-19 containment scores than those in Europe or the Americas before March 2021.

Supplementary Figure S32a shows the monthly average containment performance scores of all 50 countries grouped according to the three baseline risk levels. Supplementary Figure S32b shows the monthly average people covered by vaccines grouped according to the three baseline risk levels.

Supplementary Figure S11. **Different baseline risk levels of 50 countries and territories and their respective COVID-19 containment scores (with Google Trends) in March 2020.**

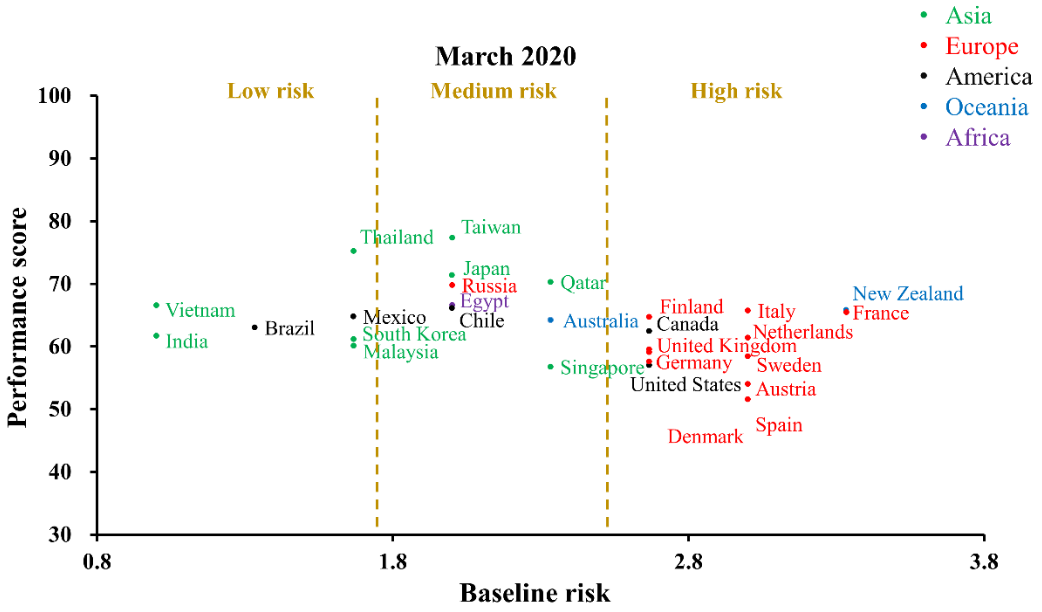

Note: some places might not be included due to missing data.

Supplementary Figure S12. **Different baseline risk levels of 50 countries and territories and their respective COVID-19 containment scores (with Google Trends) in April 2020**

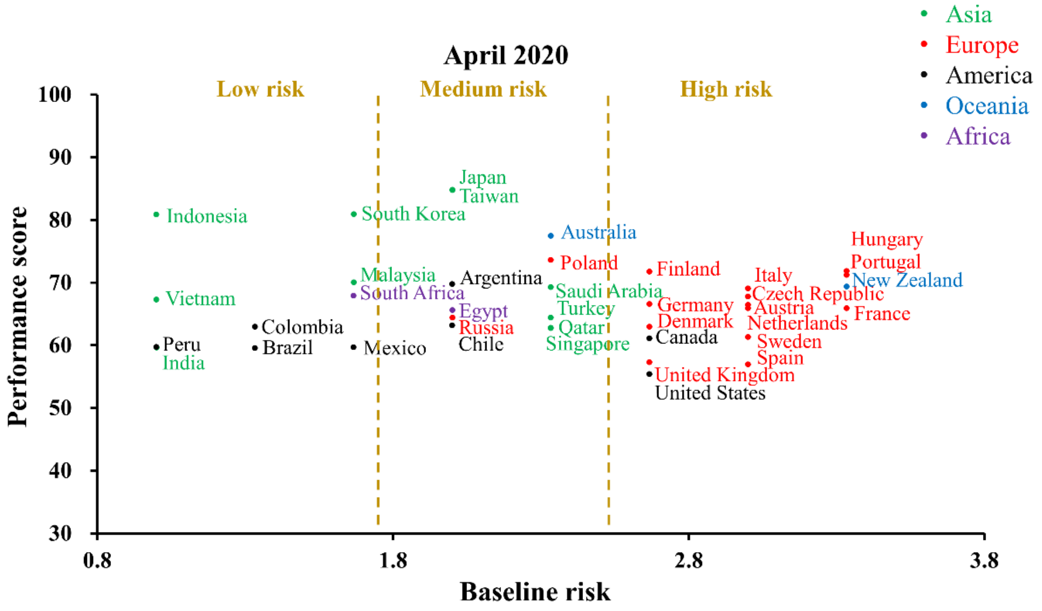

Note: some places might not be included due to missing data.

Supplementary Figure S13. **Different baseline risk levels of 50 countries and territories and their respective COVID-19 containment scores (with Google Trends) in May 2020**

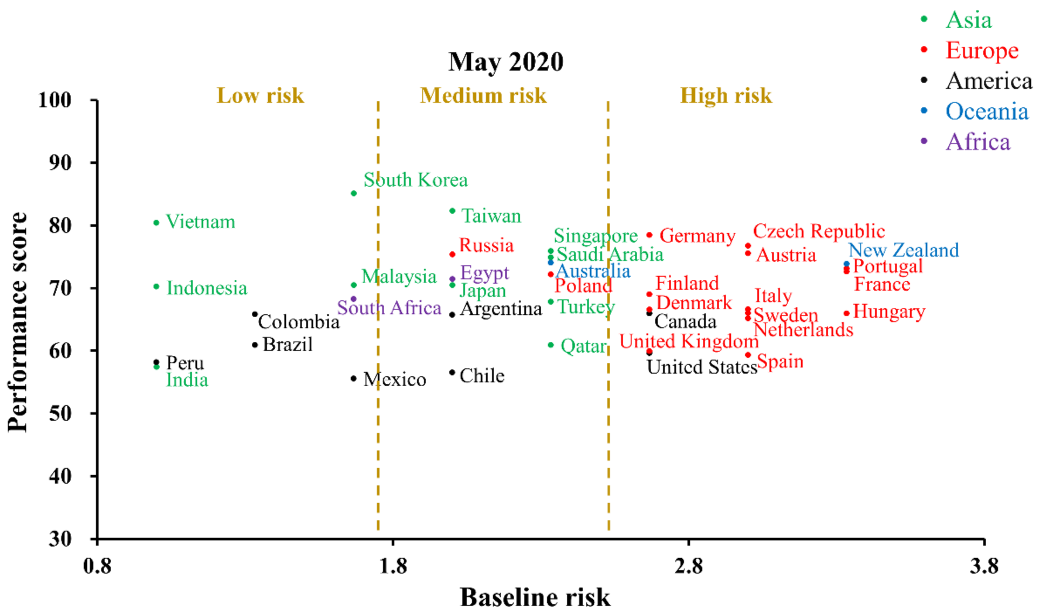

Note: some places might not be included due to missing data.

Supplementary Figure S14. **Different baseline risk levels of 50 countries and territories and their respective COVID-19 containment scores (with Google Trends) in June 2020**

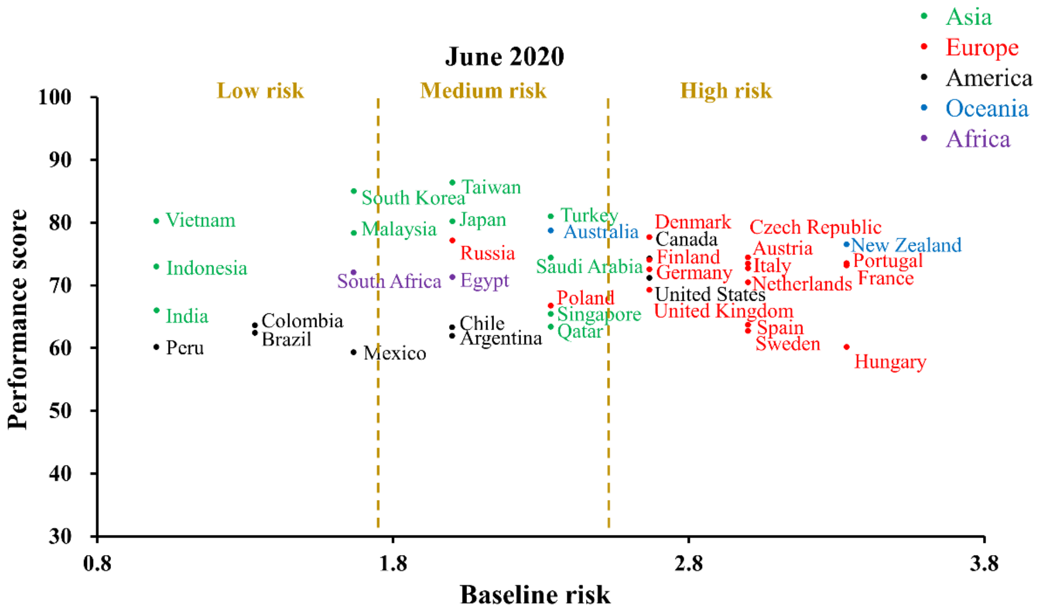

Note: some places might not be included due to missing data.

Supplementary Figure S15. **Different baseline risk levels of 50 countries and territories and their respective COVID-19 containment scores (with Google Trends) in July 2020**

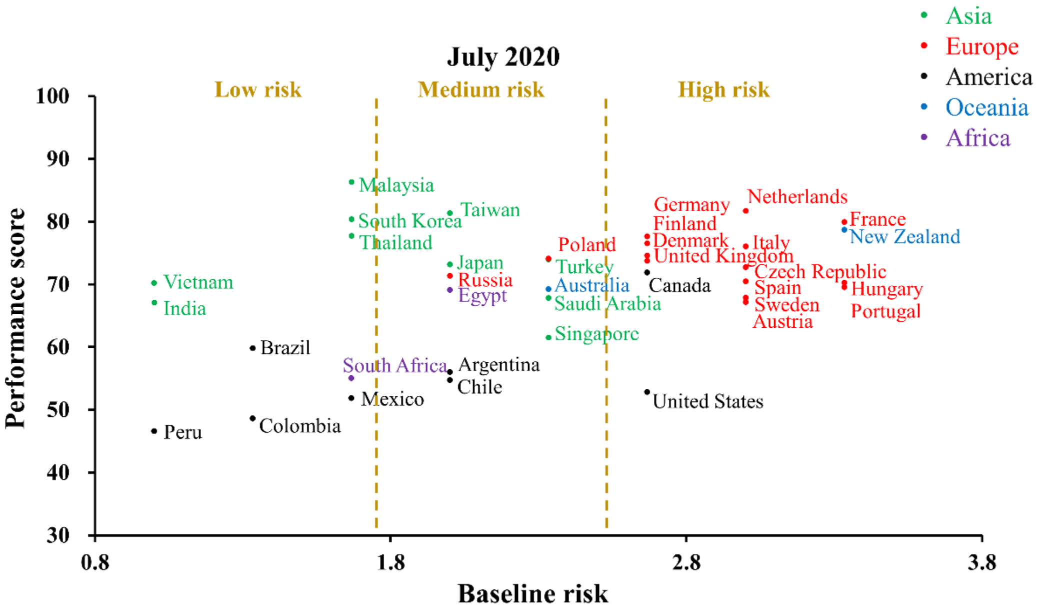

Note: some places might not be included due to missing data.

Supplementary Figure S16. **Different baseline risk levels of 50 countries and territories and their respective COVID-19 containment scores (with Google Trends) in August 2020**

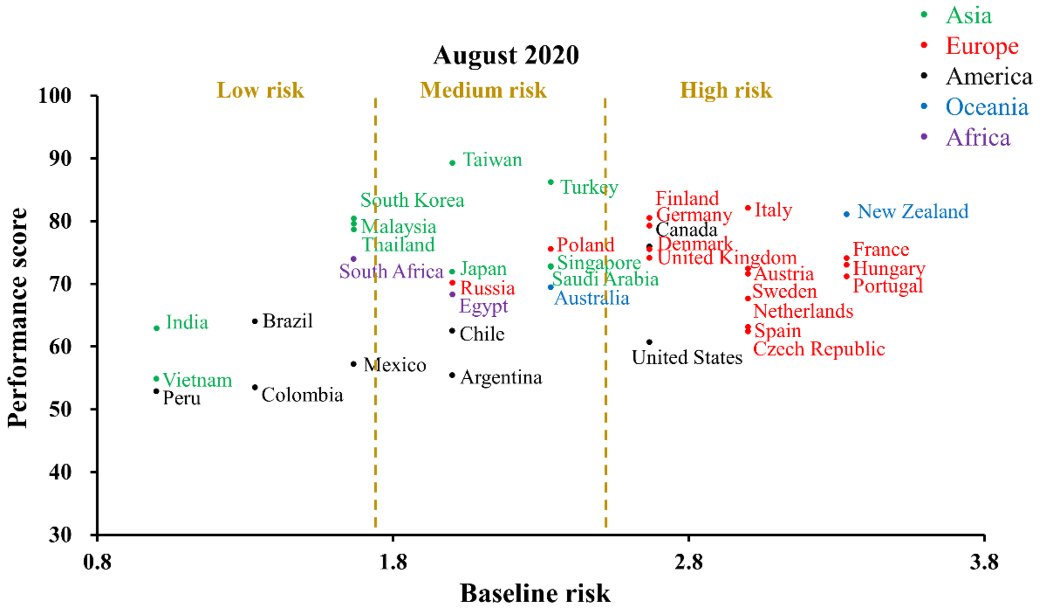

Note: some places might not be included due to missing data.

Supplementary Figure S17. **Different baseline risk levels of 50 countries and territories and their respective COVID-19 containment scores (with Google Trends) in September 2020**

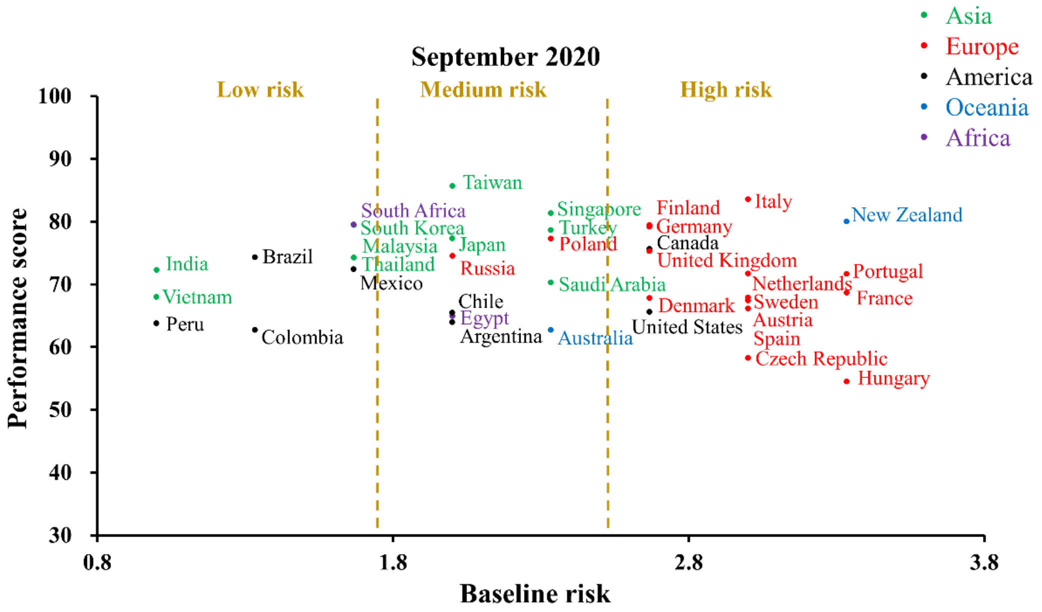

Note: some places might not be included due to missing data.

Supplementary Figure S18. **Different baseline risk levels of 50 countries and territories and their respective COVID-19 containment scores (with Google Trends) in October 2020**

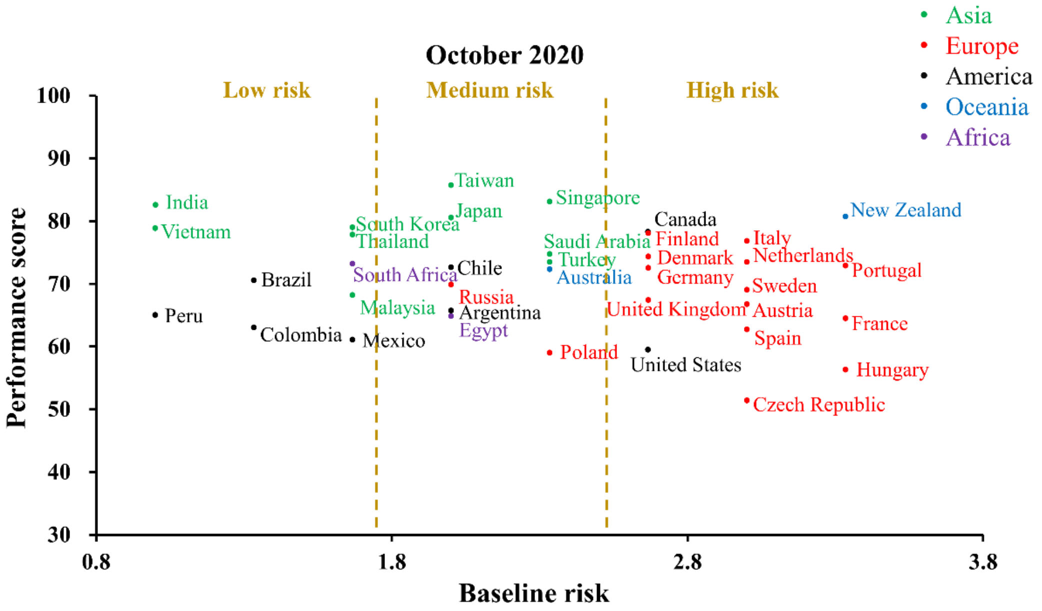

Note: some places might not be included due to missing data.

Supplementary Figure S19. **Different baseline risk levels of 50 countries and territories and their respective COVID-19 containment scores (with Google Trends) in November 2020**

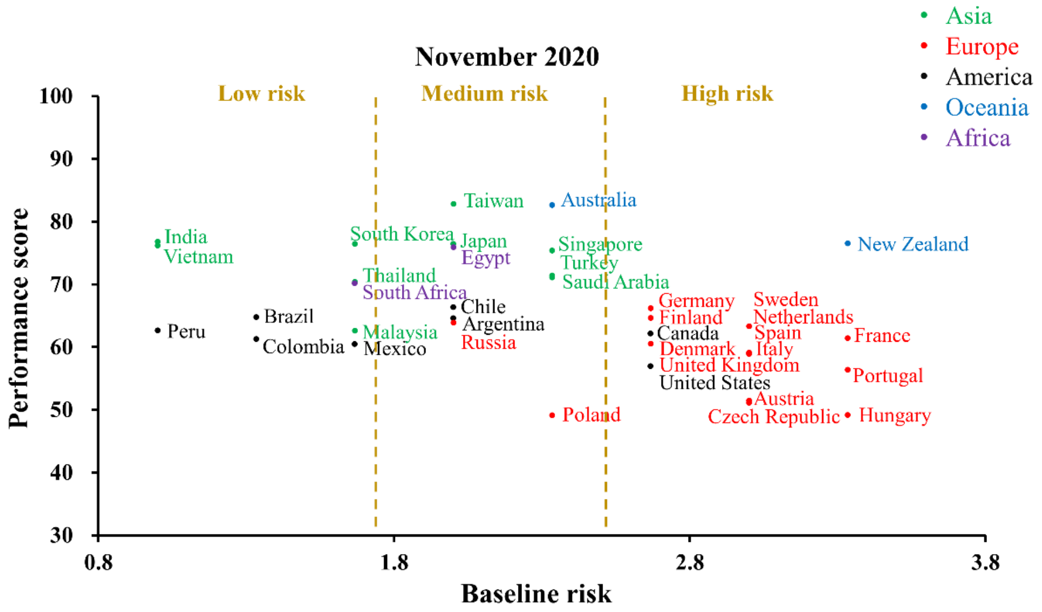

Note: some places might not be included due to missing data.

Supplementary Figure S20. **Different baseline risk levels of 50 countries and territories and their respective COVID-19 containment scores (with Google Trends) in December 2020**

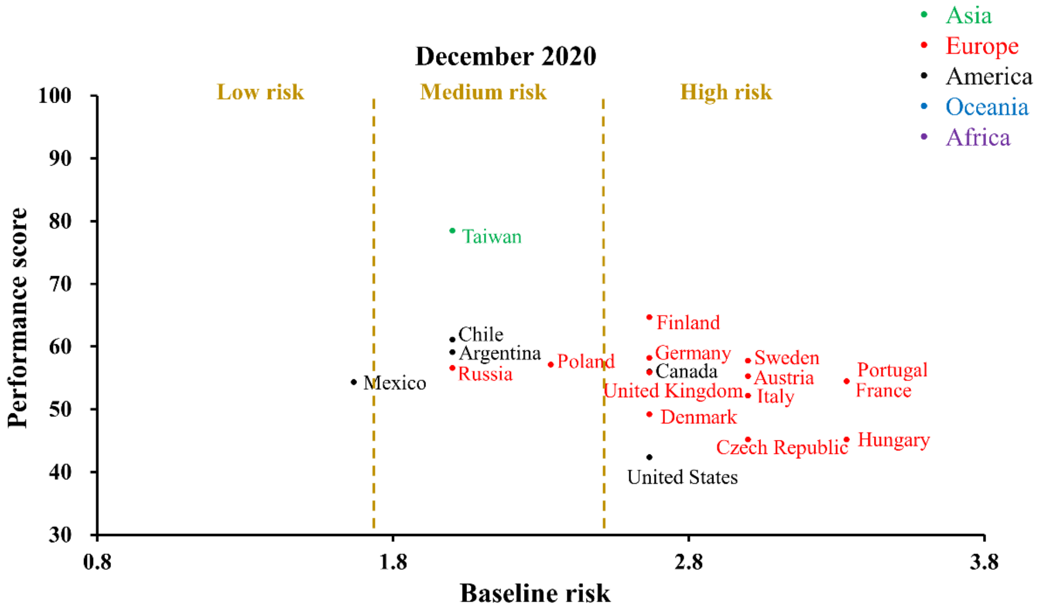

Note: some places might not be included due to missing data.

Supplementary Figure S21. **Different baseline risk levels of 50 countries and territories and their respective COVID-19 containment scores (with Google Trends) in January 2021**

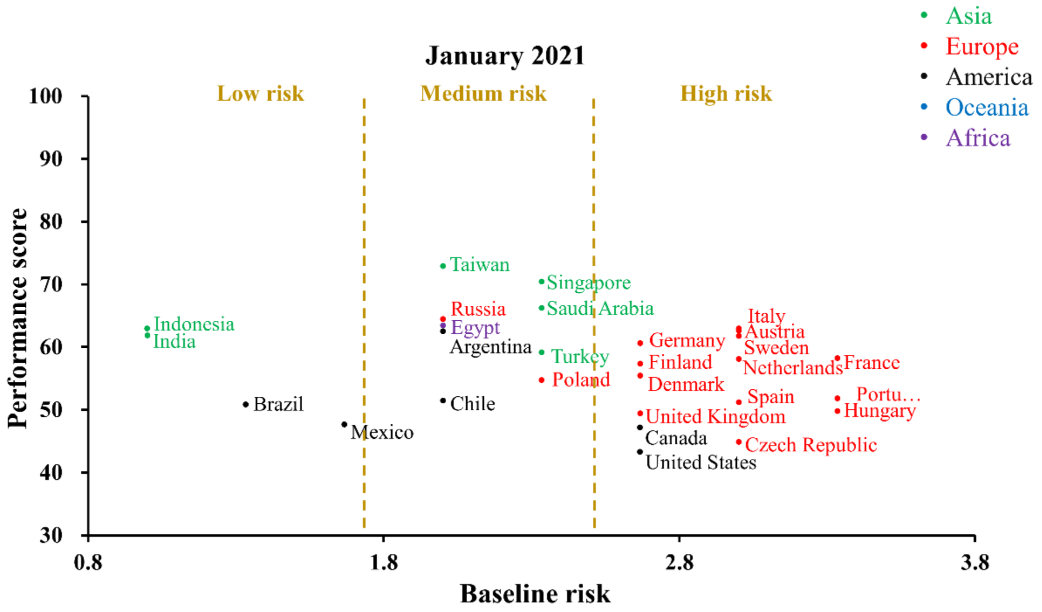

Note: some places might not be included due to missing data.

Supplementary Figure S22. **Different baseline risk levels of 50 countries and territories and their respective COVID-19 containment scores (with Google Trends) in February 2021**

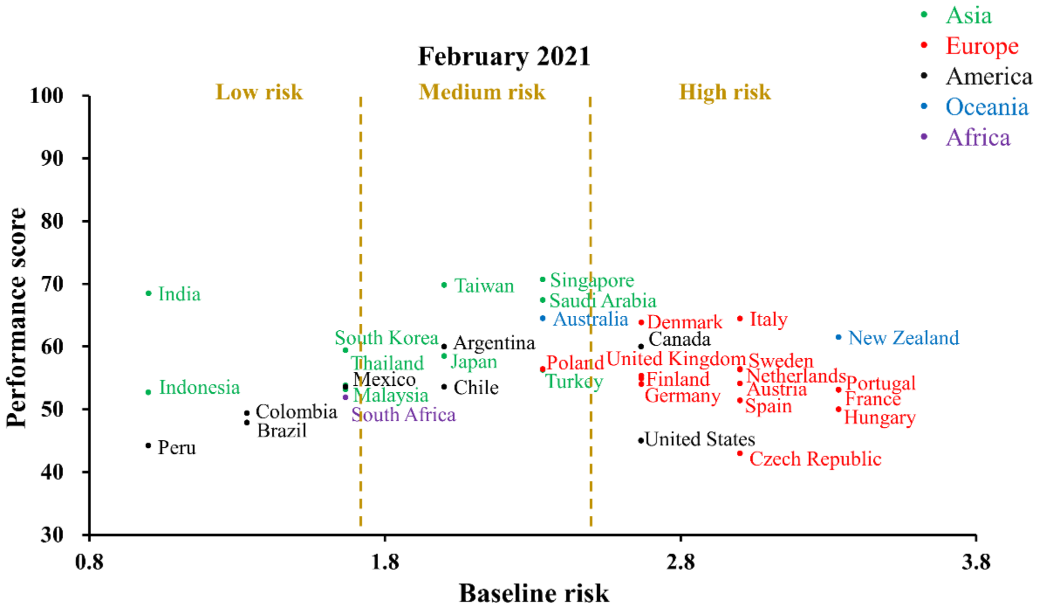

Note: some places might not be included due to missing data.

Supplementary Figure S23. **Different baseline risk levels of 50 countries and territories and their respective COVID-19 containment scores (with Google Trends) in March 2021**

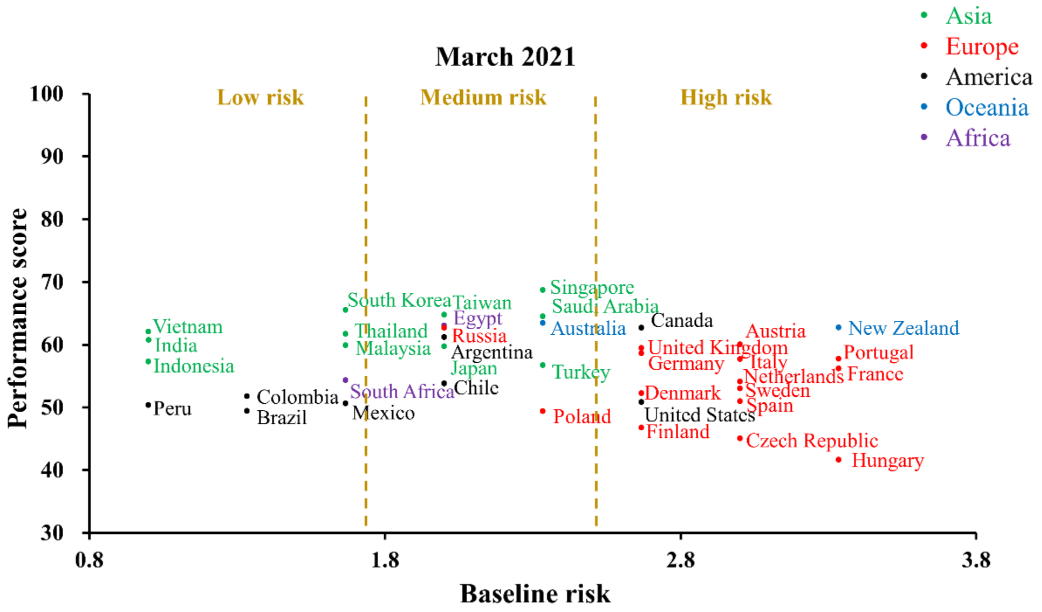

Note: some places might not be included due to missing data.

Supplementary Figure S24. **Different baseline risk levels of 50 countries and territories and their respective COVID-19 containment scores (with Google Trends) in April 2021**

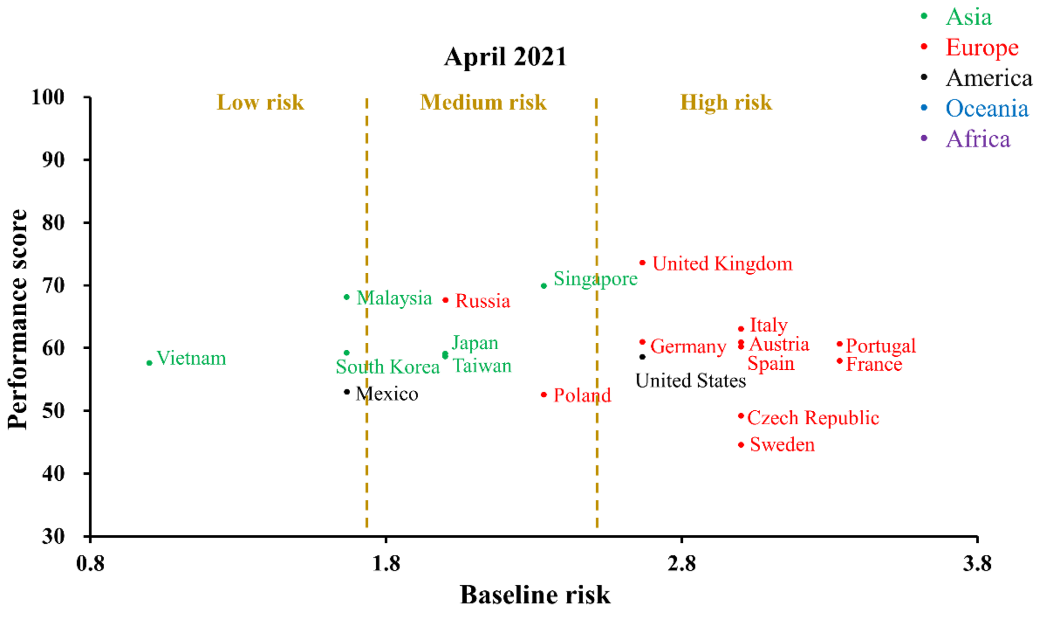

Note: some places might not be included due to missing data.

Supplementary Figure S25. **Different baseline risk levels of 50 countries and territories and their respective COVID-19 containment scores (with Google Trends) in May 2021**

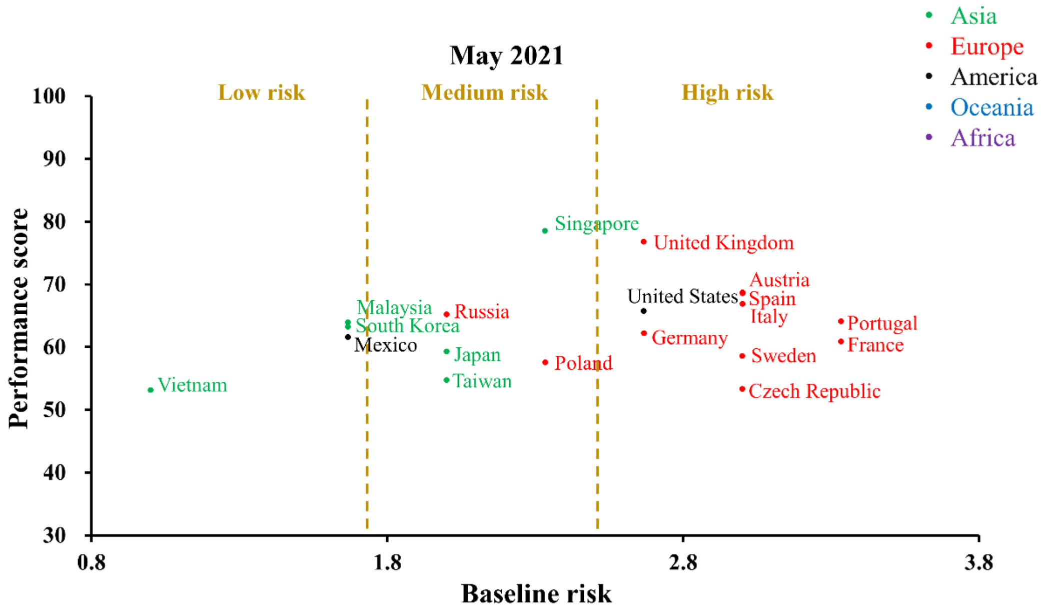

Note: some places might not be included due to missing data.

Supplementary Figure S26. **Different baseline risk levels of 50 countries and territories and their respective COVID-19 containment scores (with Google Trends) in June 2021**

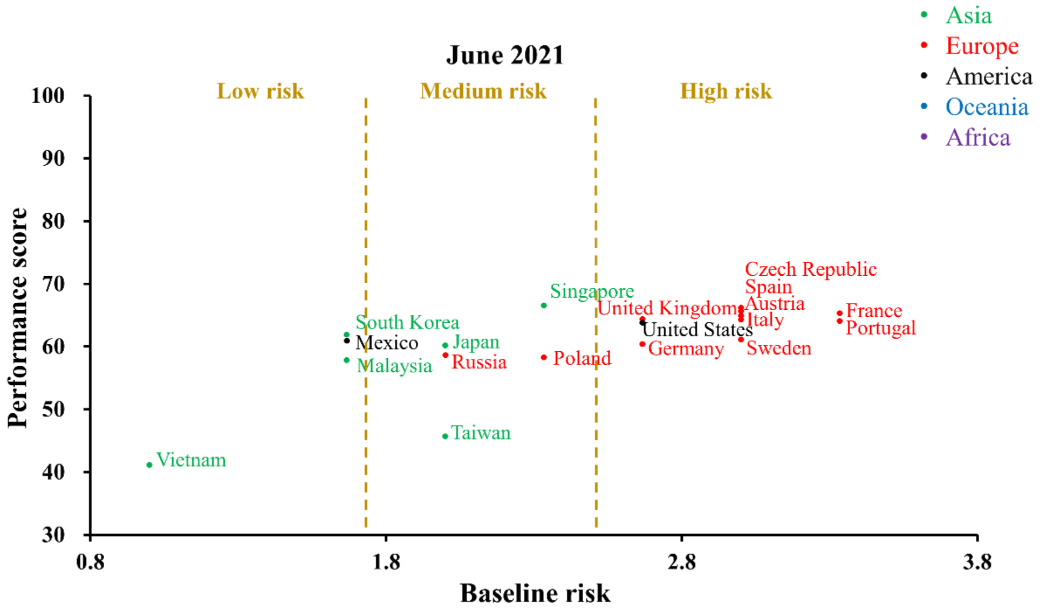

Note: some places might not be included due to missing data.

Supplementary Figure S27. **Different baseline risk levels of 50 countries and territories and their respective COVID-19 containment scores (with Google Trends) in July 2021**

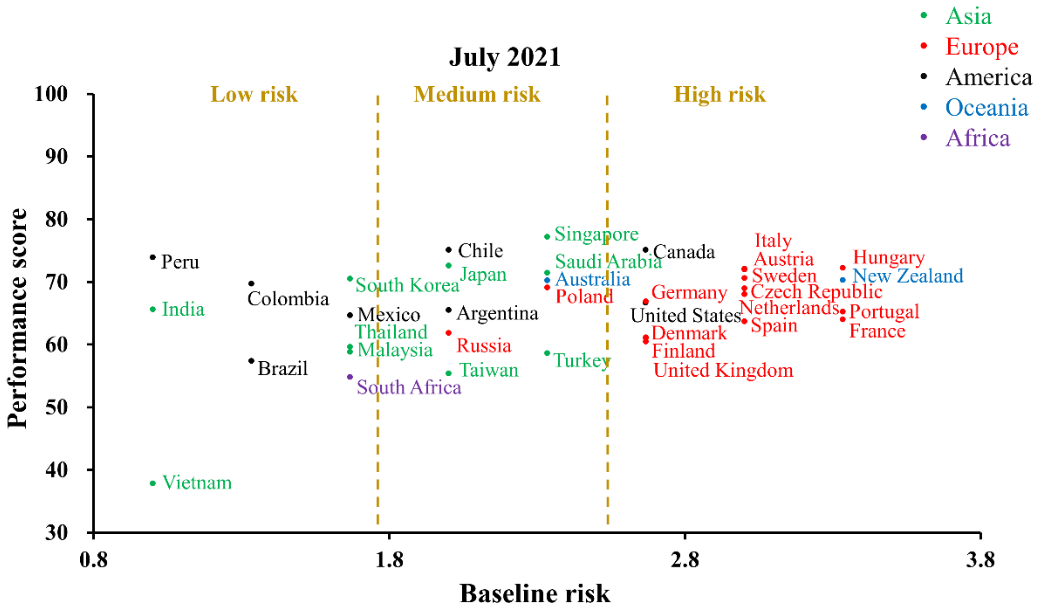

Note: some places might not be included due to missing data.

Supplementary Figure S28. **Different baseline risk levels of 50 countries and territories and their respective COVID-19 containment scores (with Google Trends) in August 2021**

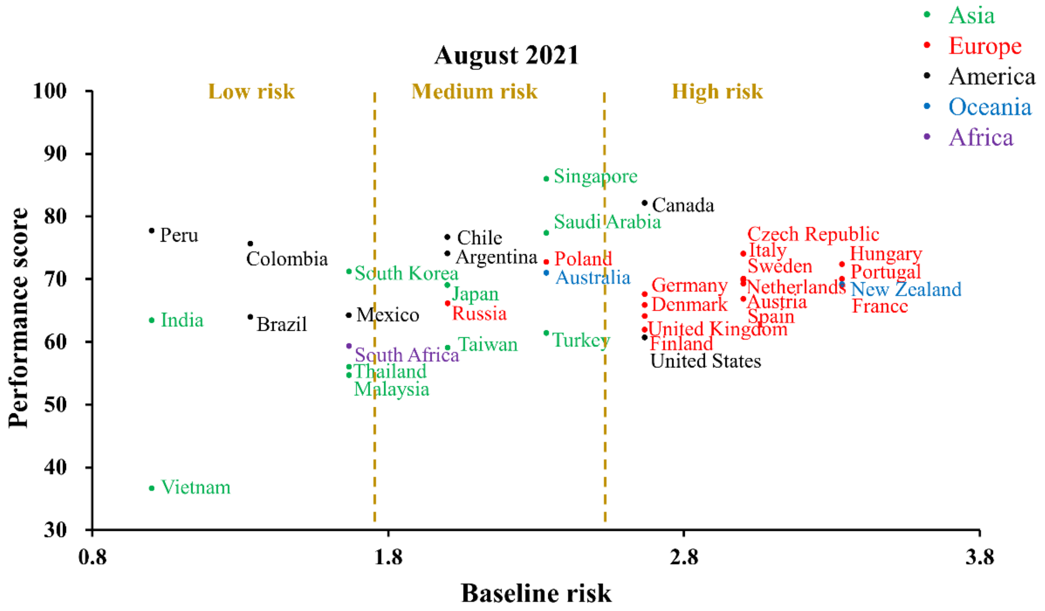

Note: some places might not be included due to missing data.

Supplementary Figure S29. **Different baseline risk levels of 50 countries and territories and their respective COVID-19 containment scores (with Google Trends) in September 2021**

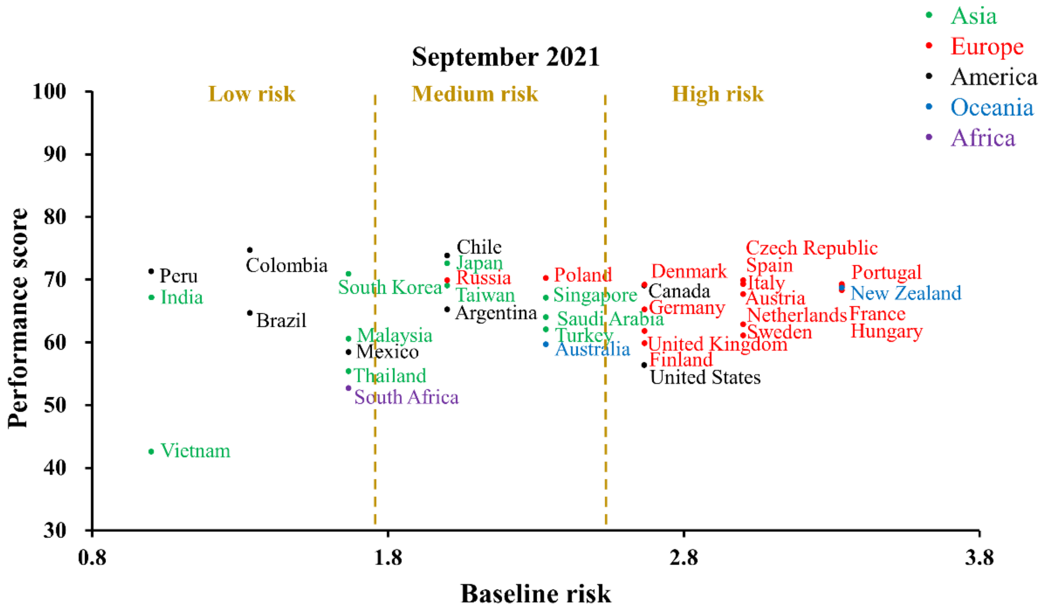

Note: some places might not be included due to missing data.

Supplementary Figure S30. **Different baseline risk levels of 50 countries and territories and their respective COVID-19 containment scores (with Google Trends) in October 2021**

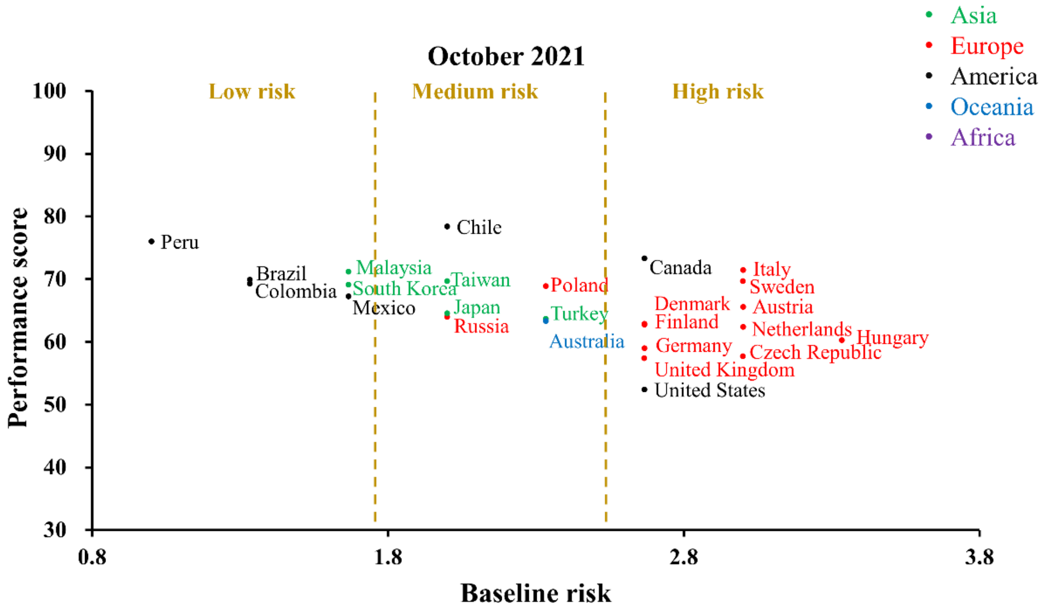

Note: some places might not be included due to missing data.

Supplementary Figure S31. **Different baseline risk levels of 50 countries and territories and their respective COVID-19 containment scores (with Google Trends) in November 2021**

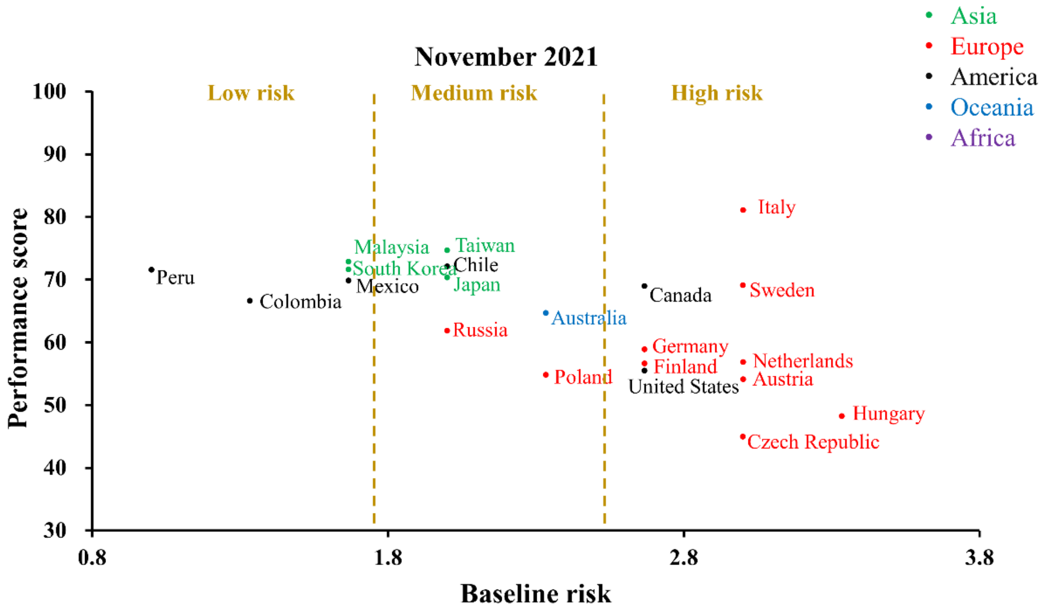

Note: some places might not be included due to missing data.

Supplementary Figure S32a. **The comparison of performance scores among three levels of baseline risk from March 2020 to November 2021, with vaccination indicator only from December 2020 to November 2021 (marked light blue area).**

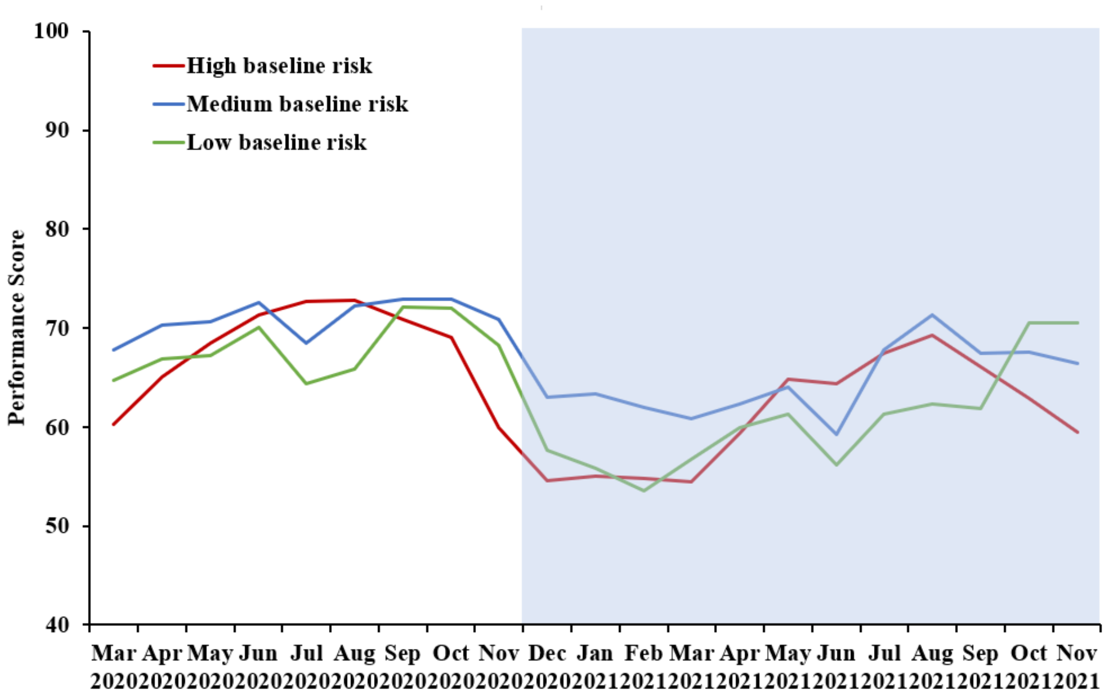

Supplementary Figure S32b. **The comparison of people covered by vaccines among three levels of baseline risk from December 2020 to November 2021**

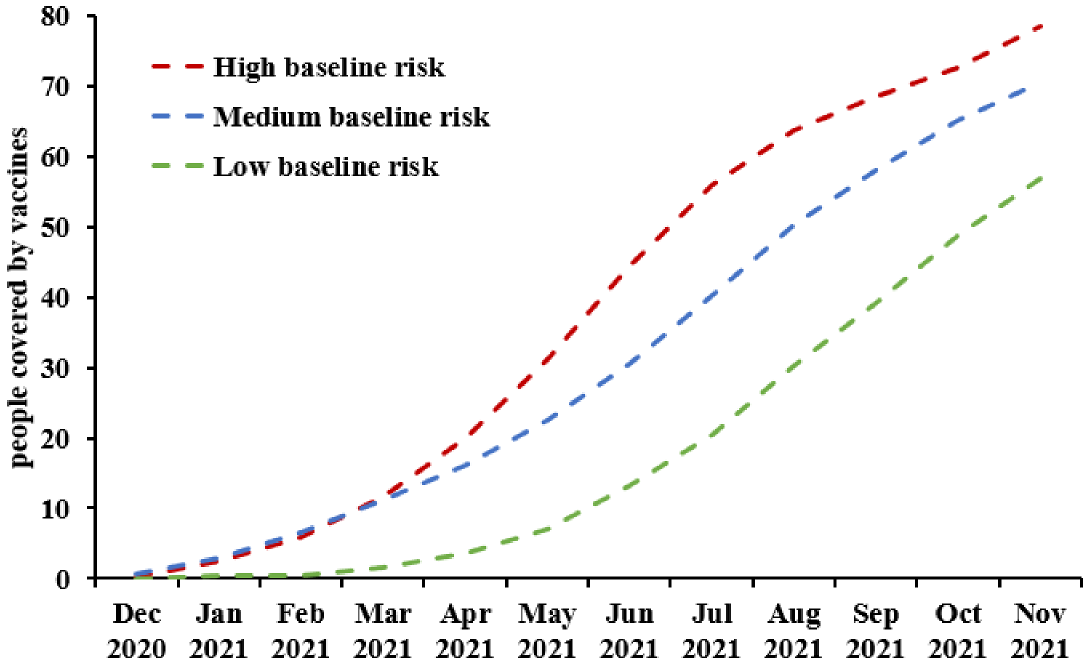

Supplementary Information 10. Two-sample t-test and Mann-Whitney U test of comparison between Latin American and non-Latin American nations

To compare the COVID-19 performance between Latin American nations and non-Latin American countries, we perform two-sample t-test and Mann–Whitney U test. The analyses of the differences in performance score with/without Google Trends show that non-Latin American nations have better overall COVID-19 containment performance than Latin American countries (Supplementary Table S8). From April 2020 to September 2020, the differences in performance score without Google Trends between the two groups of countries reached statistical significance ($P < 0.003$). Similarly, the differences in performance score with Google Trends reached statistical significance during the months April 2020 through October 2020 ($P < 0.027$).

In addition, we examine the differences between Latin American nations and non-Latin American countries in each of the government policy & hygiene education measurements (Supplementary Table S9). There is no statistically significant difference in lockdown efficiency between the two groups in the nine-month period. In terms of differences in health-system policies, non-Latin American nations performed better than their Latin American counterparts in March 2020 ($P = 0.005$). We did not observe statistically significant differences in health-system policies between the two groups in other periods. The analysis of the differences in health literacy shows that except in April 2020 and June 2020, when Latin American nations performed statistically better than others ($P < 0.032$), there are no statistically significant differences between the two groups in other months.

To uncover the influence of COVID-19 pandemic on the society, economy, and citizens' mental health, we compare the differences in six vital health and socioeconomic indicators between Latin American nations and non-Latin American countries (Supplementary Table S10).

The differences between Latin American nations and others in three key COVID-19 outcome-based indicators are analyzed (Supplementary Table S10). In the beginning of the pandemic (in March 2020 and April 2020), Latin American nations have lower one-month cases per 100,000 members of the populations than others, but these differences do not meet the statistical significance. From May 2020 to September 2020, Latin American nations have higher one-month cases per 100,000 members of the population than their non-Latin American peers ($P < 0.027$). However, in October 2020 and November 2020, there are no statistical differences between the two groups in one-month cases per 100,000 members of the population. In terms of the differences in infection growth rate, Latin American countries have higher infection growth rates than non-Latin American nations during the months from March 2020 to August 2020; except in March 2020 and August 2020, the differences in other months reach the statistical significance ($P < 0.011$). However, from September 2020 to November 2020, the

difference between the two groups of countries was not statistically significant. There are no significant differences between the Latin American nations and others in respect to one-month case fatality rate from March 2020 to June 2020; however, from July 2020 to November 2020, Latin American countries have higher one-month case fatality rate than non-Latin American nations ($P < 0.008$).

Our analysis discovers that non-Latin American nations performed better than Latin American countries in two economic indicators (Supplementary Table S10). From April 2020 to September 2020, non-Latin nations have lower GDP loss than Latin American states ($P < 0.02$). Similarly, from April 2020 to November 2020, the increase in unemployment rate in non-Latin American countries was lower than that in Latin American states. The difference between the two groups reached statistical significance from June 2020 to August 2020 and October 2020 to November 2020 ($P < 0.007$).

The comparison of the impact of the pandemic on citizens' mental health between Latin American nations and others is measured by the difference in searches for insomnia between the two groups in the nine-month period (Supplementary Table S10). From April 2020 to June 2020, the increase in Google searches for "insomnia" in Latin America nations was higher than their counterparts ($P < 0.027$), while the difference between the two groups of nations in other periods was not significant.

Supplementary Table S8. **Comparison of the overall containment effectiveness between Latin American and non-Latin American countries from March to November 2020**

	Mar 2020		Apr 2020		May 2020		Jun 2020		Jul 2020		Aug 2020		Sep 2020		Oct 2020		Nov 2020	
	N	Mean (SD) Median (IQR)	N	Mean (SD) Median (IQR)	N	Mean (SD) Median (IQR)	N	Mean (SD) Median (IQR)	N	Mean (SD) Median (IQR)	N	Mean (SD) Median (IQR)	N	Mean (SD) Median (IQR)	N	Mean (SD) Median (IQR)	N	Mean (SD) Median (IQR)
**Performance score without Google Trends**																		
Latin America	3	64.92 (10.39) 62.86 (20.48)	6	54.09 (4.87) 54.29 (1.67)	6	53.13 (4.21) 53.45 (3.57)	6	48.97 (5.79) 47.14 (5.24)	6	46.15 (4.40) 44.05 (7.14)	6	46.90 (4.41) 47.14 (8.81)	6	49.09 (5.57) 48.10 (7.14)	6	57.42 (6.22) 60.12 (9.76)	6	59.52 (5.25) 60.60 (3.57)
Non-Latin America	34	60.94 (12.29) 59.17 (21.43)	41	63.61 (12.82) 61.19 (15.24)	41	66.98 (12.83) 66.90 (15.95)	41	69.78 (12.98) 71.19 (16.90)	40	70.81 (12.85) 68.57 (21.90)	40	68.69 (11.94) 68.10 (14.76)	40	68.79 (11.87) 66.43 (16.43)	40	67.50 (15.11) 61.90 (24.64)	40	67.57 (15.57) 62.38 (28.21)
P value[a]		0.591		**0.003**		**<.0001**		**0.0004**		**<.0001**		**<.0001**		**0.0003**		0.221[b]		0.525[b]
**Performance score with Google Trends**																		
Latin America	3	66.23 (4.83) 64.17 (8.98)	6	56.42 (3.78) 57.78 (2.69)	6	55.68 (2.91) 54.72 (4.26)	6	54.06 (2.91) 54.07 (2.78)	6	52.79 (4.60) 52.69 (6.20)	6	53.38 (3.05) 51.99 (2.78)	6	55.54 (4.80) 55.42 (6.48)	6	60.40 (4.66) 62.08 (7.78)	6	64.58 (4.70) 66.57 (6.94)

Non-Latin America	27	64.65<br>(9.74)	34	65.13<br>(10.20)	34	67.95<br>(9.93)	34	71.20<br>(10.31)	33	72.60<br>(10.18)	33	70.63<br>(10.10)	33	71.19<br>(10.09)	33	70.75<br>(12.19)	33	70.79<br>(12.23)
		63.61<br>(15.93)		64.81<br>(14.72)		67.59<br>(9.07)		70.05<br>(13.89)		71.39<br>(16.67)		69.26<br>(15.93)		68.70<br>(11.67)		70.09<br>(18.06)		67.31<br>(21.57)
P value[a]		0.786		**0.027**[b]		**<.0001**		**<.0001**		**0.0002**[b]		**<.0001**		**0.001**		**0.002**		0.371[b]

[a]Unless otherwise noted, the two-sample t test is used to compare the differences between Latin American and non-Latin American countries

[b]The Mann-Whitney U test is used to compare the differences between Latin American and non-Latin American countries

Supplementary Table S9. **Comparison of government policy & hygiene education indicators between Latin American and non-Latin American countries from March 2020 to November 2020**

	Mar 2020		Apr 2020		May 2020		Jun 2020		Jul 2020		Aug 2020		Sep 2020		Oct 2020		Nov 2020	
	N	Mean (SD) Median (IQR)	N	Mean (SD) Median (IQR)	N	Mean (SD) Median (IQR)	N	Mean (SD) Median (IQR)	N	Mean (SD) Median (IQR)	N	Mean (SD) Median (IQR)	N	Mean (SD) Median (IQR)	N	Mean (SD) Median (IQR)	N	Mean (SD) Median (IQR)
**Lockdown efficiency**																		
Latin America	3	3.33 (1.15)	6	2.00 (0.63)	6	2.83 (0.41)	6	3.00 (0.00)	6	3.00 (0.00)	6	3.00 (0.00)	6	3.00 (0.00)	6	2.83 (0.41)	6	2.17 (0.75)
		4.00 (2.00)		2.00 (0.00)		3.00 (0.00)		3.00 (0.00)		3.00 (0.00)		3.00 (0.00)		3.00 (0.00)		3.00 (0.00)		2.00 (1.00)
Non-Latin America	34	2.44 (1.11)	41	2.29 (1.17)	41	2.76 (1.18)	41	3.10 (1.04)	40	3.00 (1.11)	40	2.58 (1.20)	40	2.58 (1.20)	40	2.65 (1.17)	40	2.78 (1.12)
		3.00 (2.00)		2.00 (2.00)		3.00 (2.00)		3.00 (1.00)		3.00 (2.00)		2.50 (2.50)		2.50 (2.50)		3.00 (2.00)		3.00 (2.00)
P value[a]		0.183		0.703		0.868		0.367		0.546		0.489		0.489		0.853		0.160
**Health-system policies**																		
Latin America	3	1.51 (0.15)	6	2.98 (0.59)	6	3.38 (0.28)	6	3.38 (0.28)	6	3.42 (0.26)	6	3.47 (0.29)	6	3.24 (0.49)	6	3.24 (0.46)	6	3.33 (0.28)
		1.60 (0.27)		3.20 (0.80)		3.20 (0.53)		3.20 (0.53)		3.33 (0.53)		3.47 (0.53)		3.20 (0.53)		3.33 (0.27)		3.33 (0.27)

Non-Latin America	34	2.42 (0.41)	41	2.71 (0.54)	41	3.00 (0.50)	41	3.15 (0.41)	40	3.23 (0.35)	40	3.33 (0.36)	40	3.36 (0.34)	40	3.40 (0.37)	40	3.42 (0.39)
		2.40 (0.53)		2.67 (1.07)		2.93 (0.80)		3.20 (0.53)		3.20 (0.40)		3.47 (0.27)		3.47 (0.27)		3.47 (0.53)		3.47 (0.53)
P value[a]		**0.005**		0.292		0.058		0.228		0.236		0.430		0.588		0.449		0.462
									**Health literacy**									
Latin America	3	48.07 (2.57)	6	51.04 (16.40)	6	34.67 (15.12)	6	27.77 (10.99)	6	21.98 (10.15)	6	19.42 (7.05)	6	20.82 (10.06)	6	15.44 (6.71)	6	13.85 (4.58)
		47.40 (5.00)		46.75 (12.25)		29.69 (25.00)		28.05 (13.00)		22.06 (13.75)		18.75 (10.63)		20.95 (10.80)		13.88 (11.50)		14.31 (5.88)
Non-Latin America	27	43.68 (17.88)	34	33.84 (16.70)	34	25.64 (11.47)	34	18.28 (9.42)	33	19.73 (11.74)	33	18.03 (8.88)	33	16.68 (6.57)	33	18.36 (10.33)	33	14.45 (9.54)
		43.90 (33.50)		30.31 (29.50)		24.00 (15.63)		19.70 (15.20)		16.00 (16.63)		17.25 (12.25)		17.20 (5.70)		17.50 (12.25)		12.75 (8.50)
P value[b]		0.252		**0.025**		0.098		**0.032**		0.546[a]		0.720		0.200		0.586[a]		0.770[a]

[a]The Mann-Whitney U test is used to compare the differences between Latin American and non-Latin American countries

[b]Unless otherwise noted, the two-sample t test is used to compare the differences between Latin American and non-Latin American countries

Supplementary Table S10. **Comparison of vital health & socioeconomic indicators between Latin American and non-Latin American countries from March 2020 to November 2020**

	Mar 2020		Apr 2020		May 2020		Jun 2020		Jul 2020		Aug 2020		Sep 2020		Oct 2020		Nov 2020	
	N	Mean (SD) Median (IQR)	N	Mean (SD) Median (IQR)	N	Mean (SD) Median (IQR)	N	Mean (SD) Median (IQR)	N	Mean (SD) Median (IQR)	N	Mean (SD) Median (IQR)	N	Mean (SD) Median (IQR)	N	Mean (SD) Median (IQR)	N	Mean (SD) Median (IQR)
**One-month cases per 100,000 members of the population**																		
**Latin America**	3	6.16 (7.59)	6	42.90 (41.72)	6	211.58 (216.27)	6	323.98 (276.91)	6	364.30 (145.18)	6	478.50 (222.72)	6	410.43 (212.02)	6	399.68 (278.74)	6	329.28 (171.86)
		2.69 (13.93)		26.15 (66.72)		128.30 (341.74)		250.37 (311.89)		379.66 (118.51)		543.55 (334.83)		423.19 (231.04)		304.53 (233.27)		296.76 (287.72)
**Non-Latin America**	34	48.07 (56.27)	41	101.08 (110.80)	41	88.02 (238.61)	41	82.69 (219.74)	40	79.17 (147.98)	40	86.72 (115.94)	40	159.20 (262.48)	40	447.84 (621.46)	40	608.69 (659.54)
		24.04 (71.59)		60.87 (156.19)		24.35 (67.13)		18.97 (37.62)		29.34 (63.38)		55.82 (88.43)		79.01 (180.91)		200.62 (646.37)		302.10 (1204.00)
P value[a]		0.211		0.493		**0.027**		**0.002**		**0.001**		**0.0004**		**0.004**		0.406		0.935
**Infection growth rate**																		
**Latin America**	3	152708.33 (128067.45)	6	1289.99 (1128.78)	6	406.17 (116.28)	6	172.86 (75.31)	6	107.73 (75.03)	6	64.85 (40.18)	6	33.27 (23.88)	6	24.24 (17.07)	6	15.76 (7.01)
		142100.00 (255475.00)		1021.63 (959.79)		361.59 (145.69)		160.85 (106.86)		88.86 (153.58)		52.80 (66.95)		24.70 (11.79)		19.76 (18.58)		17.42 (13.95)

**Non-Latin America**	34	120073.24	41	502.37	41	81.13	41	41.92	40	42.61	40	34.50	40	41.44	40	76.97	40	57.71
		(247829.01)		(810.08)		(125.43)		(70.23)		(60.22)		(34.62)		(59.60)		(89.66)		(53.45)
		49672.30		236.49		21.48		11.13		14.48		21.85		22.64		41.07		47.87
		(85905.00)		(436.25)		(66.78)		(40.71)		(34.35)		(36.62)		(45.49)		(111.28)		(72.90)
P value[a]		0.278		**0.011**		**0.0004**		**0.001**		**0.011**		0.052		0.525		0.320		0.100
**One-month case fatality rate**																		
**Latin America**	3	2.11	6	5.36	6	4.27	6	4.83	6	4.98	6	4.17	6	3.70	6	3.48	6	3.29
		(1.57)		(3.10)		(3.75)		(4.18)		(3.04)		(2.99)		(2.72)		(2.16)		(2.13)
		2.39		5.30		2.77		3.46		4.18		3.13		2.67		2.69		2.66
		(3.09)		(4.28)		(2.87)		(1.31)		(5.03)		(1.75)		(0.47)		(1.18)		(0.75)
**Non-Latin America**	34	3.56	41	8.86	41	7.19	41	4.19	40	1.60	40	1.56	40	2.03	40	1.27	40	1.40
		(4.22)		(15.22)		(6.14)		(5.07)		(1.58)		(2.32)		(3.41)		(1.64)		(1.19)
		2.20		5.78		5.30		2.82		1.30		0.92		0.79		0.77		1.24
		(4.79)		(6.49)		(7.87)		(3.14)		(1.31)		(1.15)		(1.57)		(1.04)		(1.43)
P value[a]		0.889		0.714		0.315		0.364		**0.002**		**0.003**		**0.008**		**0.003**		**0.006**
**GDP loss**																		
**Latin America**	3	-1.67	6	-19.25	6	-19.25	6	-19.25	6	-9.72	6	-9.72	6	-9.72	6	-3.02	6	-3.02
		(0.86)		(6.43)		(6.43)		(6.43)		(2.87)		(2.87)		(2.87)		(2.85)		(2.85)
		-1.50		-18.30		-18.30		-18.30		-10.10		-10.10		-10.10		-3.40		-3.40
		(1.70)		(3.60)		(3.60)		(3.60)		(3.90)		(3.90)		(3.90)		(1.10)		(1.10)
**Non-Latin America**	34	-2.51	41	-12.54	41	-12.54	41	-12.54	40	-5.41	40	-5.41	40	-5.41	40	-4.47	40	-4.47
		(4.78)		(6.38)		(6.38)		(6.38)		(4.03)		(4.03)		(4.03)		(3.14)		(3.14)
		-2.50		-11.30		-11.30		-11.30		-5.30		-5.30		-5.30		-3.85		-3.85

		(2.80)		(8.30)		(8.30)		(8.30)		(2.90)		(2.90)		(2.90)		(3.55)		(3.55)
P value[b]		0.404[a]		**0.020**		**0.020**		**0.020**		**0.016**		**0.016**		**0.016**		0.290		0.290
**Unemployment rate**																		
**Latin America**	3	0.07	6	2.81	6	4.18	6	5.17	6	5.20	6	4.53	6	4.57	6	4.22	6	3.77
		(0.81)		(3.37)		(3.96)		(4.09)		(3.96)		(3.16)		(3.44)		(3.18)		(2.70)
		-0.30		1.80		3.25		3.70		3.80		3.95		3.90		3.60		3.35
		(1.50)		(1.32)		(5.74)		(8.09)		(7.50)		(4.00)		(3.60)		(2.80)		(1.90)
**Non-Latin America**	34	0.17	41	1.59	41	1.79	41	1.47	40	1.48	40	1.41	40	1.32	40	1.17	40	1.09
		(1.23)		(3.91)		(3.63)		(2.70)		(1.43)		(1.24)		(1.23)		(1.16)		(1.11)
		0.05		0.60		0.90		1.10		1.10		1.20		1.10		1.05		0.95
		(0.79)		(1.60)		(1.60)		(1.57)		(1.15)		(1.05)		(1.05)		(1.10)		(1.10)
P value[a]		0.636		0.101		0.067		**0.007**		**0.003**		**0.003**		0.069[b]		**0.003**		**0.003**
**Insomnia**																		
**Latin America**	3	1.00	6	3.00	6	2.33	6	1.67	6	0.83	6	0.67	6	0.17	6	0.67	6	-0.17
		(1.00)		(1.10)		(1.37)		(1.63)		(1.33)		(1.63)		(0.98)		(0.82)		(0.41)
		1.00		3.00		2.50		1.50		0.00		0.00		0.00		0.50		0.00
		(2.00)		(1.00)		(1.00)		(3.00)		(2.00)		(0.00)		(0.00)		(1.00)		(0.00)
**Non-Latin America**	27	0.26	34	0.91	34	0.74	34	0.32	33	0.36	33	0.39	33	0.39	33	0.18	33	0.15
		(1.16)		(1.24)		(1.19)		(0.77)		(0.82)		(1.00)		(1.14)		(1.04)		(0.76)
		0.00		0.50		0.00		0.00		0.00		0.00		0.00		0.00		0.00
		(1.00)		(2.00)		(2.00)		(1.00)		(1.00)		(0.00)		(1.00)		(0.00)		(0.00)
P value[a]		0.240		**0.002**		**0.009**		**0.027**		0.519		0.979		0.500		0.130		0.238

[a]The Mann-Whitney U test is used to compare the differences between Latin American and non-Latin American countries.

[b]Unless otherwise noted, the two-sample t test is used to compare the differences between Latin American and non-Latin American countries.

Supplementary Information 11. Two-sample t-test and Mann-Whitney U test of government actions and COVID-19 containment effectiveness for Asian and non-Asian nations and territories

Both lockdown efficiency and health-system policies are indicators of government actions against COVID-19. For the early stages of the pandemic (from March 2020 to May 2020), Asian nations and territories performed better than non-Asian ones in lockdown efficiency (Supplementary Table S11). In particular, the difference in lockdown efficiency in April between Asian and non-Asian nations and territories reached statistical significance ($P = 0.007$). In October 2020, Asian nations and territories also showed better performance of lockdown efficiency than non-Asian nations and territories ($P = 0.027$).

Another indicator of government action is health-system policies. Health-system policies include public information campaigns, testing policy, contact-tracing, and mask-wearing policy. Asian nations and territories performed better than their non-Asian peers in health system policies throughout the nine-month period (Supplementary Table S11). Particularly in both March 2020 and April 2020, the differences in health-system policies between Asian nations and territories and non-Asian countries reached statistical significance ($P < 0.003$). From the analyses of lockdown efficiency and health-system policies, we determine that Asian governments were able to enact appropriate lockdown measures and health-system policies in the early stages of the pandemic.

Utilizing the performance score with/without Google Trends, we evaluate the difference in containment effectiveness between Asian and non-Asian countries and territories (Supplementary Table S12). It is observed that Asian countries and territories perform better than non-Asian nations in performance score with/without Google Trends. From March 2020 to November 2020, in addition to the performance score without Google Trends in July 2020, both the differences of the performance score with/without Google Trends between Asian and non-Asian countries and territories reached statistical significance ($P < 0.039$).

Supplementary Table S11. **Comparison of government actions between Asian and non-Asian nations and territories from March 2020 to November 2020**

	Mar 2020		Apr 2020		May 2020		Jun 2020		Jul 2020		Aug 2020		Sep 2020		Oct 2020		Nov 2020	
	N	Mean (SD)<br>Median (IQR)	N	Mean (SD)<br>Median (IQR)	N	Mean (SD)<br>Median (IQR)	N	Mean (SD)<br>Median (IQR)	N	Mean (SD)<br>Median (IQR)	N	Mean (SD)<br>Median (IQR)	N	Mean (SD)<br>Median (IQR)	N	Mean (SD)<br>Median (IQR)	N	Mean (SD)<br>Median (IQR)
**Lockdown efficiency**																		
Asia	13	2.85 (1.14)<br>3.00 (2.00)	15	2.93 (1.16)<br>3.00 (2.00)	15	3.07 (0.88)<br>3.00 (2.00)	15	3.07 (0.96)<br>3.00 (2.00)	14	2.71 (1.20)<br>3.00 (2.00)	14	2.79 (1.12)<br>3.00 (2.00)	14	2.93 (0.92)<br>3.00 (2.00)	14	3.21 (0.97)<br>4.00 (2.00)	14	3.14 (1.03)<br>4.00 (2.00)
Non-Asia	24	2.33 (1.09)<br>3.00 (2.00)	32	1.94 (0.95)<br>2.00 (2.00)	32	2.63 (1.18)<br>3.00 (3.00)	32	3.09 (1.00)<br>3.00 (1.00)	32	3.13 (0.94)<br>3.00 (1.00)	32	2.56 (1.13)<br>3.00 (2.00)	32	2.50 (1.19)<br>3.00 (2.50)	32	2.44 (1.08)<br>3.00 (2.00)	32	2.50 (1.08)<br>3.00 (2.00)
P value[a]		0.180		**0.007**		0.264		0.865		0.307		0.545		0.272		**0.027**		0.065
**Health system policies**																		
Asia	13	2.73 (0.42)<br>2.67 (0.53)	15	3.08 (0.50)<br>3.20 (0.80)	15	3.20 (0.44)<br>3.20 (0.53)	15	3.32 (0.35)<br>3.47 (0.27)	14	3.35 (0.33)<br>3.47 (0.27)	14	3.43 (0.31)<br>3.47 (0.27)	14	3.41 (0.30)<br>3.47 (0.00)	14	3.49 (0.32)<br>3.47 (0.27)	14	3.52 (0.41)<br>3.73 (0.27)
Non-Asia	24	2.13	32	2.59	32	2.98	32	3.11	32	3.21	32	3.32	32	3.32	32	3.33	32	3.36

	(0.34)	(0.51)	(0.50)	(0.40)	(0.34)	(0.36)	(0.38)	(0.39)	(0.36)
	2.13	2.40	2.93	3.20	3.20	3.20	3.20	3.33	3.47
	(0.40)	(0.80)	(0.53)	(0.27)	(0.27)	(0.40)	(0.27)	(0.27)	(0.40)
P value[a]	**0.0003**	**0.003**	0.175	0.054	0.147	0.201	0.282	0.099	0.104

[a]*P* value is obtained through Mann–Whitney U test

Supplementary Table S12. **Comparison of the containment effectiveness between Asian and non-Asian nations and territories from March 2020 to November 2020**

	Mar 2020		Apr 2020		May 2020		Jun 2020		Jul 2020		Aug 2020		Sep 2020		Oct 2020		Nov 2020	
	N	Mean (SD)<br>Median (IQR)	N	Mean (SD)<br>Median (IQR)	N	Mean (SD)<br>Median (IQR)	N	Mean (SD)<br>Median (IQR)	N	Mean (SD)<br>Median (IQR)	N	Mean (SD)<br>Median (IQR)	N	Mean (SD)<br>Median (IQR)	N	Mean (SD)<br>Median (IQR)	N	Mean (SD)<br>Median (IQR)
**Performance score without Google Trends**																		
Asia	13	69.36 (10.06)<br>71.19 (13.33)	15	72.17 (12.52)<br>72.86 (23.33)	15	73.81 (12.25)<br>72.14 (18.57)	15	73.30 (15.74)<br>76.67 (27.62)	14	72.43 (14.83)<br>71.79 (24.05)	14	72.19 (15.41)<br>70.36 (21.43)	14	75.70 (13.29)<br>75.71 (20.48)	14	79.54 (14.18)<br>84.17 (14.29)	14	80.19 (12.13)<br>82.50 (16.90)
Non-Asia	24	56.88 (10.86)<br>55.83 (15.12)	32	57.81 (9.62)<br>58.10 (9.88)	32	61.18 (11.29)<br>60.12 (13.57)	32	64.23 (12.52)<br>65.00 (17.50)	32	65.48 (14.34)<br>65.00 (24.88)	32	63.07 (11.69)<br>64.64 (18.21)	32	62.07 (10.72)<br>61.43 (12.98)	32	60.34 (10.50)<br>58.81 (10.71)	32	60.54 (11.72)<br>58.33 (12.98)
P value[a]		**0.002**		**<.0001**		**0.001**		**0.039**		0.141		**0.033**		**0.001**		**0.0002**[b]		**<.0001**[b]
**Performance score with Google Trends**																		
Asia	11	69.80 (9.64)<br>68.61 (17.13)	13	72.07 (8.31)<br>72.78 (10.56)	13	73.75 (9.47)<br>69.35 (15.37)	13	75.53 (11.90)<br>78.24 (15.93)	12	75.76 (10.69)<br>73.80 (20.19)	12	74.85 (11.93)<br>75.28 (18.38)	12	77.33 (9.77)<br>75.97 (14.26)	12	81.22 (8.28)<br>82.59 (6.94)	12	80.99 (8.24)<br>82.50 (10.09)
Non-Asia	19	61.92	27	59.85	27	62.44	27	65.31	27	66.79	27	64.93	27	64.98	27	63.80	27	64.88

	(8.03)	(8.22)	(8.47)	(9.68)	(11.57)	(9.68)	(9.40)	(9.05)	(9.17)
	63.61	58.89	62.87	66.48	64.44	65.83	65.19	62.41	62.22
	(15.37)	(9.91)	(15.37)	(13.80)	(15.93)	(16.67)	(13.89)	(14.72)	(10.37)
P value[a]	**0.023**	**<.0001**	**0.001**	**0.006**	**0.028**	**0.009**	**0.001**	**<.0001**	**0.0001[b]**

[a]Unless otherwise noted, the two-sample t test is used to compare the differences between Asia and Non-Asia nations and territories

[b]The Mann-Whitney U test is used to compare the differences between Asia and Non-Asia nations and territories

Supplementary Information 12. Complete ranking and raw scores of 50 countries and territories, from March 2020 to November 2021

Supplementary Tables S13–S33 show the raw scores of all the indicators during the period of March 2020 to November 2021. These tables illustrate the performance scores with/without Google Trends data and the rankings of 50 countries. It is evident that most Asian nations and territories — such as Taiwan, and Korea — have higher scores than those in Europe or the Americas before November 2020. However, when the vaccination indicator was included in our containment performance calculation, the situation has changed, and the performance of countries in the Americas or Europe has improved.

Supplementary Table S13. **Complete ranking and raw scores of indicators in 50 countries and territories in March 2020**

			Concern indicators									
			Government policy		Vital health and socioeconomic measures						Hygiene education	
Country	Rank	Performance score (with Google Trends)[a]	Lockdown efficiency	Health-system policies	One-month cases per 100,000 members of the population	Infection growth rate (%)	Fatality rate (%)	GDP loss (%)	Unemployment rate (%)	Insomnia[b]	Health literacy[c]	Performance score (without Google Trends)
Taiwan	1	77.40	4	2.93	1.19	725.64	1.41	0.67	0.04	0	59.2	81.70
Thailand	2	75.30	4	2.13	2.31	3830.95	0.62	-4.9	0	1	82.5	76.81
Japan	3	71.39	4	2.4	1.59	820.41	3.03	-2.1	0	0	43.1	77.45
Qatar	4	70.34	3	2.13	27.07	78000	0.26	18.1	0	0	18.4	81.44
Russia	5	69.84	2	2.4	1.6	116750	0.73	1	0	0	58.9	72.04
Egypt	6	66.64	4	1.87	0.69	70900	6.49	-0.3	-0.4	0	30.9	73.98
Vietnam	7	66.60	4	3.2	0.2	1225	0	-3.14	0.05	3	33.4	81.95
Chile	8	66.13	4	1.6	14.87	142100	0.42	-0.9	1	1	47.4	72.61
New Zealand	9	65.84	2	2.4	13.4	64600	0.15	-3	0	0	42.8	70.38
Italy	10	65.77	3	2.67	173.11	9278.72	11.85	-6.2	-2.9	-1	76.6	60.13
France	11	65.52	3	2.67	77.23	52178	6.75	-7.6	-0.9	-1	50	65.55
Mexico	12	64.83	4	1.33	0.94	30275	2.39	-2.6	-0.3	2	45.9	74.12
Finland	13	64.75	1	2.13	25.54	47166.67	1.2	-1.5	0.3	-1	49.9	64.58
Australia	14	64.27	1	2.4	17.78	18136	0.4	-0.1	0.1	0	44.5	67.98
Brazil	15	63.06	2	1.6	2.69	285750	3.52	-1.5	-0.5	0	50.9	65.05

Canada	16	62.46	1	2.4	22.54	42535	1.79	-2.1	2	0	61.6	61.97
India	17	61.77	2	2.4	0.1	46466.67	2.51	-2.7	2.1	0	38.4	66.10
Hong Kong	18	61.46	4	2.93	8.26	651.58	0.32	-9.8	1.4	2	23.3	74.67
Netherlands	19	61.45	3	2.13	73.89	211016.67	8.21	-1.8	-0.4	-2	25.4	62.78
South Korea	20	61.22	1	3.2	12.94	210.67	2.2	-0.3	0	2	42.5	70.21
Malaysia	21	60.17	3	2.93	8.47	10964	1.57	-3.8	0.5	2	16.4	74.50
United Kingdom	22	59.58	1	2.13	57.09	63531.15	6.34	-3.9	0.2	-1	51	57.69
Denmark	23	59.13	3	2.13	52.42	101200	2.96	-2.7	0.4	0	17.2	67.28
Sweden	24	58.47	1	2.4	47.73	34428.57	7.99	-1.7	-0.6	1	55.7	60.97
Germany	25	57.63	1	2.4	85.61	90796.2	1.08	-3.3	0.6	0	43.9	59.58
United States	26	57.03	1	2.13	58.09	769104	2.79	-2	0.6	-1	60.6	52.34
Singapore	27	56.80	1	2.67	14.08	807.84	0.36	-1.1	0.2	2	22.8	68.79
Austria	28	54.07	3	2.13	112.93	113011.11	1.26	-5.8	4.8	0	46	54.55
Spain	29	51.63	3	2.13	205.07	213062.22	8.83	-6.5	-0.29	1	61.9	50.83
Argentina	NA[d]	NA[d]	2	2.4	2.33	NA[d]	2.56	0.7	0.3	1	38.5	NA[d]
Belgium	NA[d]	NA[d]	3	2.13	110.22	1277400	5.52	-3.9	-0.6	NA[d]	NA[d]	49.62
China	NA[d]	NA[d]	2	3.47	0.16	2.91	20.4	-13.2	0.7	NA[d]	NA[d]	55.71
Colombia	NA[d]	NA[d]	4	2.13	1.78	NA[d]	1.77	-2.9	1.8	1	58.5	NA[d]
Czech Republic	Nad	NA[d]	3	2.4	30.89	NA[d]	0.94	-4.2	0	0	37	NA[d]
Greece	NA[d]	NA[d]	3	1.6	12.57	32750	3.74	-2.3	-2.3	2	NA[d]	73.58
Hungary	NA[d]	NA[d]	4	2.4	5.09	NA[d]	3.25	-2.9	0.1	0	61.3	NA[d]
Indonesia	NA[d]	NA[d]	4	2.13	0.56	NA[d]	8.9	-2.1	-0.07	0	59.7	NA[d]
Ireland	NA[d]	NA[d]	1	2.4	65.49	323400	2.2	1.6	0.1	NA[d]	NA[d]	60.76
Israel	NA[d]	NA[d]	3	2.4	65.98	95183.33	0.37	-2.7	-0.2	2	NA[d]	70.30
Norway	NA[d]	NA[d]	3	1.87	85.33	30840	0.84	-0.4	-0.3	0	NA[d]	68.68
Pakistan	NA[d]	NA[d]	2	2.13	0.96	52850	1.28	NA[d]	NA[d]	NA[d]	NA[d]	NA[d]
Peru	NA[d]	NA[d]	2	2.13	3.23	NA[d]	2.82	-6.2	-0.4	2	43.7	NA[d]
Philippines	NA[d]	NA[d]	2	2.67	1.9	69366.67	4.18	-6.6	0.1	NA[d]	18.9	67.44

Poland	NA[d]	NA[d]	4	1.6	6.11	NA[d]	1.43	-3.5	-0.5	0	61.5	NA[d]
Portugal	NA[d]	NA[d]	3	2.4	72.99	NA[d]	2.15	-4.7	-0.1	1	36.3	NA[d]
Saudi Arabia	NA[d]	NA[d]	2	2.67	4.49	NA[d]	0.64	-2.7	0	0	42.2	NA[d]
South Africa	NA[d]	NA[d]	4	2.4	2.28	NA[d]	0.37	0.4	2.5	1	44.4	NA[d]
Switzerland	NA[d]	NA[d]	3	2.13	191.65	92150	2.61	-1.1	0.5	0	NA[d]	58.47
Turkey	NA[d]	NA[d]	4	2.4	16.04	NA[d]	1.58	7.1	-1.1	0	40	NA[d]
United Arab Emirates	NA[d]	NA[d]	4	2.4	6.5	3061.9	0.93	NA[d]	NA[d]	1	42.6	NA[d]

Notes:

a. Google Trends include indicators as insomnia and health literacy.

b. Increase in Google searches for “insomnia”.

c. Google Trends number for “wash hands” and “face mask”.

d. NA: not applicable. Indicates a country not ranked due to incomplete data.

Supplementary Table S14. **Complete ranking and raw scores of indicators in 50 countries and territories in April 2020**

			Concern indicators									
			Government policy		Vital health and socioeconomic measures						Hygiene education	
Country	Rank	Performance score (with Google Trends)[a]	Lockdown efficiency	Health-system policies	One-month cases per 100,000 members of the population	Infection growth rate (%)	Fatality rate (%)	GDP loss (%)	Unemployment rate (%)	Insomnia[b]	Health literacy[c]	Performance score (without Google Trends)
Japan	1	84.86	4	2.4	9.51	533.44	3.44	-10.5	0.2	0	78.63	83.85
Taiwan	2	84.81	4	2.93	0.45	33.23	0.93	-2.25	0.37	1	50.88	91.95
South Korea	3	80.97	4	3.2	1.93	10.1	8.7	-4.7	-0.2	0	15.25	90.98
Indonesia	4	80.87	4	3.2	3.14	562.17	7.64	-10.37	1.79	1	59.5	85.23
Australia	5	77.52	4	2.4	8.65	48.41	3.4	-7.9	1.2	0	17.75	86.06
Hong Kong	6	73.94	4	2.93	4.31	45.24	0	-9.4	2.4	2	18.25	87.08
Poland	7	73.65	2	2.93	27.92	457.2	5.78	-13.7	0.2	0	42.63	76.33
Hungary	8	71.89	4	2.4	23.63	464.02	12.97	-17.9	0.2	0	19	78.58
Finland	9	71.80	1	2.13	64.56	252.26	5.42	-7.2	0.1	0	49.25	72.68
Portugal	10	71.28	3	2.67	172.62	236.49	4.71	-18.5	-0.7	0	42.25	73.35
Malaysia	11	70.09	4	3.47	10	116.99	1.82	-22	1.6	2	15.5	82.65
Argentina	12	69.79	2	3.2	7.47	320.11	5.66	-19.4	2.5	1	43.25	74.11
New Zealand	13	69.43	2	2.67	17.25	128.59	2.16	-13.4	0	1	22.5	77.61
Saudi Arabia	14	69.34	2	2.67	60.87	1355.73	0.72	-7.5	3.4	0	30.38	73.13
Italy	15	69.10	3	3.73	164.85	94.21	15.59	-18.7	-2.7	3	50.88	77.47
South Africa	16	67.91	2	2.93	7.24	317.37	2.28	-18.7	-5.7	4	47.88	79.37
Czech Republic	17	67.82	1	3.47	40.84	132.22	4.69	-13.2	0.7	0	8.38	75.38
Vietnam	18	67.30	2	3.47	0.06	27.36	0	-6.34	0.57	4	15.63	84.76
Germany	19	66.63	1	2.67	108.85	127.01	6.41	-11.4	0.9	1	39.13	70.83
Austria	20	66.49	1	3.2	58.54	51.79	8.65	-15	5.5	0	30.25	69.49
France	21	65.97	3	2.4	172.55	223.01	17.86	-20.7	-1.3	2	54.88	69.82
Netherlands	22	65.97	1	2.13	156.67	211.93	14.05	-11	0.1	-1	26.5	66.68
Egypt	23	65.63	2	2.4	4.72	679.86	7.17	-7.1	2.1	2	18.88	76.28

Russia	24	64.47	2	2.93	71.38	4457.04	1.01	-8.9	1.1	0	42.5	64.55
Turkey	25	64.43	1	2.4	126.48	788.36	2.77	-8.6	0.5	1	32.13	69.34
Chile	26	63.19	1	3.2	77.72	522.43	1.45	-15.8	1.9	3	48.25	70.37
Denmark	27	63.02	1	2.13	109.06	207.86	5.73	-11.3	1.7	0	13.38	68.26
Colombia	28	62.99	2	3.73	11.01	618.21	4.95	-18.9	9.5	3	45.25	70.69
Qatar	29	62.84	3	2.4	438.31	1616.9	0.06	-4.7	0	0	22.38	66.30
Singapore	30	62.80	1	3.73	260.55	1646.11	0.08	-13.5	0.6	0	29.5	64.89
Sweden	31	61.36	1	2.13	166.02	346.86	13.13	-8.9	1.4	1	27.63	66.26
Canada	32	61.12	1	2.4	121.69	538.64	8.77	-14.7	7.3	1	48.38	61.97
Peru	33	59.74	3	3.2	108.91	3371.92	2.84	-31.3	1.7	3	81	59.67
Mexico	34	59.73	2	2.13	13.97	1482.22	10.16	-17.7	1.18	4	55.5	67.39
India	35	59.68	2	4	2.43	2395.56	3.34	-29.8	16.1	0	50.88	56.78
Brazil	36	59.58	2	2.4	38.33	1425.05	7.13	-12.4	0.1	4	33	71.50
United Kingdom	37	57.35	1	2.13	206.16	360.57	17.36	-22.9	0.2	0	27.88	58.19
Spain	38	56.98	3	2.13	251.34	122.51	13.68	-23.7	1.31	3	39.63	64.04
United States	39	55.44	1	2.4	268.49	462.15	6.85	-11	11.2	2	51.25	56.97
Belgium	NA[d]	NA[d]	3	2.13	308.41	279.8	19.27	-15.7	-0.6	NA[d]	NA[d]	66.04
China	NA[d]	NA[d]	4	3.47	0.09	1.66	98.08	-3	1	NA[d]	NA[d]	79.06
Greece	NA[d]	NA[d]	2	2.13	12.25	97.18	7.13	-16.2	-1.6	1	NA[d]	74.89
Ireland	NA[d]	NA[d]	3	2.13	351.92	537.16	6.68	-8.8	-0.4	NA[d]	NA[d]	68.90
Israel	NA[d]	NA[d]	3	3.2	117.6	178.05	2	-11.5	-0.23	1	NA[d]	80.75
Norway	NA[d]	NA[d]	1	2.13	57.13	66.73	5.52	-2.8	1.2	0	NA[d]	74.94
Pakistan	NA[d]	NA[d]	2	2.4	7.24	755.24	2.44	NA[d]	NA[d]	NA[d]	NA[d]	NA[d]
Philippines	NA[d]	NA[d]	2	2.67	5.84	307.29	7.5	-22.6	12.6	NA[d]	6.38	63.85
Switzerland	NA[d]	NA[d]	1	2.13	149.99	78.18	10.05	-7.9	1	0	NA[d]	68.84
Thailand	NA[d]	NA[d]	4	3.2	1.87	78.92	3.38	-14.5	NA[d]	2	25.88	NA[d]
United Arab Emirates	NA[d]	NA[d]	3	4	119.48	1779.67	0.84	NA[d]	NA[d]	1	30.63	NA[d]

Notes:

a. Google Trends include indicators as insomnia and health literacy.

b. Increase in Google searches for “insomnia”.
c. Google Trends number for “wash hands” and “face mask”.
d. NA: not applicable. Indicates a country not ranked due to incomplete data.

Supplementary Table S15. **Complete ranking and raw scores of indicators in 50 countries and territories in May 2020**

Country	Rank	Performance score (with Google Trends)[a]	Concern indicators: Government policy: Lockdown efficiency	Concern indicators: Government policy: Health-system policies	Concern indicators: Vital health and socioeconomic measures: One-month cases per 100,000 members of the population	Concern indicators: Vital health and socioeconomic measures: Infection growth rate (%)	Concern indicators: Vital health and socioeconomic measures: Fatality rate (%)	Concern indicators: Vital health and socioeconomic measures: GDP loss (%)	Concern indicators: Vital health and socioeconomic measures: Unemployment rate (%)	Concern indicators: Vital health and socioeconomic measures: Insomnia[b]	Concern indicators: Hygiene education: Health literacy[c]	Performance score (without Google Trends)
South Korea	1	85.17	4	3.47	1.42	6.77	3.16	-4.7	0.3	0	23.13	90.29
Taiwan	2	82.36	4	2.93	0.05	3.03	7.69	-2.25	0.4	0	24.13	86.39
Vietnam	3	80.46	4	3.47	0.06	21.48	0	-6.34	0.57	1	11.25	91.20
Germany	4	78.54	4	3.2	24.35	12.52	9.4	-11.4	1.1	0	26.88	80.69
Czech Republic	5	76.78	4	3.47	14.81	20.65	5.3	-13.2	1	0	8.63	83.64
Singapore	6	75.96	3	3.73	319.9	115.75	0.04	-13.5	0.6	0	18.75	79.70
Austria	7	75.58	4	3.2	14.2	8.28	6.57	-15	4.7	0	20.88	78.60
Russia	8	75.44	3	3.73	205.12	281.08	1.21	-8.9	1.6	0	23.88	77.57
Saudi Arabia	9	74.95	3	2.93	179.55	274.72	0.55	-7.5	3.4	0	30.63	75.00
Hong Kong	10	74.61	4	2.93	0.63	4.53	0	-9.4	3.1	2	14.25	86.39
Australia	11	74.11	4	2.93	1.71	6.44	2.29	-7.9	1.9	2	12.38	86.29
New Zealand	12	73.93	4	2.67	0.52	1.69	12	-13.4	0	0	18.25	77.23
Portugal	13	73.16	1	3.2	73.11	29.77	5.65	-18.5	-0.7	1	54.25	69.53
France	14	72.68	3	3.2	32.73	13.1	20.15	-20.7	-1.3	0	50.13	66.51
Poland	15	72.25	2	2.93	28.82	84.72	3.85	-13.7	0.6	0	22.13	73.97
Egypt	16	71.48	2	2.93	19	351.24	2.92	-7.1	2.1	0	30.63	70.55
Japan	17	70.51	4	2.4	1.95	17.27	16.9	-10.5	0.5	2	41	73.48
Malaysia	18	70.50	2	3.47	5.61	30.27	0.72	-22	2	0	12.13	74.58
Indonesia	19	70.28	4	3.2	5.98	161.64	5.02	-10.37	1.79	3	28.13	80.43
Finland	20	69.05	1	2.13	33.64	37.32	5.85	-7.2	1.8	0	23.13	69.57
South Africa	21	68.29	3	3.73	45.59	478.77	2.15	-18.7	-5.7	3	41.75	73.98
Turkey	22	67.84	3	2.67	51.86	36.39	3.12	-8.6	0.7	3	16.88	80.51
Italy	23	66.68	1	3.73	45.54	13.4	19.79	-18.7	-1.6	0	36.88	62.58
Denmark	24	66.63	1	2.67	43.39	26.86	4.85	-11.3	1.9	0	9.75	70.28

Sweden	25	66.04	1	2.13	168.92	78.98	10.6	-8.9	1.9	0	32.5	63.02
Canada	26	66.01	1	3.2	100.74	69.82	9.8	-14.7	8.3	0	39.63	60.94
Hungary	27	65.99	4	2.93	11.4	39.68	19.44	-17.9	0.7	0	10.75	69.17
Colombia	28	65.83	3	3.73	44.96	351.56	2.82	-18.9	10.91	2	50.25	64.82
Argentina	29	65.78	2	3.2	27.49	280.56	2.58	-19.4	2.5	0	17.25	67.03
Netherlands	30	65.19	1	2.13	41.63	18.05	16.32	-11	0.3	0	33.25	61.71
Qatar	31	60.95	3	3.47	1509.9	324.42	0.06	-4.7	0	2	19.75	67.26
Brazil	32	60.95	3	3.73	201.2	490.51	5.45	-12.4	0.6	3	27.63	68.58
United Kingdom	33	59.98	1	2.4	116.03	44.06	13.68	-22.9	0.3	0	25	57.37
United States	34	59.66	1	2.67	216.83	66.39	5.8	-11	9.7	2	33.38	61.70
Spain	35	59.37	3	2.93	55.7	12.2	9.92	-23.7	1.31	4	30.5	69.30
Peru	36	58.22	3	3.2	386.69	344.82	2.71	-31.3	6.4	3	55.88	57.00
India	37	57.49	2	4	11.29	446.74	2.73	-29.8	14.7	0	31.5	52.31
Chile	38	56.56	3	3.2	553.71	597.94	0.78	-15.8	4	2	25.25	60.04
Mexico	39	55.61	3	3.2	55.41	371.62	11.3	-17.7	0.66	4	31.75	64.11
Belgium	NA[d]	NA[d]	3	2.93	85.09	20.33	18.99	-15.7	-0.6	NA[d]	NA[d]	67.67
China	NA[d]	NA[d]	2	3.47	0.01	0.17	0.7	-3	0.9	NA[d]	NA[d]	85.48
Greece	NA[d]	NA[d]	4	2.67	3.13	12.58	10.74	-16.2	-0.1	0	NA[d]	76.53
Ireland	NA[d]	NA[d]	3	2.13	88.66	21.24	9.59	-8.8	-0.4	NA[d]	NA[d]	74.68
Israel	NA[d]	NA[d]	2	3.2	13.67	7.44	5.24	-11.5	0.58	0	NA[d]	77.04
Norway	NA[d]	NA[d]	4	2.13	12.95	9.07	3.7	-2.8	1.2	0	NA[d]	85.26
Pakistan	NA[d]	NA[d]	2	2.67	24.6	300.02	2.07	NA[d]	NA[d]	NA[d]	NA[d]	NA[d]
Philippines	NA[d]	NA[d]	2	2.67	8.76	113.08	4.05	-22.6	12.6	NA[d]	5.88	59.63
Switzerland	NA[d]	NA[d]	4	2.93	14.74	4.31	14.34	-7.9	1.2	0	NA[d]	78.16
Thailand	NA[d]	NA[d]	2	3.47	0.18	4.3	2.36	-14.5	NA[d]	0	13	NA[d]
United Arab Emirates	NA[d]	NA[d]	1	4	223.21	176.88	0.72	NA[d]	NA[d]	0	22.63	NA[d]

Notes:

a. Google Trends include indicators as insomnia and health literacy.

b. Increase in Google searches for “insomnia”.

c. Google Trends number for “wash hands” and “face mask”.

d. NA: not applicable. Indicates a country not ranked due to incomplete data.

Supplementary Table S16. **Complete ranking and raw scores of indicators in 50 countries in June 2020**

Country	Rank	Performance score (with Google Trends)[a]	Concern indicators									Performance score (without Google Trends)
			Government policy		Vital health and socioeconomic measures						Hygiene education	
			Lockdown efficiency	Health-system policies	One-month cases per 100,000 members of the population	Infection growth rate (%)	Fatality rate (%)	GDP loss (%)	Unemployment rate (%)	Insomnia[b]	Health literacy[c]	
Taiwan	1	86.45	4	3.2	0.02	1.13	0	-2.25	0.23	0	23	92.49
South Korea	2	85.06	4	3.47	2.63	11.71	0.82	-4.7	0.2	0	21.4	91.28
Turkey	3	81.03	3	3.73	42.64	21.94	1.64	-8.6	0.3	-1	15.7	85.26
Vietnam	4	80.25	4	3.47	0.03	8.23	0	-6.34	0.57	0	5.1	90.90
Japan	5	80.25	4	2.67	1.47	11.13	3.97	-10.5	0.5	1	34.6	83.24
Australia	6	78.76	4	2.93	2.82	9.97	0.14	-7.9	2.2	0	11.4	86.74
Malaysia	7	78.38	4	3.47	2.53	10.49	0.73	-22	1.6	0	23.9	81.79
Denmark	8	77.74	4	2.67	18.97	9.26	2.82	-11.3	1.8	0	19.6	82.50
Russia	9	77.18	3	3.73	165.2	59.4	1.91	-8.9	1.8	0	21.8	80.99
New Zealand	10	76.57	4	2.67	0.5	1.6	0	-13.4	0	0	7.6	85.27
Czech Republic	11	74.49	4	3.2	25.08	28.98	1.08	-13.2	1.1	0	2.7	84.34
Saudi Arabia	12	74.45	3	3.73	303.22	123.81	1.09	-7.5	3.4	1	31.2	77.00
Canada	13	74.31	3	3.2	36.08	14.73	6.42	-14.7	6.8	-1	27.5	72.42
Finland	14	74.10	4	2.13	6.41	5.18	2.25	-7.2	1.7	0	4.1	83.34
Hong Kong	15	73.93	4	2.93	1.61	11.16	2.48	-9.4	3.4	1	11.2	83.45
Portugal	16	73.59	3	3.2	94.55	29.66	1.72	-18.5	-0.7	0	16.2	78.38
Austria	17	73.53	4	3.2	11.49	6.19	3.57	-15	3.6	0	9	80.86
France	18	73.20	4	3.2	19.64	6.95	7.85	-20.7	-1.3	0	13.4	78.88
Indonesia	19	73.02	2	3.2	10.94	112.99	4.22	-10.37	1.79	0	29.9	72.76
Italy	20	72.76	4	3.73	12.54	3.25	17.83	-18.7	-0.4	0	24	74.53
Germany	21	72.61	2	3.47	14.33	6.55	3.75	-11.4	1.3	0	13.5	78.08
South Africa	22	72.09	3	3.73	199.85	362.65	1.67	-18.7	-5.7	0	34.7	69.86
Egypt	23	71.35	3	3.2	42.34	173.41	4.6	-7.1	2.1	1	26.4	74.73
United States	24	71.20	3	2.67	254.82	46.89	2.34	-11	7.4	0	28.1	71.07

Netherlands	25	70.54	1	3.2	22.4	8.23	4.09	-11	0.9	1	26.4	73.68
United Kingdom	26	69.34	3	2.93	40.77	10.75	10.67	-22.9	0.2	0	24.9	69.81
Poland	27	66.81	1	2.93	28.03	44.59	3.76	-13.7	0.8	0	14.7	70.19
India	28	66.06	3	3.47	28.61	207.16	3.04	-29.8	2.3	0	28.7	64.24
Singapore	29	65.48	1	3.73	154.23	25.87	0.03	-13.5	0.6	3	19.8	75.23
Spain	30	63.75	4	3.2	20.94	4.09	12.54	-23.7	1.31	2	13.8	72.30
Colombia	31	63.65	3	3.73	134.55	233	3.5	-18.9	10.4	1	35	61.76
Qatar	32	63.43	3	3.47	1359.85	68.84	0.19	-4.7	0	1	6.1	71.78
Chile	33	63.32	3	3.2	815.24	126.14	2.97	-15.8	4.9	0	22	63.11
Sweden	34	62.77	1	2.4	289.75	75.69	3.21	-8.9	2.6	1	20.9	65.65
Brazil	35	62.46	3	3.73	417.39	172.32	3.41	-12.4	1.3	4	27.3	71.55
Argentina	36	61.97	3	3.2	105.49	282.94	1.61	-19.4	2.5	0	10.7	65.40
Peru	37	60.22	3	3.2	366.18	73.41	4.28	-31.3	10	2	42.8	57.42
Hungary	38	60.20	4	2.93	2.89	7.2	21.15	-17.9	1.3	1	3.8	68.44
Mexico	39	59.36	3	3.2	105.04	149.37	13.17	-17.7	1.91	3	28.8	64.17
Belgium	NA[d]	NA[d]	1	3.2	26.28	5.22	9.19	-15.7	0	NA[d]	NA[d]	68.70
China	NA[d]	NA[d]	2	3.47	0.04	0.62	0	-3	0.6	NA[d]	NA[d]	85.68
Greece	NA[d]	NA[d]	4	3.2	4.72	16.87	3.46	-16.2	0.7	1	NA[d]	82.26
Ireland	NA[d]	NA[d]	2	2.4	9.78	1.93	17.39	-8.8	0.1	NA[d]	NA[d]	67.49
Israel	NA[d]	NA[d]	3	3.2	96.57	48.94	0.48	-11.5	0.46	0	NA[d]	80.97
Norway	NA[d]	NA[d]	4	2.67	8.1	5.2	3.19	-2.8	1.2	0	NA[d]	87.17
Pakistan	NA[d]	NA[d]	3	3.2	63.84	194.6	2.02	NA[d]	NA[d]	NA[d]	NA[d]	NA[d]
Philippines	NA[d]	NA[d]	2	2.67	17.73	107.42	1.59	-22.6	12.6	NA[d]	9.2	58.34
Switzerland	NA[d]	NA[d]	4	3.2	9.84	2.76	5.05	-7.9	1.1	1	NA[d]	85.46
Thailand	NA[d]	NA[d]	4	3.47	0.13	2.92	1.11	-14.5	NA[d]	0	14.1	NA[d]
United Arab Emirates	NA[d]	NA[d]	3	3.73	142.66	40.83	0.36	NA[d]	NA[d]	2	25.3	NA[d]

Notes:

a. Google Trends include indicators as insomnia and health literacy.

b. Increase in Google searches for “insomnia”.

c. Google Trends number for “wash hands” and “face mask”.

d. NA: not applicable. Indicates a country not ranked due to incomplete data.

***Supplementary Table S17*. Complete ranking and raw scores of indicators in 50 countries and territories in July 2020**

			Concern indicators									
			Government policy		Vital health and socioeconomic measures						Hygiene education	
Country	Rank	Performance score (with Google Trends)[a]	Lockdown efficiency	Health-system policies	One-month cases per 100,000 members of the population	Infection growth rate (%)	Fatality rate (%)	GDP loss (%)	Unemployment rate (%)	Insomnia[b]	Health literacy[c]	Performance score (without Google Trends)
Malaysia	1	86.32	4	3.47	1.04	3.9	1.19	-7.1	1.4	0	49	85.99
Netherlands	2	81.75	4	3.2	23.97	8.14	0.83	-4.2	1.1	0	27.5	86.92
Taiwan	3	81.37	4	3.2	0.08	4.47	0	1.23	0.18	1	16.38	93.52
South Korea	4	80.41	4	3.47	2.9	11.56	1.28	-3	0.1	0	13.75	89.55
France	5	79.97	4	3.2	34.03	11.26	1.84	-5.5	0.7	-1	16	84.69
New Zealand	6	78.73	4	2.67	0.71	2.23	0	-2.3	1.1	0	10.63	88.38
Thailand	7	77.77	4	3.47	0.2	4.38	0	-9.1	1	0	10.88	87.05
Germany	8	77.69	4	3.47	17.88	7.67	1.05	-4.5	1.4	0	10.25	87.15
Finland	9	76.58	4	2.4	3.93	3.02	0.46	-4.3	1.7	0	13.88	84.58
Italy	10	76.07	4	3.73	11.51	2.89	5.37	-5.7	0.2	0	17.13	82.90
Denmark	11	74.61	4	2.93	18.3	8.17	0.94	-5.2	1.5	1	16.5	84.79
Poland	12	74.12	4	2.93	29.84	32.84	2.24	-6.3	0.9	0	14.88	81.10
Turkey	13	74.00	1	3.73	36.72	15.49	1.81	5.3	0.5	0	10	82.49
United Kingdom	14	73.77	2	3.2	28.84	6.86	4.06	-9.9	0.5	0	41.63	72.18
Japan	15	73.18	4	2.67	13.93	94.65	0.2	-6.7	0.7	1	26.75	79.71
Czech Republic	16	72.78	4	3.2	43.14	38.65	0.71	-7.2	1.1	0	3.88	82.86
Canada	17	71.91	2	3.2	32.28	11.48	2.01	-7	5.2	0	39.38	70.50
Russia	18	71.40	3	3.47	131.25	29.61	2.42	-5	1.8	0	18.13	76.57
Spain	19	70.53	3	3.2	83.95	15.75	0.23	-10.4	2.34	0	13.63	76.88
Hungary	20	70.31	4	2.93	3.62	8.42	3.14	-9.4	1.4	0	4.88	79.37
Vietnam	21	70.21	2	3.47	0.21	57.18	1.48	-4.69	0.33	0	9	77.94
Portugal	22	69.58	3	3.2	87.59	21.19	1.78	-7.6	1.7	0	10.38	76.68
Australia	23	69.29	2	3.2	36.71	118.18	1.04	-5.6	2.3	0	31.13	69.74

Egypt	24	69.16	3	3.2	25.18	37.72	7.19	-4.9	-0.5	0	18.5	73.58
Sweden	25	67.89	3	2.4	90.62	13.47	4.48	-4.3	2	1	30.13	71.84
Saudi Arabia	26	67.87	3	3.73	244.39	44.59	1.43	-4.1	3	0	11	74.29
Austria	27	67.22	1	3.2	37.35	18.94	0.39	-5.4	2.7	0	12.38	73.02
India	28	67.12	3	3.47	80.47	189.67	1.72	-12	0.1	1	43.5	66.61
Hong Kong	29	63.54	2	2.93	27.57	171.54	0.97	-0.8	3.2	0	14.88	67.49
Singapore	30	61.53	1	3.73	141.84	18.9	0.01	-6.5	1.3	3	19.63	74.13
Brazil	31	59.82	3	3.73	592.98	89.9	2.61	-5.2	2	0	17.5	61.88
Argentina	32	56.05	3	3.2	280.5	196.45	1.76	-8.3	2	0	7.75	60.12
South Africa	33	55.07	3	3.73	576.6	226.16	1.56	-6.3	1.7	1	31.5	54.92
Chile	34	54.74	3	3.2	399	27.3	4.94	-12.4	5.6	0	15.5	55.99
United States	35	52.84	3	3.47	581.64	72.87	1.38	-4.9	6.5	3	33.88	58.44
Mexico	36	51.89	3	3.2	153.99	87.82	9.53	-8.5	1.7	3	29.25	58.69
Colombia	37	48.65	3	3.73	388.46	202.01	3.43	-11.7	9.5	0	26.63	44.64
Peru	38	46.61	3	3.47	370.86	42.87	7.64	-12.2	10.4	2	35.25	46.43
Belgium	NA[d]	NA[d]	1	3.2	63.19	11.92	1.28	-6.2	0.7	NA[d]	NA[d]	73.64
China	NA[d]	NA[d]	2	3.47	0.06	0.96	0	-1.1	0.4	NA[d]	NA[d]	85.84
Greece	NA[d]	NA[d]	4	3.47	10.25	31.33	1.31	-11.6	-0.5	1	NA[d]	83.56
Indonesia	NA[d]	NA[d]	2	3.2	19.01	92.21	4.34	-8.51	NA[d]	0	26.88	NA[d]
Ireland	NA[d]	NA[d]	4	3.2	11.99	2.32	4.56	1.8	1.1	NA[d]	NA[d]	85.68
Israel	NA[d]	NA[d]	1	3.2	534.87	182	0.51	-4.4	1	0	NA[d]	53.36
Norway	NA[d]	NA[d]	4	2.67	6.66	4.07	1.39	-1	1.6	0	NA[d]	86.18
Pakistan	NA[d]	NA[d]	3	3.2	29.35	30.37	2.4	NA[d]	NA[d]	NA[d]	NA[d]	NA[d]
Philippines	NA[d]	NA[d]	3	2.93	50.96	148.85	1.36	-17.9	4.6	NA[d]	4.25	58.99
Qatar	NA[d]	NA[d]	3	3.47	507	15.2	0.42	-4.3	NA[d]	1	4.38	NA[d]
Switzerland	NA[d]	NA[d]	1	3.47	40.65	11.09	0.51	-2.6	1.1	0	NA[d]	78.05
United Arab Emirates	NA[d]	NA[d]	1	3.73	119.7	24.33	0.3	NA[d]	NA[d]	0	6.25	NA[d]

Notes:

a. Google Trends include indicators as insomnia and health literacy.

b. Increase in Google searches for “insomnia”.

c. Google Trends number for “wash hands” and “face mask”.
d. NA: not applicable. Indicates a country not ranked due to incomplete data.

Supplementary Table S18. **Complete ranking and raw scores of indicators in 50 countries and territories in August 2020**

Country	Rank	Performance score (with Google Trends)[a]	Concern indicators										Performance score (without Google Trends)
			Government policy		Vital health and socioeconomic measures							Hygiene education	
			Lockdown efficiency	Health-system policies	One-month cases per 100,000 members of the population	Infection growth rate (%)	Fatality rate (%)	GDP loss (%)	Unemployment rate (%)	Insomnia[b]		Health literacy[c]	
Taiwan	1	89.30	4	3.47	0.09	4.5	0	1.23	0.1	0		24.5	93.76
Turkey	2	86.24	4	3.73	46.55	17.01	1.73	5.3	-1	0		13.63	94.47
Italy	3	82.16	4	3.73	35.85	8.76	1.58	-5.7	0.4	0		18.63	87.09
New Zealand	4	81.13	2	2.93	3.94	12.16	0	-2.3	1.1	0		31.38	80.31
Finland	5	80.55	4	2.67	11.8	8.8	1.07	-4.3	1.6	0		21.88	83.63
South Korea	6	80.44	4	3.47	11.4	40.78	0.39	-3	0.2	0		14.75	86.54
Malaysia	7	79.62	2	3.47	1.12	4.06	0.55	-7.1	1.4	0		29.5	79.18
Germany	8	79.30	4	3.47	41.06	16.35	0.45	-4.5	1.5	0		13	85.82
Thailand	9	78.70	4	3.47	0.15	3.08	0	-9.1	0.9	0		9.63	86.49
Canada	10	75.97	2	3.2	33.48	10.68	1.33	-7	4.5	0		35.38	71.96
Poland	11	75.60	4	3.2	57.29	47.46	1.49	-6.3	0.9	0		16	79.77
Denmark	12	75.56	1	2.93	58.39	24.11	0.27	-5.2	1.2	0		33.63	72.19
United Kingdom	13	74.17	2	3.2	49.04	10.92	0.95	-9.9	0.6	0		21.88	75.43
France	14	74.13	1	4	139.01	41.33	0.4	-5.5	0.7	-1		20.88	72.94
South Africa	15	74.00	3	3.73	225.7	27.14	4.59	-6.3	1.7	0		28.5	72.37
Hungary	16	73.09	4	2.93	16.91	36.27	1.16	-9.4	1.2	0		10.63	78.85
Saudi Arabia	17	72.84	3	3.73	114.51	14.45	2.59	-4.1	3	0		12.38	77.78
Singapore	18	72.79	1	3.73	78.75	8.82	0	-6.5	1.3	0		17.25	75.63
Austria	19	72.46	4	3.2	70.04	29.85	0.24	-5.4	2.3	1		9.25	81.49
Japan	20	71.93	4	2.67	25.43	88.76	0.9	-6.7	0.8	1		23.5	74.71
Sweden	21	71.65	1	2.4	72.31	9.48	1.07	-4.3	1.9	1		33	70.29
Portugal	22	71.24	3	3.2	68.06	13.59	1.25	-7.6	1.7	0		7.13	77.97
Russia	23	70.20	1	3.47	105.49	18.36	2.07	-5	2.1	0		21	70.70
Australia	24	69.50	2	4	33.49	49.42	5.34	-5.6	1.6	0		18.38	70.91

Egypt	25	68.36	2	3.2	4.75	5.17	12.67	-4.9	-0.5	0	21.25	68.22
Netherlands	26	67.69	1	3.2	108.8	34.15	0.46	-4.2	1.1	0	11.25	71.64
Hong Kong	27	64.54	2	3.73	20.51	47	4.03	-0.8	3.2	2	12.5	72.78
Brazil	28	64.02	3	3.73	586.09	46.79	2.32	-5.2	2.6	0	15.13	65.27
Spain	29	63.18	3	3.2	372.87	60.42	0.37	-10.4	2.34	0	12.13	65.47
India	30	62.94	3	3.47	144.58	117.64	1.44	-12	0.2	0	11.25	65.54
Chile	31	62.55	3	3.2	293.25	15.76	3.27	-12.4	5.3	0	15.25	63.32
Czech Republic	32	62.46	1	3.2	75.11	48.53	0.52	-7.2	1.1	0	2	68.87
United States	33	60.72	3	3.73	440.93	31.95	2.03	-4.9	4.7	4	26.88	67.42
Mexico	34	57.24	3	3.2	135.67	41.19	10.13	-8.5	1.5	4	25.75	63.43
Argentina	35	55.47	3	3.2	501	118.36	2.26	-8.3	2	0	10	56.46
Vietnam	36	54.90	2	2.93	0.5	87.1	6.38	-4.69	0.33	3	8.75	64.84
Colombia	37	53.53	3	3.73	628.08	108.15	2.99	-11.7	6	0	22.25	48.73
Peru	38	52.91	3	3.73	726.91	58.82	4.08	-12.2	9.8	0	28.13	45.41
Belgium	NA[d]	NA[d]	1	3.47	142.24	23.98	0.33	-6.2	1.3	NA[d]	NA[d]	71.67
China	NA[d]	NA[d]	2	3.47	0.05	0.85	0	-1.1	0.4	NA[d]	NA[d]	85.17
Greece	NA[d]	NA[d]	4	3.47	56.03	130.44	1.03	-11.6	0.4	1	NA[d]	69.94
Indonesia	NA[d]	NA[d]	2	3.2	24.28	61.29	3.44	-8.51	NA[d]	0	22.5	NA[d]
Ireland	NA[d]	NA[d]	2	3.2	55.61	10.54	0.51	1.8	1.6	NA[d]	NA[d]	81.72
Israel	NA[d]	NA[d]	1	3.2	521.89	62.97	0.86	-4.4	1.19	1	NA[d]	59.79
Norway	NA[d]	NA[d]	4	2.93	28.44	16.69	0.58	-1	1.6	1	NA[d]	85.98
Pakistan	NA[d]	NA[d]	4	3.47	7.94	6.3	1.96	NA[d]	NA[d]	NA[d]	NA[d]	NA[d]
Philippines	NA[d]	NA[d]	3	3.47	116.32	136.54	1.2	-17.9	4.6	NA[d]	9.38	54.92
Qatar	NA[d]	NA[d]	3	3.47	280.56	7.3	0.28	-4.3	NA[d]	0	24	NA[d]
Switzerland	NA[d]	NA[d]	1	3.47	80.25	19.71	0.36	-2.6	1.2	1	NA[d]	75.65
United Arab Emirates	NA[d]	NA[d]	1	3.73	98.33	16.07	0.34	NA[d]	NA[d]	2	26.38	NA[d]

Notes:

a. Google Trends include indicators as insomnia and health literacy.

b. Increase in Google searches for "insomnia".

c. Google Trends number for “wash hands” and “face mask”.
d. NA: not applicable. Indicates a country not ranked due to incomplete data.

Supplementary Table S19. **Complete ranking and raw scores of indicators in 50 countries and territories in September 2020**

Country	Rank	Performance score (with Google Trends)[a]	Concern indicators									Performance score (without Google Trends)
			Government policy		Vital health and socioeconomic measures						Hygiene education	
			Lockdown efficiency	Health-system policies	One-month cases per 100,000 members of the population	Infection growth rate (%)	Fatality rate (%)	GDP loss (%)	Unemployment rate (%)	Insomnia[b]	Health literacy[c]	
Taiwan	1	85.74	4	3.47	0.11	5.33	0	1.23	0.04	0	18.6	93.90
Italy	2	83.59	4	3.73	75.5	16.96	0.9	-5.7	0.2	0	24.6	88.44
Singapore	3	81.41	4	3.73	16.29	1.68	0	-6.5	1.3	0	18.1	88.56
New Zealand	4	80.08	4	3.2	1.99	5.48	3.13	-2.3	1.1	0	17.8	86.99
South Africa	5	79.56	4	3.73	79.75	7.54	5.47	-6.3	1.7	0	25.1	83.03
Finland	6	79.51	4	2.67	34.4	23.57	0.42	-4.3	1.7	-2	13.3	84.20
South Korea	7	79.47	4	3.47	7.23	18.37	2.45	-3	0.6	0	13.7	88.06
Germany	8	79.21	4	3.47	57.42	19.65	0.4	-4.5	1.4	0	14.7	87.26
Turkey	9	78.71	2	3.73	57.54	17.97	3.76	5.3	-1.2	0	12.5	87.62
Japan	10	77.35	4	2.67	12.01	22.22	1.82	-6.7	0.6	0	18.4	83.21
Poland	11	77.33	4	3.2	63.79	35.83	1.96	-6.3	1	0	17.3	83.68
Canada	12	75.65	2	3.2	79.99	23.06	0.66	-7	3.5	0	32.8	74.53
United Kingdom	13	75.32	3	3.47	173.47	34.83	0.55	-9.9	1	0	22	78.97
Russia	14	74.57	1	3.47	122.24	17.98	1.96	-5	1.8	-1	26.4	73.98
Brazil	15	74.34	3	3.73	424.66	23.1	2.5	-5.2	2.8	0	23.1	77.22
Malaysia	16	74.32	2	3.47	5.82	20.17	0.48	-7.1	1.3	0	19	79.04
Thailand	17	74.31	4	3.47	0.22	4.6	0.64	-9.1	0.79	1	7.7	86.16
Mexico	18	72.44	3	3.2	111.42	23.96	9.21	-8.5	1.3	2	36.7	72.73
India	19	72.31	3	3.47	189.96	71.02	1.27	-12	-0.5	1	21.9	77.19
Netherlands	20	71.76	1	3.2	307.89	72.04	0.39	-4.2	0.9	0	27.9	71.74
Portugal	21	71.73	3	3.2	171.92	30.22	0.85	-7.6	1.7	1	17.2	78.56
Saudi Arabia	22	70.33	2	3.73	54.1	5.96	4.62	-4.1	3	0	12.9	76.66
France	23	68.70	1	4	421.43	88.66	0.47	-5.5	0.7	0	18.2	72.18
Vietnam	24	68.01	2	2.93	0.05	4.79	2	-4.69	0.33	1	5	79.28

Sweden	25	67.94	2	2.4	84.01	10.05	0.85	-4.3	2.3	2	18.5	75.15
Denmark	26	67.85	1	3.2	191.1	63.58	0.23	-5.2	1.1	0	15.1	72.48
Austria	27	67.46	1	3.47	192.92	63.32	0.38	-5.4	1.7	0	14	72.48
Spain	28	66.20	3	3.2	655.18	66.18	0.88	-10.4	2.34	0	16.1	69.91
Hong Kong	29	65.88	2	3.47	3.69	5.76	5.78	-0.8	3.5	2	9.1	76.74
United States	30	65.65	3	3.73	365.11	20.05	1.94	-4.9	4.3	5	22	76.74
Chile	31	65.52	3	3.2	268.18	12.45	2.83	-12.4	5	0	14.1	69.93
Egypt	32	64.98	2	3.2	4.16	4.3	11.95	-4.9	-0.5	2	14.3	73.24
Argentina	33	63.98	3	3.2	737.38	79.78	2.48	-8.3	2	-1	7.3	68.98
Peru	34	63.80	3	3.73	499.22	25.43	2.19	-12.2	10.7	0	24.9	62.86
Australia	35	62.79	2	4	5.01	4.95	18.09	-5.6	1.7	0	10.3	68.14
Colombia	36	62.76	3	2.4	421.72	34.89	2.95	-11.7	5.6	0	18.8	64.27
Czech Republic	37	58.32	1	3.47	430.9	187.44	0.5	-7.2	1.1	0	5.9	64.38
Hungary	38	54.56	1	2.93	210.37	331.03	0.74	-9.4	0.9	1	16	57.03
Belgium	NA[d]	NA[d]	1	3.47	286.6	38.97	0.36	-6.2	1.2	NA[d]	NA[d]	72.77
China	NA[d]	NA[d]	2	3.47	0.02	0.42	0	-1.1	0.2	NA[d]	NA[d]	85.34
Greece	NA[d]	NA[d]	4	3.47	78.27	79.07	1.53	-11.6	-0.4	1	NA[d]	81.39
Indonesia	NA[d]	NA[d]	2	3.2	41.02	64.2	2.96	-8.51	NA[d]	0	29	NA[d]
Ireland	NA[d]	NA[d]	1	3.2	148.73	25.49	0.37	1.8	2	NA[d]	NA[d]	77.65
Israel	NA[d]	NA[d]	3	3.2	1507.76	111.63	0.5	-4.4	1	0	NA[d]	65.47
Norway	NA[d]	NA[d]	4	2.93	59.86	30.1	0.31	-1	1.6	0	NA[d]	86.84
Pakistan	NA[d]	NA[d]	4	3.47	7.68	5.73	1.12	NA[d]	NA[d]	NA[d]	NA[d]	NA[d]
Philippines	NA[d]	NA[d]	3	3.47	82.93	41.15	2.14	-17.9	4.6	NA[d]	6.2	69.05
Qatar	NA[d]	NA[d]	3	3.47	242.34	5.88	0.24	-4.3	NA[d]	1	25.6	NA[d]
Switzerland	NA[d]	NA[d]	1	3.47	128.31	26.33	0.61	-2.6	1.1	0	NA[d]	76.95
United Arab Emirates	NA[d]	NA[d]	1	3.73	242.25	34.11	0.15	NA[d]	NA[d]	0	16.6	NA[d]

Notes:

a. Google Trends include indicators as insomnia and health literacy.

b. Increase in Google searches for "insomnia".

c. Google Trends number for "wash hands" and "face mask".

d. NA: not applicable. Indicates a country not ranked due to incomplete data.

Supplementary Table S20. **Complete ranking and raw scores of indicators in 50 countries and territories in October 2020**

Country	Rank	Performance score (with Google Trends)[a]	Concern indicators									Performance score (without Google Trends)
			Government policy		Vital health and socioeconomic measures						Hygiene education	
			Lockdown efficiency	Health-syste m policies	One-month cases per 100,000 members of the population	Infection growth rate (%)	Fatality rate (%)	GDP loss (%)	Unemployment rate (%)	Insomnia[b]	Health literacy[c]	
Taiwan	1	85.79	4	3.47	0.17	7.98	0	1.86	0.05	0	14.63	96.22
Singapore	2	83.16	4	3.73	4.27	0.43	0.4	-3.4	1	-1	12.88	91.01
India	3	82.61	2	3.73	135.62	29.65	1.25	-2.8	-1.1	0	41.75	83.69
New Zealand	4	80.78	4	2.93	2.3	6.01	0	-2.6	0.8	-1	7.5	89.62
Japan	5	80.57	4	2.67	14.02	21.22	1.09	0.3	0.7	0	17.5	88.62
South Korea	6	79.07	4	3.73	5.36	11.49	1.86	-3.4	0.7	0	12.38	88.28
Vietnam	7	78.90	4	2.93	0.09	7.86	0	-2.31	0.22	0	4.38	90.55
Canada	8	78.34	4	3.2	201.91	47.3	1.16	-4.8	3.4	-1	26.38	80.60
Finland	9	78.10	4	2.67	110.47	61.26	0.23	-2.2	1.2	0	17.63	85.40
Thailand	10	77.90	4	3.47	0.31	6.02	0	-5.5	1.19	0	6.5	88.60
Italy	11	76.89	3	3.73	602.97	115.79	0.75	-6.4	0.5	-2	23.38	77.29
Hong Kong	12	76.40	4	3.47	3.15	4.64	0	0.2	3.3	2	8.75	90.75
Saudi Arabia	13	74.81	4	3.73	36.41	3.79	5	-3.6	1.7	0	17.63	81.17
Denmark	14	74.42	1	3.2	317.39	64.55	0.39	-3.8	0.9	0	38.75	74.09
Turkey	15	73.52	2	3.73	67.23	17.79	3.63	-0.5	-0.5	0	11.88	81.30
Netherlands	16	73.51	3	3.2	1351.93	183.87	0.43	-4.4	0.8	0	45.88	70.70
South Africa	17	73.25	4	3.73	86.18	7.58	4.97	-3.7	3.4	0	19.63	78.54
Portugal	18	72.93	3	3.2	644.69	87.02	0.82	-8.3	0.4	0	30.75	74.68
Chile	19	72.67	3	3.2	247.25	10.21	3.1	2	4.5	0	15.88	78.97
Germany	20	72.60	2	3.47	285.11	81.55	0.41	-3.7	1.4	0	20.13	77.55
Australia	21	72.38	2	4	1.96	1.84	3.81	-3.3	1.7	-1	11.13	77.70
Brazil	22	70.58	3	3.73	340.93	15.06	2.2	-2.7	2.7	1	11	80.17
Russia	23	69.92	1	3.47	298.4	37.19	1.64	-3.9	1.7	0	24.38	72.79
Sweden	24	69.09	1	2.4	311.82	33.91	0.14	-2.9	1.8	1	29.5	72.50

Malaysia	25	68.24	2	3.73	62.79	181.08	0.56	-7	1.5	1	25.5	72.66
United Kingdom	26	67.47	3	3.2	823.36	122.62	0.79	-8.5	1.1	0	18.88	71.35
Austria	27	66.81	3	3.47	667.44	134.14	0.52	-5.8	1.7	1	13	74.71
Argentina	28	65.78	3	3.47	920.27	55.38	3.38	-3.2	2.1	0	7.25	72.79
Peru	29	65.05	3	3.47	268.14	10.89	2.28	-3.6	10	1	22.5	69.49
Egypt	30	64.89	2	3.2	4.26	4.22	7.71	-3.6	-0.8	0	10.88	70.52
France	31	64.56	1	4	1196.6	133.43	0.6	-5.6	-0.1	0	14.38	69.00
Colombia	32	63.09	3	2.4	480.53	29.47	2.17	-6.8	4.9	0	11.88	67.89
Spain	33	62.77	3	3.47	890.8	54.15	0.98	-10.6	2.35	2	16.38	70.84
Mexico	34	61.12	2	3.2	140.96	24.45	7.76	-3.8	1.1	2	24.13	66.31
United States	35	59.53	1	3.73	583.13	26.68	1.27	-4.7	3.3	4	19.25	70.55
Poland	36	59.05	1	3.2	716.62	296.37	1.15	-6.4	1.1	0	23	59.23
Hungary	37	56.38	1	3.2	505.78	184.65	2.02	-7.8	0.8	1	10	62.23
Czech Republic	38	51.48	3	3.47	2468.39	373.56	0.98	-6.8	1.1	0	5.25	55.03
Belgium	NA[d]	NA[d]	1	3.47	2681.51	262.37	0.52	-6.5	0.8	NA[d]	NA[d]	52.49
China	NA[d]	NA[d]	2	3.47	0.04	0.68	0	0.5	0.2	NA[d]	NA[d]	88.01
Greece	NA[d]	NA[d]	2	4	199.33	112.45	1.13	-7.8	-0.3	0	NA[d]	76.11
Indonesia	NA[d]	NA[d]	4	3.2	45	42.88	2.54	-7.16	NA[d]	0	23.88	NA[d]
Ireland	NA[d]	NA[d]	3	3.2	512.39	69.98	0.43	-4.4	1.5	NA[d]	NA[d]	78.63
Israel	NA[d]	NA[d]	3	3.47	773.35	27.06	1.43	-4.4	1.1	1	NA[d]	78.52
Norway	NA[d]	NA[d]	4	2.93	116.28	44.94	0.13	-2.3	1	0	NA[d]	87.28
Pakistan	NA[d]	NA[d]	4	3.47	9.58	6.77	1.6	NA[d]	NA[d]	NA[d]	NA[d]	NA[d]
Philippines	NA[d]	NA[d]	2	3.47	63	22.15	2.49	-14.9	4.2	NA[d]	22.13	64.11
Qatar	NA[d]	NA[d]	3	3.47	235.89	5.4	0.26	-3.3	NA[d]	0	0	NA[d]
Switzerland	NA[d]	NA[d]	1	3.47	1166.65	189.5	0.22	-3.2	1	1	NA[d]	66.44
United Arab Emirates	NA[d]	NA[d]	1	3.73	388.65	40.81	0.2	NA[d]	NA[d]	0	22.5	NA[d]

Notes:

a. Google Trends include indicators as insomnia and health literacy.

b. Increase in Google searches for "insomnia".

c. Google Trends number for “wash hands” and “face mask”.
d. NA: not applicable. Indicates a country not ranked due to incomplete data.

***Supplementary Table S21.* Complete ranking and raw scores of indicators in 50 countries and territories in November 2020**

			Concern indicators									
			Government policy		Vital health and socioeconomic measures						Hygiene education	
Country	Rank	Performance score (with Google Trends)[a]	Lockdown efficiency	Health-system policies	One-month cases per 100,000 members of the population	Infection growth rate (%)	Fatality rate (%)	GDP loss (%)	Unemployment rate (%)	Insomnia[b]	Health literacy[c]	Performance score (without Google Trends)
Taiwan	1	82.84	4	3.47	0.5	21.62	0	1.86	0.04	0	16.5	95.12
Australia	2	82.69	4	3.73	1.24	1.15	0.32	-3.3	1.7	-2	8.13	90.16
India	3	76.82	2	3.73	92.66	15.62	1.21	-2.8	-0.7	0	30	83.56
New Zealand	4	76.57	4	2.93	2.07	5.1	0	-2.6	0.8	0	7.75	89.53
South Korea	5	76.47	4	3.73	15.64	30.1	0.75	-3.4	0.6	0	10.63	88.59
Japan	6	76.44	4	2.67	37.66	47.01	0.64	0.3	0.8	0	18.25	86.40
Vietnam	7	76.19	4	2.93	0.17	14.15	0	-2.31	0.22	0	4.5	89.97
Egypt	8	75.93	4	3.47	8.17	7.77	4.6	-3.6	-0.8	0	24.38	84.01
Singapore	9	75.44	4	3.73	3.47	0.35	0.49	-3.4	1	1	10.63	90.84
Turkey	10	71.43	2	3.73	312.41	70.19	1.33	-0.5	-0.3	-1	9.13	78.96
Saudi Arabia	11	71.13	4	3.73	28.95	2.9	4.9	-3.6	1.7	0	13.63	80.87
Thailand	12	70.43	4	2.93	0.32	5.92	0.45	-5.5	0.9	1	4.88	86.03
Hong Kong	13	70.40	4	3.47	13.22	18.62	0.4	0.2	3.1	2	8.5	88.54
South Africa	14	70.17	4	3.73	108.84	8.9	3.5	-3.7	3.4	0	12.75	79.89
Chile	15	66.40	2	3.2	217.03	8.13	2.9	2	3.8	0	11.13	75.50
Germany	16	66.24	3	2.93	642.27	101.19	1.15	-3.7	1.4	-1	17	70.07
Brazil	17	64.81	1	3.73	376.49	14.46	1.65	-2.7	2.9	0	16.5	71.94
Finland	18	64.70	4	2.67	158.81	54.61	0.47	-2.2	1	2	2.63	82.87
Argentina	19	64.63	3	3.47	569.98	22.08	3	-3.2	2.1	0	7	74.40
Russia	20	63.91	1	3.47	458.88	41.69	1.75	-3.9	1.5	0	22	69.23
Sweden	21	63.38	1	2.4	1176.07	95.51	0.63	-2.9	0.9	0	52	60.05
Peru	22	62.67	2	3.47	189.1	6.93	2.43	-3.6	8.8	-1	17	65.48
Malaysia	23	62.67	2	3.73	105.51	108.24	0.33	-7	1.6	0	9.63	71.13
Canada	24	62.19	1	3.2	382.18	60.78	1.36	-4.8	2.6	0	26.63	65.71
France	25	61.44	3	4	1276.58	60.98	1.85	-5.6	-0.1	1	14.63	71.71

Colombia	26	61.29	3	2.93	476.82	22.59	2.25	-6.8	4	0	12.13	68.65
United Kingdom	27	60.59	3	3.2	911.74	60.99	1.92	-8.5	1.2	0	15	66.94
Denmark	28	60.58	1	3.2	589.4	72.85	0.34	-3.8	0.8	0	8.5	68.77
Mexico	29	60.48	2	3.2	146.26	20.39	7.52	-3.8	1	0	19.38	65.56
Spain	30	59.15	3	3.47	989.22	39.01	1.99	-10.6	2.35	0	13.88	65.40
Netherlands	31	59.03	1	3.2	1017.17	48.73	1.14	-4.4	0.5	0	11.38	65.96
Italy	32	58.93	3	3.73	1525.13	135.72	1.84	-6.4	0.1	0	23.75	62.33
United States	33	56.97	1	3.73	1360.93	49.15	0.87	-4.7	3.2	0	17.38	61.61
Portugal	34	56.42	3	3.47	1537.57	110.97	1.27	-8.3	0.4	0	12.75	62.22
Austria	35	51.49	3	3.73	1971.16	169.2	1.17	-5.8	2.2	0	15	55.25
Czech Republic	36	51.17	3	3.47	1757.37	56.16	2.68	-6.8	1.2	1	1.5	62.21
Hungary	37	49.20	3	3.2	1467.87	188.26	2.17	-7.8	0.9	0	9.5	53.85
Poland	38	49.18	3	3.2	1659.54	173.15	1.83	-6.4	1	1	16.88	55.31
Belgium	NA[d]	NA[d]	3	3.47	1278.01	34.51	3.39	-6.5	0.6	NA[d]	NA[d]	67.09
China	NA[d]	NA[d]	2	4	0.04	0.63	0	0.5	0.1	NA[d]	NA[d]	90.23
Greece	NA[d]	NA[d]	3	4	633.4	168.2	2.7	-7.8	-0.5	0	NA[d]	65.28
Indonesia	NA[d]	NA[d]	4	3.2	47.09	31.41	2.39	-7.16	NA[d]	0	22.13	NA[d]
Ireland	NA[d]	NA[d]	3	3.2	224.55	18.04	1.26	-4.4	1.3	NA[d]	NA[d]	79.69
Israel	NA[d]	NA[d]	2	4	263.9	7.27	1.36	-4.4	0.8	1	NA[d]	80.07
Norway	NA[d]	NA[d]	1	3.2	291.8	77.81	0.32	-2.3	1	0	NA[d]	71.53
Pakistan	NA[d]	NA[d]	4	3.73	30.11	19.92	1.91	NA[d]	NA[d]	NA[d]	NA[d]	NA[d]
Philippines	NA[d]	NA[d]	2	3.47	46.45	13.37	2.3	-14.9	4.2	NA[d]	28.75	63.55
Qatar	NA[d]	NA[d]	2	3.47	217.87	4.74	0.08	-3.3	NA[d]	1	8	NA[d]
Switzerland	NA[d]	NA[d]	1	3.47	1996.86	112.04	1.46	-3.2	1	0	NA[d]	54.77
United Arab Emirates	NA[d]	NA[d]	1	4	366.32	27.32	0.21	NA[d]	NA[d]	1	7.5	NA[d]

Notes:
a. Google Trends include indicators as insomnia and health literacy.
b. Increase in Google searches for “insomnia”.
c. Google Trends number for “wash hands” and “face mask”.
d. NA: not applicable. Indicates a country not ranked due to incomplete data.

Supplementary Table S22. **Complete ranking and raw scores of indicators in 50 countries and territories in December 2020**

Country	Rank	Performance score (with Google Trends)[a]	Concern indicators										
			Government policy			Vital health and socioeconomic measures						Hygiene education	Performance score (without Google Trends)
			Lockdown efficiency	Health-system policies	People covered by vaccines	One-month cases per 100,000 members of the population	Infection growth rate (%)	Fatality rate (%)	GDP loss (%)	Unemployment rate (%)	Insomnia[b]	Health literacy[c]	
Taiwan	1	78.50	4	2.67	0	0.52	18.37	0	1.86	0.04	-1	16.4	80.84
Finland	2	64.73	4	2.1	0.02	202.05	44.94	1.45	-2.2	1.8	-1	2	68.41
Chile	3	61.14	2	2.67	0.02	299.38	10.37	2.09	2	3.2	0	11.1	65.07
Argentina	4	59.15	3	2.67	0.04	444.69	14.11	2.25	-3.2	2.1	0	5.1	64.57
Germany	5	58.22	3	2.29	0.12	824.27	64.55	2.48	-3.7	0.7	0	16	59.79
Sweden	6	57.77	3	1.9	0.01	1923.41	79.9	1.05	-2.9	2.2	0	39.6	51.38
Poland	7	57.18	3	2.48	0.06	803.42	30.69	3.75	-6.4	1	0	19	57.49
Russia	8	56.64	1	2.67	0.02	583.42	37.41	1.97	-3.9	1.3	0	17.5	57.31
Canada	9	56.05	3	2.67	0.13	542.42	53.49	1.72	-4.8	3.2	1	18.9	60.28
United Kingdom	10	55.91	3	3.05	0.74	1270.51	52.79	1.75	-8.5	1.3	0	12.4	58.09
Austria	11	55.32	3	2.86	0.03	870.04	27.74	3.88	-5.8	2.5	0	15.3	56.40
Portugal	12	54.51	3	2.67	0.16	1133.87	38.79	2.08	-8.3	0.4	0	7.1	58.10
France	13	54.49	1	2.86	0	593.2	17.6	2.98	-5.6	-0.1	0	7.9	57.82
Mexico	14	54.39	2	2.48	0.01	242.41	28.07	6.36	-3.8	0.9	0	14.3	55.56
Italy	15	52.22	3	2.86	0.03	836.25	31.57	3.68	-6.4	0.2	2	19	59.63
Denmark	16	49.24	1	2.48	0.29	1434.93	102.61	0.55	-3.8	0.7	0	6	51.89
Hungary	17	45.23	3	2.29	0.01	1090.97	48.54	4.47	-7.8	0.8	1	3	52.04
Czech Republic	18	45.22	3	3.05	0.06	1824.29	37.33	1.68	-6.8	1.1	2	5.1	55.50
United States	19	42.39	1	3.24	0.42	1942.45	47.03	1.26	-4.7	3.2	2	15.1	48.64
Australia	NA[d]	NA[d]	2	2.48	NA[d]	2.01	1.84	0.19	-3.3	1.5	-1	7.6	NA[d]
Belgium	NA[d]	NA[d]	1	2.67	0	596.66	11.98	4.17	-6.5	0.8	NA[d]	NA[d]	53.48
Brazil	NA[d]	NA[d]	1	2.86	NA[d]	630.46	21.15	1.63	-2.7	2.9	1	14.9	NA[d]

China	NA[d]	NA[d]	4	2.48	0.16	0.04	0.61	0	0.5	0	NA[d]	NA[d]	80.55
Colombia	NA[d]	NA[d]	1	2.29	NA[d]	640.63	24.75	1.98	-6.8	3.9	1	9.5	NA[d]
Egypt	NA[d]	NA[d]	2	2.67	NA[d]	21.65	19.11	4.43	-3.6	-0.8	0	19.4	NA[d]
Greece	NA[d]	NA[d]	3	2.86	0.01	322.16	31.9	7.24	-7.8	-0.7	0	NA[d]	56.99
Hong Kong	NA[d]	NA[d]	2	2.67	NA[d]	33.77	40.1	1.54	0.2	3.3	0	8.5	NA[d]
India	NA[d]	NA[d]	2	2.86	NA[d]	59.7	8.71	1.38	-2.8	1.5	0	14.9	NA[d]
Indonesia	NA[d]	NA[d]	4	2.48	NA[d]	74.7	37.91	2.54	-7.16	NA[d]	1	24.4	NA[d]
Ireland	NA[d]	NA[d]	2	2.48	0.02	389.55	26.51	0.96	-4.4	1.5	NA[d]	NA[d]	62.73
Israel	NA[d]	NA[d]	3	2.86	5.72	994.4	25.53	0.53	-4.4	1.1	0	NA[d]	76.98
Japan	NA[d]	NA[d]	4	2.1	NA[d]	68.67	58.3	1.4	0.3	0.8	0	16.7	NA[d]
Malaysia	NA[d]	NA[d]	2	2.86	NA[d]	146.18	72.02	0.23	-7	1.5	0	15.9	NA[d]
Netherlands	NA[d]	NA[d]	3	2.29	NA[d]	1613.39	51.97	0.75	-4.4	0.7	0	12.5	NA[d]
New Zealand	NA[d]	NA[d]	4	2.48	NA[d]	2.14	5	0	-2.6	0.8	-1	6	NA[d]
Norway	NA[d]	NA[d]	4	2.48	0.02	247.49	37.11	0.78	-2.3	1	0	NA[d]	71.94
Pakistan	NA[d]	NA[d]	2	2.86	NA[d]	36.98	20.4	2.55	NA[d]	NA[d]	NA[d]	NA[d]	NA[d]
Peru	NA[d]	NA[d]	4	2.86	NA[d]	159.55	5.47	6.88	-3.6	7.7	1	15	NA[d]
Philippines	NA[d]	NA[d]	4	2.67	NA[d]	38.72	9.83	2.01	-14.9	4.2	NA[d]	18.1	NA[d]
Qatar	NA[d]	NA[d]	4	2.67	0	173.58	3.6	0.16	-3.3	NA[d]	0	7.1	NA[d]
Saudi Arabia	NA[d]	NA[d]	4	2.86	NA[d]	15.46	1.51	6.08	-3.6	1.7	0	15	NA[d]
Singapore	NA[d]	NA[d]	4	2.86	NA[d]	6.51	0.65	0	-3.4	1	0	19.6	NA[d]
South Africa	NA[d]	NA[d]	4	2.86	NA[d]	450.45	33.82	2.6	-3.7	3.4	0	12.2	NA[d]
South Korea	NA[d]	NA[d]	2	2.48	NA[d]	52.89	78.26	1.44	-3.4	0.8	0	9.6	NA[d]
Spain	NA[d]	NA[d]	3	2.67	NA[d]	599.04	16.99	2.06	-10.6	2.35	1	15.5	NA[d]
Switzerland	NA[d]	NA[d]	1	2.67	NA[d]	1446.9	38.29	2.26	-3.2	1	1	NA[d]	52.20
Thailand	NA[d]	NA[d]	4	2.67	NA[d]	4.52	78.72	0.1	-5.5	0.88	-1	10.6	NA[d]
Turkey	NA[d]	NA[d]	1	2.86	NA[d]	885.21	245.72	0.96	-0.5	-1	2	7.9	NA[d]
United Arab Emirates	NA[d]	NA[d]	1	3.05	NA[d]	393.94	23.07	0.25	NA[d]	NA[d]	2	15.9	NA[d]

Vietnam	NA[d]	NA[d]	4	2.86	NA[d]	0.12	8.76	0	-2.31	0.22	0	5.1	NA[d]

Notes:

a. Google Trends include indicators as insomnia and health literacy.

b. Increase in Google searches for “insomnia”.

c. Google Trends number for “wash hands” and “face mask”.

d. NA: not applicable. Indicates a country not ranked due to incomplete data.

Supplementary Table S23. **Complete ranking and raw scores of indicators in 50 countries and territories in January 2021**

Country	Rank	Performance score (with Google Trends)[a]	Concern indicators										Performance score (without Google Trends)
			Government policy			Vital health and socioeconomic measures						Hygiene education	
			Lockdown efficiency	Health-system policies	People covered by vaccines	One-month cases per 100,000 members of the population	Infection growth rate (%)	Fatality rate (%)	GDP loss (%)	Unemployment rate (%)	Insomnia[b]	Health literacy[c]	
Taiwan	1	72.95	4	2.67	0	0.47	14.02	0.89	6.41	0.04	-1	18.75	71.93
Singapore	2	70.47	4	3.24	1.33	16.02	1.6	0	1.3	0.5	1	19.75	73.44
Saudi Arabia	3	66.29	4	3.43	0.63	15.32	1.47	2.85	-2	0.8	0	14.5	67.74
Russia	4	64.50	4	3.24	0.34	466.65	21.78	2.31	-2.1	1.1	1	25.13	63.90
Egypt	5	63.48	4	2.86	0	27.25	20.2	6.04	-2.1	-0.3	1	28	61.52
Italy	6	63.01	3	3.43	1.68	737.43	21.16	3.22	5	0.6	1	21.5	63.44
Indonesia	7	62.98	4	2.86	0.09	122.52	45.09	2.35	-2.23	1.32	1	25.5	61.86
Austria	8	62.63	3	3.24	1.46	594.94	14.85	2.8	-1.9	2.7	0	28.125	57.92
Argentina	9	62.52	3	2.86	0.41	667.6	18.56	1.57	7.7	-0.2	0	5.63	66.46
India	10	61.88	2	3.24	0.14	34.12	4.58	1.15	-1.4	-0.7	1	12.25	65.61
Sweden	11	61.80	3	2.48	1.67	1283.04	29.63	2.21	-0.3	1.8	0	33.625	54.75
Germany	12	60.68	3	2.48	1.51	555.16	26.42	5.02	-0.9	0.5	1	33.63	55.85
Turkey	13	59.20	2	3.05	1.18	318.73	12.17	1.9	2.5	-0.5	1	7.75	63.99
France	14	58.25	1	3.24	1.26	855.86	21.6	1.98	6.7	0.3	0	11.38	58.90
Netherlands	15	58.15	3	2.48	1.03	1072.04	22.72	1.41	-2.6	0.6	1	19.75	58.04
Finland	16	57.38	4	2.48	1.46	164.8	25.29	1.2	0.1	1.6	2	6.5	64.70
Denmark	17	55.50	3	3.05	2.29	604.95	21.35	2.36	-1.3	0.7	1	2.375	61.44
Poland	18	54.77	3	2.67	1.55	577.35	16.87	3.95	-2.9	1	2	18.25	56.90
Hungary	19	51.87	3	2.67	1.55	466.57	13.98	6.63	-4.2	0.9	0	5.13	53.34
Chile	20	51.50	3	2.86	0.18	617.99	19.4	1.56	0.1	2.8	3	10.75	58.20
Spain	21	51.23	3	3.05	1.72	1742.82	42.26	0.92	0	1.57	2	8.88	56.09
Brazil	22	50.84	1	2.86	0.49	719.21	19.92	1.93	1.3	3	1	13.63	51.28
Portugal	23	49.83	3	3.05	1.7	3009.19	74.17	1.82	-3.1	0.3	0	12.25	48.05
United	24	49.48	3	3.05	7.21	1962.04	53.36	2.46	-3.9	1.1	3	15.88	53.70

Kingdom													
Mexico	25	47.66	2	2.86	0.26	339.84	30.72	7.47	-2.3	0.9	3	21.75	49.16
Canada	26	47.19	1	3.05	1.27	520.12	33.42	2.18	0.7	3.9	3	18.25	49.92
Czech Republic	27	44.93	3	3.43	1.31	2484.95	37.03	1.78	-0.3	1.2	3	1.25	53.66
United States	28	43.31	1	3.05	4.7	1857.72	30.59	1.58	0.1	2.8	4	15.75	48.53
Australia	NA[d]	NA[d]	4	2.48	NA[d]	1.54	1.38	0	-0.5	1	-1	11.75	NA[d]
Belgium	NA[d]	NA[d]	1	2.86	1.48	549.26	9.85	2.46	1.4	1.3	NA[d]	NA[d]	55.30
China	NA[d]	NA[d]	4	2.86	0.83	0.17	2.86	0.08	25.1	0.1	NA[d]	NA[d]	82.73
Colombia	NA[d]	NA[d]	3	2.67	NA[d]	888.53	27.52	2.38	0.4	4.3	3	11	NA[d]
Greece	NA[d]	NA[d]	2	3.43	1.3	173.72	13.04	5.29	-1.8	0	0	NA[d]	58.23
Hong Kong	NA[d]	NA[d]	2	2.67	NA[d]	21.42	18.16	2.05	17	3.6	2	8.38	NA[d]
Ireland	NA[d]	NA[d]	3	2.86	2.02	2121.76	114.15	1.02	5.8	2.1	NA[d]	NA[d]	50.28
Israel	NA[d]	NA[d]	3	3.43	28.61	2543.72	52.02	0.67	-1	1	0	NA[d]	65.89
Japan	NA[d]	NA[d]	4	2.1	NA[d]	122.04	65.46	1.59	0.5	0.5	0	18.5	NA[d]
Malaysia	NA[d]	NA[d]	2	2.86	NA[d]	314.99	90.21	0.28	-1.2	1.7	0	30.75	NA[d]
New Zealand	NA[d]	NA[d]	4	2.67	NA[d]	2.94	6.57	0	2.4	0.5	0	4.63	NA[d]
Norway	NA[d]	NA[d]	2	2.86	1.22	247.16	27.03	0.96	-2.8	1.4	0	NA[d]	58.51
Pakistan	NA[d]	NA[d]	4	2.86	NA[d]	29.09	13.32	2.35	NA[d]	NA[d]	NA[d]	NA[d]	NA[d]
Peru	NA[d]	NA[d]	4	2.67	NA[d]	373.35	12.13	8.72	7.5	6.7	0	21.75	NA[d]
Philippines	NA[d]	NA[d]	4	2.67	NA[d]	47.05	10.87	2.92	-3.5	3.4	NA[d]	25.88	NA[d]
Qatar	NA[d]	NA[d]	4	3.24	0.87	260.36	5.22	0.04	NA[d]	NA[d]	0	3.25	NA[d]
South Africa	NA[d]	NA[d]	3	2.86	NA[d]	668.7	37.52	3.96	-3.6	2.5	1	13.5	NA[d]
South Korea	NA[d]	NA[d]	4	2.86	NA[d]	32.65	27.1	3.03	0.4	1.5	0	9.25	NA[d]
Switzerland	NA[d]	NA[d]	1	3.05	1.77	797.54	15.26	2.52	-0.4	1.1	0	NA[d]	54.08
Thailand	NA[d]	NA[d]	4	2.29	NAd	17.84	173.88	0.11	-0.5	0.93	0	11.13	NA[d]
United Arab Emirates	NA[d]	NA[d]	1	3.05	16.86	968.48	46.09	0.19	NA[d]	NA[d]	0	19.25	NA[d]
Vietnam	NA[d]	NA[d]	4	2.86	NA[d]	0.36	24.03	0	0.8	0.2	0	5	NA[d]

Notes:

a. Google Trends include indicators as insomnia and health literacy.
b. Increase in Google searches for “insomnia”.
c. Google Trends number for “wash hands” and “face mask”.
d. NA: not applicable. Indicates a country not ranked due to incomplete data.

Supplementary Table S24. **Complete ranking and raw scores of indicators in 50 countries and territories in February 2021**

Country	Rank	Performance score (with Google Trends)[a]	Concern indicators										Performance score (without Google Trends)
			Government policy			Vital health and socioeconomic measures						Hygiene education	
			Lockdown efficiency	Health-system policies	People covered by vaccines	One-month cases per 100,000 members of the population	Infection growth rate (%)	Fatality rate (%)	GDP loss (%)	Unemployment rate (%)	Insomnia[b]	Health literacy[c]	
Singapore	1	70.74	4	3.43	3.08	6.84	0.67	0	1.3	0.5	-1	16	74.15
Taiwan	2	69.87	4	2.67	0	0.18	4.83	2.27	6.41	0.03	-2	18.5	70.24
India	3	68.54	4	3.43	0.52	25.7	3.3	0.78	-1.4	-0.9	0	18.63	72.70
Saudi Arabia	4	67.47	4	3.62	1.12	26.74	2.53	1.28	-2	0.8	0	22	70.36
Australia	5	64.56	4	2.67	0.06	0.63	0.56	0	-0.5	0.7	0	11.13	69.97
Italy	6	64.51	3	3.43	3.65	615.65	14.58	2.47	5	0.5	0	35.63	62.58
Denmark	7	63.87	4	3.05	5.19	219.73	6.39	1.85	-1.3	0.8	0	17.75	67.13
New Zealand	8	61.54	4	2.48	0.07	1.53	3.21	1.35	2.4	0.5	1	9.75	68.69
Canada	9	60.03	4	3.05	2.5	231.86	11.17	2.23	0.7	2.6	0	16.875	62.59
Argentina	10	60.01	3	2.86	1.13	398.55	9.35	2.22	7.7	-0.2	0	7.38	65.40
South Korea	11	59.46	4	2.86	0.02	22.48	14.68	1.56	0.4	0.6	0	6.75	64.91
Japan	12	58.55	4	2.29	0.01	33.15	10.75	5.09	0.5	0.5	0	16.38	60.89
Netherlands	13	56.45	3	2.67	4.05	650.66	11.24	1.42	-2.6	0.7	0	14.88	58.71
Poland	14	56.44	3	2.67	4.41	511.54	12.79	3.4	-2.9	1	0	22.38	56.45
Sweden	15	56.38	3	2.48	4.28	894.64	15.94	1.37	-0.3	1.5	0	26.25	55.22
Turkey	16	56.32	2	3.24	5.07	265.74	9.05	1.15	2.5	0.5	1	6.5	63.14
Hong Kong	17	55.75	2	2.67	0.14	7.38	5.29	3.25	17	3.5	1	7.5	62.13
United Kingdom	18	55.38	3	3.05	15.53	531.24	9.42	4.64	-3.9	0.9	1	13	60.02
Finland	19	54.96	4	2.48	4.08	224.41	27.49	0.57	0.1	1.2	0	5.5	59.66
Austria	20	54.17	3	3.43	3.92	500.11	10.87	1.86	-1.9	2.6	1	11.5	58.96
Germany	21	54.08	2	2.48	3.76	268.11	10.09	5.78	-0.9	0.4	-1	13.63	54.04
Thailand	22	53.85	4	2.29	0	9.19	32.69	0.09	-0.5	0.93	0	6	58.12
Mexico	23	53.63	4	3.05	0.95	172.71	11.94	12.21	-2.3	0.8	0	20.63	53.46
Chile	24	53.62	3	3.05	8.82	510.12	13.41	2.17	0.1	2.5	1	9.375	58.91

Malaysia	25	53.24	3	2.86	0.03	265.07	39.91	0.43	-1.2	1.5	0	25.13	51.63
Portugal	26	53.18	3	3.24	4.47	824.25	11.66	4.56	-3.1	0.3	0	7.88	56.72
France	27	53.16	1	3.43	3.48	828.42	17.19	1.85	6.7	0.3	0	10.25	55.98
Indonesia	28	52.73	2	3.24	0.49	93.71	23.77	2.41	-2.23	1.32	1	23.25	53.64
South Africa	29	51.95	4	3.05	0.06	100.55	4.1	9.77	-3.6	2.5	1	13.88	55.47
Spain	30	51.46	3	3.05	4.1	952.7	16.24	2.43	0	1.57	2	18.75	55.48
Hungary	31	50.03	3	2.86	4.85	631.58	16.6	4.02	-4.2	1	0	3.38	54.13
Colombia	32	49.41	3	2.67	0.13	308.17	7.49	3.69	0.4	3.7	3	18.38	55.12
Brazil	33	47.91	1	3.43	1.98	633.48	14.63	2.26	1.3	2.8	1	12.13	50.95
United States	34	45.03	1	3.05	11.36	725.7	9.15	2.73	0.1	2.7	4	15.25	52.66
Peru	35	44.24	3	2.67	0.49	562.98	16.31	10.07	7.5	7.4	0	19.75	41.99
Czech Republic	36	43.02	3	3.43	3.11	2341.08	25.46	1.61	-0.3	1.3	1	4.25	47.20
Belgium	NA[d]	NA[d]	1	3.05	3.75	529.42	8.64	1.61	1.4	1.7	NA[d]	NA[d]	55.05
China	NA[d]	NA[d]	4	2.86	1.82	0.02	0.39	0	25.1	-0.7	NA[d]	NA[d]	84.12
Egypt	NA[d]	NA[d]	4	3.05	NA[d]	16.1	9.93	8.33	-2.1	-0.3	0	15.25	NA[d]
Greece	NA[d]	NA[d]	2	3.24	4.25	327.57	21.75	2.07	-1.8	0.2	0	NA[d]	56.25
Ireland	NA[d]	NA[d]	3	3.05	4.45	466.71	11.72	4.39	5.8	2.7	NA[d]	NA[d]	58.37
Israel	NA[d]	NA[d]	3	3.62	46.88	1529.33	20.57	0.72	-1	1.7	0	NA[d]	65.35
Norway	NA[d]	NA[d]	2	2.86	4.73	148.31	12.77	0.72	-2.8	1.4	0	NA[d]	58.14
Pakistan	NA[d]	NA[d]	4	3.05	0.02	15.82	6.39	3.47	NA[d]	NA[d]	NA[d]	NA[d]	NA[d]
Philippines	NA[d]	NA[d]	2	2.67	0	46.3	9.65	3.09	-3.5	3.4	NA[d]	44.88	52.08
Qatar	NA[d]	NA[d]	1	3.62	2.43	427.93	8.15	0.08	NA[d]	NA[d]	0	3.13	NA[d]
Russia	NA[d]	NA[d]	1	2.67	NAd	267.28	10.24	3.25	-2.1	1.1	0	28.75	NA[d]
Switzerland	NA[d]	NA[d]	1	3.05	4.67	388.37	6.45	1.74	-0.4	1.1	1	NA[d]	56.74
United Arab Emirates	NA[d]	NA[d]	1	3.43	30.44	888.89	28.96	0.42	NA[d]	NA[d]	0	10.63	NA[d]
Vietnam	NA[d]	NA[d]	2	2.67	NA[d]	0.65	34.73	0	0.8	0.2	0	6.5	NA[d]

Notes:

a. Google Trends include indicators as insomnia and health literacy.

b. Increase in Google searches for “insomnia”.

c. Google Trends number for “wash hands” and “face mask”.

d. NA: not applicable. Indicates a country not ranked due to incomplete data.

***Supplementary Table S25.* Complete ranking and raw scores of indicators in 50 countries and territories in March 2021**

Country	Rank	Performance score (with Google Trends)[a]	Concern indicators										Performance score (without Google Trends)
			Government policy			Vital health and socioeconomic measures						Hygiene education	
			Lockdown efficiency	Health-system policies	People covered by vaccines	One-month cases per 100,000 members of the population	Infection growth rate (%)	Fatality rate (%)	GDP loss (%)	Unemployment rate (%)	Insomnia[b]	Health literacy[c]	
Singapore	1	68.77	4	3.43	11.27	7.61	0.74	0.22	1.3	0.5	0	13.6	73.61
South Korea	2	65.57	4	3.24	0.87	26.54	15.11	0.96	0.4	0.1	-1	15.1	66.53
Taiwan	3	64.82	4	2.67	0.03	0.31	7.85	1.33	6.41	-0.04	0	13.4	68.77
Saudi Arabia	4	64.58	4	3.81	6.37	36.26	3.35	1.39	-2	0.8	1	16	69.94
Australia	5	63.51	4	2.86	1.31	1.35	1.19	0	-0.5	0.4	0	8.1	69.20
Egypt	6	63.12	4	3.43	0.08	19.26	10.8	6.63	-2.1	-0.3	0	27.1	61.28
New Zealand	7	62.82	4	2.67	0.76	2.55	5.17	0	2.4	0.5	0	7.3	68.64
Russia	8	62.74	4	2.67	3.9	202.72	7.05	4.23	-2.1	0.7	0	26.1	61.20
Canada	9	62.72	4	3.24	7.54	313.31	13.57	0.82	0.7	-0.3	1	15.5	67.81
Hong Kong	10	62.12	2	3.43	3.37	6.16	4.2	1.3	17	2.6	0	7.2	67.81
Vietnam	11	62.10	4	2.86	0.03	0.16	6.33	0	0.8	0.2	0	5.4	68.49
Thailand	12	61.77	4	2.1	0.13	4.06	10.88	0.39	-0.5	0.93	-2	5.2	63.15
Argentina	13	61.24	3	3.05	4.31	534.24	11.46	1.61	7.7	-0.2	0	11.8	64.91
India	14	60.82	4	3.62	2.36	80.39	9.98	0.52	-1.4	-2.3	2	6	71.65
Austria	15	60.11	3	3.43	9.55	963.64	18.89	0.9	-1.9	-2.8	0	12.2	63.33
Malaysia	16	59.98	2	3.24	1.09	138.26	14.88	0.32	-1.2	0.8	0	22.8	59.04
Japan	17	59.78	4	2.29	0.4	33.64	9.85	2.98	0.5	0.1	0	13.2	62.54
United Kingdom	18	59.51	2	3.24	26.27	252.12	4.09	2.26	-3.9	0.8	0	10.4	63.30
Germany	19	58.69	3	2.67	8.44	469.48	16.05	1.64	-0.9	0.2	-1	10.8	59.62
Portugal	20	57.80	2	3.43	8.69	168.29	2.13	3.09	-3.1	0.3	0	10.8	61.00
Italy	21	57.77	3	3.43	8.66	1090.99	22.55	1.77	5	2.7	0	20.9	57.01
Indonesia	22	57.36	2	3.43	2.13	64.74	13.27	2.65	-2.23	1.32	0	19.7	56.98
Turkey	23	56.77	3	3.24	9.39	729.9	22.79	0.48	2.5	0.2	0	6.2	61.51

France	24	56.28	3	3.43	8.62	1316.42	23.31	1.04	6.7	0.3	0	7.9	60.23
South Africa	25	54.40	4	3.05	0.22	58.62	2.3	8.21	-3.6	2.5	0	12.1	56.24
Netherlands	26	54.18	3	2.86	7.26	1104.43	17.15	0.53	-2.6	0.6	0	9.9	56.82
Chile	27	53.86	3	3.24	27.46	894.07	20.73	1.5	0.1	2.2	2	12	60.61
Sweden	28	53.09	3	2.67	8.15	1461.26	22.45	0.43	-0.3	2.9	0	19.2	51.83
Denmark	29	52.31	1	3.05	9.65	335.14	9.16	0.3	-1.3	0.3	1	6.7	58.24
Colombia	30	51.82	2	2.67	2.2	304.01	6.87	2.36	0.4	1.6	1	10.4	56.18
Spain	31	51.03	3	3.05	8.59	204.9	3	6.59	0	1.57	2	8.3	58.52
United States	32	50.87	1	3.24	22.7	547.93	6.33	2.07	0.1	1.6	3	14.3	58.47
Mexico	33	50.65	4	3.05	3.04	117.85	7.28	11.51	-2.3	1	1	11.8	54.18
Peru	34	50.39	3	3.05	1.58	682.23	16.99	9.3	7.5	7.5	0	29.2	44.54
Brazil	35	49.49	3	3.43	4.51	1033.82	20.83	3.03	1.3	2.5	3	21.1	54.08
Poland	36	49.46	3	2.67	8.28	1624.27	36.01	1.51	-2.9	1	0	17.7	47.88
Finland	37	46.86	1	2.86	8.86	356.99	34.3	0.52	0.1	0.8	0	1.2	51.07
Czech Republic	38	45.10	3	3.62	8.09	2771.99	24.03	2.05	-0.3	1.2	0	1.4	48.80
Hungary	39	41.74	3	3.05	14.77	2317.04	52.22	2.57	-4.2	0.8	0	8.1	41.98
Belgium	NA[d]	NA[d]	1	3.05	8.56	957.25	14.38	0.85	1.4	1.9	NA[d]	NA[d]	52.56
China	NA[d]	NA[d]	4	2.86	4.16	0.02	0.34	0	25.1	-0.6	NA[d]	NA[d]	82.16
Greece	NA[d]	NA[d]	3	3.62	8.15	696.43	37.98	2.19	-1.8	1	0	NA[d]	54.26
Ireland	NA[d]	NA[d]	2	3.24	8.76	329.34	7.41	2.26	5.8	2.7	NA[d]	NA[d]	60.21
Israel	NA[d]	NA[d]	1	3.81	58.02	661.98	7.39	0.8	-1	1.8	1	NA[d]	67.76
Norway	NA[d]	NA[d]	3	3.05	9.25	462.5	35.31	0.2	-2.8	1.4	1	NA[d]	55.67
Pakistan	NA[d]	NA[d]	4	3.24	0.18	41.45	15.75	1.78	NA[d]	NA[d]	NA[d]	NA[d]	NAd
Philippines	NA[d]	NA[d]	2	2.86	0.34	155.99	29.66	0.57	-3.5	3.4	NA[d]	33.2	49.64
Qatar	NA[d]	NA[d]	1	3.62	14.17	565.76	9.96	0.2	NA[d]	NA[d]	3	9.6	NA[d]
Switzerland	NA[d]	NA[d]	1	3.05	8.98	533.73	8.32	0.8	-0.4	0.5	0	NA[d]	57.00
United Arab Emirates	NA[d]	NA[d]	1	3.62	42	706.95	17.86	0.39	NA[d]	NA[d]	1	16.2	NA[d]

Notes:

a. Google Trends include indicators as insomnia and health literacy.

b. Increase in Google searches for "insomnia".

c. Google Trends number for "wash hands" and "face mask".
d. NA: not applicable. Indicates a country not ranked due to incomplete data.

Supplementary Table S26. **Complete ranking and raw scores of indicators in 50 countries and territories in April 2021**

			Concern indicators										
			Government policy			Vital health and socioeconomic measures						Hygiene education	
Country	Rank	Performance score (with Google Trends)[a]	Lockdown efficiency	Health-system policies	People covered by vaccines	One-month cases per 100,000 members of the population	Infection growth rate (%)	Fatality rate (%)	GDP loss (%)	Unemployment rate (%)	Insomnia[b]	Health literacy[c]	Performance score (without Google Trends)
United Kingdom	1	73.63	4	3.24	36.33	106.45	1.66	1.13	43.6	0.7	0	10.13	81.31
Singapore	2	69.91	4	3.81	18.92	13.06	1.27	0	28	-0.1	1	16.88	76.71
Malaysia	3	68.16	4	3.24	2.25	195.31	18.3	0.37	33.3	-0.4	-1	18	70.02
Russia	4	67.68	4	3.24	6.8	175.78	5.71	4.32	18.1	-0.6	0	43.75	63.77
Italy	5	63.08	3	3.62	16.73	724.02	12.21	2.62	35.5	2.8	0	21.63	64.66
Germany	6	61.00	3	3.05	17.72	670.44	19.75	1.16	20.5	0.1	-2	11.75	60.86
Spain	7	60.98	3	3.05	17.5	512.73	7.3	1.15	41.4	-0.07	2	9	69.99
Portugal	8	60.71	2	3.43	16.33	144.86	1.8	0.85	31.9	1	0	2.13	67.56
Austria	9	60.25	3	3.43	17.81	806.55	13.3	1.19	25	-4.2	0	6.5	65.66
South Korea	10	59.27	4	3.43	3.5	37.05	18.33	0.51	8.5	-0.1	0	9	63.70
Japan	11	59.11	4	2.48	1.73	93.79	24.99	0.88	17.6	0.2	0	15	61.69
Taiwan	12	58.70	4	3.05	0.12	0.41	9.51	2.04	7.08	-0.39	0	15	61.18
United States	13	58.60	1	3.62	36.28	569.71	6.19	1.26	21.2	-8.7	3	14.25	67.53
France	14	58.00	3	3.62	16.55	1439.77	20.67	0.91	37.1	0.9	0	9.25	62.03
Vietnam	15	57.64	4	3.24	0.27	0.33	12.49	0	6.22	-0.11	0	4.38	63.04
Hong Kong	16	57.24	2	3.43	9.43	4.09	2.68	1.3	16.5	1.2	0	7.75	61.53
Mexico	17	53.08	4	3.05	7.01	82.11	4.73	12.94	38.4	0	3	10.25	61.82
Poland	18	52.63	3	2.86	15.15	1242.98	20.26	3.07	19.2	0.5	0	16.38	53.18
Czech Republic	19	49.22	1	3.43	14.91	919.1	6.42	2.89	18.6	0.7	0	5.5	52.18
Sweden	20	44.61	1	2.67	15.69	1670.6	20.96	0.35	17.7	1.2	1	16.75	45.12
Argentina	NA[d]	NA[d]	3	3.24	8.7	1390.71	26.76	1.27	NA[d]	NA[d]	0	12.38	NA[d]
Australia	NA[d]	NA[d]	4	3.24	4.27	1.92	1.67	0.2	NA[d]	-0.9	-1	10	NA[d]

Belgium	NA[d]	NA[d]	1	3.24	17.06	929.94	12.21	1.13	28.5	1.8	NA[d]	NA[d]	55.62
Brazil	NA[d]	NA[d]	3	3.62	10.04	898.7	14.98	4.31	NA[d]	2.1	4	20.13	NA[d]
Canada	NA[d]	NA[d]	3	3.24	17.78	635.4	24.24	0.53	NA[d]	-4.9	0	18.25	NA[d]
Chile	NA[d]	NA[d]	3	3.43	39.05	1060.39	20.36	1.59	NA[d]	1.2	0	15.5	NA[d]
China	NA[d]	NA[d]	4	3.43	9.21	0.03	0.5	0	4.7	-0.9	NA[d]	NA[d]	66.66
Colombia	NA[d]	NA[d]	3	3.05	4.9	890.96	18.84	2.27	NA[d]	-4.7	1	13.38	NA[d]
Denmark	NA[d]	NA[d]	1	3.05	17.08	344.5	8.63	0.32	NA[d]	-0.9	0	20.88	NA[d]
Egypt	NA[d]	NA[d]	4	3.05	0.32	24.84	12.58	5.29	NA[d]	-2.3	0	7.13	NA[d]
Finland	NA[d]	NA[d]	4	2.86	16.52	168.86	12.08	0.75	NA[d]	0.9	-1	2.5	NA[d]
Greece	NA[d]	NA[d]	3	3.43	14.86	780.42	30.85	2.81	NA[d]	1.2	0	NA[d]	NA[d]
Hungary	NA[d]	NA[d]	3	3.43	30.91	1313.77	19.45	5.36	NA[d]	0.6	0	3.5	NA[d]
India	NA[d]	NA[d]	2	3.81	5.51	503.14	56.81	0.7	NA[d]	-15.5	0	11.13	NA[d]
Indonesia	NA[d]	NA[d]	2	3.43	3.67	57.27	10.36	2.98	12.39	NA[d]	1	17.25	NA[d]
Ireland	NA[d]	NA[d]	2	3.24	15.93	263.6	5.52	1.66	NA[d]	2.6	NA[d]	NA[d]	NA[d]
Israel	NA[d]	NA[d]	4	3.81	60.38	62.11	0.65	2.86	NA[d]	1.7	1	NA[d]	NA[d]
Netherlands	NA[d]	NA[d]	3	2.86	14.74	1343.27	17.8	0.31	NA[d]	0	0	11.75	NA[d]
New Zealand	NA[d]	NA[d]	4	2.86	2.86	2.32	4.48	0	NA[d]	0	1	5.63	NA[d]
Norway	NA[d]	NA[d]	3	3.05	16.5	311.57	17.58	0.49	NA[d]	0.5	0	NAd	NA[d]
Pakistan	NA[d]	NA[d]	2	3.24	0.48	69.08	22.68	2.25	NA[d]	NA[d]	NA[d]	NA[d]	NA[d]
Peru	NA[d]	NA[d]	3	3.24	2.75	760.16	16.18	9.29	NA[d]	6.1	0	34.13	NA[d]
Philippines	NA[d]	NA[d]	2	3.43	0.88	264.8	38.83	1.36	28.8	-9	NA[d]	25.13	64.11
Qatar	NA[d]	NA[d]	3	3.81	27.01	891.62	14.27	0.65	NA[d]	NA[d]	0	13.75	NA[d]
Saudi Arabia	NA[d]	NA[d]	4	3.81	13.42	78.58	7.01	1.05	8.5	NA[d]	0	15.38	NA[d]
South Africa	NA[d]	NA[d]	4	3.05	0.27	55.73	2.13	4.55	NA[d]	NA[d]	0	13.38	NA[d]
Switzerland	NA[d]	NA[d]	1	3.43	16.32	679.98	9.79	0.5	NA[d]	0	0	NA[d]	NA[d]
Thailand	NA[d]	NA[d]	4	2.1	1.06	51.99	125.73	0.3	19.6	NA[d]	1	9.5	NA[d]
Turkey	NA[d]	NA[d]	3	3.43	13.53	1782.58	45.32	0.57	NA[d]	0.3	0	6.5	NA[d]

United Arab Emirates	NA[d]	NA[d]	1	3.62	53.32	594.44	12.74	0.15	NA[d]	NA[d]	0	5.5	NA[d]

Notes:

a. Google Trends include indicators as insomnia and health literacy.

b. Increase in Google searches for “insomnia”.

c. Google Trends number for “wash hands” and “face mask”.

d. NA: not applicable. Indicates a country not ranked due to incomplete data.

***Supplementary Table S27*. Complete ranking and raw scores of indicators in 50 countries and territories in May 2021**

			Concern indicators										
			Government policy			Vital health and socioeconomic measures						Hygiene education	
Country	Rank	Performance score (with Google Trends)[a]	Lockdown efficiency	Health-system policies	People covered by vaccines	One-month cases per 100,000 members of the population	Infection growth rate (%)	Fatality rate (%)	GDP loss (%)	Unemployment rate (%)	Insomnia[b]	Health literacy[c]	Performance score (without Google Trends)
Singapore	1	78.53	4	3.81	34.59	15.49	1.48	0.33	28	-0.1	0	30.5	77.61
United Kingdom	2	76.81	4	3.43	48.03	104.56	1.6	0.38	43.6	0.7	0	10.13	82.50
Austria	3	68.77	3	3.43	28.88	288.07	4.19	1.53	25	-3.8	0	11.88	71.85
Spain	4	68.61	3	3.05	29	330.05	4.38	1.13	41.4	-0.07	0	10.5	72.12
Italy	5	66.96	3	3.62	29.2	322.8	4.85	2.73	35.5	1.7	0	15.38	68.37
United States	6	65.77	1	3.62	44.55	276.96	2.83	1.98	21.2	-7.5	2	16.38	71.55
Russia	7	65.26	4	3.24	9.77	180.05	5.53	4.25	18.1	-1.2	0	23.88	63.31
Portugal	8	64.14	2	3.62	28.34	123.57	1.51	0.4	31.9	1	0	4	68.80
Malaysia	9	63.94	3	3.43	4.68	505.6	40.04	0.79	33.3	-0.8	1	20.75	65.25
South Korea	10	63.26	4	3.43	7.77	35.43	14.81	0.73	8.5	-0.5	0	13.13	64.54
Germany	11	62.23	3	3.05	30.26	339.63	8.36	1.93	20.5	-0.1	0	9.13	64.63
Mexico	12	61.63	4	3.24	11.82	53.51	2.94	9.66	38.4	-0.2	0	9	63.92
France	13	60.91	1	3.81	27.49	591.47	0.9	1.25	37.1	0.9	0	7.25	63.63
Hong Kong	14	60.41	2	3.62	16.01	0.89	0.57	1.49	16.5	0.1	0	10	62.05
Japan	15	59.33	4	2.48	6.59	121.7	25.95	1.82	17.6	0.1	1	14.75	61.56
Sweden	16	58.60	3	2.86	26.66	939.36	9.74	0.42	17.7	0.8	0	12.38	58.96
Poland	17	57.61	2	3.24	26.53	211.75	2.87	7.79	19.2	0.1	-1	12.88	55.06
Taiwan	18	54.75	2	3.62	0.67	31	654.52	1.52	7.08	-0.01	-1	36.125	43.43
Czech Republic	19	53.33	1	3.24	25.05	284.94	1.87	2.76	18.6	0.3	0	0.5	56.49
Vietnam	20	53.18	2	3.24	0.57	4.63	153.83	0.27	6.22	-0.11	0	9.38	53.23
Argentina	NA[d]	NA[d]	3	3.24	13.56	1779.86	27.02	1.77	NA[d]	NA[d]	0	9.25	NA[d]
Australia	NA[d]	NA[d]	4	3.24	8.32	1.2	1.03	0	NA[d]	-2	0	7.13	NA[d]

Belgium	NA[d]	NA[d]	1	3.43	29.6	619.28	7.25	1.01	28.5	1.5	NA[d]	NA[d]	59.41
Brazil	NA[d]	NA[d]	3	3.62	15.87	887.54	12.87	3.13	NA[d]	1.7	4	24	NA[d]
Canada	NA[d]	NA[d]	3	3.62	31.61	425.28	13.06	0.81	NA[d]	-5.5	-1	14.13	NA[d]
Chile	NA[d]	NA[d]	3	3.43	49.1	973.52	15.53	1.58	NA[d]	-1.2	0	9.75	NA[d]
China	NA[d]	NA[d]	4	3.62	22.98	0.03	0.5	0	4.7	-0.9	NA[d]	NA[d]	68.64
Colombia	NA[d]	NA[d]	3	3.24	9.92	1074.49	19.12	2.75	NA[d]	-5.8	1	10.63	NA[d]
Denmark	NA[d]	NA[d]	1	3.05	29.68	530.64	12.23	0.11	NA[d]	-1.6	0	12.38	NA[d]
Egypt	NA[d]	NA[d]	2	3.05	1.26	34.3	15.42	5.01	NA[d]	-2.3	1	12.25	NA[d]
Finland	NA[d]	NA[d]	4	2.86	26.55	102.51	6.54	0.74	NA[d]	-1	-1	0	NA[d]
Greece	NA[d]	NA[d]	1	3.81	26.85	549.48	16.6	2.99	NA[d]	-1.3	0	NA[d]	NA[d]
Hungary	NA[d]	NA[d]	1	3.43	45.71	260.76	3.23	8.71	NA[d]	0	0	2.88	NA[d]
India	NA[d]	NA[d]	3	3.43	7.62	652.9	47.01	1.33	NA[d]	-9.8	2	13	NA[d]
Indonesia	NA[d]	NA[d]	2	3.43	4.94	56.06	9.19	3.3	12.39	NA[d]	1	16.63	NA[d]
Ireland	NA[d]	NA[d]	4	3.24	24.17	266.78	5.29	0.29	NA[d]	1.9	NA[d]	NA[d]	NA[d]
Israel	NA[d]	NA[d]	4	3.62	61.16	11.48	0.12	4.93	NA[d]	1.2	1	NA[d]	NA[d]
Netherlands	NA[d]	NA[d]	3	2.86	28.2	897.42	10.1	0.32	NA[d]	-0.3	0	10.75	NA[d]
New Zealand	NA[d]	NA[d]	4	2.86	6.75	1.26	2.33	0	NA[d]	0	1	7.5	NA[d]
Norway	NA[d]	NA[d]	4	3.05	24.9	224.04	10.75	0.22	NA[d]	0.5	0	NA[d]	NA[d]
Pakistan	NA[d]	NA[d]	2	3.43	1.39	44.05	11.79	2.97	NA[d]	NA[d]	NA[d]	NA[d]	NA[d]
Peru	NA[d]	NA[d]	3	3.43	6.55	473.2	8.67	10.95	NA[d]	-1.1	0	31.38	NA[d]
Philippines	NA[d]	NA[d]	2	3.62	2.36	175.98	18.59	1.94	28.8	-9	NA[d]	26.63	71.00
Qatar	NA[d]	NA[d]	3	3.81	44.17	409.78	5.74	0.83	NA[d]	NA[d]	0	8.5	NA[d]
Saudi Arabia	NA[d]	NA[d]	4	3.81	20.41	95	7.92	1.22	8.5	NA[d]	1	8.75	NA[d]
South Africa	NA[d]	NA[d]	4	3.05	0.88	142.32	5.34	2.55	NA[d]	NA[d]	0	13.13	NA[d]
Switzerland	NA[d]	NA[d]	1	3.43	28.88	401.7	5.27	0.51	NA[d]	-0.3	0	NA[d]	NA[d]
Thailand	NA[d]	NA[d]	4	2.86	2.63	135.59	145.26	0.87	19.6	NA[d]	3	9.38	NA[d]
Turkey	NA[d]	NA[d]	3	3.43	17.29	508.44	8.9	1.72	NA[d]	-0.4	1	6.38	NA[d]

United Arab Emirates	NA[d]	NA[d]	1	3.81	65.07	511.61	9.73	0.18	NA[d]	NA[d]	1	5.63	NA[d]

Notes:

a. Google Trends include indicators as insomnia and health literacy.

b. Increase in Google searches for "insomnia".

c. Google Trends number for "wash hands" and "face mask".

d. NA: not applicable. Indicates a country not ranked due to incomplete data.

***Supplementary Table S28.* Complete ranking and raw scores of indicators in 50 countries and territories in June 2021**

Country	Rank	Performance score (with Google Trends)[a]	Concern indicators										Performance score (without Google Trends)
			Government policy			Vital health and socioeconomic measures						Hygiene education	
			Lockdown efficiency	Health-system policies	People covered by vaccines	One-month cases per 100,000 members of the population	Infection growth rate (%)	Fatality rate (%)	GDP loss (%)	Unemployment rate (%)	Insomnia[b]	Health literacy[c]	
Singapore	1	66.54	2	4	45.86	9.03	0.85	0.57	28	-0.1	1	16.6	71.81
Czech Republic	2	66.21	4	3.43	38.65	56.17	0.36	3.24	18.6	0	-1	8.8	69.64
Spain	3	65.69	1	3.24	44.94	279.27	3.55	0.71	41.4	-0.07	-1	12	68.00
France	4	65.34	1	3.81	40.52	160.55	1.89	1.43	37.1	0.9	-2	7.1	66.99
Austria	5	64.95	2	3.43	43.36	62.14	0.87	1.77	25	-3.1	0	5.7	71.10
United Kingdom	6	64.40	1	3.43	57.38	462.64	6.97	0.11	43.6	0.6	0	9.5	69.24
Italy	7	64.31	2	3.81	42.99	69.61	1	3.42	35.5	0.4	1	13.7	69.92
Portugal	8	64.09	3	3.62	41.77	298.76	3.59	0.23	31.9	1	0	3.4	70.73
United States	9	63.82	1	3.62	49.32	120.05	1.19	2.6	21.2	-5.2	1	11.9	69.86
South Korea	10	61.93	4	3.43	18.81	33.01	12.02	0.34	8.5	-0.5	0	13.4	64.96
Sweden	11	61.12	3	2.86	39.85	213.06	2.01	0.83	17.7	0.5	0	11.7	64.46
Mexico	12	60.97	4	3.24	17.8	81.85	4.37	8.98	38.4	-1.5	2	10	68.97
Germany	13	60.44	2	3.62	44.98	55.24	1.25	5.06	20.5	-0.2	0	9	64.45
Japan	14	60.18	4	2.48	20.15	42	7.11	3.25	17.6	0.1	0	13.9	62.62
Russia	15	58.66	1	3.81	13.71	298.82	8.7	3.1	18.1	-1.4	0	28	56.37
Poland	16	58.31	4	3.24	38.63	20.16	0.27	16.73	19.2	-0.2	0	15.8	59.69
Malaysia	17	57.87	3	3.43	12.71	554.97	31.38	1.32	33.3	-0.1	1	14.2	61.71
Hong Kong	18	56.08	2	3.62	24.87	1.09	0.69	1.22	16.5	-0.7	2	6.9	63.80
Taiwan	19	45.69	2	3.43	4.29	26.42	73.94	8.33	7.08	0.79	1	27.7	42.33
Vietnam	20	41.15	2	3.24	1.94	9.88	129.44	0.35	6.22	-0.11	1	7.4	42.91
Argentina	NA[d]	NA[d]	3	3.24	22.73	1523.57	18.21	2.35	NA[d]	NA[d]	0	6.6	NA[d]
Australia	NA[d]	NA[d]	2	3.43	14.99	2.06	1.74	0	NA[d]	-2.5	-1	10.7	NA[d]

Belgium	NA[d]	NA[d]	1	3.62	47.53	199.58	2.18	0.94	28.5	0.8	NA[d]	NA[d]	64.95
Brazil	NA[d]	NA[d]	3	3.62	23.25	946.36	12.16	2.75	NA[d]	NA[d]	4	24.3	NA[d]
Canada	NA[d]	NA[d]	2	3.62	49.53	88.38	2.4	2.23	NA[d]	-4.5	-1	14.2	NA[d]
Chile	NA[d]	NA[d]	3	3.62	60.36	897.44	12.39	1.89	NA[d]	-2.7	1	9.9	NA[d]
China	NA[d]	NA[d]	4	3.62	43.24	0.05	0.74	0	4.7	-0.7	NA[d]	NA[d]	70.21
Colombia	NA[d]	NA[d]	3	3.24	17.96	1640.09	24.5	2.13	NA[d]	-5.4	2	10.4	NA[d]
Denmark	NA[d]	NA[d]	1	3.24	45.44	216.12	4.44	0.14	NA[d]	-1.7	0	7.9	NA[d]
Egypt	NA[d]	NA[d]	4	3.05	2.09	18.21	7.09	5.76	NA[d]	-2.3	0	25.5	NA[d]
Finland	NA[d]	NA[d]	4	3.05	39.01	58.73	3.52	0.4	NA[d]	-0.3	1	0	NA[d]
Greece	NA[d]	NA[d]	1	3.81	40.25	193.32	5.01	2.94	NA[d]	NA[d]	0	NA[d]	NA[d]
Hungary	NA[d]	NA[d]	4	3.24	52.56	37.16	0.45	7.21	NA[d]	-0.5	0	1.9	NA[d]
India	NA[d]	NA[d]	3	3.43	11.93	162.07	7.94	3.02	NA[d]	-1	0	12.8	NA[d]
Indonesia	NA[d]	NA[d]	2	3.43	7.81	130.36	19.57	2.22	12.39	NA[d]	1	19	NA[d]
Ireland	NA[d]	NA[d]	1	3.24	40.56	199.79	3.76	0.58	NA[d]	1	NA[d]	NA[d]	NA[d]
Israel	NA[d]	NA[d]	4	3.43	62.22	26.6	0.27	0.74	NA[d]	0.6	0	NA[d]	NA[d]
Netherlands	NA[d]	NA[d]	3	3.05	45	210.12	2.15	0.36	NA[d]	-1.1	0	11.1	NA[d]
New Zealand	NA[d]	NA[d]	4	2.86	12.18	1.43	2.58	0	NA[d]	0	0	6.8	NA[d]
Norway	NA[d]	NA[d]	4	3.24	39.01	114.36	4.96	0.18	NA[d]	0.5	0	NA[d]	NA[d]
Pakistan	NA[d]	NA[d]	2	3.43	3.05	16.11	3.86	4.13	NA[d]	NA[d]	NA[d]	NA[d]	NA[d]
Peru	NA[d]	NA[d]	3	3.43	11.68	292.97	4.94	8.85	NA[d]	-6	0	21.4	NA[d]
Philippines	NA[d]	NA[d]	3	3.43	4.77	166.32	14.81	2.03	28.8	-9	NA[d]	40.6	74.11
Qatar	NA[d]	NA[d]	3	3.62	54.58	160.11	2.12	0.74	NA[d]	NA[d]	0	4.8	NA[d]
Saudi Arabia	NA[d]	NA[d]	4	3.81	25.68	106.73	8.25	1.23	8.5	NA[d]	0	10.4	NA[d]
South Africa	NA[d]	NA[d]	3	3.43	2.55	519.92	18.51	1.34	NA[d]	NA[d]	0	12.5	NA[d]
Switzerland	NA[d]	NA[d]	1	3.24	43.51	95.49	1.19	0.98	NA[d]	-0.4	1	NA[d]	NA[d]
Thailand	NA[d]	NA[d]	4	2.86	7.11	142.56	62.27	1	19.6	NA[d]	0	7.3	NA[d]
Turkey	NA[d]	NA[d]	3	3.43	30	208.98	3.36	1.25	NA[d]	-2.7	0	7	NA[d]

United Arab Emirates	NA[d]	NA[d]	1	3.81	77.66	627.59	10.87	0.21	NA[d]	NA[d]	0	14.3	NA[d]

Notes:

a. Google Trends include indicators as insomnia and health literacy

b. Increase in Google searches for “insomnia”.

c. Google Trends number for “wash hands” and “face mask”.

d. NA: not applicable. Indicates a country not ranked due to incomplete data.

Supplementary Table S29. Complete ranking and raw scores of indicators in 50 countries and territories in July 2021

			Concern indicators										
			Government policy			Vital health and socioeconomic measures						Hygiene education	
Country	Rank	Performance score (with Google Trends)[a]	Lockdown efficiency	Health-system policies	People covered by vaccines	One-month cases per 100,000 members of the population	Infection growth rate (%)	Fatality rate (%)	GDP loss (%)	Unemployment rate (%)	Insomnia[b]	Health literacy[c]	Performance score (without Google Trends)
Singapore	1	77.26	2	4.00	69.46	44.04	3.84	0.04	12.9	-1	0	15.5	79.54
Canada	2	75.15	2	3.43	64.98	40.16	1.07	1.98	9.1	-3.4	0	15.375	76.96
Chile	3	75.12	3	3.62	67.39	311.62	3.85	4.85	26.2	-4.17	1	7.25	83.58
Peru	4	73.93	3	3.62	20.39	177.84	2.89	6.78	20.4	-7	0	12.5	77.06
Japan	5	72.64	4	2.86	35.34	100.77	15.89	0.32	7	-0.1	0	16.875	72.98
Hungary	6	72.27	4	3.43	57.33	14.15	0.17	2.49	10.7	-0.9	0	4.25	79.62
Italy	7	72.17	2	3.62	57.03	149.28	2.12	0.55	9.1	-0.7	0	14.875	73.52
Austria	8	72.01	4	3.62	54.23	95.59	1.33	0.40	9.5	-2.5	1	3	82.07
Saudi Arabia	9	71.51	2	3.81	38.01	107.92	7.82	1.10	11.6	-1.9	0	13.875	73.26
Sweden	10	70.64	4	2.86	51.00	98.92	0.92	0.26	7.2	-0.9	0	5.875	76.66
South Korea	11	70.57	4	3.43	25.02	81.99	26.67	0.18	5	-0.8	0	11.625	73.34
New Zealand	12	70.37	4	3.05	18.79	2.56	4.78	0.00	-0.7	-1.9	-2	7	71.52
Australia	13	70.28	2	3.43	23.91	14.50	12.21	0.37	7.7	-2.9	0	15.375	70.88
Colombia	14	69.75	3	3.24	26.84	1061.79	12.84	2.60	21.7	-5.9	1	15.75	72.09
Poland	15	69.15	4	3.24	45.55	8.01	0.11	7.93	7	-0.3	0	12.625	71.01
Czech Republic	16	69.00	4	3.62	48.51	58.64	0.38	1.11	8	-0.1	0	0.375	77.71
Netherlands	17	68.11	1	3.24	60.50	1052.01	10.71	0.05	7.8	-1.4	-1	20.75	63.06
Germany	18	66.95	2	3.62	55.40	48.29	1.08	1.78	6.3	1.6	-1	6.625	69.55
United States	19	66.71	1	3.62	53.12	396.53	3.91	0.66	7.8	-4.8	2	13.125	71.85
Hong Kong	20	66.13	2	3.62	37.49	0.81	0.51	1.64	9	-1.1	0	5.375	71.31
India	21	65.66	2	3.43	16.56	89.29	4.09	2.00	15.8	-0.4	1	13.75	68.10
Argentina	22	65.57	3	3.24	34.95	1007.31	10.28	2.49	22	-3.5	0	4.125	71.31
Portugal	23	65.31	3	3.43	60.10	876.03	10.13	0.30	9.9	-1.9	0	1.625	72.39

Mexico	24	64.71	3	3.24	25.61	252.55	13.06	2.39	13.2	-1	1	8.25	70.00
France	25	64.06	1	3.62	55.24	522.34	6.05	0.23	6.8	-1	0	8.75	66.83
Spain	26	63.74	1	3.05	60.10	1365.03	16.75	0.10	12	-1.69	0	17.375	61.58
Russia	27	61.88	1	3.43	21.21	504.18	13.50	3.12	7.8	-1.8	0	16	60.03
Denmark	28	61.19	1	3.43	63.25	397.88	7.88	0.06	5.8	-1.5	1	2.625	68.76
Finland	29	61.08	1	3.24	48.66	199.34	11.55	0.14	6.8	-0.6	0	2.25	66.76
United Kingdom	30	60.54	1	3.62	62.41	1554.75	22.07	0.14	15.3	0.3	0	11.25	61.02
Thailand	31	59.69	3	2.86	12.64	483.18	130.35	0.84	6.1	0.35	0	10.75	60.23
Malaysia	32	58.91	3	3.43	32.20	1102.30	48.05	1.07	-1.8	0.1	0	11.875	58.63
Turkey	33	58.67	1	3.43	43.08	354.40	5.55	0.53	1.1	-2.3	1	3.875	64.90
Brazil	34	57.40	3	3.62	33.25	631.72	7.28	2.82	7.9	-0.1	3	6.25	66.15
Taiwan	35	55.45	2	3.43	16.95	3.65	5.88	15.98	-0.56	0.46	1	21.375	51.05
South Africa	36	54.86	3	3.24	6.08	788.58	23.99	2.40	9.1	4.1	1	12.5	55.30
Vietnam	37	37.86	2	3.24	3.16	135.49	780.01	0.92	-8.64	1.22	4	7.875	42.90
Belgium	NA[d]	NA[d]	1	3.81	63.12	340.29	3.65	0.17	9.2	0.2	NA[d]	NA[d]	69.69
China	NA[d]	NA[d]	4	3.62	57.22	0.08	1.32	0.00	0	-0.6	NA[d]	NA[d]	78.07
Egypt	NA[d]	NA[d]	4	3.05	2.56	2.86	1.06	11.91	NA[d]	0.2	0	0	NA[d]
Greece	NA[d]	NA[d]	3	3.62	50.80	683.15	16.77	0.37	23.4	-2.5	0	NA[d]	78.47
Indonesia	NA[d]	NA[d]	3	3.43	12.26	445.57	56.53	2.89	7	NA[d]	4	22.25	NA[d]
Ireland	NA[d]	NA[d]	1	3.43	59.90	583.35	10.69	0.13	3.3	-0.1	NA[d]	NA[d]	64.19
Israel	NA[d]	NA[d]	1	3.43	60.39	347.01	3.83	0.14	7.27	0.2	0	NA[d]	67.35
Norway	NA[d]	NA[d]	4	3.24	49.85	115.47	4.81	0.08	5.3	-1.2	0	NA[d]	77.28
Pakistan	NA[d]	NA[d]	2	3.43	6.58	33.94	7.97	1.44	NA[d]	NA[d]	NA[d]	NA[d]	NA[d]
Philippines	NA[d]	NA[d]	2	3.43	8.72	158.86	12.49	1.83	18.7	-3.1	NA[d]	17.875	70.42
Qatar	NA[d]	NA[d]	4	3.81	64.50	142.23	1.88	0.26	NA[d]	NA[d]	0	0	NA[d]
Switzerland	NA[d]	NA[d]	4	3.43	51.87	168.22	2.09	0.14	5.4	-0.4	0	NA[d]	76.89
United Arab	NA[d]	NA[d]	3	3.81	84.05	479.94	7.58	0.29	NA[d]	NA[d]	0	0	NA[d]

Emirates

Notes:

a. Google Trends include indicators as insomnia and health literacy

b. Increase in Google searches for “insomnia”.

c. Google Trends number for “wash hands” and “face mask”.

d. NA: not applicable. Indicates a country not ranked due to incomplete data.

***Supplementary Table S30*. Complete ranking and raw scores of indicators in 50 countries and territories in August 2021**

			Concern indicators										
			Government policy			Vital health and socioeconomic measures						Hygiene education	
Country	Rank	Performance score (with Google Trends)[a]	Lockdown efficiency	Health-system policies	People covered by vaccines	One-month cases per 100,000 members of the population	Infection growth rate (%)	Fatality rate (%)	GDP loss (%)	Unemployment rate (%)	Insomnia[b]	Health literacy[c]	Performance score (without Google Trends)
Singapore	1	86.01	4	4.00	81.51	48.39	4.06	0.68	12.9	-1	0	18.875	86.53
Canada	2	82.15	4	3.43	70.30	180.30	4.77	0.57	9.1	-3.1	0	15.625	83.60
Peru	3	77.76	2	3.62	28.78	114.50	1.81	5.00	20.4	-6.1	-1	14	76.56
Saudi Arabia	4	77.40	2	3.43	52.54	49.77	3.35	1.56	11.6	-1.9	0	21.5	74.25
Chile	5	76.73	2	3.62	75.62	119.21	1.42	6.50	26.2	-4.35	0	6.75	81.99
Colombia	6	75.66	4	3.62	34.67	241.42	2.59	3.41	21.7	-4.5	2	11.875	82.68
Czech Republic	7	74.11	4	3.62	53.69	52.34	0.34	0.55	8	-0.2	-1	2.125	78.90
Argentina	8	74.09	3	3.43	46.33	561.02	5.19	2.38	22	-3.5	-1	3.125	78.30
Italy	9	74.07	3	3.62	64.65	314.68	4.37	0.61	9.1	-0.6	1	15	76.37
Poland	10	72.77	4	3.24	48.00	15.16	0.20	1.47	7	-0.3	1	12.625	76.12
Hungary	11	72.43	4	3.43	60.22	29.54	0.35	1.12	10.7	-0.5	0	0.5	80.24
South Korea	12	71.24	4	3.81	42.95	104.59	26.86	0.36	5	-0.5	0	6.125	75.49
Australia	13	71.05	2	3.43	38.12	80.31	60.23	0.42	7.7	-2.3	-1	13.25	68.61
Sweden	14	70.09	4	2.67	62.18	263.51	2.43	0.14	7.2	-0.3	2	11.5	75.93
Portugal	15	70.07	3	3.62	72.71	681.52	7.15	0.55	9.9	-1.9	0	0.875	77.08
Netherlands	16	69.88	1	3.62	66.67	445.42	4.09	0.26	7.8	-1.4	0	11.875	70.44
Austria	17	69.30	2	3.62	58.35	323.44	4.44	0.12	9.5	-2.1	0	3.375	74.67
New Zealand	18	69.24	2	3.24	34.32	13.59	24.23	0.00	-0.7	-1.9	0	16.625	66.89
Japan	19	69.07	3	2.67	51.92	450.43	61.27	0.15	7	-0.2	0	17	66.45
France	20	69.03	3	3.62	65.56	925.91	10.12	0.41	6.8	-1	0	6.875	72.29
Hong Kong	21	67.86	2	3.62	50.24	1.69	1.07	0.00	9	-1.4	1	4.5	74.71
Germany	22	67.62	2	3.62	60.94	225.22	5.00	0.30	6.3	1.4	0	7.75	70.01

Spain	23	66.90	1	3.43	70.73	872.86	9.18	0.70	12	-1.69	0	7.875	69.04
Russia	24	66.16	1	3.81	27.50	435.50	10.27	3.79	7.8	-2	-1	12.625	62.86
Denmark	25	65.90	1	3.81	73.75	496.89	9.12	0.12	5.8	-1.4	0	2.625	70.84
Mexico	26	64.25	3	3.43	32.61	387.03	17.70	3.65	13.2	-0.9	2	10.5	69.21
United Kingdom	27	64.14	1	3.62	66.71	1370.01	15.93	0.31	15.3	0	0	8.75	65.09
Brazil	28	63.99	1	3.05	45.37	403.31	4.33	2.79	7.9	-1.3	1	15.25	63.62
India	29	63.47	2	3.43	23.14	82.89	3.65	1.27	15.8	-0.1	0	0	69.34
Finland	30	61.98	1	3.24	61.30	367.32	19.08	0.22	6.8	-1.2	0	0	67.48
Turkey	31	61.44	1	3.24	55.36	777.56	11.55	0.81	1.1	-0.9	-1	4.5	61.69
United States	32	60.74	1	3.62	56.73	1288.11	12.23	0.65	7.8	-3.2	4	18.25	65.31
South Africa	33	59.38	1	3.62	10.47	549.96	13.49	3.10	9.1	4.1	-1	15.875	52.49
Taiwan	34	59.11	2	3.24	22.97	1.35	2.05	14.95	-0.56	0.25	-1	16.875	51.58
Thailand	35	56.07	3	3.43	22.71	868.38	101.70	1.11	6.1	0.35	1	8.125	57.87
Malaysia	36	54.72	3	3.43	54.21	1931.22	56.86	1.21	-1.8	-0.1	1	7.875	56.32
Vietnam	37	36.67	2	3.43	10.29	317.86	207.94	3.13	-8.64	1.22	4	6.875	41.84
Belgium	NA[d]	NA[d]	1	3.81	69.99	515.37	5.33	0.23	9.2	0	NA[d]	NA[d]	69.83
China	NA[d]	NA[d]	2	3.62	71.58	0.13	2.03	0.00	0	-0.5	NA[d]	NA[d]	73.40
Egypt	NA[d]	NA[d]	4	3.05	4.19	4.01	1.47	5.07	NA[d]	0.2	0	14.375	NA[d]
Greece	NA[d]	NA[d]	3	3.62	55.00	912.76	19.19	0.77	23.4	-3.3	1	NA[d]	78.78
Indonesia	NA[d]	NA[d]	3	3.62	17.91	246.11	19.95	5.72	7	NA[d]	2	18.25	NA[d]
Ireland	NA[d]	NA[d]	1	3.62	69.19	1032.95	17.10	0.11	3.3	-1.6	NA[d]	NA[d]	65.23
Israel	NA[d]	NA[d]	3	3.43	74.26	2070.11	22.01	0.30	7.27	0.1	0	NA[d]	64.24
Norway	NA[d]	NA[d]	4	3.43	63.94	412.52	16.38	0.07	5.3	-1.2	0	NA[d]	77.31
Pakistan	NA[d]	NA[d]	2	3.62	12.60	57.22	12.45	1.91	NA[d]	NA[d]	NA[d]	NA[d]	NA[d]
Philippines	NA[d]	NA[d]	2	3.43	15.18	361.01	25.23	1.39	18.7	-1.9	NA[d]	4.75	68.37
Qatar	NA[d]	NA[d]	4	3.81	76.00	221.97	2.88	0.02	NA[d]	NA[d]	0	0	NA[d]

Switzerland	NA[d]	NA[d]	1	3.62	55.46	711.39	8.64	0.17	5.4	-0.6	0	NA[d]	65.18
United Arab Emirates	NA[d]	NA[d]	1	3.81	90.88	375.45	5.51	0.25	NA[d]	NA[d]	0	10.125	NA[d]

Notes:

a. Google Trends include indicators as insomnia and health literacy

b. Increase in Google searches for “insomnia”.

c. Google Trends number for “wash hands” and “face mask”.

d. NA: not applicable. Indicates a country not ranked due to incomplete data.

***Supplementary Table S31*. Complete ranking and raw scores of indicators in 50 countries and territories in September 2021**

Country	Rank	Performance score (with Google Trends)[a]	Concern indicators										Performance score (without Google Trends)
			Government policy			Vital health and socioeconomic measures						Hygiene education	
			Lockdown efficiency	Health-system policies	People covered by vaccines	One-month cases per 100,000 members of the population	Infection growth rate (%)	Fatality rate (%)	GDP loss (%)	Unemployment rate (%)	Insomnia[b]	Health literacy[c]	
Colombia	1	74.72	4	3.62	39.41	94.00	0.98	2.81	21.7	-3.7	0	11.2	80.15
Chile	2	73.86	2	3.43	84.39	81.14	0.95	3.41	26.2	-3.89	0	9.1	80.26
Japan	3	72.62	4	2.86	65.39	167.15	14.10	0.75	7	-0.2	0	18.6	73.36
Peru	4	71.31	2	3.62	41.70	77.08	1.20	4.29	20.4	-6.5	-1	11.2	74.50
South Korea	5	70.95	4	3.81	62.18	117.59	23.80	0.34	5	-1	0	10.5	75.83
Poland	6	70.30	4	3.24	49.23	48.68	0.64	1.66	7	-0.5	0	13.1	73.55
Czech Republic	7	69.95	4	3.62	55.20	114.69	0.73	0.45	8	-0.3	-2	0.5	77.43
Russia	8	69.92	3	3.81	30.94	397.78	8.51	4.06	7.8	-2	-4	18.2	64.65
Spain	9	69.37	4	3.43	75.04	222.54	2.14	1.99	12	-1.69	2	6.1	79.11
Portugal	10	69.32	3	3.62	78.61	308.34	3.02	0.74	9.9	-1.9	0	2.7	78.19
Italy	11	69.31	2	3.81	70.37	219.26	2.92	1.28	9.1	-0.7	0	13	72.38
Denmark	12	69.23	4	3.81	76.14	225.40	3.79	0.55	5.8	-1.5	1	1.2	80.31
Taiwan	13	69.08	4	3.81	33.29	0.96	1.43	3.07	-0.56	0.14	-1	18.9	67.38
Canada	14	69.08	1	3.62	73.96	327.19	8.26	0.74	9.1	-2.1	0	15.9	70.45
France	15	69.01	3	3.62	70.71	391.14	3.88	0.85	6.8	-1	0	9.1	74.19
New Zealand	16	68.74	2	3.62	51.03	14.09	20.23	0.14	-0.7	-1.9	1	22.2	67.87
Hungary	17	68.35	4	3.43	62.86	107.62	1.28	1.27	10.7	-0.5	0	1.6	77.59
Austria	18	67.71	3	3.62	60.81	605.88	7.96	0.43	9.5	-1.9	0	6.7	73.91
India	19	67.19	2	3.62	31.81	68.60	2.91	0.97	15.8	0.2	1	16.3	69.25
Singapore	20	67.16	1	4.00	86.21	529.94	42.74	0.14	12.9	-1	-1	12.2	68.75
Hong Kong	21	65.76	2	3.62	57.44	1.39	0.87	0.95	9	-1.9	1	5.7	73.44
Germany	22	65.27	3	3.62	64.44	326.69	6.91	0.54	6.3	1.4	0	6.8	70.82

Argentina	23	65.27	2	3.43	56.69	156.30	1.37	4.72	22	-3.5	0	5.1	71.77
Brazil	24	64.68	2	3.24	55.89	244.07	3.11	3.11	7.9	-2	0	13.4	66.36
Saudi Arabia	25	64.05	2	3.81	59.58	10.80	0.70	5.35	11.6	-1.9	2	14.9	67.51
Netherlands	26	62.89	1	3.62	68.50	364.28	3.22	0.28	7.8	-1.3	0	3.7	69.59
Turkey	27	62.11	3	3.24	64.38	900.42	11.99	0.96	1.1	-1.2	-1	5.3	66.32
United Kingdom	28	61.82	1	3.62	69.37	1493.22	14.98	0.41	15.3	-0.5	-1	9.5	63.59
Sweden	29	61.15	1	2.67	67.51	256.62	2.31	0.66	7.2	-0.1	0	8.2	64.88
Malaysia	30	60.58	3	3.43	68.53	1523.79	28.60	1.94	-1.8	-0.1	0	19.4	57.86
Finland	31	59.88	1	3.24	68.04	259.00	11.30	0.35	6.8	-0.6	-1	0	66.52
Australia	32	59.73	2	3.62	54.36	201.98	94.55	0.57	7.7	-2.3	-1	11	60.14
Mexico	33	58.47	1	3.43	38.84	239.37	9.30	5.83	13.2	-0.9	1	19.5	56.55
United States	34	56.36	1	3.62	60.08	1246.41	10.54	1.41	7.8	-3	5	13.7	62.74
Thailand	35	55.43	3	3.24	32.90	570.04	33.10	1.29	6.1	0.35	1	5.9	60.41
South Africa	36	52.74	1	3.81	14.58	208.21	4.50	4.29	9.1	4.1	0	14.9	50.59
Vietnam	37	42.57	3	3.81	21.84	334.79	71.12	2.51	-8.64	1.22	4	6	48.45
Belgium	NA[d]	NA[d]	1	3.81	72.33	518.30	5.09	0.37	9.2	0	NA[d]	NA[d]	68.44
China	NA[d]	NA[d]	4	3.62	76.56	0.09	1.33	0.00	0	-0.5	NA[d]	NA[d]	79.02
Egypt	NA[d]	NA[d]	4	3.05	8.13	15.43	5.58	3.70	NA[d]	0.2	0	6.8	NA[d]
Greece	NA[d]	NA[d]	3	3.62	58.50	653.79	11.53	1.68	23.4	-3.7	0	NA[d]	77.79
Indonesia	NA[d]	NA[d]	2	3.62	25.73	45.34	3.06	7.12	7	NA[d]	-1	16.3	NA[d]
Ireland	NA[d]	NA[d]	1	3.62	72.85	752.27	10.64	0.42	3.3	-1.9	NA[d]	NA[d]	65.95
Israel	NA[d]	NA[d]	3	3.43	81.91	2323.39	20.24	0.33	7.27	0.5	0	NA[d]	61.85
Norway	NA[d]	NA[d]	1	3.62	71.95	535.29	18.27	0.16	5.3	-1.2	0	NA[d]	66.33
Pakistan	NA[d]	NA[d]	2	3.62	18.39	36.79	7.12	2.29	NA[d]	NA[d]	NA[d]	NA[d]	NA[d]
Philippines	NA[d]	NA[d]	3	3.62	20.53	504.39	28.15	0.87	18.7	-1.1	NA[d]	9.4	67.91
Qatar	NA[d]	NA[d]	2	4.00	80.41	133.05	1.68	0.10	NA[d]	NA[d]	0	3.4	NA[d]

Switzerland	NA[d]	NA[d]	1	3.62	60.20	696.43	7.79	0.31	5.4	-0.6	1	NA[d]	64.33
United Arab Emirates	NA[d]	NA[d]	4	3.81	100.35	176.38	2.45	0.32	NA[d]	NA[d]	0	14.6	NA[d]

Notes:

a. Google Trends include indicators as insomnia and health literacy

b. Increase in Google searches for "insomnia".

c. Google Trends number for "wash hands" and "face mask".

d. NA: not applicable. Indicates a country not ranked due to incomplete data.

***Supplementary Table S32*. Complete ranking and raw scores of indicators in 50 countries and territories in October 2021**

Country	Rank	Performance score (with Google Trends)[a]	Concern indicators										Performance score (without Google Trends)
			Government policy			Vital health and socioeconomic measures						Hygiene education	
			Lockdown efficiency	Health-system policies	People covered by vaccines	One-month cases per 100,000 members of the population	Infection growth rate (%)	Fatality rate (%)	GDP loss (%)	Unemployment rate (%)	Insomnia[b]	Health literacy[c]	
Chile	1	78.40	4	3.43	96.15	212.28	2.47	0.71	NA[d]	-3.5	1	12.5	87.47
Peru	2	76.05	2	3.81	53.04	79.41	1.22	3.32	NA[d]	-6.8	-1	16.125	77.35
Canada	3	73.32	3	3.62	77.08	241.30	5.63	1.21	NA[d]	-2.2	0	13.25	77.79
Italy	4	71.46	2	3.81	74.52	165.01	2.13	1.18	NA[d]	-0.6	-1	12.625	72.78
Malaysia	5	71.20	3	3.62	77.25	689.36	10.06	1.14	NA[d]	-0.4	-1	17.125	70.73
Brazil	6	69.91	4	3.24	65.34	178.63	1.78	2.89	NA[d]	-2.2	0	6	76.17
Taiwan	7	69.69	4	3.43	52.13	0.79	1.17	2.65	NA[d]	0.07	0	14.375	72.69
Sweden	8	69.67	4	2.67	71.98	183.32	1.62	0.86	NA[d]	-0.2	0	7.625	75.25
Hong Kong	9	69.41	2	3.62	59.87	1.71	1.06	0.00	NA[d]	-2.1	0	4.75	76.00
Colombia	10	69.33	4	3.62	46.23	87.99	0.91	2.18	NA[d]	-2.9	2	14.5	77.90
South Korea	11	69.11	2	3.81	76.56	102.55	16.77	0.69	NA[d]	-1	0	11.125	73.19
Poland	12	68.92	4	3.24	51.68	312.66	4.07	1.14	NA[d]	-0.6	0	10.875	73.04
Mexico	13	67.24	4	3.43	48.49	109.77	3.90	7.59	NA[d]	-0.8	1	37.5	63.60
Austria	14	65.60	3	3.62	64.17	971.86	11.83	0.40	NA[d]	-2.2	0	5.5	70.82
Japan	15	64.58	2	2.86	75.10	13.70	1.01	3.56	NA[d]	-0.4	0	14.25	66.17
Russia	16	64.01	3	3.62	35.80	669.50	13.20	3.14	NA[d]	-2	-1	12.375	63.30
Turkey	17	63.68	3	3.24	68.37	1033.50	12.29	0.75	NA[d]	-1.7	0	6.25	68.06
Australia	18	63.33	3	3.62	69.03	251.47	60.50	0.67	NA[d]	-1.8	1	9.375	69.28
Denmark	19	62.91	1	3.62	78.75	498.63	8.08	0.20	NA[d]	-1.5	0	0.75	69.18
Finland	20	62.76	1	3.24	74.01	287.99	11.29	0.49	NA[d]	-1.4	0	4.5	67.55
Netherlands	21	62.41	1	3.62	70.12	752.42	6.44	0.19	NA[d]	-1.4	0	7.5	65.96
Hungary	22	60.28	1	3.62	66.14	422.60	4.95	1.32	NA[d]	-0.5	0	4.25	64.46
Germany	23	59.00	1	3.62	66.84	438.84	8.68	0.55	NA[d]	1.3	0	6	62.15

Czech Republic	24	57.69	1	3.62	57.27	665.88	4.22	0.41	NA[d]	-0.3	0	0.375	62.60
United Kingdom	25	57.39	1	3.62	76.10	1835.63	16.01	0.32	NA[d]	-0.7	-1	9.625	55.84
United States	26	52.44	1	3.43	64.36	752.68	5.76	1.91	NA[d]	-2.3	4	11.625	63.00
Argentina	NA[d]	NA[d]	4	3.43	65.84	69.96	0.61	2.42	NA[d]	NA[d]	0	4.375	NA[d]
Belgium	NA[d]	NA[d]	1	3.62	75.63	994.61	9.29	0.34	NA[d]	0.5	NA[d]	NA[d]	60.96
China	NA[d]	NA[d]	2	3.62	78.73	0.07	1.12	0.00	NA[d]	-0.4	NA[d]	NA[d]	75.92
Egypt	NA[d]	NA[d]	4	3.05	13.34	25.41	8.70	4.98	NA[d]	NA[d]	0	14.375	NA[d]
France	NA[d]	NA[d]	3	3.43	73.85	227.81	2.18	0.64	NA[d]	NA[d]	-1	5.5	NA[d]
Greece	NA[d]	NA[d]	3	3.62	61.19	833.14	13.18	1.28	NA[d]	-3.1	0	NA[d]	71.23
India	NA[d]	NA[d]	2	3.62	38.13	37.25	1.54	1.95	NA[d]	NA[d]	0	15.75	NA[d]
Indonesia	NA[d]	NA[d]	2	3.05	34.98	10.59	0.69	5.01	NA[d]	NA[d]	3	20.875	NA[d]
Ireland	NA[d]	NA[d]	1	3.62	75.86	1117.06	14.27	0.34	NA[d]	-1.2	NA[d]	NA[d]	62.38
Israel	NA[d]	NA[d]	3	3.43	85.88	486.92	3.53	0.75	NA[d]	0.2	2	NA[d]	73.47
New Zealand	NA[d]	NA[d]	2	3.62	67.10	44.96	53.67	0.04	NA[d]	NA[d]	0	13.5	NA[d]
Norway	NA[d]	NA[d]	1	3.24	73.72	326.57	9.42	0.22	NA[d]	NA[d]	0	NA[d]	NA[d]
Pakistan	NA[d]	NA[d]	4	3.81	22.98	12.00	2.17	2.48	NA[d]	NA[d]	NA[d]	NA[d]	NA[d]
Philippines	NA[d]	NA[d]	3	4.00	26.71	213.70	9.31	2.06	NA[d]	-1.3	NA[d]	19.875	67.97
Portugal	NA[d]	NA[d]	1	3.62	79.77	210.19	2.00	0.85	NA[d]	NA[d]	0	4	NA[d]
Qatar	NA[d]	NA[d]	2	3.62	82.49	88.86	1.10	0.15	NA[d]	NA[d]	0	0	NA[d]
Saudi Arabia	NA[d]	NA[d]	2	3.81	64.69	4.20	0.27	5.26	NA[d]	NA[d]	0	10.25	NA[d]
Singapore	NA[d]	NA[d]	3	4.00	86.97	1867.63	105.52	0.31	NA[d]	NA[d]	0	10.125	NA[d]
South Africa	NA[d]	NA[d]	4	3.81	18.64	32.38	0.67	7.98	NA[d]	NA[d]	0	7.5	NA[d]
Spain	NA[d]	NA[d]	4	3.62	77.08	111.36	1.05	1.83	NA[d]	NA[d]	1	27.875	NA[d]
Switzerland	NA[d]	NA[d]	3	3.62	63.82	369.40	3.83	0.46	NA[d]	-0.7	0	NA[d]	73.70
Thailand	NA[d]	NA[d]	3	3.43	53.89	441.09	19.24	0.80	NA[d]	NA[d]	-1	5.125	NA[d]
United Arab	NA[d]	NA[d]	4	3.62	105.72	39.16	0.53	1.00	NA[d]	NA[d]	0	7.75	NA[d]

Emirates													
Vietnam	NA[d]	NA[d]	2	3.81	41.73	132.80	16.49	2.13	NA[d]	NA[d]	0	6	NA[d]

Notes:

a. Google Trends include indicators as insomnia and health literacy

b. Increase in Google searches for “insomnia”.

c. Google Trends number for “wash hands” and “face mask”.

d. NA: not applicable. Indicates a country not ranked due to incomplete data.

Supplementary Table S33. **Complete ranking and raw scores of indicators in 50 countries and territories in November 2021**

			Concern indicators										
			Government policy			Vital health and socioeconomic measures						Hygiene education	
Country	Rank	Performance score (with Google Trends)[a]	Lockdown efficiency	Health-system policies	People covered by vaccines	One-month cases per 100,000 members of the population	Infection growth rate (%)	Fatality rate (%)	GDP loss (%)	Unemployment rate (%)	Insomnia[b]	Health literacy[c]	Performance score (without Google Trends)
Italy	1	81.12	3	3.81	80.02	425.03	5.38	0.67	NA[d]	-9.6	-1	-1	87.65
Taiwan	2	74.70	4	3.62	66.12	0.79	1.15	0.53	NA[d]	-0.06	-2	-2	78.33
Malaysia	3	72.86	3	3.62	81.83	491.64	6.52	0.94	NA[d]	-4.8	0	0	80.12
Chile	4	72.13	1	3.43	106.87	352.39	3.99	0.87	NA[d]	-3.3	0	0	75.39
South Korea	5	71.64	4	3.81	83.26	167.55	23.46	0.93	NA[d]	-1.1	0	0	77.87
Peru	6	71.63	2	3.81	63.56	103.58	1.57	2.69	NA[d]	-6	1	1	76.23
Japan	7	70.34	4	2.86	78.25	3.41	0.25	2.17	NA[d]	-0.2	0	0	75.90
Mexico	8	69.86	4	3.43	50.91	59.38	2.03	7.22	NA[d]	-0.7	0	0	67.60
Sweden	9	69.10	4	2.67	78.22	328.21	2.85	0.38	NA[d]	-0.2	0	0	75.63
Canada	10	69.00	2	3.62	79.74	203.22	4.49	0.93	NA[d]	-2.5	0	0	74.69
Colombia	11	66.65	2	3.62	56.10	131.19	1.34	1.85	NA[d]	-2.5	1	1	70.68
Australia	12	64.72	2	3.81	76.16	153.66	23.03	0.68	NA[d]	-2.2	0	0	71.49
Hong Kong	13	64.60	2	3.62	62.61	1.19	0.73	0.00	NA[d]	-2.2	1	1	74.18
Russia	14	61.88	3	3.62	43.46	747.48	13.02	3.27	NA[d]	-1.8	0	0	65.03
Germany	15	58.94	3	3.62	73.89	1568.06	28.55	0.46	NA[d]	1.2	-1	-1	63.21
Netherlands	16	56.88	3	3.81	72.12	2976.85	23.93	0.20	NA[d]	-1.3	0	0	62.56
Finland	17	56.69	1	3.24	77.53	522.80	18.41	0.61	NA[d]	-0.9	0	0	63.86
United States	18	55.53	1	3.62	70.47	766.37	5.55	1.34	NA[d]	-2.5	5	5	66.74
Poland	19	54.86	1	3.43	55.13	1362.05	17.02	1.28	NA[d]	-0.7	0	0	56.86
Austria	20	54.17	3	3.62	77.56	3638.30	39.59	0.34	NA[d]	-2.5	0	0	58.67
Hungary	21	48.26	1	3.43	74.12	2487.91	27.76	1.58	NA[d]	-0.8	0	0	53.25
Czech Republic	22	44.96	1	3.62	63.12	3609.85	21.96	0.60	NA[d]	-0.5	0	0	49.64
Argentina	NA[d]	NA[d]	4	3.62	75.10	91.96	0.79	1.52	NA[d]	NA[d]	0	0	NA[d]

Belgium	NA[d]	NA[d]	3	3.62	81.25	3484.98	29.79	0.25	NA[d]	NA[d]	NA[d]	NA[d]	NA[d]
Brazil	NA[d]	NA[d]	2	3.43	72.31	133.42	1.31	2.42	NA[d]	NA[d]	0	0	NA[d]
China	NA[d]	NA[d]	2	3.62	86.83	0.11	1.63	0.00	NA[d]	-0.2	NA[d]	NA[d]	75.22
Denmark	NA[d]	NA[d]	1	3.43	84.53	1713.62	25.69	0.18	NA[d]	NA[d]	0	0	NA[d]
Egypt	NA[d]	NA[d]	4	2.86	19.49	26.44	8.33	6.61	NA[d]	NA[d]	0	0	NA[d]
France	NA[d]	NA[d]	3	3.43	78.74	754.58	7.05	0.29	NA[d]	NA[d]	0	0	NA[d]
Greece	NA[d]	NA[d]	3	3.62	69.01	1897.00	26.51	1.13	NA[d]	NA[d]	0	0	NA[d]
India	NA[d]	NA[d]	4	3.81	44.39	22.32	0.91	3.48	NA[d]	NA[d]	0	0	NA[d]
Indonesia	NA[d]	NA[d]	2	3.05	42.13	4.36	0.28	3.53	NA[d]	NA[d]	4	4	NA[d]
Ireland	NA[d]	NA[d]	1	3.62	83.26	2498.96	27.94	0.17	NA[d]	-1	NA[d]	NA[d]	57.28
Israel	NA[d]	NA[d]	2	3.43	87.28	174.38	1.22	0.59	NA[d]	-0.3	1	1	73.42
New Zealand	NA[d]	NA[d]	2	3.62	74.01	100.12	77.78	0.31	NA[d]	NA[d]	0	0	NA[d]
Norway	NA[d]	NA[d]	1	3.62	79.49	1108.07	29.22	0.25	NA[d]	NA[d]	0	0	NA[d]
Pakistan	NA[d]	NA[d]	4	3.81	27.32	5.19	0.92	2.40	NA[d]	NA[d]	NA[d]	NA[d]	NA[d]
Philippines	NA[d]	NA[d]	4	3.81	38.91	40.94	1.63	11.82	NA[d]	NA[d]	NA[d]	NA[d]	NA[d]
Portugal	NA[d]	NA[d]	1	3.62	86.40	556.63	5.19	0.50	NA[d]	NA[d]	0	0	NA[d]
Qatar	NA[d]	NA[d]	2	3.62	84.96	143.32	1.76	0.02	NA[d]	NA[d]	0	0	NA[d]
Saudi Arabia	NA[d]	NA[d]	2	3.62	67.12	3.21	0.21	3.70	NA[d]	NA[d]	1	1	NA[d]
Singapore	NA[d]	NA[d]	1	4.00	86.97	1216.65	33.45	0.47	NA[d]	NA[d]	0	0	NA[d]
South Africa	NA[d]	NA[d]	4	3.81	21.33	45.55	1.57	2.44	NA[d]	NA[d]	0	0	NA[d]
Spain	NA[d]	NA[d]	4	3.62	82.02	327.38	3.05	0.45	NA[d]	NA[d]	0	0	NA[d]
Switzerland	NA[d]	NA[d]	3	3.62	68.67	1630.48	16.29	0.20	NA[d]	-0.8	1	1	67.36
Thailand	NA[d]	NA[d]	3	3.81	66.02	291.42	10.66	0.77	NA[d]	NA[d]	-4	-4	NA[d]
Turkey	NA[d]	NA[d]	3	3.24	70.78	899.08	9.52	0.81	NA[d]	NA[d]	-1	-1	NA[d]
United Arab Emirates	NA[d]	NA[d]	4	3.62	109.33	21.38	0.29	0.51	NA[d]	NA[d]	0	0	NA[d]
United Kingdom	NA[d]	NA[d]	1	3.62	85.04	1724.53	12.97	0.37	NA[d]	NA[d]	0	0	NA[d]

Vietnam	NA[d]	NA[d]	3	3.81	62.18	322.87	34.41	1.00	NA[d]	NA[d]	0	0	NA[d]

Notes:

a. Google Trends include indicators as insomnia and health literacy

b. Increase in Google searches for “insomnia”.

c. Google Trends number for “wash hands” and “face mask”.

d. NA: not applicable. Indicates a country not ranked due to incomplete data.

Supplementary Information 13. Sensitivity analysis

In our study, we assess the national performance in response to COVID-19 and focus on "government policy & hygiene education" and the "vital health & socioeconomic" measures. Government policy & hygiene education includes (1) lockdown efficiency, (2) health-system policies, and (3) health literacy. The vital health & socioeconomic measures include (1) one-month cases per 100,000 members of the population, (2) the infection growth rate, (3) the fatality rate, (4) GDP loss due to the pandemic, (5) the unemployment rate, and (6) insomnia. In our analysis, we use Google Trends to obtain searching trends, including "wash hands" and "face mask" as surrogates for health literacy, and "insomnia" as a surrogate for the impact of the pandemic on mental health. We convert the performance score to a scale of 0–100, with 0 being the worst performance, and rank the 50 countries and territories based on their performance each month. In this section, we conduct sensitivity analysis of COVID-19 pandemic containment performance for the 50 selected countries and territories.

Supplementary Table S34 lists the countries and places included in the analysis and Supplementary Table S35 demonstrates the nine scenarios evaluated. Because there is yet any data of the GDP annual growth rate from October to November 2020 at the time of the analysis for the 50 countries and territories, the indicator of GDP loss from October to November 2020 is not included in the analysis. Similary, for the unemployment rate from March to November 2020, complete data was not available from several countries, such as Argentina, Belgium, Brazil, Egypt, France, Greece, Indonesia, New Zealand, Norway, Pakistan, Portugal, Qatar, Saudi Arabia, Singapore, South Africa, Spain, Thailand, Turkey, the United Arab Emirates, the United Kingdom, and Vietnam.

(1) Sensitivity analysis by changing the indicators' calculations.

We alter the calculation method of several parameters to conduct sensitivity analysis. The results of the sensitivity analyses are shown in Supplementary Tables S36–S40.

Supplementary Table S36 shows the rankings of 50 countries and territories from March 2020 to November 2020 that exclude indicators of health literacy and insomnia. We change the calculation method of the health-system policies indicator from calculating the highest frequency in each month to calculating the average of each month (one-way sensitivity analysis). Supplementary Table S37 shows the ranking of 50 places from March 2020 to November 2020 by a three-month moving average that excludes indicators of health literacy and insomnia. By using the moving average, we can smooth score data and create a constantly updating average score for the three-month period. We change the calculation method of the health-system policies indicator to calculate the average of each month (two-way sensitivity analysis). The results show that the ranking of Supplementary Table S36 is similar to the ranking of Supplementary Table S37. The leading nations are mostly based in Asia and Oceania, including Taiwan, Vietnam, Korea, Thailand, and New Zealand; the countries whose performance needs to be improved are mostly located in America and Europe, including the United States, the United Kingdom, Canada, Colombia, Peru, and Sweden.

Supplementary Tables S38 and S39 show the rankings in 50 countries from March 2020 to October 2020 that exclude indicators of health literacy and insomnia. We change the calculation method of lockdown efficiency, one-month cases per 100,000 members of the population, and the fatality rate. The identification for lockdown efficiency score changes from comparing the average stringency index of the last two weeks of the month with the total incidence for the first two weeks of the month to comparing the average stringency index with the total incidence for the last two weeks of the month. For the sensitivity analysis, the fatality rate is calculated by dividing cumulative fatalities by cumulative infections. We replace the indicator of one-month cases per 100,000 members of the population in Supplementary Table S38 to be calculated by the cumulative number of diagnosed COVID-19 cases instead. The comparison between Supplementary Tables S38 and S39 shows that Taiwan is among the top ranking from March 2020 to October 2020 and that most Asian countries are still in the lead.

Supplementary Table S40 shows the results of the ranking of 50 countries and territories from March 2020 to October 2020 excluding the indicators health literacy, insomnia, and the unemployment rate. In Supplementary Table S40, we change the calculation method for both indicators of lockdown efficiency and the fatality rate. For the purpose of sensitivity analysis, the lockdown efficiency is redefined as the

ratio of the total incidence against the average stringency index for the last two weeks of the month. The indicator of the fatality rate is also modified as calculating the ratio of cumulative deaths to cumulative infections. As some countries do not have unemployment rate data in certain months, for the sensitivity analysis, we excluded the unemployment rate indicator to confirm whether the unemployment rate indicator will affect the ranking. The comparison between Supplementary Tables S39 and S40 shows that the leading nations are still mostly located in Asia and Oceania, including Taiwan, Vietnam, Korea, Malaysia, Singapore, Australia, and New Zealand.

(2) Sensitivity analysis of the inclusion or exclusion of Google Trends data.

To conduct sensitivity analysis between including and excluding Google Trends data, we create Supplementary Tables S41, S42, S43 and S44 and compared those with Supplementary Tables S36, S37, S38, and S39 respectively (i.e., Supplementary Table S41 is compared to Supplementary Table S36; Supplementary Table S42 is compared to Supplementary Table S37; Supplementary Table S43 is compared to Supplementary Table S38; Supplementary Table S44 is compared to Supplementary Table S39).

Supplementary Table S41 shows the rankings of the 50 countries and territories from March 2020 to November 2020. This analysis included indicators of health literacy and insomnia. We change the calculation method of the health-system policies indicator from calculating the frequency to calculating the average of each month.

Supplementary Table S42 shows the rankings of 50 countries and places from March 2020 to November 2020 by three-month moving average. This analysis includes indicators of health literacy and insomnia. We change the calculation method of the health-system policies indicator by calculating the three-month moving average of the indicator instead.

Supplementary Table S43 shows the rankings of 50 countries and territories from March 2020 to October 2020. We change the calculation method for lockdown efficiency, one-month cases per 100,000 members of the population and fatality rate. The identification of lockdown efficiency is changed as comparing the incidence with the stringency index for the last two weeks of the month. Indicators of one-month cases per 100,000 members of the population and fatality rate are changed as calculating the cumulative number of COVID-19 cases and the ratio of cumulative deaths to cumulative infections, respectively.

Supplementary Table S44 shows the rankings of 50 countries and places from March 2020 to October 2020. We alter the calculation method of both lockdown efficiency and fatality rate. The identification of lockdown efficiency is altered as comparing the incidence and the stringency index for the last two weeks of the month. Indicator of fatality rate is redefined as calculating the ratio of cumulative deaths to cumulative infections.

The results of including or excluding Google Trends indicators indicate that the overall rankings of certain countries and regions have slightly changed, but Taiwan and Korea in Asia and New Zealand in Oceania are still the leading countries and regions.

Supplementary Table S45 and Supplementary Table S46 list the alternative calculation methods for the ten indicators we mentioned in Supplementary Information 7. Supplementary Table S45 includes Google Trends indicators, that is, health literacy and insomnia indicators, while Supplementary Table S46 does not include Google Trends indicators. The lockdown efficiency is identified by comparing the average stringency index of the last two weeks of the month with the total incidence for the first two weeks of the month. The calculation method of health system policy indicators is to calculate the highest frequency in each month. The one-month cases per 100,000 members of the population is the number of COVID-19 cases per 100,000 members of a population in the past month. The fatality rate is the ratio of one-month cumulative deaths to one-month cumulative infections. Please refer to Table 1 for the definition of each indicator.

Supplementary Table S34. **List of 50 countries and territories included in the analysis**

**Country**	**Start Date**	**End Date**	**Remarks**
Argentina	31/03/2020	30/11/2020	no unemployment rate data from October to November
Australia	31/03/2020	30/11/2020	
Austria	31/03/2020	30/11/2020	
Belgium	31/03/2020	30/11/2020	no unemployment rate data for November
Brazil	31/03/2020	30/11/2020	no unemployment rate data for November
Canada	31/03/2020	30/11/2020	
Chile	31/03/2020	30/11/2020	
China	31/03/2020	30/11/2020	google trend data is not available
Colombia	31/03/2020	30/11/2020	
Czech Republic	31/03/2020	30/11/2020	
Denmark	31/03/2020	30/11/2020	
Egypt	31/03/2020	30/11/2020	no unemployment rate data from October to November
Finland	31/03/2020	30/11/2020	
France	31/03/2020	30/11/2020	no unemployment rate data from October to November
Germany	31/03/2020	30/11/2020	
Greece	31/03/2020	30/11/2020	no unemployment rate data for November
Hong Kong	31/03/2020	30/11/2020	
Hungary	31/03/2020	30/11/2020	
India	31/03/2020	30/11/2020	
Indonesia	31/03/2020	30/11/2020	no unemployment rate data from July to November
Ireland	31/03/2020	30/11/2020	
Israel	31/03/2020	30/11/2020	
Italy	31/03/2020	30/11/2020	
Japan	31/03/2020	30/11/2020	
Malaysia	31/03/2020	30/11/2020	
Mexico	31/03/2020	30/11/2020	
Netherlands	31/03/2020	30/11/2020	
New Zealand	31/03/2020	30/11/2020	no unemployment rate data from October to November
Norway	31/03/2020	30/11/2020	no unemployment rate data for November
Pakistan	31/03/2020	30/11/2020	no data
Peru	31/03/2020	30/11/2020	
Philippines	31/03/2020	30/11/2020	
Poland	31/03/2020	30/11/2020	
Portugal	31/03/2020	30/11/2020	no unemployment rate data from October to November
Qatar	31/03/2020	30/11/2020	no unemployment rate data from April to November
Russia	31/03/2020	30/11/2020	
Saudi Arabia	31/03/2020	30/11/2020	no unemployment rate data from July to November
Singapore	31/03/2020	30/11/2020	no unemployment rate data from October to November
South Africa	31/03/2020	30/11/2020	no unemployment rate data from October to November
Korea	31/03/2020	30/11/2020	
Spain	31/03/2020	30/11/2020	no unemployment rate data from October to November
Sweden	31/03/2020	30/11/2020	
Switzerland	31/03/2020	30/11/2020	
Taiwan	31/03/2020	30/11/2020	
Thailand	31/03/2020	30/11/2020	no unemployment rate data from April to June

Turkey	31/03/2020	30/11/2020	no unemployment rate data for November
United Arab Emirates	31/03/2020	30/11/2020	no data
United Kingdom	31/03/2020	30/11/2020	no unemployment rate data for November
United States	31/03/2020	30/11/2020	
Vietnam	31/03/2020	30/11/2020	no unemployment rate data from July to November

There are no data of the GDP annual growth rate from October to November in the 50 counties, so the indicator of GDP loss from October to November is not included in the analysis.

Supplementary Table S35: **Summary of sensitivity analysis for COVID-19 pandemic performance**

**Concern indicators**	**Policy and hygiene education**			**Vital outcomes**						**Remarks**
	Lockdown efficiency	Health-system policies	Health literacy	One-month cases per 100,000 members of the population	Infection growth rate (%)	Fatality rate (%)	GDP loss (%)	Unemployment rate (%)	Insomnia	
Supplementary Table S36		a monthly average	excluded						excluded	
Supplementary Table S37		a monthly average	excluded						excluded	three-month moving average
Supplementary Table S38	The comparison between incidence and stringency index for the last two weeks of the month		excluded	cumulative number of COVID-19 cases		ratio of cumulative deaths to cumulative infections			excluded	
Supplementary Table S39	The comparison between incidence and stringency index for the last two weeks of the month		excluded			ratio of cumulative deaths to cumulative infections			excluded	

Supplementary Table S40	The comparison between incidence and stringency index for the last two weeks of the month		excluded			ratio of cumulative deaths to cumulative infections		excluded	excluded	
Supplementary Table S41		a monthly average								
Supplementary Table S42		a monthly average								three-month moving average
Supplementary Table S43	The comparison between incidence and stringency index for the last two weeks of the month			cumulative number of COVID-19 cases		ratio of cumulative deaths to cumulative infections				
Supplementary Table S44	The comparison between incidence and stringency index for the last two weeks of the month					ratio of cumulative deaths to cumulative infections				

Supplementary Table S36. **Rankings of 50 countries and territories March–November 2020, excluding indicators of health literacy and insomnia (one-way sensitivity analysis)**

Rank	Mar	Apr	May	Jun	Jul	Aug	Sep	Oct	Nov
1	Egypt	Vietnam	Vietnam	Taiwan	Taiwan	Taiwan	Taiwan	Taiwan	Taiwan
2	Taiwan	Taiwan	Taiwan	Korea	Korea	Korea	Korea	Thailand	Thailand
3	Vietnam	Korea	Australia	Vietnam	New Zealand	Turkey	Italy	China	China
4	Qatar	Hong Kong	China	New Zealand	China	Thailand	Singapore	Korea	Australia
5	Japan	Australia	Korea	China	Ireland	Ireland	New Zealand	Hong Kong	Korea
6	Norway	China	Hong Kong	Australia	Italy	New Zealand	China	Japan	Japan
7	Turkey	Israel	Norway	Finland	Norway	Italy	Germany	Norway	Israel
8	South Africa	New Zealand	Greece	Malaysia	Malaysia	Finland	Thailand	India	India
9	Korea	Malaysia	New Zealand	Switzerland	Thailand	China	Norway	Finland	Hong Kong
10	Russia	Japan	Turkey	Norway	Finland	Malaysia	Japan	Australia	Finland
11	Hong Kong	Norway	Japan	Japan	Netherlands	Norway	Ireland	Turkey	Ireland
12	Poland	South Africa	Switzerland	Turkey	Germany	Poland	Turkey	Israel	Malaysia
13	Greece	Finland	Czech Republic	Greece	Greece	United Kingdom	Finland	Denmark	Denmark
14	Mexico	Greece	Israel	Denmark	France	Japan	Malaysia	Italy	Mexico
15	Thailand	Indonesia	Germany	Italy	Switzerland	Greece	Poland	Canada	Peru
16	Indonesia	Turkey	South Africa	Czech Republic	Poland	Switzerland	Australia	Mexico	Germany
17	Singapore	Czech Republic	Austria	Hong Kong	Denmark	Denmark	South Africa	Netherlands	Chile
18	Saudi Arabia	Brazil	Indonesia	Hungary	Turkey	Singapore	Hong Kong	United Kingdom	Italy
19	Colombia	Portugal	Singapore	Ireland	Japan	Hong Kong	Greece	Malaysia	Netherlands

20	Denmark	Singapore	Malaysia	Israel	Czech Republic	Austria	Switzerland	Ireland	Austria
21	Malaysia	Ireland	Russia	France	Hungary	Germany	France	Brazil	Philippines
22	Australia	Russia	Ireland	Austria	Belgium	Netherlands	Portugal	Austria	Poland
23	Hungary	Saudi Arabia	Belgium	Germany	United Kingdom	France	Israel	Belgium	Hungary
24	Sweden	Italy	Poland	Netherlands	Sweden	Hungary	Netherlands	Czech Republic	Colombia
25	Israel	Egypt	Saudi Arabia	Portugal	Portugal	Portugal	United Kingdom	Germany	Czech Republic
26	Netherlands	Hungary	Egypt	Spain	Hong Kong	Belgium	India	Peru	Russia
27	Brazil	Poland	France	Russia	India	Sweden	Denmark	Philippines	Switzerland
28	Czech Republic	Belgium	Hungary	Saudi Arabia	Singapore	Canada	Russia	Switzerland	United States
29	Italy	Philippines	Denmark	Singapore	Russia	Australia	Belgium	Sweden	Sweden
30	Chile	Germany	Brazil	South Africa	United States	India	United States	Chile	Canada
31	China	India	Finland	Belgium	Israel	South Africa	Sweden	Hungary	Argentina
32	New Zealand	France	Netherlands	United Kingdom	Spain	Russia	Canada	Colombia	Belgium
33	Argentina	Denmark	Spain	Brazil	Austria	United States	Mexico	United States	Brazil
34	Finland	Switzerland	Italy	Egypt	Australia	Czech Republic	Brazil	Russia	Egypt
35	Peru	Peru	Philippines	United States	Canada	Israel	Austria	Poland	France
36	France	Chile	Chile	Indonesia	South Africa	Mexico	Philippines	Argentina	Greece
37	India	Argentina	Mexico	Poland	Brazil	Brazil	Peru	Egypt	Indonesia
38	Belgium	Colombia	Portugal	Canada	Philippines	Spain	Czech Republic	France	New Zealand
39	Switzerland	Austria	India	Mexico	Argentina	Philippines	Hungary	Greece	Norway
40	Philippines	Netherlands	Colombia	India	Mexico	Argentina	Chile	Indonesia	Pakistan
41	Ireland	Mexico	Argentina	Philippines	Peru	Chile	Spain	New Zealand	Portugal
42	Austria	Spain	Peru	Argentina	Chile	Colombia	Argentina	Pakistan	Qatar

43	Spain	Canada	United States	Chile	Colombia	Peru	Colombia	Portugal	Saudi Arabia
44	Portugal	United States	United Kingdom	Colombia	Egypt	Egypt	Egypt	Qatar	Singapore
45	Germany	Sweden	Sweden	Sweden	Indonesia	Indonesia	Indonesia	Saudi Arabia	South Africa
46	United Kingdom	United Kingdom	Canada	Peru	Pakistan	Pakistan	Pakistan	Singapore	Spain
47	Canada	Pakistan	Pakistan	Pakistan	Qatar	Qatar	Qatar	South Africa	Turkey
48	United States	Qatar	Qatar	Qatar	Saudi Arabia	Saudi Arabia	Saudi Arabia	Spain	United Arab Emirates
49	Pakistan	Thailand	Thailand	Thailand	United Arab Emirates	United Arab Emirates	United Arab Emirates	United Arab Emirates	United Kingdom
50	United Arab Emirates	United Arab Emirates	United Arab Emirates	United Arab Emirates	Vietnam	Vietnam	Vietnam	Vietnam	Vietnam

Gray shading indicates the relevant data and indicators of the countries are not ranked due to incomplete information.

***Supplementary Table S37*. Rankings of 50 countries and territories March–November 2020 by a three-month moving average, excluding indicators of health literacy and insomnia (two-way sensitivity analysis)**

Rank	Mar	Apr	May	Jun	Jul	Aug	Sep	Oct	Nov
1	Egypt	Vietnam	Vietnam	Taiwan	Taiwan	Taiwan	Taiwan	Taiwan	Taiwan
2	Taiwan	Taiwan	Taiwan	Vietnam	Korea	Korea	Korea	Korea	Thailand
3	Vietnam	Korea	Korea	Korea	China	New Zealand	New Zealand	Thailand	China
4	Qatar	Hong Kong	Hong Kong	China	New Zealand	China	China	China	Korea
5	Japan	Japan	Australia	Australia	Norway	Finland	Italy	Norway	Japan
6	Norway	Norway	China	New Zealand	Greece	Malaysia	Thailand	Italy	Australia
7	Turkey	South Africa	Japan	Hong Kong	Malaysia	Italy	Ireland	Japan	Hong Kong
8	South Africa	Australia	Norway	Norway	Australia	Norway	Norway	Turkey	Finland
9	Korea	Egypt	Turkey	Japan	Switzerland	Ireland	Finland	Finland	India
10	Russia	Israel	Greece	Malaysia	Turkey	Turkey	Malaysia	Hong Kong	Israel
11	Hong Kong	Malaysia	South Africa	Greece	Japan	Greece	Turkey	Ireland	Italy
12	Poland	Turkey	New Zealand	Israel	Finland	Switzerland	Germany	Malaysia	Ireland
13	Greece	China	Israel	Turkey	Ireland	Japan	Poland	Australia	Malaysia
14	Mexico	Greece	Malaysia	Czech Republic	Germany	Denmark	Japan	Germany	Germany
15	Thailand	New Zealand	Indonesia	Finland	Italy	Netherlands	Greece	United Kingdom	Denmark
16	Indonesia	Indonesia	Egypt	Switzerland	Hong Kong	Germany	Singapore	Poland	Mexico
17	Singapore	Russia	Czech Republic	South Africa	Czech Republic	France	Netherlands	Denmark	Netherlands
18	Saudi Arabia	Singapore	Russia	Ireland	Denmark	Hungary	Switzerland	Switzerland	Poland
19	Colombia	Poland	Singapore	Germany	Netherlands	Australia	France	India	Switzerland

20	Denmark	Saudi Arabia	Poland	Singapore	France	Poland	Denmark	Netherlands	Austria
21	Malaysia	Finland	Saudi Arabia	Indonesia	Hungary	Hong Kong	Hong Kong	Israel	Peru
22	Australia	Czech Republic	Finland	Hungary	Israel	United Kingdom	United Kingdom	Canada	Philippines
23	Hungary	Brazil	Brazil	Italy	Belgium	Czech Republic	Portugal	Austria	Canada
24	Sweden	Hungary	Switzerland	Denmark	Singapore	Portugal	Belgium	Belgium	Russia
25	Israel	Denmark	Hungary	Russia	Austria	Singapore	Australia	Sweden	Sweden
26	Netherlands	Italy	Denmark	Austria	Poland	Belgium	Sweden	Mexico	Chile
27	Brazil	Mexico	Ireland	France	Russia	Austria	Hungary	Hungary	Czech Republic
28	Czech Republic	Colombia	Belgium	Saudi Arabia	South Africa	Israel	South Africa	Brazil	Hungary
29	Italy	Chile	Germany	Belgium	Portugal	Russia	Austria	Russia	United States
30	Chile	Ireland	Italy	Brazil	Spain	Spain	India	United States	Colombia
31	China	Belgium	Mexico	Portugal	Brazil	South Africa	Czech Republic	Czech Republic	Argentina
32	New Zealand	Netherlands	France	Egypt	United Kingdom	United States	Israel	Philippines	Belgium
33	Argentina	Argentina	Austria	Poland	India	Canada	Canada	Chile	Brazil
34	Finland	Peru	Colombia	Netherlands	United States	Sweden	Russia	Peru	Egypt
35	Peru	India	Netherlands	Spain	Philippines	India	United States	Colombia	France
36	France	France	Chile	Philippines	Mexico	Brazil	Brazil	Argentina	Greece
37	India	Portugal	Portugal	India	Sweden	Mexico	Mexico	Egypt	Indonesia
38	Belgium	Philippines	India	Chile	Canada	Philippines	Spain	France	New Zealand
39	Switzerland	Switzerland	Philippines	Mexico	Argentina	Argentina	Philippines	Greece	Norway
40	Philippines	Sweden	Argentina	Argentina	Chile	Chile	Argentina	Indonesia	Pakistan
41	Ireland	Germany	Peru	Colombia	Colombia	Colombia	Chile	New Zealand	Portugal
42	Austria	Austria	Spain	United States	Peru	Peru	Peru	Pakistan	Qatar

43	Spain	Spain	Sweden	Peru	Egypt	Egypt	Colombia	Portugal	Saudi Arabia
44	Portugal	Canada	United Kingdom	United Kingdom	Indonesia	Indonesia	Egypt	Qatar	Singapore
45	Germany	United Kingdom	United States	Canada	Pakistan	Pakistan	Indonesia	Saudi Arabia	South Africa
46	United Kingdom	United States	Canada	Sweden	Qatar	Qatar	Pakistan	Singapore	Spain
47	Canada	Pakistan	Pakistan	Pakistan	Saudi Arabia	Saudi Arabia	Qatar	South Africa	Turkey
48	United States	Qatar	Qatar	Qatar	Thailand	Thailand	Saudi Arabia	Spain	United Arab Emirates
49	Pakistan	Thailand	Thailand	Thailand	United Arab Emirates	United Arab Emirates	United Arab Emirates	United Arab Emirates	United Kingdom
50	United Arab Emirates	United Arab Emirates	United Arab Emirates	United Arab Emirates	Vietnam	Vietnam	Vietnam	Vietnam	Vietnam

Gray shading indicates the relevant data and indicators of the countries are not ranked due to incomplete information.

Supplementary Table S38. **Rankings of 50 countries and territories March–October 2020, excluding indicators of health literacy and insomnia (three-way sensitivity analysis)**

Rank	Mar	Apr	May	Jun	Jul	Aug	Sep	Oct
1	Taiwan	Taiwan	Vietnam	Taiwan	Taiwan	Taiwan	Taiwan	Taiwan
2	Vietnam	Vietnam	Taiwan	Korea	Korea	Turkey	Korea	Korea
3	Egypt	Korea	Korea	Vietnam	Turkey	Korea	Malaysia	Japan
4	Qatar	Australia	Australia	Australia	Malaysia	Malaysia	Japan	India
5	Korea	China	New Zealand	New Zealand	Thailand	Thailand	China	Hong Kong
6	Mexico	Israel	Turkey	Malaysia	Norway	China	Hong Kong	China
7	Brazil	Norway	China	Switzerland	Italy	Norway	Norway	Israel
8	Japan	Hong Kong	Greece	Japan	China	Hong Kong	India	Australia
9	Hong Kong	Malaysia	Hong Kong	Norway	Germany	Italy	Italy	Austria
10	Norway	Japan	Israel	China	Hong Kong	Singapore	Singapore	Finland
11	Poland	New Zealand	Japan	Finland	Finland	Finland	Germany	Denmark
12	South Africa	Czech Republic	Czech Republic	Hong Kong	Denmark	Denmark	Poland	Netherlands
13	Russia	Austria	Indonesia	Turkey	Netherlands	Poland	Finland	Czech Republic
14	Indonesia	Russia	Norway	Greece	Poland	Japan	Israel	Poland
15	Singapore	Saudi Arabia	Finland	Israel	Japan	India	Portugal	Switzerland
16	Greece	South Africa	Hungary	Hungary	Russia	Germany	Russia	Hungary
17	Denmark	Turkey	Switzerland	Italy	India	Hungary	Switzerland	Mexico
18	Thailand	Greece	Russia	South Africa	Switzerland	Portugal	United States	United States
19	Saudi Arabia	Indonesia	Singapore	Netherlands	Hungary	Russia	Czech Republic	Germany

20	Czech Republic	Finland	South Africa	Denmark	Austria	Switzerland	Denmark	Russia
21	Argentina	Ireland	Malaysia	Russia	Portugal	Sweden	Mexico	Colombia
22	Australia	Hungary	Ireland	Ireland	Czech Republic	Czech Republic	France	Canada
23	Colombia	Singapore	Poland	Austria	United Kingdom	Philippines	Austria	Ireland
24	Hungary	Poland	Germany	France	France	Israel	Philippines	Peru
25	Israel	Germany	Italy	Saudi Arabia	Singapore	Netherlands	Sweden	Sweden
26	Turkey	Italy	Saudi Arabia	Singapore	Israel	France	Netherlands	Argentina
27	Netherlands	Portugal	Austria	Germany	United States	United Kingdom	United Kingdom	Belgium
28	Sweden	Brazil	Belgium	Czech Republic	Belgium	United States	Peru	Brazil
29	Malaysia	Belgium	France	Indonesia	Philippines	Austria	Chile	Chile
30	Italy	France	Brazil	Poland	Sweden	Belgium	Hungary	Egypt
31	New Zealand	Switzerland	Colombia	Portugal	Mexico	Chile	Belgium	France
32	Belgium	Netherlands	Mexico	United Kingdom	Chile	Mexico	Colombia	Greece
33	Finland	India	Portugal	Brazil	Colombia	Peru	Spain	Indonesia
34	Peru	Denmark	Netherlands	India	Peru	Colombia	Argentina	Italy
35	Chile	Argentina	Denmark	Belgium	Spain	Spain	Australia	Malaysia
36	China	Chile	Argentina	Spain	Argentina	Argentina	Brazil	New Zealand
37	India	Mexico	Chile	Colombia	Australia	Australia	Canada	Norway
38	France	Peru	Spain	United States	Brazil	Brazil	Egypt	Pakistan
39	Switzerland	Philippines	India	Argentina	Canada	Canada	Greece	Philippines
40	Germany	Spain	Philippines	Canada	Egypt	Egypt	Indonesia	Portugal
41	Ireland	Colombia	United Kingdom	Mexico	Greece	Greece	Ireland	Qatar
42	Spain	United States	United States	Chile	Indonesia	Indonesia	New Zealand	Saudi Arabia

43	Austria	United Kingdom	Peru	Peru	Ireland	Ireland	Pakistan	Singapore
44	Canada	Canada	Sweden	Philippines	New Zealand	New Zealand	Qatar	South Africa
45	Philippines	Sweden	Canada	Sweden	Pakistan	Pakistan	Saudi Arabia	Spain
46	Portugal	Egypt	Egypt	Egypt	Qatar	Qatar	South Africa	Thailand
47	United States	Pakistan	Pakistan	Pakistan	Saudi Arabia	Saudi Arabia	Thailand	Turkey
48	United Kingdom	Qatar	Qatar	Qatar	South Africa	South Africa	Turkey	United Arab Emirates
49	Pakistan	Thailand	Thailand	Thailand	United Arab Emirates	United Arab Emirates	United Arab Emirates	United Kingdom
50	United Arab Emirates	United Arab Emirates	United Arab Emirates	United Arab Emirates	Vietnam	Vietnam	Vietnam	Vietnam

Gray shading indicates the relevant data and indicators of the countries are not ranked due to incomplete information.

Supplementary Table S39. **Rankings of 50 countries and territories March–October 2020, excluding indicators of health literacy and insomnia (two-way sensitivity analysis)**

Rank	Mar	Apr	May	Jun	Jul	Aug	Sep	Oct
1	Taiwan	Taiwan	Vietnam	Taiwan	Taiwan	Taiwan	Taiwan	Taiwan
2	Vietnam	Vietnam	Taiwan	Korea	Korea	Turkey	Korea	Korea
3	Egypt	Korea	Korea	Vietnam	Italy	Korea	Malaysia	Japan
4	Korea	Australia	Australia	Australia	Turkey	Malaysia	Singapore	India
5	Qatar	China	New Zealand	New Zealand	Norway	Thailand	Japan	Hong Kong
6	Mexico	Norway	Greece	Switzerland	Malaysia	China	China	China
7	South Africa	Hong Kong	Hong Kong	Malaysia	Thailand	Norway	Italy	Israel
8	Brazil	Israel	Turkey	Japan	China	Italy	Hong Kong	Australia
9	Japan	Czech Republic	China	Norway	Germany	Singapore	Norway	Finland
10	Hong Kong	Malaysia	Israel	China	Finland	Finland	Germany	Austria
11	Norway	New Zealand	Japan	Finland	Netherlands	Hong Kong	Poland	United States
12	Poland	Japan	Czech Republic	Hong Kong	Denmark	Denmark	India	Czech Republic
13	Russia	Austria	Norway	Hungary	Poland	Poland	Finland	Denmark
14	Indonesia	South Africa	Switzerland	Italy	Hong Kong	Japan	Israel	Mexico
15	Singapore	Turkey	Germany	Turkey	Japan	Germany	Portugal	Netherlands
16	Greece	Greece	Indonesia	Ireland	Russia	Sweden	Russia	Colombia
17	Denmark	Indonesia	Malaysia	Greece	Switzerland	Hungary	Switzerland	Russia
18	Thailand	Finland	Finland	Israel	United Kingdom	Portugal	United States	Switzerland
19	Saudi Arabia	Ireland	Hungary	Netherlands	Hungary	Russia	Mexico	Hungary

20	Czech Republic	Hungary	Italy	Denmark	France	Switzerland	Czech Republic	Canada
21	Australia	Russia	Russia	France	India	United Kingdom	Denmark	Germany
22	Colombia	Saudi Arabia	Singapore	Russia	Portugal	India	Philippines	Peru
23	Hungary	Singapore	Austria	South Africa	Singapore	Czech Republic	Sweden	Poland
24	Israel	Poland	Ireland	Austria	Austria	Israel	France	Ireland
25	Turkey	Switzerland	Poland	Germany	Belgium	Netherlands	United Kingdom	Sweden
26	Netherlands	Germany	Belgium	Spain	Sweden	United States	Austria	Argentina
27	Sweden	Italy	Saudi Arabia	Saudi Arabia	Czech Republic	Philippines	Peru	Belgium
28	Malaysia	Portugal	South Africa	Singapore	Israel	Chile	Chile	Brazil
29	Italy	Brazil	France	Belgium	United States	Mexico	Netherlands	Chile
30	China	Belgium	Portugal	Portugal	Philippines	France	Belgium	Egypt
31	Argentina	Netherlands	Netherlands	United Kingdom	Chile	Austria	Hungary	France
32	New Zealand	India	Denmark	Brazil	Mexico	Belgium	Colombia	Greece
33	Belgium	Denmark	Brazil	Czech Republic	Peru	Peru	Spain	Indonesia
34	Finland	France	Mexico	Indonesia	Spain	Colombia	Argentina	Italy
35	Peru	Chile	Spain	Poland	Colombia	Spain	Australia	Malaysia
36	India	Mexico	Colombia	India	Argentina	Argentina	Brazil	New Zealand
37	France	Peru	Argentina	United States	Australia	Australia	Canada	Norway
38	Switzerland	Philippines	Chile	Canada	Brazil	Brazil	Egypt	Pakistan
39	Chile	Argentina	India	Chile	Canada	Canada	Greece	Philippines
40	Germany	Spain	Philippines	Mexico	Egypt	Egypt	Indonesia	Portugal
41	Ireland	Colombia	United Kingdom	Peru	Greece	Greece	Ireland	Qatar
42	Spain	United States	United States	Colombia	Indonesia	Indonesia	New Zealand	Saudi Arabia

43	Austria	United Kingdom	Peru	Philippines	Ireland	Ireland	Pakistan	Singapore
44	Canada	Canada	Sweden	Argentina	New Zealand	New Zealand	Qatar	South Africa
45	Philippines	Sweden	Canada	Sweden	Pakistan	Pakistan	Saudi Arabia	Spain
46	Portugal	Egypt	Egypt	Egypt	Qatar	Qatar	South Africa	Thailand
47	United States	Pakistan	Pakistan	Pakistan	Saudi Arabia	Saudi Arabia	Thailand	Turkey
48	United Kingdom	Qatar	Qatar	Qatar	South Africa	South Africa	Turkey	United Arab Emirates
49	Pakistan	Thailand	Thailand	Thailand	United Arab Emirates	United Arab Emirates	United Arab Emirates	United Kingdom
50	United Arab Emirates	United Arab Emirates	United Arab Emirates	United Arab Emirates	Vietnam	Vietnam	Vietnam	Vietnam

Gray shading indicates the relevant data and indicators of the countries are not ranked due to incomplete information.

Supplementary Table S40. **Rankings of 50 countries and territories March–October 2020, excluding indicators of health literacy, insomnia, and unemployment rate (three-way sensitivity analysis)**

Rank	Mar	Apr	May	Jun	Jul	Aug	Sep	Oct
1	Taiwan	Taiwan	Vietnam	Taiwan	Taiwan	Taiwan	Taiwan	Saudi Arabia
2	Vietnam	Vietnam	Taiwan	Vietnam	Norway	Malaysia	Korea	Singapore
3	Korea	Korea	Australia	Australia	Malaysia	Saudi Arabia	Saudi Arabia	Taiwan
4	South Africa	Australia	Korea	Korea	Korea	Turkey	Singapore	Thailand
5	Qatar	Hong Kong	Hong Kong	Malaysia	Thailand	Korea	Hong Kong	Pakistan
6	Hong Kong	China	Norway	Thailand	Italy	Thailand	Thailand	Hong Kong
7	Egypt	Thailand	Switzerland	Switzerland	Turkey	Norway	Malaysia	Qatar
8	Japan	Norway	New Zealand	Hong Kong	Germany	Hong Kong	Norway	Korea
9	Mexico	Malaysia	China	New Zealand	Finland	Singapore	Vietnam	New Zealand
10	Norway	Czech Republic	Israel	Norway	China	Finland	Turkey	South Africa
11	Russia	Austria	Japan	Finland	Hong Kong	China	Japan	Japan
12	Singapore	Israel	Czech Republic	Japan	Vietnam	Vietnam	China	Norway
13	Brazil	Japan	Germany	China	Denmark	Italy	Germany	Vietnam
14	Colombia	Indonesia	Greece	Qatar	Netherlands	Denmark	Italy	India
15	Denmark	Saudi Arabia	Indonesia	Denmark	Saudi Arabia	Poland	Poland	Israel
16	Indonesia	New Zealand	Turkey	Hungary	Poland	Germany	Finland	Australia
17	Thailand	Qatar	Austria	Turkey	Russia	Russia	India	China
18	Malaysia	Russia	Malaysia	Austria	Hungary	Sweden	Portugal	Egypt
19	Poland	Switzerland	Thailand	Israel	Japan	Portugal	United States	Philippines

20	Saudi Arabia	Germany	Finland	Netherlands	Austria	Japan	Russia	Brazil
21	China	India	Qatar	Italy	Portugal	Hungary	Israel	Malaysia
22	Czech Republic	Hungary	Russia	Russia	Switzerland	United States	Philippines	Turkey
23	Australia	South Africa	Saudi Arabia	Saudi Arabia	Sweden	Philippines	Switzerland	United States
24	Hungary	Singapore	Hungary	Ireland	France	Switzerland	Sweden	Finland
25	Greece	Poland	Singapore	Germany	United States	Chile	Peru	Indonesia
26	Argentina	Turkey	Poland	Greece	United Kingdom	Indonesia	Chile	Colombia
27	India	Greece	Italy	Spain	India	United Kingdom	Indonesia	Austria
28	New Zealand	Chile	Ireland	France	Singapore	Austria	Mexico	Portugal
29	Finland	Finland	Belgium	Singapore	Philippines	Czech Republic	Austria	United Arab Emirates
30	Switzerland	Ireland	Denmark	South Africa	Chile	Israel	Czech Republic	Czech Republic
31	Chile	Denmark	Colombia	United States	Czech Republic	Mexico	Denmark	Chile
32	Israel	Philippines	South Africa	Canada	Indonesia	Netherlands	United Kingdom	Mexico
33	Turkey	Argentina	Argentina	Indonesia	Israel	India	Colombia	Greece
34	Germany	Mexico	Chile	Brazil	Belgium	Peru	Netherlands	Canada
35	Netherlands	Peru	France	India	Peru	Colombia	France	Argentina
36	Sweden	Netherlands	Spain	Belgium	Mexico	France	Spain	Denmark
37	Austria	Colombia	India	Chile	Spain	Belgium	Hungary	Peru
38	Canada	Italy	Brazil	Czech Republic	Colombia	Spain	Belgium	Russia
39	Italy	Portugal	Philippines	Peru	Argentina	Argentina	Argentina	Ireland
40	Belgium	Brazil	United States	Poland	Australia	Australia	Australia	Spain
41	Ireland	United States	Mexico	Portugal	Brazil	Brazil	Brazil	Germany

42	Peru	Belgium	Peru	United Kingdom	Canada	Canada	Canada	Italy
43	United States	Spain	Portugal	Colombia	Egypt	Egypt	Egypt	Switzerland
44	Philippines	Canada	Netherlands	Philippines	Greece	Greece	Greece	Netherlands
45	France	France	Sweden	Argentina	Ireland	Ireland	Ireland	Poland
46	Spain	Sweden	Canada	Mexico	New Zealand	New Zealand	New Zealand	Hungary
47	Portugal	United Kingdom	United Kingdom	Sweden	Pakistan	Pakistan	Pakistan	France
48	United Kingdom	Egypt	Egypt	Egypt	Qatar	Qatar	Qatar	United Kingdom
49	Pakistan	Pakistan	Pakistan	Pakistan	South Africa	South Africa	South Africa	Belgium
50	United Arab Emirates	United Arab Emirates	United Arab Emirates	United Arab Emirates	United Arab Emirates	United Arab Emirates	United Arab Emirates	Sweden

Gray shading indicates the relevant data and indicators of the countries are not ranked due to incomplete information.

Supplementary Table S41. **Rankings of 50 countries and territories March–November 2020 (two-way sensitivity analysis)**

Rank	Mar	Apr	May	Jun	Jul	Aug	Sep	Oct	Nov
1	Taiwan	Taiwan	Taiwan	Taiwan	Taiwan	Taiwan	Taiwan	Taiwan	Taiwan
2	Egypt	Korea	Vietnam	Korea	New Zealand	Korea	Italy	Thailand	Korea
3	Russia	Vietnam	Korea	Vietnam	Korea	Finland	Korea	Korea	Japan
4	Qatar	Japan	Australia	New Zealand	Italy	Malaysia	Singapore	Japan	India
5	Poland	New Zealand	New Zealand	Japan	Malaysia	New Zealand	New Zealand	Hong Kong	Thailand
6	Korea	Australia	Switzerland	Finland	Netherlands	Turkey	Japan	India	Australia
7	Japan	Finland	Germany	Australia	Thailand	Italy	Germany	Finland	Hong Kong
8	Vietnam	Hong Kong	Hong Kong	Malaysia	Finland	Denmark	Thailand	Netherlands	Finland
9	Thailand	Portugal	Japan	Italy	Germany	Thailand	Malaysia	Denmark	Mexico
10	South Africa	Malaysia	Czech Republic	Turkey	Switzerland	Japan	Turkey	Italy	Malaysia
11	Indonesia	Russia	Russia	Denmark	France	Poland	Finland	Turkey	Denmark
12	Turkey	Indonesia	South Africa	Switzerland	Japan	United Kingdom	Poland	Canada	Germany
13	Colombia	Czech Republic	Austria	Hong Kong	Denmark	Switzerland	South Africa	United Kingdom	Italy
14	Mexico	South Africa	France	Czech Republic	Turkey	Germany	Australia	Mexico	Netherlands
15	Sweden	Saudi Arabia	Turkey	France	Poland	France	Portugal	Australia	Austria
16	Italy	Turkey	Poland	Netherlands	United Kingdom	Netherlands	Switzerland	Malaysia	Peru
17	Saudi Arabia	Poland	Netherlands	Hungary	Czech Republic	Canada	Netherlands	Germany	Chile
18	Hungary	Singapore	Egypt	Germany	India	Australia	Hong Kong	Sweden	Russia
19	Denmark	Denmark	Portugal	Russia	Singapore	Austria	France	Brazil	Colombia
20	Australia	Germany	Finland	South Africa	Hungary	Sweden	India	Austria	Poland

21	Brazil	India	Brazil	Portugal	Sweden	Portugal	United Kingdom	Peru	Sweden
22	Czech Republic	Egypt	Saudi Arabia	Saudi Arabia	Portugal	Singapore	Canada	Switzerland	Hungary
23	Finland	Brazil	Singapore	Singapore	Russia	Hungary	Russia	Russia	Switzerland
24	Hong Kong	Hungary	Malaysia	Spain	Hong Kong	India	Denmark	Hungary	Czech Republic
25	Malaysia	France	Hungary	Austria	Australia	Hong Kong	Brazil	Czech Republic	Canada
26	France	Argentina	Denmark	United Kingdom	Canada	Russia	Austria	Poland	United States
27	New Zealand	Switzerland	Indonesia	United States	United States	South Africa	Sweden	Chile	China
28	Argentina	Italy	India	Egypt	Spain	Brazil	Mexico	Colombia	Israel
29	Singapore	Austria	Italy	Canada	Brazil	Czech Republic	Peru	United States	Ireland
30	Netherlands	Netherlands	Chile	Brazil	South Africa	Mexico	United States	China	Philippines
31	Peru	Canada	Colombia	Poland	Austria	United States	Czech Republic	Norway	Argentina
32	Chile	Peru	Argentina	India	Colombia	Spain	Hungary	Israel	Belgium
33	Switzerland	Chile	Mexico	Colombia	Argentina	Chile	Chile	Ireland	Brazil
34	Spain	Colombia	Peru	Indonesia	Peru	Argentina	Spain	Belgium	Egypt
35	Canada	Sweden	Sweden	Mexico	Chile	Peru	Colombia	Philippines	France
36	India	Mexico	Spain	Sweden	Mexico	Colombia	Argentina	Argentina	Greece
37	Austria	United States	United Kingdom	Peru	China	China	China	Egypt	Indonesia
38	United Kingdom	United Kingdom	Canada	Argentina	Belgium	Belgium	Norway	France	New Zealand
39	Germany	Spain	United States	Chile	Egypt	Egypt	Ireland	Greece	Norway
40	Portugal	China	China	China	Greece	Greece	Greece	Indonesia	Pakistan
41	United States	Israel	Norway	Norway	Indonesia	Indonesia	Israel	New Zealand	Portugal
42	China	Norway	Greece	Greece	Ireland	Ireland	Belgium	Pakistan	Qatar
43	Norway	Greece	Israel	Ireland	Israel	Israel	Philippines	Portugal	Saudi Arabia

44	Greece	Ireland	Ireland	Israel	Norway	Norway	Egypt	Qatar	Singapore
45	Israel	Belgium	Belgium	Belgium	Pakistan	Pakistan	Indonesia	Saudi Arabia	South Africa
46	Belgium	Philippines	Philippines	Philippines	Philippines	Philippines	Pakistan	Singapore	Spain
47	Philippines	Pakistan	Pakistan	Pakistan	Qatar	Qatar	Qatar	South Africa	Turkey
48	Ireland	Qatar	Qatar	Qatar	Saudi Arabia	Saudi Arabia	Saudi Arabia	Spain	United Arab Emirates
49	Pakistan	Thailand	Thailand	Thailand	United Arab Emirates	United Arab Emirates	United Arab Emirates	United Arab Emirates	United Kingdom
50	United Arab Emirates	United Arab Emirates	United Arab Emirates	United Arab Emirates	Vietnam	Vietnam	Vietnam	Vietnam	Vietnam

Gray shading indicates the relevant data and indicators of the countries are not ranked due to incomplete information.

Supplementary Table S42. **Rankings of 50 countries and territories March–November 2020 by a three-month moving average (three-way sensitivity analysis)**

Rank	Mar	Apr	May	Jun	Jul	Aug	Sep	Oct	Nov
1	Taiwan	Taiwan	Taiwan	Taiwan	Taiwan	Taiwan	Taiwan	Taiwan	Taiwan
2	Egypt	Korea	Korea	Korea	Korea	Korea	Korea	Korea	Korea
3	Russia	Vietnam	Vietnam	Vietnam	New Zealand	New Zealand	New Zealand	Italy	Japan
4	Qatar	Japan	Japan	New Zealand	Japan	Malaysia	Italy	Thailand	Thailand
5	Poland	Russia	Australia	Japan	Switzerland	Finland	Malaysia	Finland	India
6	Korea	Egypt	New Zealand	Australia	Finland	Italy	Finland	Japan	Italy
7	Japan	Australia	Russia	Finland	Malaysia	Japan	Thailand	Turkey	Finland
8	Vietnam	Indonesia	South Africa	Hong Kong	Italy	Turkey	Japan	Malaysia	Hong Kong
9	Thailand	Poland	Hong Kong	Malaysia	Germany	Denmark	Turkey	Germany	Australia
10	South Africa	South Africa	Egypt	Turkey	Netherlands	Netherlands	Germany	Denmark	Germany
11	Indonesia	New Zealand	Poland	Czech Republic	Australia	Switzerland	Netherlands	Hong Kong	Malaysia
12	Turkey	Finland	Turkey	Russia	Turkey	Germany	Poland	India	Netherlands
13	Colombia	Turkey	Czech Republic	Switzerland	France	France	Switzerland	Netherlands	Denmark
14	Mexico	Hong Kong	Indonesia	Germany	Czech Republic	United Kingdom	Singapore	United Kingdom	Mexico
15	Sweden	Saudi Arabia	Finland	South Africa	Denmark	Australia	Denmark	Poland	Poland
16	Italy	Malaysia	Saudi Arabia	Portugal	Hong Kong	Poland	France	Australia	Russia
17	Saudi Arabia	Czech Republic	Malaysia	Denmark	Russia	Hungary	United Kingdom	Canada	Canada
18	Hungary	Denmark	Switzerland	France	Portugal	Portugal	Portugal	Switzerland	Austria
19	Denmark	Hungary	Germany	Saudi Arabia	Hungary	Singapore	India	Russia	Switzerland
20	Australia	Italy	France	Italy	Singapore	Czech Republic	Australia	Austria	Peru

21	Brazil	Brazil	Brazil	Austria	South Africa	Hong Kong	Hong Kong	Sweden	Sweden
22	Czech Republic	Singapore	Denmark	Singapore	Poland	Russia	Canada	Mexico	Chile
23	Finland	Portugal	Hungary	Netherlands	Austria	Austria	Sweden	Brazil	Hungary
24	Hong Kong	Colombia	Portugal	Hungary	United Kingdom	Canada	Russia	Hungary	Czech Republic
25	Malaysia	France	Singapore	Poland	India	South Africa	South Africa	Czech Republic	Colombia
26	France	Sweden	Italy	Egypt	Brazil	India	Hungary	Peru	United States
27	New Zealand	Argentina	Netherlands	Brazil	Spain	Sweden	Austria	United States	China
28	Argentina	Mexico	Austria	Indonesia	United States	Spain	Czech Republic	Chile	Argentina
29	Singapore	Netherlands	Colombia	India	Canada	United States	Brazil	Colombia	Belgium
30	Netherlands	India	India	Canada	Sweden	Brazil	United States	China	Brazil
31	Peru	Switzerland	Argentina	Colombia	Colombia	Mexico	Spain	Argentina	Egypt
32	Chile	Peru	Sweden	United Kingdom	Chile	Colombia	Mexico	Belgium	France
33	Switzerland	Germany	Chile	Spain	Peru	Peru	Peru	Egypt	Greece
34	Spain	Canada	Mexico	United States	Argentina	Argentina	Chile	France	Indonesia
35	Canada	Chile	Peru	Chile	Mexico	Chile	Argentina	Greece	Ireland
36	India	Austria	Canada	Argentina	China	China	Colombia	Indonesia	Israel
37	Austria	Spain	Spain	Peru	Belgium	Belgium	China	Ireland	New Zealand
38	United Kingdom	United Kingdom	United Kingdom	Sweden	Egypt	Egypt	Belgium	Israel	Norway
39	Germany	United States	United States	Mexico	Greece	Greece	Egypt	New Zealand	Pakistan
40	Portugal	China	China	China	Indonesia	Indonesia	Greece	Norway	Philippines
41	United States	Belgium	Belgium	Belgium	Ireland	Ireland	Indonesia	Pakistan	Portugal
42	China	Greece	Greece	Greece	Israel	Israel	Ireland	Philippines	Qatar
43	Belgium	Ireland	Ireland	Ireland	Norway	Norway	Israel	Portugal	Saudi Arabia

44	Greece	Israel	Israel	Israel	Pakistan	Pakistan	Norway	Qatar	Singapore
45	Ireland	Norway	Norway	Norway	Philippines	Philippines	Pakistan	Saudi Arabia	South Africa
46	Israel	Pakistan	Pakistan	Pakistan	Qatar	Qatar	Philippines	Singapore	Spain
47	Norway	Philippines	Philippines	Philippines	Saudi Arabia	Saudi Arabia	Qatar	South Africa	Turkey
48	Pakistan	Qatar	Qatar	Qatar	Thailand	Thailand	Saudi Arabia	Spain	United Arab Emirates
49	Philippines	Thailand	Thailand	Thailand	United Arab Emirates	United Arab Emirates	United Arab Emirates	United Arab Emirates	United Kingdom
50	United Arab Emirates	United Arab Emirates	United Arab Emirates	United Arab Emirates	Vietnam	Vietnam	Vietnam	Vietnam	Vietnam

Gray shading indicates the relevant data and indicators of the countries are not ranked due to incomplete information.

Supplementary Table S43. **Rankings of 50 countries and territories March–October 2020 (four-way sensitivity analysis)**

Rank	Mar	Apr	May	Jun	Jul	Aug	Sep	Oct
1	Taiwan	Taiwan	Taiwan	Taiwan	Taiwan	Taiwan	Taiwan	Taiwan
2	Egypt	Korea	Vietnam	Korea	Turkey	Malaysia	Korea	Korea
3	Korea	Japan	Korea	Vietnam	Malaysia	Korea	Japan	India
4	Poland	Vietnam	New Zealand	Japan	Korea	Turkey	Malaysia	Japan
5	Vietnam	Australia	Australia	New Zealand	Thailand	Finland	Italy	Hong Kong
6	Brazil	New Zealand	Czech Republic	Australia	Italy	Denmark	India	Netherlands
7	Qatar	Russia	Finland	Malaysia	Netherlands	Thailand	Singapore	Denmark
8	Russia	Czech Republic	Japan	Finland	Japan	Italy	Germany	Finland
9	Indonesia	Saudi Arabia	Russia	Italy	Germany	Japan	Hong Kong	Austria
10	Japan	Austria	Switzerland	South Africa	Hong Kong	Poland	Poland	Australia
11	Mexico	Finland	Germany	Turkey	India	India	Portugal	Poland
12	South Africa	Malaysia	Hong Kong	Switzerland	Denmark	Singapore	Finland	Russia
13	Thailand	Hong Kong	Turkey	Hong Kong	Russia	Germany	Russia	Switzerland
14	Italy	Poland	Hungary	Netherlands	Finland	Hong Kong	Switzerland	Mexico
15	Sweden	Portugal	Poland	Russia	Poland	Russia	Denmark	Germany
16	Colombia	Germany	South Africa	Denmark	Switzerland	Switzerland	Netherlands	Hungary
17	Hungary	Indonesia	France	Hungary	Singapore	Sweden	France	Canada
18	Denmark	Turkey	Italy	Saudi Arabia	United Kingdom	Portugal	Czech Republic	Czech Republic
19	Czech Republic	Singapore	Saudi Arabia	Singapore	Portugal	Czech Republic	Austria	Peru
20	Australia	Hungary	Austria	France	Austria	Hungary	United Kingdom	Colombia

21	Saudi Arabia	South Africa	Indonesia	United Kingdom	Hungary	France	Mexico	Sweden
22	Argentina	Denmark	Portugal	Austria	France	Netherlands	United States	United States
23	Finland	Netherlands	Singapore	Germany	Czech Republic	United Kingdom	Peru	China
24	Hong Kong	India	Netherlands	Portugal	Sweden	Chile	Sweden	Argentina
25	Turkey	France	Malaysia	Colombia	United States	Mexico	Chile	Belgium
26	New Zealand	Switzerland	Brazil	India	Colombia	Austria	Hungary	Brazil
27	Singapore	Argentina	Colombia	Poland	Peru	United States	Colombia	Chile
28	France	Italy	India	United States	Chile	Peru	Spain	Egypt
29	Netherlands	Canada	Chile	Canada	Mexico	Colombia	China	France
30	Malaysia	Peru	Denmark	Czech Republic	Spain	Spain	Argentina	Greece
31	Canada	Brazil	Argentina	Spain	China	China	Australia	Indonesia
32	Peru	Mexico	United Kingdom	Brazil	Argentina	Argentina	Belgium	Ireland
33	Spain	Chile	Mexico	Indonesia	Australia	Australia	Brazil	Israel
34	Switzerland	United Kingdom	Peru	Peru	Belgium	Belgium	Canada	Italy
35	Chile	United States	Sweden	Mexico	Brazil	Brazil	Egypt	Malaysia
36	Germany	Sweden	United States	Argentina	Canada	Canada	Greece	New Zealand
37	India	Colombia	Canada	Chile	Egypt	Egypt	Indonesia	Norway
38	Austria	Spain	Spain	Sweden	Greece	Greece	Ireland	Pakistan
39	United States	China	China	China	Indonesia	Indonesia	Israel	Philippines
40	Portugal	Belgium	Belgium	Belgium	Ireland	Ireland	New Zealand	Portugal
41	United Kingdom	Egypt	Egypt	Egypt	Israel	Israel	Norway	Qatar
42	China	Greece	Greece	Greece	New Zealand	New Zealand	Pakistan	Saudi Arabia
43	Belgium	Ireland	Ireland	Ireland	Norway	Norway	Philippines	Singapore

44	Greece	Israel	Israel	Israel	Pakistan	Pakistan	Qatar	South Africa
45	Ireland	Norway	Norway	Norway	Philippines	Philippines	Saudi Arabia	Spain
46	Israel	Pakistan	Pakistan	Pakistan	Qatar	Qatar	South Africa	Thailand
47	Norway	Philippines	Philippines	Philippines	Saudi Arabia	Saudi Arabia	Thailand	Turkey
48	Pakistan	Qatar	Qatar	Qatar	South Africa	South Africa	Turkey	United Arab Emirates
49	Philippines	Thailand	Thailand	Thailand	United Arab Emirates	United Arab Emirates	United Arab Emirates	United Kingdom
50	United Arab Emirates	United Arab Emirates	United Arab Emirates	United Arab Emirates	Vietnam	Vietnam	Vietnam	Vietnam

Gray shading indicates the relevant data and indicators of the countries are not ranked due to incomplete information.

Supplementary Table S44. **Rankings of 50 countries and territories March–October 2020 (three-way sensitivity analysis)**

Rank	Mar	Apr	May	Jun	Jul	Aug	Sep	Oct
1	Taiwan	Taiwan	Taiwan	Taiwan	Taiwan	Taiwan	Taiwan	Taiwan
2	Korea	Korea	Korea	Korea	Italy	Malaysia	Korea	Korea
3	Egypt	Vietnam	Vietnam	Vietnam	Malaysia	Korea	Italy	India
4	Poland	Australia	New Zealand	Japan	Korea	Turkey	Japan	Japan
5	Vietnam	Japan	Australia	New Zealand	Turkey	Finland	Malaysia	Hong Kong
6	Brazil	New Zealand	Switzerland	Australia	Thailand	Denmark	Singapore	Finland
7	Qatar	Czech Republic	Czech Republic	Malaysia	Netherlands	Italy	India	Netherlands
8	Russia	Hong Kong	Germany	Italy	Germany	Thailand	Germany	Denmark
9	South Africa	Russia	Japan	Switzerland	Finland	Poland	Hong Kong	Australia
10	Indonesia	Austria	Finland	Netherlands	Denmark	Singapore	Poland	Austria
11	Japan	Finland	Hong Kong	Finland	Japan	Japan	Portugal	Russia
12	Mexico	Malaysia	Russia	Turkey	Poland	Germany	Finland	Mexico
13	Thailand	Saudi Arabia	France	Denmark	Hong Kong	Hong Kong	Russia	Switzerland
14	Italy	Poland	Italy	Hong Kong	Switzerland	Russia	Switzerland	Canada
15	Sweden	Portugal	Turkey	Hungary	India	Sweden	Denmark	Czech Republic
16	Colombia	Germany	Austria	Russia	United Kingdom	India	United Kingdom	Germany
17	Hungary	Indonesia	Hungary	South Africa	Russia	Switzerland	Mexico	Poland
18	Denmark	Turkey	Poland	France	Singapore	Portugal	Netherlands	Hungary
19	Czech Republic	Switzerland	Portugal	Germany	Portugal	Hungary	France	Peru
20	Australia	Singapore	Saudi Arabia	Saudi Arabia	France	United Kingdom	Czech Republic	Colombia

21	Saudi Arabia	Hungary	Netherlands	Singapore	Hungary	Czech Republic	Austria	United States
22	Finland	South Africa	Malaysia	Spain	Sweden	Netherlands	United States	Sweden
23	Hong Kong	Denmark	South Africa	United Kingdom	Austria	France	Peru	China
24	Turkey	Netherlands	Indonesia	Austria	Czech Republic	Chile	Sweden	Argentina
25	Argentina	India	Singapore	Portugal	United States	Mexico	Chile	Belgium
26	New Zealand	France	Brazil	United States	Colombia	United States	Hungary	Brazil
27	Singapore	Italy	Denmark	India	Peru	Peru	Colombia	Chile
28	France	Canada	India	Canada	Chile	Austria	Spain	Egypt
29	Netherlands	Argentina	Colombia	Poland	Spain	Colombia	China	France
30	Malaysia	Peru	Chile	Brazil	Mexico	Spain	Argentina	Greece
31	Canada	Brazil	Argentina	Colombia	China	China	Australia	Indonesia
32	Peru	Mexico	United Kingdom	Czech Republic	Argentina	Argentina	Belgium	Ireland
33	Spain	Chile	Mexico	Indonesia	Australia	Australia	Brazil	Israel
34	Switzerland	United Kingdom	Peru	Peru	Belgium	Belgium	Canada	Italy
35	Germany	United States	Sweden	Mexico	Brazil	Brazil	Egypt	Malaysia
36	United States	Sweden	United States	Chile	Canada	Canada	Greece	New Zealand
37	India	Colombia	Spain	Argentina	Egypt	Egypt	Indonesia	Norway
38	Austria	Spain	Canada	Sweden	Greece	Greece	Ireland	Pakistan
39	Chile	China	China	China	Indonesia	Indonesia	Israel	Philippines
40	Portugal	Belgium	Belgium	Belgium	Ireland	Ireland	New Zealand	Portugal
41	United Kingdom	Egypt	Egypt	Egypt	Israel	Israel	Norway	Qatar
42	China	Greece	Greece	Greece	New Zealand	New Zealand	Pakistan	Saudi Arabia
43	Belgium	Ireland	Ireland	Ireland	Norway	Norway	Philippines	Singapore

44	Greece	Israel	Israel	Israel	Pakistan	Pakistan	Qatar	South Africa
45	Ireland	Norway	Norway	Norway	Philippines	Philippines	Saudi Arabia	Spain
46	Israel	Pakistan	Pakistan	Pakistan	Qatar	Qatar	South Africa	Thailand
47	Norway	Philippines	Philippines	Philippines	Saudi Arabia	Saudi Arabia	Thailand	Turkey
48	Pakistan	Qatar	Qatar	Qatar	South Africa	South Africa	Turkey	United Arab Emirates
49	Philippines	Thailand	Thailand	Thailand	United Arab Emirates	United Arab Emirates	United Arab Emirates	United Kingdom
50	United Arab Emirates	United Arab Emirates	United Arab Emirates	United Arab Emirates	Vietnam	Vietnam	Vietnam	Vietnam

Gray shading indicates the relevant data and indicators of the countries are not ranked due to incomplete information.

Supplementary Table S45. **Rankings of 50 countries and territories March 2020–July 2021 (with Google Trends)[a]**

**Mar 2020**	**R[b]**	**Apr2020**	**R[b]**	**May2020**	**R[b]**	**Jun 2020**	**R[b]**	**Jul 2020**	**R[b]**	**Aug 2020**	**R[b]**	**Sep 2020**	**R[b]**	**Oct 2020**	**R[b]**	**Nov 2020**	**R[b]**
Taiwan	1	Taiwan	1	South Korea	1	Taiwan	1	Taiwan	1	Taiwan	1	Taiwan	1	Taiwan	1	Taiwan	1
Japan	2	South Korea	2	Taiwan	2	South Korea	2	South Korea	2	South Korea	2	Singapore	2	Vietnam	2	Vietnam	2
Thailand	3	Japan	3	Vietnam	3	Vietnam	3	Netherlands	3	Turkey	3	South Korea	3	New Zealand	3	South Korea	3
Russia	4	Australia	4	Germany	4	Australia	4	New Zealand	4	New Zealand	4	Italy	4	Singapore	4	Egypt	4
Vietnam	5	Vietnam	5	Australia	5	New Zealand	5	Malaysia	5	Finland	5	New Zealand	5	Japan	5	New Zealand	5
Qatar	6	Finland	6	New Zealand	6	Malaysia	6	Finland	6	Thailand	6	Germany	6	India	6	India	6
Egypt	7	Hong Kong	7	Hong Kong	7	Turkey	7	Italy	7	Italy	7	Japan	7	South Korea	6	Australia	7
Mexico	8	Indonesia	8	Japan	8	Japan	8	France	8	Egypt	8	Finland	8	Hong Kong	8	Singapore	8
Australia	9	New Zealand	9	Austria	9	Finland	9	Germany	9	Malaysia	9	Thailand	9	Thailand	8	Japan	9
South Korea	10	Malaysia	10	Czech Republic	10	Italy	10	Thailand	9	Germany	10	South Africa	10	Saudi Arabia	10	Saudi Arabia	10
Italy	11	Qatar	11	France	11	Denmark	11	Denmark	11	Denmark	11	Turkey	10	Finland	11	Thailand	11
New Zealand	12	Russia	12	Malaysia	12	Germany	12	Japan	12	Singapore	12	Malaysia	12	Egypt	12	Hong Kong	12
Finland	13	Portugal	13	South Africa	13	Qatar	13	Egypt	13	United Kingdom	13	Vietnam	13	South Africa	13	South Africa	13
Chile	14	South Africa	14	Indonesia	14	France	14	Vietnam	14	Japan	14	Egypt	14	Turkey	13	Turkey	13
Hong Kong	15	Czech Republic	15	Qatar	15	Hong Kong	15	Turkey	15	Poland	15	Poland	14	Australia	15	Finland	15
Canada	16	Poland	16	Saudi Arabia	16	Russia	16	United Kingdom	16	Hungary	16	Saudi Arabia	16	Italy	16	Mexico	16

Sweden	16	Italy	17	Singapore	17	Czech Republic	17	Poland	17	Sweden	17	Canada	17	Denmark	17	Peru	17
France	18	Germany	18	Egypt	18	Austria	18	Czech Republic	18	France	18	Netherlands	17	Canada	18	Italy	18
Brazil	19	Saudi Arabia	18	Poland	18	Portugal	18	Hong Kong	19	Canada	19	Australia	19	Germany	19	Chile	19
India	20	India	20	Russia	20	South Africa	20	Hungary	19	Saudi Arabia	20	United Kingdom	20	Netherlands	20	France	20
Singapore	21	Hungary	21	Turkey	21	Netherlands	21	India	21	Austria	21	France	21	Chile	21	Germany	21
Malaysia	22	Singapore	22	Hungary	22	Hungary	22	Sweden	22	Netherlands	21	Denmark	22	Portugal	21	Brazil	22
Netherlands	23	Turkey	23	Italy	23	United Kingdom	22	Austria	23	Portugal	23	Hong Kong	23	Mexico	23	Portugal	23
Denmark	24	Austria	24	Portugal	24	Saudi Arabia	24	Canada	23	Russia	24	Russia	23	Peru	24	Sweden	24
United Kingdom	24	Argentina	25	Netherlands	25	United States	25	Spain	23	Hong Kong	25	Austria	25	Brazil	25	Malaysia	25
Germany	26	Peru	25	Denmark	26	Indonesia	26	Singapore	26	South Africa	25	Brazil	26	United Kingdom	26	Denmark	26
Austria	27	Brazil	27	India	27	Canada	27	Russia	27	Australia	27	Portugal	27	France	27	Netherlands	26
Spain	27	France	27	Brazil	28	Egypt	27	Saudi Arabia	28	India	28	India	28	Russia	28	Argentina	28
United States	27	Chile	29	Finland	29	Spain	29	Brazil	29	Vietnam	29	Sweden	29	Malaysia	29	Russia	28
Argentina	NA[c]	Denmark	30	Colombia	30	India	30	South Africa	29	Brazil	30	United States	30	Sweden	30	Austria	30
Belgium	NA[c]	Netherlands	30	Argentina	31	Singapore	31	Australia	31	United States	31	Peru	31	Austria	31	United Kingdom	31
China	NA[c]	Colombia	32	Spain	32	Poland	32	Portugal	31	Czech	32	Mexico	32	Colombia	32	United States	32

										Republic									
Colombia	NA[c]	Egypt	33	Canada	33	Brazil	33	United States	33	Chile	33	Spain	33	Poland	33	Canada	33		
Czech Republic	NA[c]	Canada	34	Chile	33	Chile	34	Argentina	34	Spain	33	Czech Republic	34	Argentina	34	Poland	33		
Greece	NA[c]	United States	34	Mexico	33	Colombia	35	Colombia	35	Colombia	35	Hungary	35	Czech Republic	34	Colombia	35		
Hungary	NA[c]	Mexico	36	Peru	33	Argentina	36	Chile	36	Argentina	36	Chile	36	Spain	36	Spain	36		
Indonesia	NA[c]	Spain	36	Sweden	37	Mexico	36	Peru	37	Mexico	36	Colombia	37	United States	37	Hungary	37		
Ireland	NA[c]	Sweden	38	United Kingdom	38	Peru	38	Mexico	38	Peru	38	Argentina	38	Hungary	38	Czech Republic	38		
Israel	NA[c]	United Kingdom	39	United States	39	Sweden	39	Belgium	NA[c]	Belgium	NA[c]	Belgium	NA[c]	Belgium	NA[c]	Belgium	NA[c]		
Norway	NA[c]	Belgium	NA[c]	Belgium	NA[c]	Belgium	NA[c]	China	NA[c]	China	NA[c]	China	NA[c]	China	NA[c]	China	NA[c]		
Pakistan	NA[c]	China	NA[c]	China	NA[c]	China	NA[c]	Greece	NA[c]	Greece	NA[c]	Greece	NA[c]	Greece	NA[c]	Greece	NA[c]		
Peru	NA[c]	Greece	NA[c]	Greece	NA[c]	Greece	NA[c]	Indonesia	NA[c]	Indonesia	NA[c]	Indonesia	NA[c]	Indonesia	NA[c]	Indonesia	NA[c]		
Philippines	NA[c]	Ireland	NA[c]	Ireland	NA[c]	Ireland	NA[c]	Ireland	NA[c]	Ireland	NA[c]	Ireland	NA[c]	Ireland	NA[c]	Ireland	NA[c]		
Poland	NA[c]	Israel	NA[c]	Israel	NA[c]	Israel	NA[c]	Israel	NA[c]	Israel	NA[c]	Israel	NA[c]	Israel	NA[c]	Israel	NA[c]		
Portugal	NA[c]	Norway	NA[c]	Norway	NA[c]	Norway	NA[c]	Norway	NA[c]	Norway	NA[c]	Norway	NA[c]	Norway	NA[c]	Norway	NA[c]		
Saudi Arabia	NA[c]	Pakistan	NA[c]	Pakistan	NA[c]	Pakistan	NA[c]	Pakistan	NA[c]	Pakistan	NA[c]	Pakistan	NA[c]	Pakistan	NA[c]	Pakistan	NA[c]		
South Africa	NA[c]	Philippines	NA[c]	Philippines	NA[c]	Philippines	NA[c]	Philippines	NA[c]	Philippines	NA[c]	Philippines	NA[c]	Philippines	NA[c]	Philippines	NA[c]		
Switzerland	NA[c]	Switzerland	NA[c]	Switzerland	NA[c]	Switzerland	NA[c]	Qatar	NA[c]	Qatar	NA[c]	Qatar	NA[c]	Qatar	NA[c]	Qatar	NA[c]		
Turkey	NA[c]	Thailand	NA[c]	Thailand	NA[c]	Thailand	NA[c]	Switzerland	NA[c]	Switzerland	NA[c]	Switzerland	NA[c]	Switzerland	NA[c]	Switzerland	NA[c]		
United Arab	NA[c]	United Arab	NA[c]	United Arab	NA[c]	United Arab	NA[c]	United Arab	NA[c]	United Arab	NA[c]	United Arab	NA[c]	United Arab	NA[c]	United Arab	NA[c]		

Emirates	Emirates	Emirates	Emirates	Emirates	Emirates	Emirates	Emirates	Emirates

Notes:

a. For the calculation method of the indicator, please refer to the alternative calculation method we mentioned in Supplementary Information 7.

b. R means each country's ranking.

c. NA: not applicable. Indicates a country not ranked due to incomplete data.

Dec 2020	R[b]	Jan 2021	R[b]	Feb 2021	R[b]	Mar 2021	R[b]	Apr 2021	R[b]	May 2021	R[b]	Jun 2021	R[b]	Jul 2021	R[b]
Taiwan	1	Singapore	1	Singapore	1	Singapore	1	Singapore	1	Singapore	1	Singapore	1	Singapore	1
Germany	2	Taiwan	2	India	2	Vietnam	2	United Kingdom	2	United Kingdom	2	United States	2	Qatar	2
Chile	3	Saudi Arabia	3	Saudi Arabia	3	New Zealand	3	Malaysia	3	Mexico	3	Austria	3	Canada	3
Finland	4	Turkey	4	Denmark	4	Saudi Arabia	4	Russia	4	Austria	4	Poland	4	Hungary	4
Argentina	5	Italy	5	Turkey	5	South Korea	5	Austria	5	Russia	5	Germany	5	Sweden	5
Poland	6	India	6	Taiwan	6	Taiwan	6	United States	6	Portugal	6	South Korea	6	Austria	6
Portugal	7	Argentina	7	New Zealand	7	Australia	7	Mexico	7	Italy	7	Czech Republic	7	Italy	6
Mexico	8	Egypt	8	Australia	8	Canada	8	Portugal	8	Spain	8	Portugal	8	Germany	8
Denmark	9	Finland	9	Italy	9	Turkey	8	Vietnam	9	United States	9	Italy	9	New Zealand	9
Austria	10	Germany	9	South Korea	10	Austria	10	Italy	10	South Korea	10	Mexico	10	Poland	10
Italy	10	Denmark	11	Argentina	11	Hong Kong	10	Spain	11	Germany	11	Spain	11	Czech Republic	11
Canada	12	Portugal	11	Japan	12	Japan	12	Germany	12	Malaysia	12	Hong Kong	12	Hong Kong	12
Russia	12	Austria	13	Finland	13	Argentina	13	Taiwan	12	Hong Kong	13	United Kingdom	13	United Arab Emirates	13
United Kingdom	14	France	13	Mexico	14	Egypt	14	South Korea	14	Poland	14	Sweden	14	South Korea	14
Sweden	15	Russia	15	France	15	United Kingdom	15	France	15	France	15	France	15	Australia	15
France	16	Hungary	16	Canada	16	India	16	Japan	16	Sweden	16	Japan	16	Egypt	16
United States	17	Sweden	17	Sweden	17	Malaysia	17	Hong Kong	17	Vietnam	17	Malaysia	17	Chile	17
Czech Republic	18	Spain	18	Thailand	18	Thailand	18	Poland	18	Taiwan	18	Vietnam	18	Denmark	18

Hungary	19	Poland	19	Germany	19	Germany	19	Czech Republic	19	Japan	19	Russia	19	Saudi Arabia	19
Australia	NA[c]	Netherlands	20	Austria	20	Russia	19	Sweden	20	Czech Republic	20	Taiwan	20	Portugal	20
Belgium	NA[c]	Brazil	21	Malaysia	21	Denmark	21	Argentina	NA[c]	Argentina	NA[c]	Argentina	NA[c]	United Kingdom	21
Brazil	NA[c]	Indonesia	21	Hong Kong	22	France	22	Australia	NA[c]	Australia	NA[c]	Australia	NA[c]	Spain	22
China	NA[c]	United Kingdom	23	United Kingdom	23	Portugal	22	Belgium	NA[c]	Belgium	NA[c]	Belgium	NA[c]	Japan	23
Colombia	NA[c]	Chile	24	Portugal	24	United States	24	Brazil	NA[c]	Brazil	NA[c]	Brazil	NA[c]	Finland	24
Egypt	NA[c]	Czech Republic	25	Netherlands	25	Spain	25	Canada	NA[c]	Canada	NA[c]	Canada	NA[c]	Netherlands	24
Greece	NA[c]	United States	26	Chile	26	Italy	26	Chile	NA[c]	Chile	NA[c]	Chile	NA[c]	France	26
Hong Kong	NA[c]	Mexico	27	Poland	27	Colombia	27	China	NA[c]	China	NA[c]	China	NA[c]	Taiwan	27
India	NA[c]	Canada	28	South Africa	28	Sweden	27	Colombia	NA[c]	Colombia	NA[c]	Colombia	NA[c]	Peru	28
Indonesia	NA[c]	Australia	NA[c]	Czech Republic	29	Peru	29	Denmark	NA[c]	Denmark	NA[c]	Denmark	NA[c]	United States	28
Ireland	NA[c]	Belgium	NA[c]	Colombia	30	Mexico	30	Egypt	NA[c]	Egypt	NA[c]	Egypt	NA[c]	Malaysia	30
Israel	NA[c]	China	NA[c]	Spain	31	South Africa	30	Finland	NA[c]	Finland	NA[c]	Finland	NA[c]	India	31
Japan	NA[c]	Colombia	NA[c]	Indonesia	32	Netherlands	32	Greece	NA[c]	Greece	NA[c]	Greece	NA[c]	Thailand	32
Malaysia	NA[c]	Greece	NA[c]	Peru	33	Finland	33	Hungary	NA[c]	Hungary	NA[c]	Hungary	NA[c]	Turkey	33
Netherlands	NA[c]	Hong Kong	NA[c]	United States	34	Czech Republic	34	India	NA[c]	India	NA[c]	India	NA[c]	Brazil	34
New Zealand	NA[c]	Ireland	NA[c]	Brazil	35	Indonesia	35	Indonesia	NA[c]	Indonesia	NA[c]	Indonesia	NA[c]	Argentina	35
Norway	NA[c]	Israel	NA[c]	Hungary	36	Hungary	36	Ireland	NA[c]	Ireland	NA[c]	Ireland	NA[c]	Colombia	35
Pakistan	NA[c]	Japan	NA[c]	Belgium	NA[c]	Chile	37	Israel	NA[c]	Israel	NA[c]	Israel	NA[c]	Mexico	37
Peru	NA[c]	Malaysia	NA[c]	China	NA[c]	Poland	38	Netherlands	NA[c]	Netherlands	NA[c]	Netherlands	NA[c]	South Africa	38
Philippines	NA[c]	New Zealand	NA[c]	Egypt	NA[c]	Brazil	39	New Zealand	NA[c]	New Zealand	NA[c]	New Zealand	NA[c]	Vietnam	38

Qatar	NA[c]	Norway	NA[c]	Greece	NA[c]	Belgium	NA[c]	Norway	NA[c]	Norway	NA[c]	Norway	NA[c]	Indonesia	40
Saudi Arabia	NA[c]	Pakistan	NA[c]	Ireland	NA[c]	China	NA[c]	Pakistan	NA[c]	Pakistan	NA[c]	Pakistan	NA[c]	Russia	41
Singapore	NA[c]	Peru	NA[c]	Israel	NA[c]	Greece	NA[c]	Peru	NA[c]	Peru	NA[c]	Peru	NA[c]	Belgium	NA[c]
South Africa	NA[c]	Philippines	NA[c]	Norway	NA[c]	Ireland	NA[c]	Philippines	NA[c]	Philippines	NA[c]	Philippines	NA[c]	China	NA[c]
South Korea	NA[c]	Qatar	NA[c]	Pakistan	NA[c]	Israel	NA[c]	Qatar	NA[c]	Qatar	NA[c]	Qatar	NA[c]	Greece	NA[c]
Spain	NA[c]	South Africa	NA[c]	Philippines	NA[c]	Norway	NA[c]	Saudi Arabia	NA[c]	Saudi Arabia	NA[c]	Saudi Arabia	NA[c]	Ireland	NA[c]
Switzerland	NA[c]	South Korea	NA[c]	Qatar	NA[c]	Pakistan	NA[c]	South Africa	NA[c]	South Africa	NA[c]	South Africa	NA[c]	Israel	NA[c]
Thailand	NA[c]	Switzerland	NA[c]	Russia	NA[c]	Philippines	NA[c]	Switzerland	NA[c]	Switzerland	NA[c]	Switzerland	NA[c]	Norway	NA[c]
Turkey	NA[c]	Thailand	NA[c]	Switzerland	NA[c]	Qatar	NA[c]	Thailand	NA[c]	Thailand	NA[c]	Thailand	NA[c]	Pakistan	NA[c]
United Arab Emirates	NA[c]	United Arab Emirates	NA[c]	United Arab Emirates	NA[c]	Switzerland	NA[c]	Turkey	NA[c]	Turkey	NA[c]	Turkey	NA[c]	Philippines	NA[c]
Vietnam	NA[c]	Vietnam	NA[c]	Vietnam	NA[c]	United Arab Emirates	NA[c]	United Arab Emirates	NA[c]	United Arab Emirates	NA[c]	United Arab Emirates	NA[c]	Switzerland	NA[c]

Notes:

a. For the calculation method of the indicator, please refer to the alternative calculation method we mentioned in Supplementary Information 7.

b. R means each country’s ranking.

c. NA: not applicable. Indicates a country not ranked due to incomplete data.

Supplementary Table S46. **Rankings of 50 countries and territories March 2020–August 2021 (without Google Trends)[a]**

Mar 2020	R[b]	Apr 2020	R[b]	May 2020	R[b]	Jun 2020	R[b]	Jul 2020	R[b]	Aug 2020	R[b]	Sep 2020	R[b]	Oct 2020	R[b]	Nov 2020	R[b]
Taiwan	1	Taiwan	1	South Korea	1	Taiwan	1	Taiwan	1	Taiwan	1	Taiwan	1	Taiwan	1	Taiwan	1
Vietnam	2	Vietnam	2	Vietnam	2	South Korea	2	New Zealand	2	China	2	China	2	Vietnam	2	China	2
Japan	3	South Korea	3	Australia	3	Vietnam	3	China	3	South Korea	2	Singapore	3	New Zealand	3	New Zealand	3
Mexico	4	Hong Kong	4	China	4	New Zealand	4	South Korea	3	Turkey	4	South Korea	4	China	4	Vietnam	3
Thailand	5	China	5	Taiwan	5	China	5	Finland	5	Thailand	5	Thailand	5	Singapore	5	Australia	5
Egypt	6	Australia	6	Hong Kong	6	Australia	6	Netherlands	6	New Zealand	6	New Zealand	6	Hong Kong	6	Singapore	5
Norway	6	Israel	7	Norway	7	Finland	7	Germany	7	Italy	7	Germany	7	South Korea	7	South Korea	5
Russia	8	Malaysia	8	New Zealand	8	Norway	8	Malaysia	7	Finland	8	Norway	8	Japan	8	Thailand	8
South Korea	9	New Zealand	9	Germany	9	Switzerland	9	Thailand	7	Norway	9	Vietnam	8	Thailand	9	Egypt	9
Qatar	10	Japan	10	Switzerland	10	Turkey	10	Ireland	10	Germany	10	Italy	10	Norway	10	Hong Kong	9
Hong Kong	11	South Africa	11	Greece	11	Japan	11	Italy	11	Egypt	11	Finland	11	India	11	India	11
Singapore	12	Qatar	12	Czech Republic	12	Malaysia	12	Norway	12	Ireland	11	Japan	11	Saudi Arabia	11	Finland	12
Greece	13	Indonesia	13	Qatar	12	Qatar	12	France	13	Malaysia	13	Egypt	13	Finland	13	Japan	12
Australia	14	Finland	14	Japan	14	Hong Kong	14	Denmark	14	Switzerland	13	Turkey	14	Egypt	14	Saudi Arabia	14
Malaysia	15	Greece	14	Indonesia	15	Italy	15	Greece	15	Singapore	15	Malaysia	15	Turkey	15	Israel	15
Chile	16	Norway	14	Israel	15	Denmark	16	Japan	16	Japan	16	South Africa	16	Australia	16	South Africa	16
Israel	17	Czech Republic	17	Turkey	17	Germany	17	Switzerland	17	Greece	17	Hong Kong	17	South Africa	17	Turkey	16

New Zealand	17	Italy	18	Malaysia	18	Czech Republic	18	Vietnam	17	Austria	18	Poland	18	Brazil	18	Ireland	18
Italy	19	Portugal	19	Austria	19	Greece	18	Turkey	19	Poland	18	Australia	19	Italy	18	Chile	19
China	20	Brazil	20	Singapore	20	Israel	18	Czech Republic	20	United Kingdom	18	Saudi Arabia	20	Germany	20	Norway	19
India	21	Hungary	20	South Africa	20	Hungary	21	Egypt	20	Denmark	21	Greece	21	Israel	20	France	21
Sweden	21	Ireland	22	Ireland	22	Ireland	22	Hungary	22	Hungary	21	Switzerland	21	Canada	22	Peru	22
Finland	23	Poland	23	Belgium	23	Austria	23	Poland	22	Sweden	23	Ireland	23	Chile	22	Mexico	23
France	24	Russia	23	Hungary	23	France	23	Belgium	24	Netherlands	24	United Kingdom	24	Denmark	22	Germany	24
Brazil	25	Germany	25	Poland	23	Netherlands	25	United Kingdom	24	France	25	Sweden	25	Ireland	22	Brazil	25
Canada	26	Saudi Arabia	25	Russia	26	Portugal	25	Singapore	26	Vietnam	26	Canada	26	Mexico	22	Italy	25
Netherlands	27	Egypt	27	France	27	Spain	25	Sweden	27	Hong Kong	27	Denmark	26	Greece	27	Malaysia	25
Philippines	28	Turkey	27	Egypt	28	Russia	28	Austria	28	Saudi Arabia	27	Israel	26	Peru	28	Argentina	28
Belgium	29	Belgium	29	Saudi Arabia	28	Saudi Arabia	28	Spain	28	Belgium	29	Netherlands	26	Switzerland	28	Portugal	28
Denmark	29	India	30	Brazil	30	Belgium	30	Hong Kong	30	Canada	30	Portugal	26	Netherlands	30	Denmark	30
Switzerland	29	Singapore	31	Denmark	31	United Kingdom	31	Saudi Arabia	31	Portugal	30	France	31	Portugal	30	Netherlands	30
Ireland	32	Philippines	32	Portugal	32	Brazil	32	India	32	United States	32	United States	32	France	32	Greece	32
Germany	33	France	33	Netherlands	33	Singapore	32	Canada	33	India	33	Austria	33	Malaysia	33	Belgium	33
Austria	34	Argentina	34	Spain	34	South Africa	32	Israel	33	Russia	33	Belgium	33	Austria	34	Philippines	33
Spain	34	Austria	34	Italy	35	Egypt	35	Portugal	33	Czech	35	India	33	Sweden	35	Poland	35

										Republic							
United Kingdom	34	Chile	34	Philippines	36	United States	36	Russia	36	Israel	35	Russia	33	United Kingdom	36	United Kingdom	35
United States	37	Peru	34	Chile	37	Canada	37	United States	36	Australia	37	Brazil	37	United States	37	Austria	37
Argentina	NA[c]	Denmark	38	Mexico	37	Indonesia	37	Brazil	38	Brazil	38	Mexico	38	Argentina	38	Russia	38
Colombia	NA[c]	Switzerland	38	Finland	39	Poland	39	South Africa	38	South Africa	38	Czech Republic	39	Czech Republic	38	Switzerland	38
Czech Republic	NA[c]	Colombia	40	India	40	Mexico	40	Argentina	40	Mexico	40	Philippines	39	Philippines	38	Sweden	40
Hungary	NA[c]	Netherlands	41	Colombia	41	Philippines	41	Australia	40	Spain	40	Hungary	41	Russia	38	Hungary	41
Indonesia	NA[c]	Mexico	42	Argentina	42	India	42	Philippines	42	Philippines	42	Peru	42	Spain	38	Colombia	42
Pakistan	NA[c]	Spain	42	Peru	43	Argentina	43	Peru	43	Argentina	43	Argentina	43	Belgium	43	United States	43
Peru	NA[c]	United States	44	United States	44	Chile	43	Chile	44	Chile	43	Chile	43	Colombia	44	Czech Republic	44
Poland	NA[c]	Sweden	45	United Kingdom	45	Colombia	45	Mexico	44	Colombia	45	Spain	43	Hungary	45	Spain	44
Portugal	NA[c]	Canada	46	Canada	46	Peru	46	Colombia	46	Peru	45	Colombia	46	Poland	45	Canada	46
Saudi Arabia	NA[c]	United Kingdom	47	Sweden	47	Sweden	47	Indonesia	NA[c]	Indonesia	NA[c]	Indonesia	NA[c]	Indonesia	NA[c]	Indonesia	NA[c]
South Africa	NA[c]	Pakistan	NA[c]	Pakistan	NA[c]	Pakistan	NA[c]	Pakistan	NA[c]	Pakistan	NA[c]	Pakistan	NA[c]	Pakistan	NA[c]	Pakistan	NA[c]
Turkey	NA[c]	Thailand	NA[c]	Thailand	NA[c]	Thailand	NA[c]	Qatar	NA[c]	Qatar	NA[c]	Qatar	NA[c]	Qatar	NA[c]	Qatar	NA[c]
United Arab	NA[c]	United Arab	NA[c]	United Arab	NA[c]	United Arab	NA[c]	United Arab	NA[c]	United Arab	NA[c]	United Arab	NA[c]	United Arab	NA[c]	United Arab	NA[c]

Emirates	Emirates	Emirates	Emirates	Emirates	Emirates	Emirates	Emirates	Emirates

Notes:

a. For the calculation method of the indicator, please refer to the alternative calculation method we mentioned in Supplementary Information 7.

b. R means each country's ranking.

c. NA: not applicable. Indicates a country not ranked due to incomplete data.

Dec 2020	R[b]	Jan 2021	R[b]	Feb 2021	R[b]	Mar 2021	R[b]	Apr 2021	R[b]	May 2021	R[b]	Jun 2021	R[b]	Jul 2021	R[b]	Aug 2021	R[b]
China	1	Singapore	1	Singapore	1	Singapore	1	Singapore	1	Singapore	1	Singapore	1	China	1	China	1
Taiwan	2	China	2	China	2	China	2	United Kingdom	2	United Kingdom	2	China	2	Switzerland	2	Singapore	2
Norway	3	Taiwan	3	India	3	Vietnam	3	China	3	China	3	United States	3	Qatar	3	Czech Republic	3
Finland	4	Turkey	4	Turkey	4	New Zealand	4	Malaysia	4	Mexico	4	Austria	4	Singapore	4	Sweden	4
Israel	5	Italy	5	New Zealand	5	Australia	5	Mexico	5	Portugal	5	Germany	5	Austria	5	Canada	5
Argentina	6	Saudi Arabia	5	Denmark	6	Saudi Arabia	6	Spain	5	United States	6	Hong Kong	5	Hungary	6	Hungary	5
Chile	7	India	7	Taiwan	7	Taiwan	7	United States	7	Austria	7	Portugal	5	Norway	7	Poland	7
Germany	8	Argentina	8	Saudi Arabia	8	India	8	Austria	8	Spain	8	South Korea	8	Sweden	8	Austria	8
Portugal	9	Finland	9	Australia	9	Canada	9	Portugal	8	Italy	9	Mexico	9	Canada	9	Italy	8
Denmark	10	Denmark	10	Argentina	10	South Korea	9	Russia	10	Russia	10	Poland	9	Czech Republic	9	Norway	10
Ireland	10	Greece	11	South Korea	10	Turkey	9	Vietnam	10	South Korea	11	Italy	11	New Zealand	11	Belgium	11
Italy	12	Israel	11	Finland	12	Hong Kong	12	South Korea	12	Germany	12	Czech Republic	12	Germany	12	Chile	12
Poland	13	Spain	13	Italy	13	Austria	13	Germany	13	France	13	Belgium	13	Italy	12	Germany	12
Czech Republic	14	Belgium	14	Japan	14	Japan	14	Taiwan	13	Hong Kong	14	United Kingdom	14	Poland	14	Portugal	12
Greece	15	Egypt	14	Thailand	14	United	15	France	15	Malaysia	15	Spain	15	Belgium	15	Saudi Arabia	15

						Kingdom											
Canada	16	Portugal	16	Belgium	16	Thailand	16	Italy	15	Poland	16	Sweden	16	United Arab Emirates	15	Egypt	16
Switzerland	16	Ireland	17	Switzerland	16	Argentina	17	Hong Kong	17	Philippines	17	France	17	Hong Kong	17	Denmark	17
Mexico	18	Hungary	18	Norway	18	Denmark	17	Philippines	17	Belgium	18	Philippines	18	Israel	18	South Korea	17
United Kingdom	19	Germany	19	Hong Kong	19	Spain	17	Japan	19	Sweden	19	Japan	19	Egypt	19	United Arab Emirates	17
Austria	20	Czech Republic	20	Israel	20	Egypt	20	Belgium	20	Vietnam	20	Malaysia	20	Chile	20	Colombia	20
France	20	France	21	Austria	21	France	20	Czech Republic	21	Japan	21	Vietnam	21	Denmark	21	New Zealand	21
United States	22	Switzerland	22	France	21	United States	22	Poland	22	Czech Republic	22	Russia	22	Portugal	21	France	22
Sweden	23	Chile	23	Greece	23	Switzerland	23	Sweden	23	Taiwan	23	Taiwan	23	South Korea	21	Netherlands	22
Belgium	24	Norway	23	Canada	24	Germany	24	Argentina	NA[c]	Argentina	NA[c]	Argentina	NA[c]	Australia	24	Australia	24
Russia	24	Poland	25	Chile	24	Portugal	25	Australia	NA[c]	Australia	NA[c]	Australia	NA[c]	Ireland	24	India	24
Hungary	26	Austria	26	Ireland	24	Malaysia	26	Brazil	NA[c]	Brazil	NA[c]	Brazil	NA[c]	Finland	26	Ireland	24
Australia	NA[c]	Russia	26	United Kingdom	24	Norway	27	Canada	NA[c]	Canada	NA[c]	Canada	NA[c]	Saudi Arabia	27	Israel	24
Brazil	NA[c]	United Kingdom	28	Germany	28	Israel	28	Chile	NA[c]	Chile	NA[c]	Chile	NA[c]	France	28	Taiwan	28
Colombia	NA[c]	United States	28	Sweden	28	Colombia	29	Colombia	NA[c]	Colombia	NA[c]	Colombia	NA[c]	United Kingdom	28	Japan	29

Egypt	NA[c]	Brazil	30	Czech Republic	30	Russia	29	Denmark	NA[c]	Denmark	NA[c]	Denmark	NA[c]	United States	28	Peru	30
Hong Kong	NA[c]	Netherlands	31	Portugal	31	Ireland	31	Egypt	NA[c]	Egypt	NA[c]	Egypt	NA[c]	India	31	Switzerland	30
India	NA[c]	Sweden	31	Mexico	32	Mexico	32	Finland	NA[c]	Finland	NA[c]	Finland	NA[c]	Pakistan	31	United Kingdom	30
Indonesia	NA[c]	Indonesia	33	Malaysia	33	Finland	33	Greece	NA[c]	Greece	NA[c]	Greece	NA[c]	Taiwan	31	Finland	33
Japan	NA[c]	Mexico	34	Netherlands	34	Sweden	34	Hungary	NA[c]	Hungary	NA[c]	Hungary	NA[c]	Turkey	31	Pakistan	34
Malaysia	NA[c]	Canada	35	South Africa	35	Czech Republic	35	India	NA[c]	India	NA[c]	India	NA[c]	Netherlands	35	Argentina	35
Netherlands	NA[c]	Australia	NA[c]	Spain	35	Greece	35	Indonesia	NA[c]	Indonesia	NA[c]	Indonesia	NA[c]	Spain	36	Spain	36
New Zealand	NA[c]	Colombia	NA[c]	United States	35	Italy	37	Ireland	NA[c]	Ireland	NA[c]	Ireland	NA[c]	Japan	37	Greece	37
Pakistan	NA[c]	Hong Kong	NA[c]	Colombia	38	Chile	38	Israel	NA[c]	Israel	NA[c]	Israel	NA[c]	Peru	38	Indonesia	37
Peru	NA[c]	Japan	NA[c]	Poland	38	Belgium	39	Netherlands	NA[c]	Netherlands	NA[c]	Netherlands	NA[c]	Philippines	39	South Africa	37
Philippines	NA[c]	Malaysia	NA[c]	Hungary	40	South Africa	39	New Zealand	NA[c]	New Zealand	NA[c]	New Zealand	NA[c]	Brazil	40	United States	37
Qatar	NA[c]	New Zealand	NA[c]	Brazil	41	Netherlands	41	Norway	NA[c]	Norway	NA[c]	Norway	NA[c]	Greece	40	Turkey	41
Saudi Arabia	NA[c]	Pakistan	NA[c]	Indonesia	42	Hungary	42	Pakistan	NA[c]	Pakistan	NA[c]	Pakistan	NA[c]	Malaysia	42	Brazil	42
Singapore	NA[c]	Peru	NA[c]	Peru	43	Peru	42	Peru	NA[c]	Peru	NA[c]	Peru	NA[c]	Argentina	43	Mexico	43
South Africa	NA[c]	Philippines	NA[c]	Philippines	43	Brazil	44	Qatar	NA[c]	Qatar	NA[c]	Qatar	NA[c]	Mexico	43	Philippines	43
South Korea	NA[c]	Qatar	NA[c]	Egypt	NA[c]	Indonesia	44	Saudi Arabia	NA[c]	Saudi Arabia	NA[c]	Saudi Arabia	NA[c]	Vietnam	43	Thailand	43
Spain	NA[c]	South Africa	NA[c]	Pakistan	NA[c]	Poland	46	South Africa	NA[c]	South Africa	NA[c]	South Africa	NA[c]	Thailand	46	Vietnam	43
Thailand	NA[c]	South Korea	NA[c]	Qatar	NA[c]	Philippines	47	Switzerland	NA[c]	Switzerland	NA[c]	Switzerland	NA[c]	Indonesia	47	Russia	47
Turkey	NA[c]	Thailand	NA[c]	Russia	NA[c]	Pakistan	NA[c]	Thailand	NA[c]	Thailand	NA[c]	Thailand	NA[c]	Colombia	48	Hong Kong	NA[c]
United Arab	NA[c]	United Arab	NA[c]	United Arab	NA[c]	Qatar	NA[c]	Turkey	NA[c]	Turkey	NA[c]	Turkey	NA[c]	South Africa	49	Malaysia	NA[c]

Emirates		Emirates		Emirates													
Vietnam	NA[c]	Vietnam	NA[c]	Vietnam	NA[c]	United Arab Emirates	NA[c]	United Arab Emirates	NA[c]	United Arab Emirates	NA[c]	United Arab Emirates	NA[c]	Russia	50	Qatar	NA[c]

Notes:

a. For the calculation method of the indicator, please refer to the alternative calculation method we mentioned in Supplementary Information 7.

b. R means each country’s ranking.

c. NA: not applicable. Indicates a country not ranked due to incomplete data.

Supplementary Information 14. Vaccination Coverage

***Supplementary Table S47.* Association between people covered by vaccines and one-month cases per 100,000 members of the population from December 2020 to August 2021.**

People covered by vaccines	One-month cases per 100,000 members of the population														
	Dec 2020			Jan 2021			Feb 2021			Mar 2021			Apr 2021		
	n	r_s[a]	*P* value[b]	n	r_s[a]	*P* value[b]	n	r_s[a]	*P* value[b]	n	r_s[a]	*P* value[b]	n	r_s[a]	*P* value[b]
Dec 2020	27	0.49	**0.010**	27	0.50	**0.009**	27	0.15	0.452	27	-0.19	0.351	27	-0.33	0.093
Jan 2021	37	0.63	**<.0001**	37	0.67	**<.0001**	37	0.59	**0.0001**	37	0.31	0.058	37	0.09	0.587
Feb 2021	47	0.71	**<.0001**	47	0.64	**<.0001**	47	0.66	**<.0001**	47	0.62	**<.0001**	47	0.46	**0.001**
Mar 2021	50	0.62	**<.0001**	50	0.58	**<.0001**	50	0.63	**<.0001**	50	0.62	**<.0001**	50	0.48	**0.0004**
Apr 2021	50	0.60	**<.0001**	50	0.57	**<.0001**	50	0.63	**<.0001**	50	0.60	**<.0001**	50	0.45	**0.001**
May 2021	50	0.62	**<.0001**	50	0.57	**<.0001**	50	0.63	**<.0001**	50	0.59	**<.0001**	50	0.46	**0.001**
Jun 2021	50	0.57	**<.0001**	50	0.54	**<.0001**	50	0.59	**<.0001**	50	0.55	**<.0001**	50	0.43	**0.002**
Jul 2021	50	0.50	**0.0002**	50	0.52	**0.0001**	50	0.56	**<.0001**	50	0.49	**0.0003**	50	0.39	**0.005**
Aug 2021	50	0.44	**0.001**	50	0.48	**0.0004**	50	0.52	**0.0001**	50	0.44	**0.001**	50	0.34	**0.017**

[a] Spearman Correlation Coefficients.

[b] *P* value indicating correlation is significantly different than zero.

People covered by vaccines	One-month cases per 100,000 members of the population											
	May 2021			Jun 2021			Jul 2021			Aug 2021		
	n	r_s[a]	*P* value[b]	n	r_s[a]	*P* value[b]	n	r_s[a]	*P* value[b]	n	r_s[a]	*P* value[b]
Dec 2020	27	-0.22	0.280	27	-0.02	0.912	27	0.13	0.533	27	0.24	0.233
Jan 2021	37	0.01	0.943	37	0.07	0.700	37	0.25	0.133	37	0.50	**0.002**
Feb 2021	47	0.30	**0.038**	47	0.23	0.123	47	0.26	0.083	47	0.38	**0.009**
Mar 2021	50	0.33	**0.021**	50	0.21	0.136	50	0.22	0.133	50	0.32	**0.023**
Apr 2021	50	0.31	**0.026**	50	0.15	0.308	50	0.14	0.318	50	0.25	0.086
May 2021	50	0.33	**0.019**	50	0.14	0.329	50	0.14	0.336	50	0.23	0.115
Jun 2021	50	0.32	**0.025**	50	0.14	0.340	50	0.14	0.316	50	0.24	0.090
Jul 2021	50	0.32	**0.024**	50	0.17	0.248	50	0.16	0.254	50	0.24	0.099
Aug 2021	50	0.28	0.052	50	0.16	0.275	50	0.17	0.235	50	0.26	0.068

[a] Spearman Correlation Coefficients.

[b] *P* value indicating correlation is significantly different than zero.

Supplementary Table S48. **Association between people covered by vaccines and infection growth rate from December 2020 to August 2021.**

People covered by vaccines	Infection growth rate														
	Dec 2020			Jan 2021			Feb 2021			Mar 2021			Apr 2021		
	n	r_s[a]	*P* value[b]	n	r_s[a]	*P* value[b]	n	r_s[a]	*P* value[b]	n	r_s[a]	*P* value[b]	n	r_s[a]	*P* value[b]
Dec 2020	27	0.36	0.064	27	0.064	**0.017**	27	-0.15	0.446	27	-0.41	**0.035**	27	-0.39	**0.046**
Jan 2021	37	0.38	**0.021**	37	0.44	**0.007**	37	0.22	0.191	37	-0.05	0.791	37	-0.28	0.092
Feb 2021	47	0.23	0.127	47	0.17	0.242	47	0.24	0.107	47	0.20	0.168	47	-0.16	0.271
Mar 2021	50	0.14	0.341	50	0.06	0.702	50	0.11	0.453	50	0.18	0.220	50	-0.17	0.242
Apr 2021	50	0.12	0.424	50	0.09	0.550	50	0.13	0.377	50	0.15	0.310	50	-0.16	0.272
May 2021	50	0.13	0.361	50	0.06	0.678	50	0.09	0.546	50	0.12	0.410	50	-0.16	0.279
Jun 2021	50	0.14	0.348	50	0.08	0.599	50	0.06	0.673	50	0.08	0.572	50	-0.16	0.258
Jul 2021	50	0.09	0.545	50	0.10	0.502	50	0.07	0.640	50	0.05	0.751	50	-0.18	0.217
Aug 2021	50	0.07	0.628	50	0.11	0.437	50	0.08	0.584	50	0.03	0.841	50	-0.21	0.150

[a] Spearman Correlation Coefficients.

[b] *P* value indicating correlation is significantly different than zero.

People covered by vaccines	Infection growth rate											
	May 2021			Jun 2021			Jul 2021			Aug 2021		
	n	r_s[a]	*P* value[b]	n	r_s[a]	*P* value[b]	n	r_s[a]	*P* value[b]	n	r_s[a]	*P* value[b]
Dec 2020	27	-0.36	0.062	27	-0.25	0.205	27	-0.08	0.692	27	0.08	0.689
Jan 2021	37	-0.49	**0.002**	37	-0.49	**0.002**	37	-0.08	0.647	37	0.29	0.085
Feb 2021	47	-0.35	**0.015**	47	-0.43	**0.003**	47	-0.34	**0.021**	47	-0.19	0.202
Mar 2021	50	-0.46	**0.001**	50	-0.52	**0.0001**	50	-0.37	**0.008**	50	-0.22	0.121
Apr 2021	50	-0.46	**0.001**	50	-0.56	**<.0001**	50	-0.42	**0.002**	50	-0.26	0.071
May 2021	50	-0.45	**0.001**	50	-0.58	**<.0001**	50	-0.43	**0.002**	50	-0.29	**0.044**
Jun 2021	50	-0.42	**0.002**	50	-0.55	**<.0001**	50	-0.40	**0.004**	50	-0.26	0.065
Jul 2021	50	-0.40	**0.004**	50	-0.51	**0.0002**	50	-0.37	**0.009**	50	-0.27	0.060
Aug 2021	50	-0.41	**0.003**	50	-0.49	**0.0003**	50	-0.33	**0.020**	50	-0.20	0.168

[a] Spearman Correlation Coefficients.

[b] *P* value indicating correlation is significantly different than zero.

Supplementary Table S49. **Association between people covered by vaccines and one-month case fatality rate from December 2020 to August2021.**

People covered by vaccines	One-month case fatality rate														
	Dec 2020			Jan 2021			Feb 2021			Mar 2021			Apr 2021		
	n	r_s[a]	*P* value[b]	n	r_s[a]	*P* value[b]	n	r_s[a]	*P* value[b]	n	r_s[a]	*P* value[b]	n	r_s[a]	*P* value[b]
Dec 2020	27	-0.23	0.249	27	-0.04	0.848	27	0.15	0.459	27	-0.01	0.968	27	-0.17	0.401
Jan 2021	37	-0.12	0.466	37	-0.01	0.959	37	0.03	0.883	37	-0.10	0.559	37	-0.23	0.175
Feb 2021	47	0.07	0.660	47	0.03	0.865	47	-0.05	0.735	47	-0.02	0.915	47	-0.03	0.828
Mar 2021	50	0.02	0.873	50	-0.05	0.731	50	-0.14	0.329	50	-0.10	0.472	50	-0.08	0.594
Apr 2021	50	0.05	0.726	50	-0.04	0.774	50	-0.12	0.406	50	-0.10	0.511	50	-0.09	0.526
May 2021	50	0.05	0.734	50	-0.01	0.919	50	-0.11	0.441	50	-0.10	0.497	50	-0.12	0.403
Jun 2021	50	-0.03	0.817	50	-0.08	0.562	50	-0.15	0.295	50	-0.15	0.315	50	-0.19	0.187
Jul 2021	50	-0.07	0.616	50	-0.15	0.301	50	-0.17	0.248	50	-0.16	0.262	50	-0.23	0.105
Aug 2021	50	-0.18	0.207	50	-0.26	0.066	50	-0.27	0.054	50	-0.26	0.067	50	-0.33	**0.018**

[a] Spearman Correlation Coefficients.

[b] *P* value indicating correlation is significantly different than zero.

People covered by vaccines	One-month case fatality rate											
	May 2021			Jun 2021			Jul 2021			Aug 2021		
	n	r_s[a]	*P* value[b]	n	r_s[a]	*P* value[b]	n	r_s[a]	*P* value[b]	n	r_s[a]	*P* value[b]
Dec 2020	27	-0.16	0.421	27	-0.28	0.162	27	-0.21	0.302	27	-0.13	0.517
Jan 2021	37	-0.30	0.067	37	-0.40	**0.013**	37	-0.53	**0.001**	37	-0.58	**0.0002**
Feb 2021	47	-0.13	0.386	47	-0.24	0.104	47	-0.34	**0.018**	47	-0.31	**0.032**
Mar 2021	50	-0.14	0.324	50	-0.25	0.078	50	-0.41	**0.003**	50	-0.42	**0.002**
Apr 2021	50	-0.15	0.312	50	-0.22	0.124	50	-0.38	**0.006**	50	-0.44	**0.001**
May 2021	50	-0.16	0.263	50	-0.22	0.118	50	-0.40	**0.004**	50	-0.45	**0.001**
Jun 2021	50	-0.24	0.091	50	-0.29	**0.039**	50	-0.47	**0.001**	50	-0.51	**0.0001**
Jul 2021	50	-0.31	**0.027**	50	-0.33	**0.019**	50	-0.50	**0.0002**	50	-0.52	**0.0001**
Aug 2021	50	-0.41	**0.003**	50	-0.45	**0.001**	50	-0.60	**<.0001**	50	-0.58	**<.0001**

[a] Spearman Correlation Coefficients.

[b] *P* value indicating correlation is significantly different than zero.

Supplementary Information 15. COVID-19 government responses in border closure

Supplementary Table S50. Comparison of the time to border closure among geographic regions.

	N	Median (IQR)	*P* value[c]
**Time to any border closure from first reported case in China (days)[a]**			
Asia	18	24.50 (24.00)	**0.0003**
Non-Asia	32	67.50 (40.50)	
Americas	8	68.50 (33.00)	0.3207
Non- Americas	42	33.00 (45.00)	
Europe	20	69.00 (25.00)	**0.0054**
Non-Europe	30	30.50 (46.00)	
Oceania	2	32.50 (1.00)	0.8044
Non-Oceania	48	48.00 (47.00)	
Africa	2	51.00 (56.00)	0.7103
Non-Africa	48	36.00 (46.00)	
**Time to any border closure from first case in reference country (days)[b]**			
Asia	18	-10.50 (24.00)	**0.0027**
Non-Asia	32	8.00 (21.00)	
Americas	8	3.00 (14.50)	0.8219
Non-Americas	42	5.00 (36.00)	
Europe	20	14.00 (24.50)	**0.0019**
Non-Europe	30	-2.00 (27.00)	
Oceania	2	-10.00 (32.00)	0.4574
Non-Oceania	48	5.00 (23.50)	

Africa	2	-4.00 (76.00)	0.9408
Non- Africa	48	5.00 (22.50)	

[a] The first reported case in China was 31 December 2019

[b] Reference country is each index country.

[c] The Mann-Whitney U test.

Supplemental References

1. EIU. How well have OECD countries responded to the coronavirus crisis? *The Economist Intelligence Unit*, 2020. https://www.eiu.com/n/campaigns/oecd-countries-responded-to-the-coronavirus-crisis/. Accessed November 25, 2020.
2. OxCGRT. Coronavirus government response tracker. *University of Oxford*, 2020. https://www.bsg.ox.ac.uk/research/research-projects/coronavirus-government-response-tracker. Accessed November 25, 2020.
3. NLI Research Institute. The impact of coronavirus on national economy—which countries survive from the pandemic, the ranking of 49 countries. *NLI Research Institute*; July 3, 2020. https://www.nli-research.co.jp/report/detail/id=64863?site=nli. Accessed November 25, 2020.
4. Fernández-Villaverde J, Jones CI. Macroeconomic outcomes and COVID-19: a progress report. *National Bureau of Economic Research 2020*. https://www.nber.org/papers/w28004. Accessed March 19, 2021.
5. Penghu County Government. A summary of COVID-19 cases in Taiwan. 2020. https://www.penghu.gov.tw/wuhanpneumonia/home.jsp?id=5&act=view&dataserno=202011300007. Accessed March 15, 2020.
6. Lin YH, Liu CH, Chiu YC. Google searches for the keywords of "wash hands" predict the speed of national spread of COVID-19 outbreak among 21 countries. *Brain, Behav, and Immun* 2020; **87:** 30–32. doi: 10.1016/j.bbi.2020.04.020.
7. Lin YH, Chiang TW, Lin YL. Increased internet searches for insomnia as an indicator of global mental health during the COVID-19 pandemic: multinational longitudinal study. *J Med Internet Res* 2020; **22(9):** e22181. doi: 10.2196/22181.
8. Hyndman RJ, Khandakar Y. Automatic time series forecasting: the forecast package for R. *J Stat Soft* 2008; **27(3)**. doi:10.18637/jss.v000.i00.
9. FREOPP. How We Compiled the 2021 FREOPP World Index of Healthcare Innovation. https://freopp.org/wihi-methodology-freopp-world-index-of-healthcare-innovation-10dea21c8c2f. Accessed December 10, 2021.
10. OxCGRT. Methodology for calculating indices. University of Oxford, 2020. https://github.com/OxCGRT/covid-policy-tracker/blob/master/documentation/index_methodology.md. Accessed December 10, 2021.
